# Global, regional, and national burdens of ischemic heart disease and stroke attributable to exposure to long working hours for 194 countries, 2000–2016: A systematic analysis from the WHO/ILO Joint Estimates of the Work-related Burden of Disease and Injury

**DOI:** 10.1016/j.envint.2021.106595

**Published:** 2021-09

**Authors:** Frank Pega, Bálint Náfrádi, Natalie C. Momen, Yuka Ujita, Kai N. Streicher, Annette M. Prüss-Üstün, Technical Advisory Group, Alexis Descatha, Tim Driscoll, Frida M. Fischer, Lode Godderis, Hannah M. Kiiver, Jian Li, Linda L. Magnusson Hanson, Reiner Rugulies, Kathrine Sørensen, Tracey J. Woodruff

**Affiliations:** a Department of Environment, Climate Change and Health, World Health Organization, Geneva, Switzerland; b Labour Administration, Labour Inspection and Occupational Safety and Health Branch, International Labour Organization, Geneva, Switzerland; c UNIV Angers, CHU Angers, Univ Rennes, Inserm, EHESP, Irset (Institut de recherche en santé, environnement et travail) – UMR_S 1085, Angers, France; d AP-HP (Paris Hospital), Occupational Health Unit, Poincaré University Hospital, Garches, France; e Versailles St-Quentin Univ-Paris Saclay Univ (UVSQ), UMS 011, UMR-S 1168, France; f Inserm, U1168 UMS 011, Villejuif, France; g Sydney School of Public Health, Faculty of Medicine and Health, University of Sydney, Sydney, NSW, Australia; h Department of Environmental Health, School of Public Health, University of Sao Paulo, Sao Paulo, SP, Brazil; i Department of Public Health and Primary Care, KU Leuven, Leuven, Belgium; j Eurostat, European Commission, Luxembourg, Luxembourg; k Department of Environmental Health Sciences, Fielding School of Public Health, School of Nursing, University of California Los Angeles, Los Angeles, CA, USA; l Stress Research Institute at Department of Psychology, Stockholm University, Stockholm, Sweden; m National Research Centre for the Working Environment, Copenhagen, Denmark; n Department of Public Health, University of Copenhagen, Copenhagen, Denmark; o Department of Psychology, University of Copenhagen, Copenhagen, Denmark; p Program on Reproductive Health and the Environment, University of California San Francisco, San Francisco, CA, USA

**Keywords:** Burden of disease, Working hours, Ischemic heart disease, Stroke

## Abstract

**Background:**

World Health Organization (WHO) and International Labour Organization (ILO) systematic reviews reported sufficient evidence for higher risks of ischemic heart disease and stroke amongst people working long hours (≥55 hours/week), compared with people working standard hours (35–40 hours/week). This article presents WHO/ILO Joint Estimates of global, regional, and national exposure to long working hours, for 194 countries, and the attributable burdens of ischemic heart disease and stroke, for 183 countries, by sex and age, for 2000, 2010, and 2016.

**Methods and Findings:**

We calculated population-attributable fractions from estimates of the population exposed to long working hours and relative risks of exposure on the diseases from the systematic reviews. The exposed population was modelled using data from 2324 cross-sectional surveys and 1742 quarterly survey datasets. Attributable disease burdens were estimated by applying the population-attributable fractions to WHO’s Global Health Estimates of total disease burdens.

**Results:**

In 2016, 488 million people (95% uncertainty range: 472–503 million), or 8.9% (8.6–9.1) of the global population, were exposed to working long hours (≥55 hours/week). An estimated 745,194 deaths (705,786–784,601) and 23.3 million disability-adjusted life years (22.2–24.4) from ischemic heart disease and stroke combined were attributable to this exposure. The population-attributable fractions for deaths were 3.7% (3.4–4.0) for ischemic heart disease and 6.9% for stroke (6.4–7.5); for disability-adjusted life years they were 5.3% (4.9–5.6) for ischemic heart disease and 9.3% (8.7–9.9) for stroke.

**Conclusions:**

WHO and ILO estimate exposure to long working hours (≥55 hours/week) is common and causes large attributable burdens of ischemic heart disease and stroke. Protecting and promoting occupational and workers’ safety and health requires interventions to reduce hazardous long working hours.

## Highlights

• We present the first WHO/ILO Joint Estimates of the Work-related Burden of Disease and Injury.
• Globally in 2016, 488 million people were exposed to long working hours (≥55 hours/week).
• This exposure had 745,194 attributable deaths and 23.3 million DALYs from ischemic heart disease and stroke.
• These are 4.9% of all deaths and 6.9% of all DALYs from these causes.
• The Western Pacific, South-East Asia, men, and older people carried higher burdens.

## 1 Introduction

The protection and promotion of occupational and workers’ safety and health requires actions to prevent exposures to occupational risk factors. One such occupational risk factor is exposure to long working hours. The very first International Labour Standard, *Hours of Work (Industry) Convention, 1919 (No. 1)* (1st International Labour Conference, 1919), and several other international labour standards (International Labour Organization, 2019a) adopted since have limited hours of work. In 2019, countries renewed their commitment to ensuring maximum limits on working time in the *Centenary Declaration on the Future of Work* (International Labour Organization, 2019b).

The *Hours of Work (Industry) Convention* provides that the working hours of employed persons shall not exceed 8 hours/day and 48 hours/week (with some exceptions). In some countries, however, the definition of long working hours depends on national regulations. Nevertheless, many countries define standard working hours as 35–40 hours/week and working ≥41 hours/week as overtime work (International Labour Organization, 2017). Occupational epidemiologists often categorize long working hours into the three analytical categories of 41–48, 49–54, and ≥55 hours/week, and compare these to standard working hours (35–40 hours/week) (Descatha et al., 2020, Li et al., 2020a). After average working time decreased steadily over the second half of the 20th century in most countries, this overall downward trend ceased and even began to reverse in some countries during the 21st Century (Messenger, 2018). As new information and communication technologies revolutionize work, working time is predicted to further increase for some industries (Messenger, 2018).

**Fig. 1:**
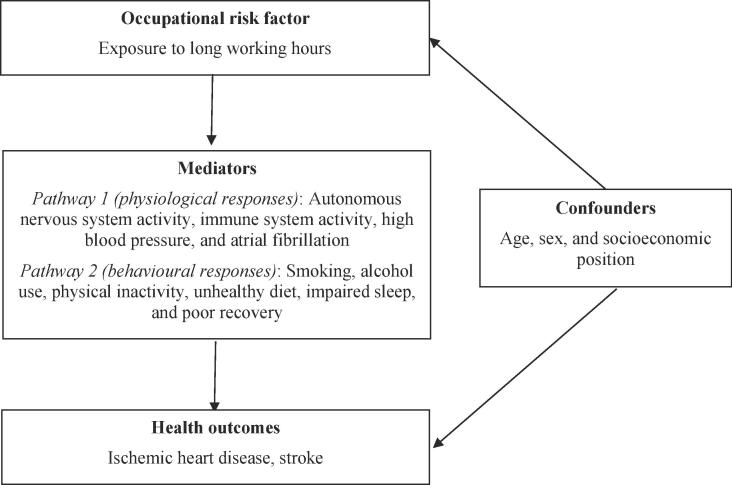
Conceptual model of the possible causal relationship between exposure to long working hours and ischemic heart disease and stroke. Footnote: Adapted from Li et al. (2020a) and Descatha et al. (2020). Some variables in this conceptual model, such as smoking and physical inactivity, could be confounders, mediators or both at the same time.

Evidence from previous studies suggests working long hours can increase mortality and morbidity from ischemic heart disease and stroke through psychosocial stress. Two primary causal pathways are conceivable (Fig. 1). The first is through biological responses to psychosocial stress: release of excessive stress hormones due to working long hours (Chandola et al., 2010, Jarczok et al., 2013, Nakata, 2012) may trigger functional dysregulations in the cardiovascular system and structural lesions (Kivimäki and Steptoe, 2018). The second pathway is through behavioral responses to stress that are established cardiovascular risk factors, including tobacco use, harmful alcohol use, unhealthy diet, physical inactivity, and resultant impaired sleep (Sonnentag et al., 2017, Taris et al., 2011, Virtanen et al., 2009).

Some long working hours categories are associated with a higher risk of ischemic heart disease and stroke events. The World Health Organization (WHO) and the International Labour Organization (ILO), supported by large Working Groups of individual experts, have conducted systematic reviews and meta-analyses of the relative risks of ischemic heart disease (Li et al., 2020a) and stroke (Descatha et al., 2020) among persons working 41–48, 49–54, and ≥55 hours/week, compared with persons working 35–40 hours/week (Table 1). Confounding was considered by, at least, sex, age, and an indicator of socioeconomic status (SES; e.g., income, education, or occupational grade). The Working Groups of individual experts judged there to be sufficient evidence that working ≥55 hours/week is associated with a higher risk of both ischemic heart disease and stroke, compared with working 35–40 hours/week (Table 1). To our knowledge, these are the first systematic reviews on these topics that are based on pre-published protocols (Descatha et al., 2018, Li et al., 2018); applied a dedicated systematic review framework (the Navigation Guide; Woodruff et al., 2011); and formally proceeded to assess quality and strength of evidence based on pre-published criteria and methods.

**Table 1:** Bodies of evidence from systematic reviews and meta-analyses on the effect of exposure to long working hours on ischemic heart disease and stroke, by long working hours category.

Health outcome	Long working hours category	Number of studies in meta-analysis (participants)	Relative risk (95% confidence interval)	Navigation Guide rating of quality of evidence (Woodruff et al., 2011)^a^	Navigation Guide rating of strength of evidence for human data (Woodruff et al., 2011)^b^	Evidence judged as sufficient (as per Ezzati et al., 2002) to proceed to estimation for this category
**Ischemic heart disease (**Li et al., 2020a**)**^c^	41–48 hours/week	20 studies (312,209 participants)	0.99 (0.88–1.12)	Low quality	Inadequate evidence of harmfulness	No
	49–54 hours/week	18 studies (308,405 participants)	1.01 (0.82–1.25)	Low quality	Inadequate evidence of harmfulness	No
	≥55 hours/week	22 studies (339,680 participants)	1.17 (1.05–1.31)	Moderate quality	Sufficient evidence of harmfulness	Yes
**Stroke (**Descatha et al., 2020**)**^d^	41–48 hours/week	12 studies (265,937 participants)	1.04 (0.94–1.14)	Low quality	Inadequate evidence of harmfulness	No
	49–54 hours/week	17 studies (275,181 participants)	1.13 (1.00–1.28)^e^	Moderate quality	Limited evidence of harmfulness	No
	≥55 hours/week	7 studies (162,644 participants)	1.35 (1.13–1.61)	Moderate quality	Sufficient evidence of harmfulness	Yes

a Navigation Guide quality of evidence ratings (Woodruff et al., 2011): *High quality* = Further research is very unlikely to change our confidence in the estimate of effect. *Moderate quality* = Further research is likely to have an important impact on our confidence in the estimate of effect and may change the estimate. *Low quality* = Further research is very likely to have an important impact on our confidence in the estimate of effect and is likely to change the estimate.

b Navigation Guide ratings of strength of evidence (Woodruff et al., 2011): *Sufficient evidence of harmfulness*: The available evidence usually includes consistent results from well‐designed, well‐conducted studies, and the conclusion is unlikely to be strongly affected by the results of future studies. For human evidence a positive relationship is observed between exposure and outcome where chance, bias, and confounding, can be ruled out with reasonable confidence. *Limited evidence of harmfulness*: The available evidence is sufficient to determine the effects of the exposure, but confidence in the estimate is constrained by such factors as: the number, size, or quality of individual studies, the confidence in the effect, or inconsistency of findings across individual studies. As more information becomes available, the observed effect could change, and this change may be large enough to alter the conclusion. For human evidence a positive relationship is observed between exposure and outcome where chance, bias, and confounding cannot be ruled out with reasonable confidence. *Inadequate evidence of harmfulness*: Studies permit no conclusion about a toxic effect. The available evidence is insufficient to assess effects of the exposure. Evidence is insufficient because of: the limited number or size of studies, low quality of individual studies, or inconsistency of findings across individual studies. More information may allow an estimation of effects. *Evidence of lack of harmfulness*: The available evidence includes consistent results from well‐designed, well‐conducted studies, and the conclusion is unlikely to be strongly affected by the results of future studies. For human evidence more than one study showed no effect on the outcome of interest at the full range of exposure levels that humans are known to encounter, where bias and confounding can be ruled out with reasonable confidence. The conclusion is limited to the age at exposure and/or other conditions and levels of exposure studied.

c A prior decision was made to select as input for modelling disease burden the “best” effect estimate (relative risks for morbidity versus mortality) based on strength of evidence ratings. When strength of evidence was rated sufficient for mortality, the relative risk for mortality was used. When the strength of evidence was considered inadequate for mortality, but sufficient for morbidity, the relative risks for morbidity were used. For ischemic heart disease, strength of evidence was rated sufficient for ischemic heart disease mortality, therefore relative risk for mortality was used.

d For stroke, strength of evidence was rated insufficient for stroke mortality but sufficient for stroke morbidity, therefore relative risk for morbidity was used.

e P = 0.04.

Countries, along with relevant international and regional organizations, and social partners, would benefit from accurate and transparent estimates of long working hours exposure and the attributable burden of disease. These provide the evidence base for designing, developing, planning, costing, implementing, and evaluating legislation, regulations, policies, programmes, and interventions to mitigate occupational risk factor exposure and its attributable disease burden. This article presents WHO/ILO Joint Estimates of the Work-related Burden of Disease and Injury (WHO/ILO Joint Estimates). These are estimates of exposures to long working hours (≥55 hours/week), for 194 countries, and those of the burdens of ischemic heart disease and stroke attributable to these, for 183 countries. All estimates are reported at the global, regional (WHO regions), and national levels, by sex and age group, for the years 2000, 2010, and 2016.

## 2 Materials and methods

### 2.1 Overview

WHO and ILO produced the estimates as part of the global Comparative Risk Assessment (e.g., World Health Organization (2016)), which systematically estimates health burden arising from exposure to risk factors at levels above a counterfactual distribution (Murray et al., 2004). We developed definitions of the occupational risk factor, risk factor levels, and theoretical minimum risk exposure level (Table S1 in Supplementary data 1). Estimates of attributable burden were based on combining the distribution of exposure in the population with relative risks of health outcomes due the effects of exposure. This combination is then used to calculate the population-attributable fractions (PAFs) (Murray et al., 2004). PAFs quantify the proportion of death or disease that is attributable to a specific risk factor, and, therefore, the respective reduction that would be expected if exposure was removed or present at a reduced level. Estimates were produced and are reported for populations defined by country, sex (i.e., three categories: both sexes, female, and male), and age group (i.e., 18 categories: ≥15, 15–19, 20–24, …, 90–94, ≥95 years). The estimates are reported according to the *Guidelines for Accurate and Transparent Health Estimates Reporting* (GATHER) (Stevens et al., 2016). The complete script used to generate the estimates in the computer software R can be found in Supplementary data 2.

### 2.2 Data sources

The estimates were produced using six sets of input data. Details of these and their sources are shown in Table 2. An overview of how data sources, input data, and models were combined to produce model outputs and burden estimates is provided in Fig. S1 (Supplementary data 1). Additionally, Tables S2–S5 provide detailed descriptions of the databases compiled from surveys (see Supplementary data 1). Briefly, data on the proportions exposed were obtained from Input Data 1 and 2 (databases compiled from survey data); estimates of the total population (Input Data 3) and the probability of death (Input Data 4) came from the United Nations population prospect (United Nations, 2019) and WHO life tables (World Health Organization, 2020), respectively; estimates of relative risks of the association between exposure to long working hours and the two health outcomes (Input Data 5) came from the WHO/ILO systematic reviews (Descatha et al., 2020, Li et al., 2020a); and estimates of the total number of deaths and disability-adjusted life years (DALYs) for ischemic heart disease and stroke by country, sex, and age group for the years 2000, 2010, and 2016 (Input Data 6) were sourced from the WHO Global Health Estimates (World Health Organization 2018a).

**Table 2:** Description of input data and data sources.

**Input Data**	**Source data**	**Details about data extracted**
**1. Cross-sectional data of proportion of survey participants in working hours categories**	• WHO/ILO Cross-sectional Global Working Hours Database • 467 million observations from 2324 surveys conducted in 154 countries between 1 January 1976 and 31 December 2018 (Tables S2 and S3; Supplementary data 1) • Official surveys (primarily Labour Force Surveys which target all working-age population of a country) collected by national statistical offices and shared by countries with ILO or WHO (96.4%) • Gallup surveys (3.6%) shared by countries with ILO (used if no official surveys were available for a country)	• Microdata on the total number of hours usually or actually worked per week in main job (and second job, if held) • Official data on working hours were generally collected in line with the ILO resolution concerning the measurement of working time (International Labour Organization, 2008) • Microdata were harmonized into six working hours categories: 0 hours/week (labour market inactive) (coded as 0); 0–34 hours/week (1), 35–40 hours/week (2), 41–48 hours/week (3), 49–54 hours/week (4), and ≥55 hours/week (5) • Data were weighted using the survey weights produced by countries • Aggregated by population defined by country, year, sex, and age group • All workers are covered except unpaid domestic workers
**2. Longitudinal data on proportion of survey participants per working hours category and labour force activity category**	• WHO/ILO Global Longitudinal Working Hours Database • 155 million observations from 1742 quarterly data sets of Labour Force Surveys conducted in 46 countries between 1 January 2000 and 31 December 2018 (Tables S4 and S5; Supplementary data 1) • Official surveys conducted by national statistical offices and shared by countries with ILO or Eurostat	• Repeated measures from the same survey available for participants over consecutive years • Because the microdata did not include individual participant identifiers, data were probabilistically linked longitudinally, with matching by household number, household sequence number, sex, and birth year • Microdata on the number of working hours were extracted; harmonized into the standard working hours categories; weighted using survey weights; and aggregated by population defined by country, year, sex, and age group • Data on labour force activity were also extracted and included
**3. Estimates of the total population**	United Nations latest global population estimates (United Nations, 2019)	Estimates of the total number of population by country, year, sex, and age group for the years 1950–2018
**4. Estimates of probability of death**	WHO life tables (World Health Organization, 2020)	Estimates of probability of death due to ischemic heart disease and stroke by country, year, sex, and age group
**5. Estimates of relative risks**	WHO/ILO systematic reviews and meta-analyses (Descatha et al., 2020, Li et al., 2020a)	These concluded sufficient evidence of associations of working ≥55 hours/week with higher risks of both ischemic heart disease and stroke (Table 1)
**6. Estimates of total number of deaths and DALYs**	WHO Global Health Estimates (World Health Organization, 2018a)	Estimates of total number of deaths and DALYs for ischemic heart disease and stroke by country, sex, and age group for the years 2000, 2010, and 2016

The WHO/ILO systematic reviews (Descatha et al., 2020, Li et al., 2020a) generated estimates of relative risk of the effect of exposure to long working hours on both morbidity (disease incidence) and mortality, which were used to produce estimates of burden of disease (Fig. S1; Supplementary data 1). As in other burden of disease studies, we only proceeded to estimation if the effect estimate identified in the systematic review was statistically significant (P < 0.05) and the strength of evidence was rated as “sufficient evidence for harmfulness” (Ezzati et al., 2002). We applied the following *a priori* criteria to select the “best” effect estimate (relative risks for morbidity versus mortality) based on strength of evidence ratings: First, if there is any evidence for fatal or non-fatal events of the outcome that was rated as “sufficient evidence for harmfulness” (using standard Navigation Guide ratings as per Woodruff et al., 2011), proceed to selection of “best” estimate. Second, if there is such sufficient evidence:

• *either* only for fatal events *or* only for non-fatal events of the outcome (i.e. not both), then the relative risk for the event type with sufficient evidence is selected.
• in the event that both fatal *and* non-fatal events have sufficient evidence, estimates for fatal events are prioritized.

For exposure to ≥55 hours/week and ischemic heart disease, strength of evidence was rated “sufficient evidence of harmfulness” for mortality (Li et al., 2020a and Table 1), so the relative risk for fatal events (RR 1.17, 95% confidence interval 1.05–1.31) was used as the best estimate. For exposure to ≥55 hours/week and stroke, the strength of evidence was considered “inadequate evidence of harmfulness” for mortality, but “sufficient evidence for harmfulness” regarding morbidity (Descatha et al., 2020 and Table 1); the relative risk for non-fatal events (RR 1.35, 95% confidence interval 1.13–1.61) was therefore the best estimate we used.

Because there was no evidence for effect modification by country, sex, and age group, neither with regard to ischemic heart disease (Li et al., 2020a) nor with regard to stroke (Descatha et al., 2020), the best estimate was assigned to all cohorts defined by country, sex, and age.

### 2.3 Statistical modelling

The estimation strategy comprised modelling the input data in four distinct models that consecutively built on each other (Models 1–4). These are described below.

#### 2.3.1 Model 1: Multilevel model to estimate proportion of population in each working hours category by year

For each year between 1980 and 2016, for each population defined by country, sex, and age group, we produced estimates of the proportion of the population in each of the six standard working hours categories. Because we had microdata on the number of hours worked (direct exposure data), we produced these estimates without needing to consider proxies (e.g., occupation and number of years of labour market activity in the population). As for other WHO estimates (Bonjour et al., 2013, Wolf et al., 2013, Wolf et al., 2019), we modelled this proportion based on Input Data 1 using an established multilevel model that predicts the prevalence over time and in the geographical region (Goldstein, 2010, Leyland and Goldstein, 2001). This method is used by WHO to produce Sustainable Development Goal indicators (e.g., indicator 3.9.1; see https://unstats.un.org/sdgs/metadata/files/Metadata-03–09-01.pdf) and has therefore passed the approval of the United Nations Statistical Commission. It creates continuous estimates over the specified time period and estimates an average intercept and an average slope with residual variances across countries. Where there is reliable survey information of a specific country, the country curve will closely follow the data; where there are few country data points or high within-country variability, estimates will be close to survey data points, but the trend will tend to follow the overall mean.

The model used is described below (Model 1):

$${\mathrm{proportion}}_{i}=A_{i}\left(sex,age,country\right)+B_{i}\left(sex,age,country\right)*year$$

where *proportion_i_* is the proportion of the population in the working hours category *i* in a given population defined by country, sex, and age group; *year* is the survey year; and *sex*, *age*, and *country* are the sex, age group, and country of the population. *A_i_(sex, age, country)* and *B_i_(sex, age, country)* of the year, dependent on *sex*, *age*, and *country*, are estimated using a multilevel model with country as fixed effects, and with *sex* and *age* as random effects, nested in the *country* within the region (with regions treated independently). Because *proportion_i_* was strongly non-linearly dependent on *age*, we linearized *age* by 5th order orthogonal polynomials to prevent collinearity. For countries and years without any data on long working hours, estimates were produced using multilevel modelling based on data from countries with such data.

#### 2.3.2 Model 2: Model of transition probabilities between working hours categories

For each population defined by country, sex, and age group, we estimated the probability (${\mathrm{probability}}_{j}$) of transitioning from one of the six working hours categories in year_t_ to one specific working hours category in year_t+1_. The *j* denotes one of 36 possible transitions. We adopted the methods developed by Eurostat for calculating these transition probabilities (Kiiver and Espelage, 2016). Using Input Data 2, we scaled the survey weights for the target year (year_t+1_) to represent the correct labour market status by country, sex, and age group for the index year (year_t_) and the target year and then adjusted the complete sample in the target year to match margins for labour market status in both years, using iterative raking by sex. We modelled the probability based on Input Data 2 using the following multinomial logit regression model (Model 2):

$${\mathrm{probability}}_{j}=\frac{e^{\beta_{j}X_{j}}}{1+\sum_{\alpha }e^{\beta_{\alpha }X_{j}}}$$

where $\beta_{j}$ is the set of regression coefficients describing the longitudinal weights associated with the transition *j*; $X_{j}$ is a set of explanatory variables (sex, and age as a 2-degree fractional polynomial associated with the transition *j*); and the summation (index α) goes through all possible transitions of *j* (except the transition from *i* = 0 in year_t_ to *i* = 0 in year_t+1_, which was chosen as a pivot outcome).

With Model 2, WHO and ILO derived 15,900 transition probabilities for the 15 countries with data in Input Data 2. In addition, Eurostat derived 31,104 transition probabilities covering 27 countries based on sub-samples of the European Union Labour Force Surveys using Model 2, and it shared these transition probabilities with WHO and ILO. For populations defined by country, sex, and age group for whom probability_i_ could not be calculated because the required longitudinal data were unavailable, probability_i_ was imputed. The imputed probability_i_ was the mean of all transition probabilities of the population defined by the same sex and age in the region, weighted by the number of observations contributing to the transition probabilities.

#### 2.3.3 Model 3: Microsimulation model to estimate exposed population over a time window

For each population defined by country, sex, and age group, we estimated the proportion (${\mathrm{proportion}}_{k}$) of the population in each working hours category (*k*) over the time window of exposure. We defined *k* as the highest working hours category *i* in any year in the time window. Our modelling assumptions and the evidence supporting these are presented in Table 3. The approach assumed a 10-year latency between exposure and occurrence of clinical disease, and it adopted a 10-year exposure time window (centred on exposure year) to identify the highest exposure category to assign to the relevant exposure year (Fig. S2; Supplementary data 1). For example, to estimate the burden in 2016, the relevant exposure year is 2006; we assume the exposure for a person is the highest exposure category to which this person belonged during the time window of exposure, 2001–2010. In effect, this considers occupational turnover, with someone exposed at any time during the 10-year window considered at-risk for 2006.

**Table 3:** Modelling assumptions and their evidence bases.

	Variable	Assumption in main analysis (sensitivity analyses)	Explanation	Example for burden of disease estimates for the year 2016	Evidence base
1	Lag time (*a*)	Assumed 10 years (assumed 8 and 12 years)	For an outcome event in year_t_, the exposure is assumed to have occurred in the lag year (year_t-a_)	Burden of disease in 2016 is attributable to exposure 10 years earlier, with the lag year being 2006	From a theoretical viewpoint, lag time would vary, depending on the mechanism via which long working hours are associated with ischemic heart disease and stroke. The mechanism could be: • Direct – exposure to long working hours has a direct effect on pathophysiology; • Indirect – exposure to long working hours impacts risk factors for ischemic heart disease and/or stroke; • Trigger – exposure to long working hours trigger events which lead to ischemic heart disease and/or stroke events; and/or • Prognostic factor – exposure to long working hours affects prognosis of coronary heart disease or cerebrovascular disease (Kivimäki and Steptoe, 2018, Steptoe and Kivimäki, 2012). Depending on which of the four mechanisms is the dominant one, the lag time would vary. If direct and indirect effects are the dominant mechanisms, then the lag time could be less than 10 years, whereas if the exposure acts as a trigger or a prognostic factor, 10 years would be too long. However, if all four mechanisms contribute to risk of cardiovascular disease, an average lag of 10 years is an appropriate assumption. Additionally, there are previous examples of the use of lag times of around 10 years: • The studies included in the WHO/ILO systematic reviews and meta-analyses on ischemic heart disease and stroke on average assumed a lag or follow-up time of 10 years (Descatha et al., 2020, Li et al., 2020a). • Mean follow-up times in previous large systematic reviews and individual studies were around 9 and 8 years for ischemic heart disease and stroke, respectively (Hannerz et al., 2018, Kivimäki et al., 2015). • Other studies have suggested an incubation period of at least 10 years for coronary heart disease (Rose, 1982). According to evidence from the CONSTANCES Cohort Study in France, 10 years after exposure to long working hours increased odds ratios of ischemic heart disease and stroke were found, but not before this (Fadel et al., 2020, Fadel et al., 2019).
2	Time window of exposure (*b*)	Assumed 10 years (assumed 8 and 12 years)	Rather than occurring in year_t-a_ only, exposure occurs in any year during a “critical” time window of the length *b*, and exposure within any year in this time window can still cause the disease outcome in year_t_	To estimate burden of disease in 2016, we model exposure over a 10-year time window	As mentioned for assumption 1, the four potential mechanisms (Kivimäki and Steptoe, 2018, Steptoe and Kivimäki, 2012) are likely to have different lag times. A time window of exposure around the lag year accounts for some of this variability. Previous occupational burden of disease studies have also estimated exposure over a time window (Dalboge et al., 2018, Rushton et al., 2012). Evidence suggests that exposure (sometimes measured cumulatively) to long working hours during this 10-year time window contributes to a significant increase in cardiovascular disease incidence (Fadel et al., 2020, Fadel et al., 2019, Rose, 1982).
3	Spacing of time window (*b*) around lag year (year_t-a_)	Spaced the time window symmetrically around the lag year (no sensitivity analysis)	The time window of the exposure is equally spaced around the “lag year” of the average lag period (year_t-a_), so that the time window of the exposure is defined as between year_t-a-b/2_ and year_t-a+b/2_	To estimate burden of disease in 2016, we model exposure over the time window of 2001–2010	As mentioned for assumption 1, there are four potential mechanisms (Kivimäki and Steptoe, 2018, Steptoe and Kivimäki, 2012). With the exception of the trigger mechanism, symmetrical spacing of the time window around the lag year is a reasonable assumption. If all four mechanisms are at play, symmetrical spacing is the most appropriate model. This is also common practice in studies estimating burden of disease attributable to exposure to occupational risk factors; for example, a report on the burden of occupational cancer in the United Kingdom of Great Britain and Northern Ireland estimated the “peak latency” period for their outcomes of interest and spaced the time window of exposure symmetrically around this point (Rushton et al., 2012).
4	Assignment of exposure category (or level)	Assigned the highest exposure category in any year over the time window of exposure (assigned the most common exposure category)	For each worker, the highest exposure category they had in any year over the time window is assigned as their exposure category over the window	Over the years 2001–2010, Worker A was exposed to working ≥55 hours/week in 2001 and 2002, and to 49–54 hours/week in 2003–2011, and therefore we assign Worker A the exposure category of ≥55 hours/week	For diseases with long latency periods, which is possible for cardiovascular disorders, once the disease process has started, the worker continues to be at risk, even if exposure levels are reduced. The assignment of the highest level of exposure observed over the time window is in line with assumptions made by other studies focusing on the effect of long working hours and ischemic heart disease and stroke (Fadel et al., 2020, Fadel et al., 2019, Gardner et al., 1993, Hannerz et al., 2018, Kivimäki et al., 2015, Meyers et al., 2013).
5	Assignment of effect estimate	Assigned the “best” effect estimate	For estimating numbers of deaths and DALYs and for all cohorts defined by country, sex, and age group, we assigned the same “best” effect estimate (no sensitivity analysis)	To estimate burden of disease, in 2016 for each cohort defined by country, sex, and age group, we used the pooled effect estimate	There is no evidence for effect modification by country (or WHO region), sex or age group in the subgroup analyses in the WHO/ILO systematic reviews (Descatha et al., 2020, Li et al., 2020a). Therefore, we assigned the pooled effect estimate from the main analysis.This is the same approach used in previous WHO burden of disease studies (Murray et al., 2004). We systematically selected the “best” effect estimate, based on our pre-specified criteria (see Section 2.2). This is based on prioritizing mortality over morbidity and relatively higher strength of evidence over lower strength of evidence (Section 2.2 and Table 1).

We used microsimulation, a method for generating micro-level estimates by combining individual- and aggregate-level datasets. For each country, we initiated a synthetic population, using Input Data 3 to ensure a representative sex and age distribution at the first year of the time window (i.e. estimation year minus 15 years), and estimates outputted from Model 1 to probabilistically assign each individual to a working hours category (*i*) at the first year. Using transition probabilities outputted in Model 2, for each year over the entire time window, transitions from one working hours category to another were stochastically modelled to estimate each synthetic individual’s working hours category in each year. Using Input Data 4, at each year step from the first year of the time window to the estimation year, each individual is stochastically assigned to the states of “died” or “alive”. All synthetic individuals that reach the state “died” before the estimation year are censored. Other possible exits from the population, such as outmigration from the country, were not considered. Using this microsimulation method, ${\mathrm{proportion}}_{k}$ is derived using the following model (Model 3):

$${\mathrm{proportion}}_{k}=\frac{\sum_{l=1..n}\delta_{k,max\left(S_{l}\right)}}{n}$$

where the summation runs through individuals “alive” in the estimation year; $\delta_{k}$ is the Kronecker delta function; *S_l_* is the sequence of the *l*^th^ individual of all working hours categories (*i*) in each year in the time window; and max denotes that the highest *i* that the *l*^th^ individual experiences in the sequence is assigned.

#### 2.3.4 Model 4: Burden of disease estimation model

The Comparative Risk Assessment framework (Murray et al., 2004) is used to estimate the burden of disease attributable to exposure to long working hours. We estimate the proportional reduction in death or disease that would occur if exposure was reduced to a level with a minimum risk (i.e. working 35–40 hours/week), while other conditions remain unchanged. The population distribution of exposure to the risk factor is combined with the increased risk of acquiring the disease or dying fromit that was attributable to exposure to the risk factor.

Using estimates outputted from Model 3 and Input Data 5, we calculated the population-attributable fraction (PAF), the proportion of the health loss seen in a given population that can be attributed to exposure to the specific occupational risk factor, using Model 4:

$$\mathrm{PAF}=\frac{\sum_{k=1}^{n}P_{k}\left({\mathrm{RR}}_{k}-1\right)}{\sum_{k=1}^{n}P_{k}\left({\mathrm{RR}}_{k}-1\right)+1}$$

where *P_k_* is the proportion of the population in a working hours category *k*; *RR_k_* is the relative risk for the working hours category *k*; and *n* is the total number of long working hours categories. Applying this fraction to the total disease burden (Input Data 6), gives the population attributable disease burden. To be specific, for each cause and risk factor category, the PAF was calculated for each cohort defined by country, sex, and age group; this was then applied to the total disease burden envelope for the cause also by country, sex, and age cohort to get the respective burden for this specific cohort. As there is no evidence to suggest that the relative risk for mortality and morbidity is different, we assume the PAFs are the same for non-fatal and fatal health events, and apply the same individual cohort-level PAFs to generate numbers of deaths and DALYs (Ezzati et al., 2004); however, once individual cohorts are combined (e.g., to give regional, global, all ages or both sexes), resulting PAFs for deaths and DALYs will differ due to the different envelopes.

### 2.4 Uncertainty ranges

When calculating burden of disease, several sources of uncertainty may exist, including selection bias, confounding, and statistical error. Consistent with previous global health estimates (Bonjour et al., 2013, de Onis et al., 2004, Wolf et al., 2013, Wolf et al., 2019), for all estimates (i.e., exposure, PAF, death, and DALYs), we used bootstrapping to derive uncertainty (or prediction) ranges at the 2.5th and 97.5th percentiles of the resulting random deviates (Efron, 1979). Uncertainty in input parameters was propagated across all four models. The relative risks were log normally distributed (Descatha et al., 2020, Li et al., 2020a) and modelled using this distribution; all other input parameters were assumed to be normally distributed and modelled accordingly. Uncertainty ranges of estimates for combined cohorts (e.g., regional, global, all age groups, and both sexes) were produced using the method described in de Onis et al. (2004), as done in previous official estimations (Bonjour et al., 2013, Wolf et al., 2013, Wolf et al., 2019). For each estimate, we report the point estimate and its 95% uncertainty range (UR).

### 2.5 Sensitivity analyses

To test the assumptions made for year 2016 (i.e., lag time of 10 years; time window of 10 years from 2001 to 2010; and assignment of the highest exposure category over the time window), we conducted the following sensitivity analyses:

• Reduced the lag time to 8 years (2003–2012);
• Increased the lag time to 12 years (1999–2008);
• Reduced the time window for the exposure to 8 years (2002–2009);
• Increased the time window to 12 years (2000–2011); and
• Assigned the long working hours category with the largest number of years in the time window (censoring years spent in labour market inactivity).

## 3 Results

Global health estimates for 2016 are summarized in this article. Estimates for 2000 and 2010 are found in Table 4, Table 5, regional estimates are displayed in Tables S6–S10 in Supplementary data 1, and country estimates are available on www.who.int/teams/environment-climate-change-and-health/monitoring/who-ilo-joint-estimates/ and http://www.ilo.org/global/topics/safety-and-health-at-work/. Here, regional estimates are reported for the six WHO regions (Africa, Americas, Eastern Mediterranean, Europe, South-East Asia, and Western Pacific); however, with estimates for each country available, estimates could be based on any regional clustering, including the five ILO regions (Africa, Americas, Arab States, Asia and the Pacific, and Europe and Central Asia).

**Table 4:** Proportion of population exposed to long working hours (≥55 hours/week), 2000, 2010, and 2016, and mean percentage change for 2000–2010, 2010–2016, and 2000–2016, by sex, 194 countries.

	**Exposed (%) (UR)**			**Percent change (UR)**		
	2000	2010	2016	2000–2010	2010–2016	2000–2016
**Both sexes**	8.1 (7.8–8.4)	8.3 (8.1–8.6)	8.9 (8.6–9.1)	3.0 (−1.2–7.7)	6.0 (1.8–10.5)	9.3 (4.3–14.6)
**Males**	11.8 (11.3–12.3)	12.3 (12.0–12.7)	13.2 (12.7–13.7)	4.4 (−0.7–9.7)	7.2 (2.1–12.4)	11.8 (5.9–18.1)
**Females**	4.4 (4.1–4.7)	4.4 (4.1–4.6)	4.5 (4.2–4.8)	−0.8 (−9.2–8.6)	2.8 (−5.5–11.5)	1.9 (−7.0–12.0)

Footnote: UR = 95% uncertainty range.

**Table 5:** Number of deaths and DALYs (in ‘000s) from ischemic heart disease and stroke, attributable to exposure to long working hours, 2000, 2010, and 2016, and mean percentage change for 2000–2010, 2010–2016, and 2000–2016, by sex, 183 countries.

**Outcome**		**Both sexes**						**Males**						**Females**					
		**Number (UR)**			**Percent change (UR)**			**Number (UR)**			**Percent change (UR)**			**Number (UR)**			**Percent change (UR)**		
		2000	2010	2016	2000–2010	2010–2016	2000–2016	2000	2010	2016	2000–2010	2010–2016	2000–2016	2000	2010	2016	2000–2010	2010–2016	2000–2016
**Ischemic heart disease**^a^	Deaths	244,983 (229,742–260,225)	304,344 (282,840–325,849)	346,753 (319,658–373,848)	24.2 (12.8–36.5)	13.9 (2.4–26.6)	41.5 (27.9–56.5)	186,791 (172,463–201,119)	229,520 (209,383–249,657)	262,754 (237,265–288,242)	22.9 (9.3–38.0)	14.5 (0.2–30.6)	40.7 (24.3–59.2)	58,192 (52,995–63,389)	74,825 (67,277–82,372)	83,999 (74,808–93,190)	28.6 (12.4–46.9)	12.3 (−3.4–30.3)	44.4 (25.1–66.0)
	DALYs (in ‘000)	7548 (7108–7988)	9368 (8740–9997)	10,655 (9874–11,437)	24.1 (13.3–35.8)	13.7 (2.9–25.5)	41.2 (28.5–54.6)	5828 (5413–6243)	7146 (6555–7736)	8156 (7418–8894)	22.6 (9.5–37.0)	14.1 (0.5–29.2)	40.0 (24.3–56.8)	1720 (1572–1868)	2223 (2006–2439)	2499 (2242–2756)	29.2 (13.5–47.3)	12.4 (−2.5–29.6)	45.3 (26.6–66.4)
**Stroke**^a^	Deaths	334,855 (312,689–357,020)	366,685 (342,692–390,679)	398,441 (369,826–427,056)	9.5 (−0.2–20.1)	8.7 (−1.3–19.7)	19.0 (7.8–31.1)	229,596 (210,080–249,111)	252,523 (231,472–273,574)	276,098 (250,773–301,422)	10.0 (−2.5–23.9)	9.3 (−3.4–23.8)	20.3 (5.8–36.3)	105,259 (94,749–115,769)	114,163 (102,651–125,674)	122,343 (109,021–135,665)	8.5 (−6.0–25.3)	7.2 (−7.6–24.0)	16.2 (0.3–35.1)
	DALYs (in ‘000)	10,353 (9759–10,947)	11,471 (10,814–12,128)	12,603 (11,817–13,390)	10.8 (2.2–20.2)	9.9 (0.7–19.8)	21.7 (11.8–32.4)	7050 (6533–7567)	7825 (7257–8393)	8629 (7944–9314)	11.0 (0.1–23.3)	10.3 (−0.8–22.3)	22.4 (9.7–36.3)	3303 (3012–3595)	3646 (3316–3976)	3974 (3588–4360)	10.4 (−2.7–25.5)	9.0 (−4.5–24.4)	20.3 (5.4–37.3)

Footnotes: No estimates were produced for Andorra, Cook Islands, Dominica, Marshall Islands, Monaco, Nauru, Niue, Palau, Saint Kitts and Nevis, San Marino, and Tuvalu, because the envelopes for the burdens of ischemic heart disease and stroke were unavailable for these countries (World Health Organization 2018b).

a The effects of working ≥55 hours/week, compared with standard working hours of 35–40 hours/week, were modelled. UR = 95% uncertainty range.

### 3.1 Estimates of population exposed to long working hours (≥55 hours/week)

**Fig. 2:**
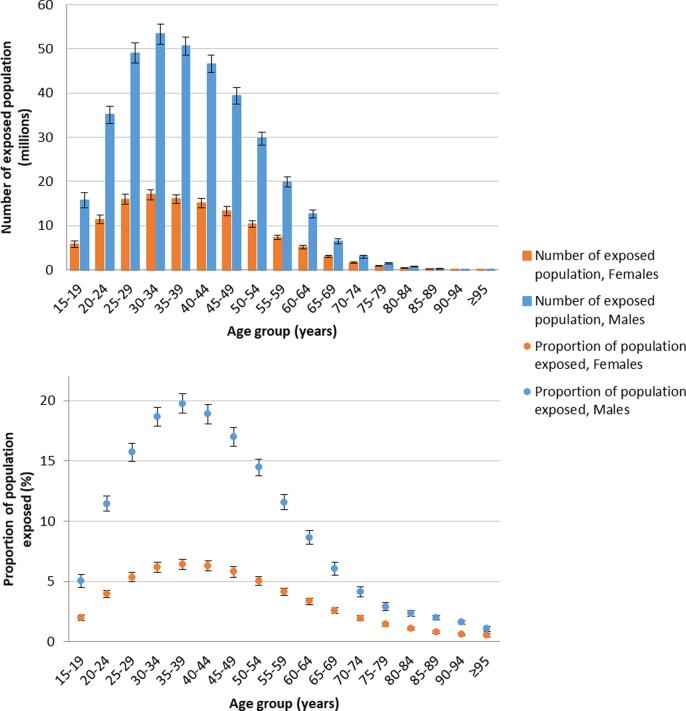
Number of exposed population and proportion of population exposed to long working hours (≥55 hours/week), by sex and age group, 2016, 194 countries.

Globally in 2016, 488 million people (UR 472–503), or 8.9% of the population (UR 8.6–9.1), worked ≥55 hours/week (Table 4). A full set of estimates can be found elsewhere (www.who.int/groups/who-ilo-joint-estimates and http://www.ilo.org/global/topics/safety-and-health-at-work/). Males and adults of early middle-age were more commonly exposed (Fig. 2). Between 2000 and 2016, the global prevalence of this exposure category increased by 9.3% (UR 4.3–14.6) (Table 4).

**Fig. 3:**
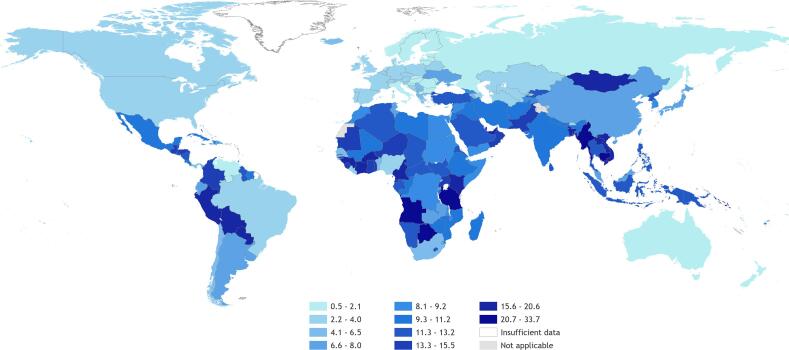
Proportion (%) of population exposed to long working hours (≥55 hours/week), 2016, 194 countries.

In 2016, regional exposure prevalence was largest for South-East Asia (11.7%, UR 10.8–12.5), and lowest in Europe (3.5%, UR 3.5–3.6) (Table S6; Supplementary data 1). Over the 2000–2016 period, the Western Pacific had the largest regional increase; prevalence decreased most in Africa. A map of the proportion of population exposed by country is shown in Fig. 3.

### 3.2 Burden of disease attributable to exposure to long working hours (≥55 hours/week)

**Fig. 4:**
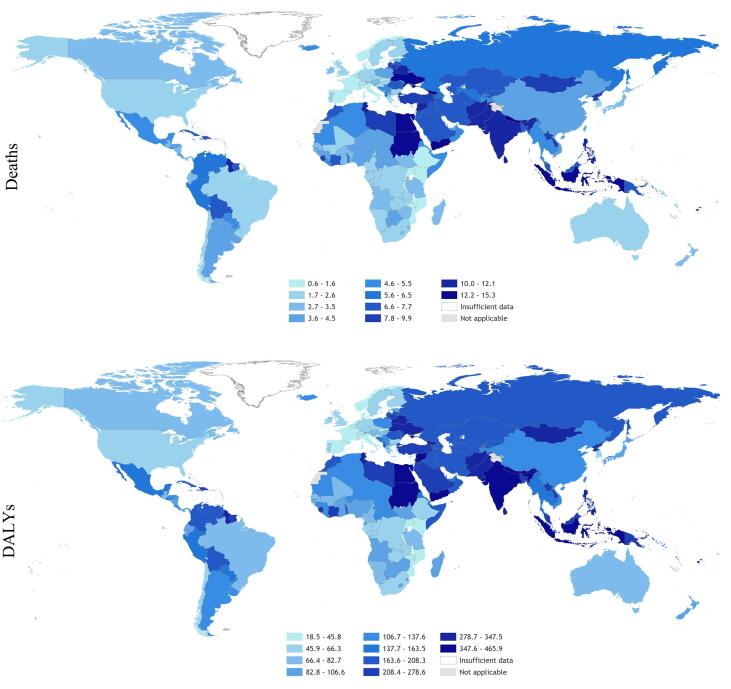
Rate of deaths (per 100,000 of population) and DALYs (per 100,000 of population) from ischemic health disease attributable to exposure to long working hours (≥55 hours/week), 2016, 183 countries. Footnote: No estimates were produced for Andorra, Cook Islands, Dominica, Marshall Islands, Monaco, Nauru, Niue, Palau, Saint Kitts and Nevis, San Marino, and Tuvalu, because the envelopes for the burdens of ischemic heart disease were unavailable for these countries (World Health Organization, 2018b).

**Fig. 5:**
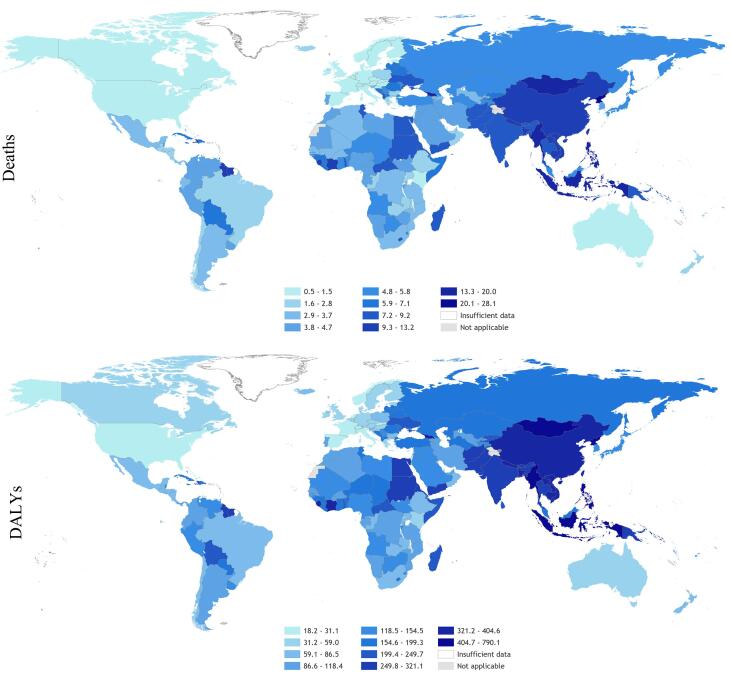
Rate of deaths (per 100,000 of population) and DALYs (per 100,000 of population) from stroke attributable to exposure to long working hours (≥55 hours/week), 2016, 183 countries. Footnote: No estimates were produced for Andorra, Cook Islands, Dominica, Marshall Islands, Monaco, Nauru, Niue, Palau, Saint Kitts and Nevis, San Marino, and Tuvalu, because the envelopes for the burdens of stroke were unavailable for these countries (World Health Organization, 2018b).

In total, an estimated 745,194 deaths (UR 705,786–784,601) and 23.3 million DALYs (UR 22.2–24.4) from ischemic heart disease and stroke combined were attributable to long working hours exposure globally in 2016. This was roughly equal between the two causes, with ischemic heart disease and stroke accounting for 46.5% and 53.5% of estimated deaths, respectively. Fig. 4, Fig. 5 present maps of the rates of deaths and DALYs from ischemic heart disease and stroke that are attributable to exposure to long working hours by country.

#### 3.2.1 Burden of ischemic heart disease attributable to exposure to long working hours (≥ 55 hours/week)

##### 3.2.1.1 Deaths

**Fig. 6:**
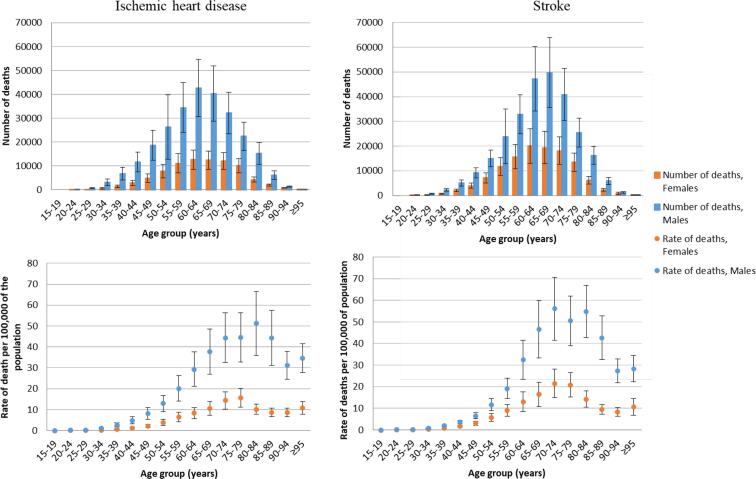
Number of deaths and rate of death (per 100,000 of population) for ischemic heart disease and stroke attributable to exposure to long working hours (≥55 hours/week), by age group, 2016, 183 countries.

Globally in 2016, an estimated total of 9,401,800 ischemic heart disease deaths occurred. Of these, 346,753 (UR 319,658–373,848) were attributable to exposure to long working hours (Table 5). Thus, the PAF is 3.7% (UR 3.4–4.0). Males carried a larger burden, and numbers and rates of deaths increased with age up to 70 years (Fig. 6). Between 2000 and 2016, the numbers of ischemic heart disease deaths attributable to exposure to long working hours increased substantially by 41.5% (UR 27.9–56.5) (Table 5). The PAF was 3.5% (UR 3.3–3.7), 3.6% (UR 3.4–3.9), and 3.7% (UR 3.4–4.0), in 2000, 2010, and 2016, respectively. The upward trend in number of these attributable ischemic heart disease deaths was therefore driven by the increase in the total disease burden envelope, rather than an increase in the exposure.

Of all WHO regions in 2016, South-East Asia had the largest number of ischemic heart disease deaths attributable to exposure to long working hours (159,832 deaths, UR 135,442–184,242; Table S8 in Supplementary data file 1). Africa had the lowest (16,942 deaths, UR 15,878–18,005).

##### 3.2.1.2 DALYs

Globally in 2016, of the 202.8 million DALYs lost from ischemic heart disease in total, 10.7 million DALYs (UR 9.9–11.4) were attributable to exposure to long working hours (Table 5). Between 2000 and 2016, attributable DALYs increased by 41.2% (UR 28.5–54.6). In 2000, 2010, and 2016, the PAF was 4.8 (UR 4.5–5.1), 5.1 (UR 4.7–5.4), and 5.3 (UR 4.9–5.6), respectively (Table 6).

**Table 6:** Population-attributable fraction (PAF) for deaths and DALYs from ischemic heart disease and stroke, attributable to exposure to long working hours (≥55 hours/week), 2000, 2010, and 2016, by sex, 183 countries.

**Outcome**		**Both sexes**						**Males**						**Females**					
		**PAF (UR)**			**Percent change (UR)**			**PAF (UR)**			**Percent change (UR)**			**PAF (UR)**			**Percent change (UR)**		
		2000	2010	2016	2000–2010	2010–2016	2000–2016	2000	2010	2016	2000–2010	2010–2016	2000–2016	2000	2010	2016	2000–2010	2010–2016	2000–2016
**Ischemic heart disease**^a^	Deaths	3.5 (3.3–3.7)	3.6 (3.4–3.9)	3.7 (3.4–4.0)	3.2 (−6.5–13.7)	2.1 (−8.6–13.6)	5.4 (−5.0–16.7)	5.2 (4.8–5.6)	5.3 (4.8–5.8)	5.3 (4.8–5.9)	1.1 (−10.7–14.2)	1.1 (−11.9–15.8)	2.2 (−10.0–15.9)	1.7 (1.6–1.9)	1.8 (1.7–2.0)	1.9 (1.7–2.1)	7.9 (−6.4–24.2)	2.3 (−12.4–19.2)	10.4 (−4.6–27.0)
	DALYs	4.8 (4.5–5.1)	5.1 (4.7–5.4)	5.3 (4.9–5.6)	5.3 (−3.6–15.0)	3.5 (−6.2–14.2)	9.0 (−0.9–19.5)	6.4 (5.9–6.9)	6.7 (6.1–7.2)	6.8 (6.2–7.5)	4.1 (−6.7–16.2)	2.7 (−9.2–16.1)	6.9 (−4.9–19.5)	2.6 (2.4–2.9)	2.9 (2.6.–3.2)	3.0 (2.7–3.3)	9.6 (−3.7–24.9)	3.8 (−10.2–19.8)	13.8 (−0.8–29.8)
**Stroke**^a^	Deaths	6.5 (6.1–7.0)	6.8 (6.3–7.3)	6.9 (6.4– 7.5)	3.8 (−6.4–14.8)	2.2 (−8.0–13.7)	6.1 (−4.9–18.0)	9.5 (8.6–10.4)	9.6 (8.7–10.5)	9.6 (8.6–10.6)	0.9 (−11.5–15.1)	0.5 (−12.7–15.7)	1.4 (−11.8–16.1)	3.9 (3.5–4.3)	4.1 (3.7–4.6)	4.3 (3.8–4.8)	6.0 (−8.7–23.2)	3.0 (−12.3–20.4)	9.2 (−6.9–28.0)
	DALYs	8.6 (8.1–9.1)	9.0 (8.5–9.5)	9.3 (8.7– 9.9)	4.5 (−3.7–13.5)	2.8 (−5.5–11.8)	7.4 (−1.3–17.1)	11.6 (10.7–12.4)	11.8 (10.9–12.7)	12.0 (11.1–13.0)	2.4 (−7.7–13.6)	1.6 (−8.8–13.0)	4.1 (−6.7–15.5)	5.6 (5.1–6.1)	6.0 (5.4.–6.5)	6.2 (5.6–6.8)	6.5 (−6.4–20.5)	3.8 (−9.2–18.9)	10.5 (−3.2–25.8)

Footnotes: No estimates were produced for Andorra, Cook Islands, Dominica, Marshall Islands, Monaco, Nauru, Niue, Palau, Saint Kitts and Nevis, San Marino, and Tuvalu, because the envelopes for the burdens of ischemic heart disease and stroke were unavailable for these countries (World Health Organization, 2018b).

a The effects of working ≥55 hours/week, compared with standard working hours of 35–40 hours/week, were modelled. UR = 95% uncertainty range.

#### 3.2.2 Burden of stroke attributable to exposure to long working hours (≥55 hours/week)

##### 3.2.2.1 Deaths

Globally, stroke caused an estimated 5,747,289 deaths in 2016. Of these, 398,441 (UR 369,826–427,056) were attributable to exposure to long working hours (Table 5). Thus, the PAF was 6.9% (UR 6.4–7.5). Both in absolute and relative terms, males and older age groups (60–74 years) carried a larger burden (Fig. 6). Over 2000–2016, the total number of stroke deaths attributable to exposure to long working hours increased by 19.0% (UR 7.8–31.1) (Table 5). The PAF was 6.5% (UR 6.1–7.0), 6.8% (UR 6.3–7.3), and 6.9% (UR 6.4–7.5), in 2000, 2010, and 2016, respectively (Table 6). The upward trend in number of attributable stroke deaths was therefore also primarily driven by the increase in the total disease burden envelope.

The largest number of deaths regionally was estimated for South-East Asia (158,987 deaths, UR 141,968–176,006; Table S10 in Supplementary file 1). The lowest number was for the Americas (18,285 deaths, UR 17,162–19,409).

##### 3.2.2.2 DALYs

Globally in 2016, of the 135.9 million DALYs lost from stroke, 12.6 million (UR 11.8–13.4) were attributable to exposure to long working hours (Table 5), up by 21.7% from 2000 (UR 11.8–32.4). In 2000, 2010, and 2016, the PAF was 8.6 (UR 8.1–9.1), 9.0 (UR 8.5–9.5), and 9.3% (UR 8.7–9.9), respectively (Table 6).

### 3.3 Sensitivity analyses

Results from sensitivity analyses showed that despite some variation in estimates, number of deaths and DALYs remained appreciable when the assumed lag time was reduced to 8 years and increased to 12 years and when the time window was reduced to 8 years and increased to 12 years (Table S11; Supplementary file 1). Assigning the most common exposure category, rather than the highest one, however, reduced the deaths and DALYs substantially.

## 4 Discussion

This article presented WHO/ILO Joint Estimates of exposure to long working hours and the attributable burdens of ischemic heart disease and stroke. In summary, in 2016, 8.9% of the global population were exposed to working ≥55 hours/week. An estimated 745,194 deaths and 23.3 million DALYs from ischemic heart disease and stroke combined were attributable to this occupational risk factor. Of all deaths from ischemic heart disease and stroke in 2016, 3.7% and 6.9% were attributable to exposure to working long hours; as were 5.3% and 9.3% of all DALYs from ischemic heart disease and stroke. The disease burdens were disproportionately higher in the South-East Asian and Western Pacific regions, men, and people of middle to older working age. Between the years 2000 and 2016, the exposed population increased by 9.3%, and the attributable burdens of deaths from ischemic heart disease and stroke increased by 41.5% and 19.0%, respectively.

### 4.1 Comparison with previous findings and interpretations

The distribution in the population and trends over time in the WHO/ILO Joint Estimates of exposure to long working hours are consistent with recent ILO analyses of population distributions and time trends observed in official survey data on working time (Messenger, 2018). To our knowledge, there are no prior estimates of burden of disease attributable to exposure to long working hours that we could compare these first WHO/ILO Joint Estimates against.

These WHO/ILO Joint Estimates demonstrate that the disease burden attributable to exposure to long working hours is the largest of any occupational risk factor calculated to date, compared with those attributable to other occupational risk factors included in global Comparative Risk Assessments (GBD 2016 Occupational Risk Factor Collaborators, 2019, World Health Organization, 2016).

The population prevalence of exposure to long working hours increased substantially between 2010 and 2016. If this trend continues, it is likely that the population exposed to this occupational risk factor will expand further. Potential reasons for this include expansion of the gig economy (Messenger, 2018), the uncertainty introduced, and new working-time arrangements (e.g., on-call work, telework, and the platform economy). Past experience has shown that working hours increased after previous economic recessions (Bloom, 2009); such increases may also be associated with the COVID-19 pandemic. Estimated increases in exposure have been largest in South-East Asia and the Western Pacific. Furthermore, the total envelopes of the burdens of ischemic heart disease and stroke are also increasing rapidly. As both exposed population and total disease burden expand, the burdens of ischemic heart disease and stroke that can be attributed to exposure to working long hours may therefore also be expected to increase.

### 4.2 Strengths and limitations

WHO and ILO have produced a detailed set of estimates of the burdens of ischemic heart disease and stroke attributable to exposure to long working hours. These estimates were based on systematic reviews and meta-analyses of evidence to date. Multiple data sources provided large samples from all regions to calculate estimates.

However, the study has some limitations, which should be considered when interpreting the findings. First, the systematic reviews and meta-analyses estimated relatively small increases in risk, even where statistical significance is reached. Evidence comes from observational studies, for which residual confounding cannot be ruled out, and we cannot be certain that a causal association exists; however most of the high-quality evidence came from prospective cohort studies which took steps to reduce confounding and will be more representative of population risk. Taking this into consideration, the Working Group of individual experts rated the evidence as of “moderate quality” and as “sufficient evidence for harmfulness” (Descatha et al., 2020, Li et al., 2020a) for the exposure category ≥55 hours/week for both ischemic heart disease and stroke. Other researchers have disagreed with the rating of the Working Group that there is “sufficient evidence for harmfulness” of long working hours with regard to ischemic heart disease (Kivimäki et al., 2020). The Working Group has acknowledged this disagreement and has elaborated why the assigned rating is supported by the evidence (Li et al., 2020b).

Second, it has been argued that, at least with regard to ischemic heart disease, not only length of working hours, but also the quality of the work in which persons spend their hours, may be of importance (Kivimäki et al., 2020). Although Kivimäki et al. (2020) presented data and the Working Group of individual experts conducted analyses stratified by SES that suggested a trend for stronger associations between exposure to long working hours and risk of ischemic heart disease among individuals of lower SES (Li et al., 2020a), these were limited to studies from high-income countries and one region only (Europe). The Working Group deemed the evidence for a possible effect modification of SES in the association between long working hours and ischemic heart disease as inconclusive for a global study (Li et al., 2020a, Li et al., 2020b). Currently, evidence does not support producing such global health estimates disaggregated by SES; however more research is needed in additional and more diverse countries and regions on the roles of SES and work quality for the association of long working hours with health outcomes.

Third, quality of data on both long working hours and burdens of ischemic heart disease and stroke will vary by source. Most data regarding long working hours were obtained from national statistics offices with established, official data collection protocols (e.g., statistical standards), but variation can still be expected. All surveys used self-reported data on working hours. Several studies showed both reliability and validity of self-reported hours, compared with administrative records (Imai et al., 2016, Saunders et al., 2005, Todd et al., 2010); however, this may vary. This could lead to under- or over-estimations of the burden, depending on the direction of the error.

Fourth, several assumptions were made during modelling. While they were based on current knowledge and transparently described in detail (Table 3), these may need review as more evidence becomes available. In addition, as noted above, we provide policy makers with alternative scenarios through sensitivity analyses. While changes to the assumed lag time and time window, in the sensitivity analyses, resulted in some variation from the main results, a relatively large reduction in attributable deaths and DALYs were seen when the most common exposure category was used, rather than the highest. As described in Table 3, the highest exposure category was used as risks from exposures can remain, even when exposure levels are reduced, for diseases with long latency periods. However, this may have resulted in overestimation of health loss attributable to exposure to long working hours. Assignment of the highest exposure category (as in our main analysis) is the standard practice in burden of disease estimation studies; however, by providing additional information for policy making, sensitivity analyses of estimates with the most common exposure category assigned, we show that if this exposure assignment was to be found to be evidence-based, then this would be the estimated disease burden, adding further transparency through adding another scenario.

Fifth, there are additional factors likely to affect both working hours and disease burdens, which were not considered in these analyses (e.g., shift work), but could be considered in future cycles of the WHO/ILO Joint Estimates (Li et al., 2020b). Seasonal variations could be a factor for some occupations (e.g., agricultural workers). For some countries longitudinal survey data were available, for which quarterly surveys took data from an individual over multiple time points. When these data were available, we took an average of the data which should mitigate some of the seasonal differences; however, the majority of the data used in the assessment of long working hours exposure were cross-sectional, so this is a limitation. There are also several potential mediators (Fig. 1), which are challenging to address (Descatha et al., 2020, Li et al., 2020a). Sixth, there may be competing risks; for example, people exposed to long working hours may die of other causes or migrate before reaching the typical ages when cardiovascular disease events occur.

## 5 Conclusions

WHO and ILO estimate that exposure to long working hours (≥55 hours/week) is a prevalent occupational risk factor, attributable for a large number of deaths and DALYs due to ischemic heart disease and stroke. In the global Comparative Risk Assessment, it is currently the occupational risk factor with the largest attributable disease burden. These first WHO/ILO Joint Estimates of the Work-related Burden of Disease and Injury provide the basis for actions to prevent exposure to hazardous long working hours and thereby reduce the attributable burden of ischemic heart disease and stroke, at the global, regional, and national levels, across the health and labour sectors. This includes implementation of the international labour standards on working time, such as setting standards on working time limits (International Labour Organization, 2019a, International Labour Organization, 2019b). Legislation, regulations, policies, programmes, and interventions on working time arrangements must ensure that the setting, monitoring, and enforcement of hours of work and the number of additional hours performed by workers occur within a framework that does not harm human health (Landsbergis, 2018, Messenger, 2018).

## Disclaimers

The authors alone are responsible for the views expressed in this article and they do not necessarily represent the views, decisions or policies of the institutions with which they are affiliated. The views expressed herein can in no way be taken to reflect the official opinion of the European Union.

The designations employed and the presentation of the material in this publication do not imply the expression of any opinion whatsoever on the part of WHO concerning the legal status of any country, territory, city or area or of its authorities, or concerning the delimitation of its frontiers or boundaries. Dotted and dashed lines on maps represent approximate border lines for which there may not yet be full agreement.

Terminology used to refer to countries, territories, and areas as well as representation of countries, territories, and areas, including delimitation of frontiers or boundaries, and any direct or indirect attribution of status in this publication follow exclusively the institutional style and practice of WHO. All reasonable precautions have been taken by WHO to verify the information contained in this publication.

However, the published material is being distributed without warranty of any kind, either expressed or implied. The responsibility for the interpretation and use of the material lies with the reader. In no event shall WHO be liable for damages arising from its use.

## Funding

This study was prepared with financial support to WHO from: the National Institute for Occupational Safety and Health of the Centres for Disease Control and Prevention of the United States of America (Grant 1E11OH0010676-02; Grant 6NE11OH010461-02-01; and Grant 5NE11OH010461-03-00); the German Federal Ministry of Health (BMG Germany) under the BMG-WHO Collaboration Programme 2020–2023 (WHO specified award ref. 70672); and the Spanish Agency for International Cooperation (AECID) (WHO specified award ref. 71208). ILO acknowledges financial support from the Vision Zero Fund (VZF) project on filling data and knowledge gaps on occupational safety and health in global supply chains funded by the European Union, which is implemented within the framework of the ILO Flagship Programme Safety + Health for All. The funders had no role in study design, data collection, and analysis, decision to publish, or preparation of the manuscript

## CRediT authorship contribution statement

**Frank Pega:** Conceptualization, Data curation, Methodology, Formal analysis, Visualization, Writing - original draft, Supervision, Writing - review & editing. **Bálint Náfrádi:** Data curation, Methodology, Formal analysis, Visualization, Writing - review & editing. **Natalie C. Momen:** Visualization, Writing - review & editing. **Yuka Ujita:** Writing - review & editing. **Kai N. Streicher:** Formal analysis, Visualization, Writing - review & editing. **Annette M. Prüss-Üstün:** Methodology, Formal analysis, Supervision, Writing - review & editing. **Technical Advisory Group:** Methodology, Writing - review & editing.

## Declaration of Competing Interest

The authors declare the following financial interests/personal relationships which may be considered as potential competing interests: Professor Alexis Descatha reports personal fees from University of Angers, personal fees from the Angers University Hospital Center, personal fees from Elsevier, and grants from Regional found (Pays de la Loire/Angers Loire Metropole), outside the submitted work.
