# Supplementary material for: Global, regional, and national burdens of ischemic heart disease and stroke attributable to exposure to long working hours for 194 countries, 2000–2016: A systematic analysis from the WHO/ILO Joint Estimates of the Work-related Burden of Disease and Injury
Source: Environ Int. 2021 Sep;154:106595. doi: 10.1016/j.envint.2021.106595 (PMC8204267; doi:10.1016/j.envint.2021.106595)
Supplement: Supplementary data file 1 [file mmc1.docx]

**Global, regional, and national burdens of ischemic heart disease and stroke attributable to exposure to long working hours for 194 countries, 2000-2016: a systematic analysis from the WHO/ILO Joint Estimates of the Work-related Burden of Disease and Injury**

**Supplementary data file 1**

**Fig. S1: Flow chart showing how data sources, input data and models were combined to produce model outputs and burden estimates**

Input data 1:

Cross-sectional survey data

Input data 2: Longitudinal Labour Force Surveys

Proportion of people in exposure category
by sex and age at first year of time window

Proportion of people in exposure category
by sex and age over the time window

Estimates of prevalence of exposure category

by sex and age at first year of time window

Average annual transition probabilities
between categories of exposure

Number of exposed population over time window

Total number of deaths/DALYs by
sex and age at estimation year

Rate of deaths by sex and age
at each year during time window

Number of people by sex and age
at first year of time window

Model 1

Model 2

Model 3

Model 4

Prevalence of exposure category by sex and age at first year of time window

Average annual transition probabilities between categories of exposure

Number of exposed population over time window

Estimated burden of disease

Input data 4:

WHO life tables

Input data 3:
UN population prospect

Input data 6:
WHO Global Health Estimates

Estimates of relative risks

Input data 5:

WHO and ILO systematic reviews

Input
variables

Models

Outputs

Data

sources

**Legend**

**Fig. S2: Details of time window of exposure**

| 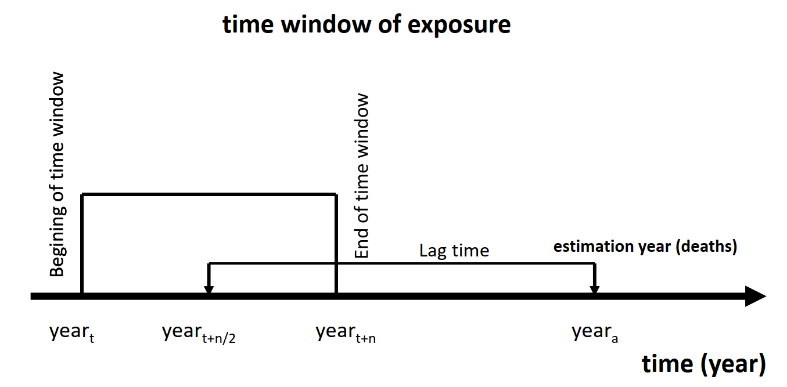 |
| --- |
| Footnotes: We seek to estimate the burden of disease at year_a_ that is attributable to past exposure to a risk factor. For this, we require estimates of the number of persons exposed to a long working hours exposure category over the time window (year_t_ to year_t+n_). The known or assumed lag time is year_t+n/2_ to year_a_. We then seek to estimate the number of persons exposed to the category (as the highest exposure category) *during* the time window. |

**Table S1: Definitions of the risk factor, risk factor levels and the minimum risk exposure level**

|  | **Definition** |
| --- | --- |
| Risk factor | Exposure to long working hours (including those spent in secondary jobs), defined as working ≥41 hours/week. |
| Risk factor levels | Four levels: 35–40, 41–48, 49–54, and ≥55 hours/week. |
| Theoretical minimum risk exposure level | Standard working hours, defined as working hours of 35–40 hours/week. |

**Tables S2 and S3: Detailed description and meta-data on the WHO/ILO Cross-sectional Global Working Hours Database**

The coverage of all of surveys and a list of countries in the WHO/ILO Cross-sectional Global Working Hours Database is presented in Tables S2 and S3. In total, 467 million observations were included from 2324 surveys conducted in 154 countries between 1 January 1976 and 31 December 2018. This database captured 77.4% of the global population with at least one survey.

**Table S2: Coverage of surveys and countries in the WHO/ILO Cross-sectional Global Working Hours Database**

|  | **Region** (defined as per WHO classification) | | | | | | **World** |
| --- | --- | --- | --- | --- | --- | --- | --- |
|  | **Africa** | **Americas** | **South-East Asia** | **Europe** | **Eastern Mediter-ranean** | **Western Pacific** |  |
| Surveys (N) | 135 | 437 | 96 | 1,435 | 66 | 155 | 2,324 |
| Countries with ≥1 survey (N) (% of countries) | 37 (78.7%) | 24 (68.6%) | 10 (90.9%) | 45 (84.9%) | 11 (50.0%) | 27 (92.3%) | 154 (77.4%) |

**Table S3: List of the surveys in each country in the WHO/ILO Cross-sectional Global Working Hours Database**

| **Country** | **Year, survey, survey participants (N)** | **Producer of survey** |
| --- | --- | --- |
| Afghanistan | 2011 GALLUP survey (GALLUP), (1000); 2012 GALLUP, (2000); 2013 GALLUP, (1000); 2008 Afghanistan Living Conditions Survey (LCS), (73343); 2012 LCS, (84023); 2017 LCS, (155680) | Central Statistics Organization; Gallup |
| Albania | 2007 Labour Force Survey (LFS), (18634); 2008 LFS, (18907); 2009 LFS, (18997); 2010 LFS, (18436); 2011 LFS, (13598); 2012 LFS, (39823); 2013 LFS, (40415); 2010 National Child Labour Survey (NCLS), (27865) | Institute Of Statistics |
| Angola | 2009 Core Welfare Indicators Questionnaire Survey (QUIBB), (58739) | National Statistical Office - Angola |
| Argentina | 2010 Annual Urban Household Survey (EAHU), (120656); 2011 EAHU, (111950); 2012 EAHU, (114265); 2013 EAHU, (94047); 2014 EAHU, (98039); 2003 Household Survey (EPH), (93244); 2004 EPH, (187930); 2005 EPH, (188755); 2006 EPH, (222968); 2007 EPH, (188433); 2008 EPH, (247819); 2009 EPH, (240967); 2010 EPH, (237055); 2011 EPH, (231657); 2012 EPH, (223622); 2013 EPH, (222230); 2014 EPH, (241181); 2015 EPH, (120173); 2016 EPH, (177515); 2017 EPH, (235332); 2018 EPH, (230083) | Instituto Nacional De Estadística Y Censos De La República Argentina |
| Armenia | 2007 Households Integrated Living Conditions Survey (HILCS), (30550); 2008 HILCS, (32756); 2009 HILCS, (32362); 2010 HILCS, (29986); 2011 HILCS, (28840); 2012 HILCS, (20134); 2013 HILCS, (18196); 2014 HILCS, (18449); 2015 HILCS, (17798); 2016 HILCS, (17828); 2017 HILCS, (26630); 2008 LFS, (7288); 2014 LFS, (29453); 2015 LFS, (29662); 2016 LFS, (28516); 2017 LFS, (28463); 2015 NCLS, (28273); 2012 School-to-Work Transition Survey (SWTS), (3216); 2014 SWTS, (2710) | National Statistical Service Of Republic Of Armenia |
| Australia | 2011 GALLUP, (1010); 2013 GALLUP, (1002); 2014 GALLUP, (2002) | Gallup |
| Austria | 1995 EU Labour Force Survey (EULFS), (60337); 1996 EULFS, (60325); 1997 EULFS, (59717); 1998 EULFS, (61902); 1999 EULFS, (61618); 2000 EULFS, (59146); 2001 EULFS, (59296); 2002 EULFS, (58717); 2003 EULFS, (56268); 2004 EULFS, (45185); 2005 EULFS, (205058); 2006 EULFS, (201083); 2007 EULFS, (201771); 2008 EULFS, (194070); 2009 EULFS, (185242); 2010 EULFS, (182154); 2011 EULFS, (180847); 2012 EULFS, (180941); 2013 EULFS, (180923); 2014 EULFS, (177254); 2015 EULFS, (173881); 2016 EULFS, (177375); 2017 EULFS, (178997); 2004 LFS, (195881); 2005 LFS, (205058); 2006 LFS, (201083); 2007 LFS, (201771); 2008 LFS, (194070); 2009 LFS, (185242); 2010 LFS, (182154); 2011 LFS, (180847); 2012 LFS, (180941); 2013 LFS, (180923); 2014 LFS, (177254); 2015 LFS, (173881); 2016 LFS, (177375); 2017 LFS, (178997); 2018 LFS, (175914) | Statistik Austria |
| Bangladesh | 2013 Labour Force Survey (CLLFS), (156987); 2011 GALLUP, (1000); 2012 GALLUP, (3000); 2013 GALLUP, (1000); 2014 GALLUP, (1000); 2006 LFS, (188487); 2010 LFS, (199274); 2013 LFS, (156987); 2016 LFS, (249626); 2017 LFS, (493886); 2013 SWTS, (9197) | Bangladesh Bureau Of Statistics; Gallup |
| Belgium | 1983 EULFS, (110468); 1984 EULFS, (43290); 1985 EULFS, (89872); 1986 EULFS, (81455); 1987 EULFS, (74576); 1988 EULFS, (77967); 1989 EULFS, (77321); 1990 EULFS, (77148); 1991 EULFS, (78026); 1992 EULFS, (77690); 1993 EULFS, (81219); 1994 EULFS, (81281); 1995 EULFS, (80385); 1996 EULFS, (81760); 1997 EULFS, (80373); 1998 EULFS, (80066); 1999 EULFS, (27479); 2000 EULFS, (27446); 2001 EULFS, (26391); 2002 EULFS, (27834); 2003 EULFS, (27565); 2004 EULFS, (27739); 2005 EULFS, (109125); 2006 EULFS, (114275); 2007 EULFS, (111978); 2008 EULFS, (105604); 2009 EULFS, (104395); 2010 EULFS, (102473); 2011 EULFS, (95940); 2012 EULFS, (96720); 2013 EULFS, (96519); 2014 EULFS, (102316); 2015 EULFS, (96561); 2016 EULFS, (98568); 2017 EULFS, (47153) | Statbel, The Belgian Statistical Office |
| Belize | 2013 Child Activity Survey (CAS), (20874); 2013 LFS, (30240); 2014 LFS, (19020); 2015 LFS, (19354); 2016 LFS, (19036); 2017 LFS, (17580); 2018 LFS, (8309) | Statistical Institute Of Belize |
| Benin | 2011 Integrated Modular Survey on Household Living Conditions (EMICOV), (87640); 2012 SWTS, (6917); 2014 SWTS, (4305) | National Statistical Office - Benin |
| Bhutan | 2013 GALLUP, (1000); 2014 GALLUP, (1020) | Gallup |
| Bolivia (Plurinational State of) | 2015 Continuous Employment Survey (ECE), (69431); 2016 ECE, (254234); 2017 ECE, (253293); 2018 ECE, (245041); 2005 Household Survey (EH), (16895); 2006 EH, (16511); 2007 EH, (16804); 2008 EH, (15030); 2009 EH, (15665); 2011 EH, (33821); 2012 EH, (31935); 2013 EH, (35693); 2014 EH, (36618); 2015 EH, (37364); 2016 EH, (38549); 2017 EH, (38201); 2008 Child Labour Survey (ETI), (21315) | Instituto Nacional De Estadística |
| Bosnia and Herzegovina | 2006 LFS, (30145); 2007 LFS, (28735); 2008 LFS, (28774); 2009 LFS, (28214); 2010 LFS, (28527); 2011 LFS, (27422); 2012 LFS, (26936); 2013 LFS, (26765); 2014 LFS, (25630); 2015 LFS, (24600); 2016 LFS, (22468); 2017 LFS, (21311); 2018 LFS, (20371) | Agency For Statistics Of Bosnia And Herzegovina |
| Botswana | 2009 Botswana Core Welfare Indicators Survey (BCWIS), (27301) | Central Statistics Office |
| Brazil | 2002 The Monthly Employment Survey (PME), (979728); 2003 PME, (1202638); 2004 PME, (1207015); 2005 PME, (1208189); 2006 PME, (1223796); 2007 PME, (1225931); 2008 PME, (1235231); 2009 PME, (1226186); 2010 PME, (1220965); 2011 PME, (1198368); 2012 PME, (1177820); 2013 PME, (1168514); 2014 PME, (1133207); 2015 PME, (1101087); 2016 PME, (175805); 1995 National Household Sample Survey (PNAD), (334263); 1996 PNAD, (331263); 1997 PNAD, (346269); 1998 PNAD, (344975); 1999 PNAD, (352393); 2001 PNAD, (378837); 2002 PNAD, (385431); 2003 PNAD, (384834); 2004 PNAD, (399354); 2005 PNAD, (408148); 2006 PNAD, (410241); 2007 PNAD, (399964); 2008 PNAD, (391868); 2009 PNAD, (399387); 2011 PNAD, (358919); 2012 PNAD, (362451); 2013 PNAD, (362555); 2014 PNAD, (362627); 2015 PNAD, (356904); 2012 Continuous National Household Sample Survey (PNADC), (2252464); 2013 PNADC, (2278177); 2014 PNADC, (2297503); 2015 PNADC, (2283038); 2016 PNADC, (2280164); 2017 PNADC, (2268651); 2018 PNADC, (2230899); 2012 SWTS, (3288) | Instituto Brasileiro De Geografia E Estatística |
| Brunei Darussalam | 2014 LFS, (17199); 2017 LFS, (14502); 2018 LFS, (13656) | Department Of Economic Planning And Development |
| Bulgaria | 2000 EULFS, (53404); 2001 EULFS, (59991); 2002 EULFS, (57549); 2003 EULFS, (38524); 2004 EULFS, (36540); 2005 EULFS, (140035); 2006 EULFS, (134298); 2007 EULFS, (130964); 2008 EULFS, (34835); 2009 EULFS, (35329); 2010 EULFS, (34791); 2011 EULFS, (33103); 2012 EULFS, (30924); 2013 EULFS, (33240); 2014 EULFS, (33846); 2015 EULFS, (31453); 2016 EULFS, (35309); 2017 EULFS, (32064) | National Statistical Institute |
| Burkina Faso | 2014 Continuous Multisectoral Survey (EMC), (83190) | Burkina Faso National Institute Of Statistics And Demography |
| Burundi | 2014 Survey on Household Living Conditions (ECVM), (22245) | Burundi Institute Of Statistics And Economic Studies |
| Cabo Verde | 2015 Minimum Wage Survey (IMO), (5426) | National Institute Of Statistics |
| Cambodia | 2011 GALLUP, (1000); 2012 GALLUP, (1000); 2013 GALLUP, (1000); 2014 GALLUP, (1000); 2007 Household Socio-Economic Survey (HSES), (17439); 2008 HSES, (16879); 2010 HSES, (16510); 2011 HSES, (16327); 2012 HSES, (17644); 2013 HSES, (17225); 2014 HSES, (53968); 2015 HSES, (17301); 2016 HSES, (16985); 2000 LFS, (27250); 2001 LFS, (26898); 2012 LFS, (48290); 2012 SWTS, (3552); 2014 SWTS, (3396) | National Institute Of Statistics Of Cambodia |
| Cameroon | 2007 Cameroon Household Survey (ECAM), (51837); 2014 ECAM, (38961) | National Statistical Office - Cameroon |
| Canada | 1976 LFS, (859477); 1977 LFS, (1349961); 1978 LFS, (1340685); 1979 LFS, (1329755); 1980 LFS, (1316809); 1981 LFS, (1323921); 1982 LFS, (1316161); 1983 LFS, (1307411); 1984 LFS, (1289328); 1985 LFS, (1216062); 1986 LFS, (1129278); 1987 LFS, (1065577); 1988 LFS, (1046247); 1989 LFS, (1031195); 1990 LFS, (1286257); 1991 LFS, (1337373); 1992 LFS, (1340400); 1993 LFS, (1259378); 1994 LFS, (1216528); 1995 LFS, (1162302); 1996 LFS, (1100946); 1997 LFS, (1114199); 1998 LFS, (1105971); 1999 LFS, (1091701); 2000 LFS, (1076281); 2001 LFS, (1101561); 2002 LFS, (1075887); 2003 LFS, (1067021); 2004 LFS, (1043675); 2005 LFS, (1066505); 2006 LFS, (1080553); 2007 LFS, (1087091); 2008 LFS, (1086951); 2009 LFS, (1114325); 2010 LFS, (1109273); 2011 LFS, (1109343); 2012 LFS, (1105719); 2013 LFS, (1094905); 2014 LFS, (1091613); 2015 LFS, (1054839); 2016 LFS, (1062807); 2017 LFS, (1065928); 2018 LFS, (1047767) | Statistics Canada |
| Chile | 1990 Chile National Socioeconomic Characterization Survey (CASEN), (105189); 1992 CASEN, (143459); 1994 CASEN, (178057); 1996 CASEN, (134262); 1998 CASEN, (188360); 2000 CASEN, (252748); 2006 CASEN, (268873); 2009 CASEN, (246924); 2011 CASEN, (294791); 2013 CASEN, (218491); 2015 CASEN, (266968); 2017 CASEN, (216439); 2012 Survey of Children and Adolescents (EANNA), (9978); 2010 National Employment Survey (ENE), (449680); 2011 ENE, (453301); 2012 ENE, (437349); 2013 ENE, (430550); 2014 ENE, (429516); 2015 ENE, (420742); 2016 ENE, (415813); 2017 ENE, (411257); 2018 ENE, (411476) | National Statistics Institute (Chile) |
| China (People’s Republic of) | 2008 Chinese Household Income Project (CHIP), (46474); 2013 CHIP, (61162); 2011 GALLUP, (4220); 2012 GALLUP, (9413); 2013 GALLUP, (4244) | National Bureau of Statistics of China; Gallup |
| Colombia | 2012 National Child Labor Survey (ENTI), (199696); 2007 Gran Encuesta Integrada de Hogares (GEIH), (838421); 2008 GEIH, (823814); 2009 GEIH, (816242); 2010 GEIH, (822087); 2011 GEIH, (827526); 2012 GEIH, (812711); 2013 GEIH, (797877); 2014 GEIH, (788101); 2015 GEIH, (787044); 2016 GEIH, (778238); 2017 GEIH, (767867); 2018 GEIH, (762753); 2013 SWTS, (6416) | National Administrative Department Of Statistics |
| Comoros | 2004 Integrated Household Survey (EIM), (17957); 2014 Survey on Employment, the Informal Sector, and Household Living Conditions (ENESI), (16865) | International Labour Office |
| Congo | 2005 QUIBB, (26599); 2015 SWTS, (3276) | National Statistical Office - Congo |
| Cook Islands | 2016 Household income and expediture survey (HIES), (2473) | Cook Islands Statistics Office |
| Costa Rica | 2010 ECE, (52885); 2011 ECE, (105892); 2012 ECE, (101571); 2013 ECE, (100796); 2014 ECE, (105891); 2015 ECE, (105571); 2016 ECE, (104536); 2017 ECE, (104950); 2018 ECE, (103669); 2005 Household Survey (EHPM), (43682); 2008 EHPM, (46101); 2009 EHPM, (48071); 2010 National Household Survey (ENAHO), (41184); 2012 ENAHO, (39390); 2013 ENAHO, (38779); 2014 ENAHO, (38399); 2015 ENAHO, (37291); 2016 ENAHO, (37006); 2017 ENAHO, (34843); 2018 ENAHO, (35096) | National Institute Of Statistics And Census Of Costa Rica |
| Côte d’Ivoire | 2012 Household Employment Survey (ENSE), (24235); 2013 ENSE, (45359); 2016 ENSE, (44003); 2017 Integrated regional survey on employment and the informal sector in UEMOA member states (ERI-ESIS) (ERIESI), (34594) | National Statistical Office - Cote D'Ivoire |
| Croatia | 2002 EULFS, (22336); 2003 EULFS, (20300); 2004 EULFS, (19185); 2005 EULFS, (36841); 2006 EULFS, (35517); 2007 EULFS, (45237); 2008 EULFS, (43847); 2009 EULFS, (42255); 2010 EULFS, (40038); 2011 EULFS, (38205); 2012 EULFS, (37276); 2013 EULFS, (35784); 2014 EULFS, (37406); 2015 EULFS, (38206); 2016 EULFS, (32537); 2017 EULFS, (32475) | Croatian Bureau Of Statistics |
| Cyprus | 2000 EULFS, (10301); 2001 EULFS, (10596); 2002 EULFS, (10667); 2003 EULFS, (10779); 2004 EULFS, (10615); 2005 EULFS, (39514); 2006 EULFS, (38227); 2007 EULFS, (38462); 2008 EULFS, (40380); 2009 EULFS, (41422); 2010 EULFS, (43662); 2011 EULFS, (43522); 2012 EULFS, (43306); 2013 EULFS, (42977); 2014 EULFS, (40772); 2015 EULFS, (40265); 2016 EULFS, (40853); 2017 EULFS, (40926) | Statistical Service |
| Czechia | 1997 EULFS, (72028); 1998 EULFS, (70721); 1999 EULFS, (68824); 2000 EULFS, (65464); 2001 EULFS, (63964); 2002 EULFS, (62091); 2003 EULFS, (60973); 2004 EULFS, (62807); 2005 EULFS, (249737); 2006 EULFS, (253254); 2007 EULFS, (250602); 2008 EULFS, (241934); 2009 EULFS, (235119); 2010 EULFS, (234219); 2011 EULFS, (45361); 2012 EULFS, (44531); 2013 EULFS, (42868); 2014 EULFS, (42010); 2015 EULFS, (42214); 2016 EULFS, (41455); 2017 EULFS, (40993); 2000 LFS, (259450); 2001 LFS, (254462); 2002 LFS, (288629); 2003 LFS, (285598); 2004 LFS, (288298); 2005 LFS, (286804); 2006 LFS, (288607); 2007 LFS, (286914); 2008 LFS, (279332); 2009 LFS, (273363); 2010 LFS, (272262); 2011 LFS, (232040); 2012 LFS, (44531); 2013 LFS, (42868); 2014 LFS, (42010); 2015 LFS, (254903); 2016 LFS, (41455); 2017 LFS, (212506); 2018 LFS, (248840) | Czech Statistical Office |
| Democratic Republic of the Congo | 2005 ENESI, (72673); 2012 ENESI, (111679) | National Institute Of Statistics |
| Denmark | 1983 EULFS, (203663); 1984 EULFS, (30051); 1985 EULFS, (30851); 1986 EULFS, (28876); 1987 EULFS, (28972); 1988 EULFS, (28817); 1989 EULFS, (28897); 1990 EULFS, (28885); 1991 EULFS, (29435); 1992 EULFS, (27028); 1993 EULFS, (29379); 1994 EULFS, (18942); 1995 EULFS, (18449); 1996 EULFS, (18354); 1997 EULFS, (18265); 1998 EULFS, (18233); 1999 EULFS, (17895); 2000 EULFS, (17993); 2001 EULFS, (15986); 2002 EULFS, (16081); 2003 EULFS, (15471); 2004 EULFS, (15445); 2005 EULFS, (59792); 2006 EULFS, (58899); 2007 EULFS, (118001); 2008 EULFS, (115508); 2009 EULFS, (118732); 2010 EULFS, (118351); 2011 EULFS, (118699); 2012 EULFS, (120428); 2013 EULFS, (119431); 2014 EULFS, (119974); 2015 EULFS, (118696); 2016 EULFS, (106172); 2017 EULFS, (109019) | Statistics Denmark |
| Djibouti | 2017 Djibouti Household Survey (EDAM), (21213) | National Statistical Office |
| Dominican Republic | 2015 National Continuous Labor Force Survey (ENCFT), (88818); 2016 ENCFT, (86628); 2017 ENCFT, (82843); 2018 ENCFT, (20627); 2000 National Workforce Survey (ENFT), (44202); 2001 ENFT, (44377); 2002 ENFT, (44452); 2003 ENFT, (51806); 2004 ENFT, (57974); 2005 ENFT, (59885); 2006 ENFT, (58334); 2007 ENFT, (56514); 2008 ENFT, (61432); 2009 ENFT, (61139); 2010 ENFT, (60394); 2011 ENFT, (58912); 2012 ENFT, (57841); 2013 ENFT, (57746); 2014 ENFT, (55407); 2015 ENFT, (53458); 2016 ENFT, (52863); 2015 SWTS, (3554) | Oficina Nacional De Estadística |
| Ecuador | 2000 Employment, Unemployment and Under-Employment Survey (ENEMDU), (62469); 2001 ENEMDU, (60749); 2003 ENEMDU, (82317); 2004 ENEMDU, (202492); 2005 ENEMDU, (77050); 2006 ENEMDU, (77964); 2007 ENEMDU, (76922); 2008 ENEMDU, (116611); 2009 ENEMDU, (78878); 2010 ENEMDU, (162006); 2011 ENEMDU, (150157); 2012 ENEMDU, (144869); 2013 ENEMDU, (158907); 2014 ENEMDU, (349826); 2015 ENEMDU, (346519); 2016 ENEMDU, (289388); 2017 ENEMDU, (285742); 2018 ENEMDU, (238392); 2012 ENTI, (146814) | Instituto Nacional De Estadística Y Censos |
| Egypt | 2008 LFS, (330851); 2009 LFS, (333847); 2010 LFS, (349495); 2011 LFS, (372384); 2012 LFS, (369775); 2013 LFS, (350850); 2015 LFS, (357401); 2016 LFS, (347604); 2017 LFS, (335399); 2012 SWTS, (5198); 2014 SWTS, (5758) | Central Agency For Public Mobilization And Statistics |
| El Salvador | 2010 EHPM, (85159); 2011 EHPM, (85291); 2012 EHPM, (85636); 2013 EHPM, (81865); 2014 EHPM, (80164); 2015 EHPM, (88184); 2016 EHPM, (76264); 2017 EHPM, (75133); 2018 EHPM, (75045); 2015 El Salvador Multipurpose Household Survey (EHPMTI), (88184); 2012 SWTS, (3451); 2014 SWTS, (3604) | Department Of Statistics And Censuses, El Salvador |
| Estonia | 1997 EULFS, (5051); 1998 EULFS, (16861); 1999 EULFS, (16361); 2000 EULFS, (4657); 2001 EULFS, (5179); 2002 EULFS, (4948); 2003 EULFS, (4735); 2004 EULFS, (4533); 2005 EULFS, (17904); 2006 EULFS, (20189); 2007 EULFS, (23342); 2008 EULFS, (22242); 2009 EULFS, (19856); 2010 EULFS, (20065); 2011 EULFS, (21639); 2012 EULFS, (24464); 2013 EULFS, (24315); 2014 EULFS, (24320); 2015 EULFS, (23763); 2016 EULFS, (24232); 2017 EULFS, (27672) | Statistics Estonia |
| Eswatini | 2016 LFS, (13623) | Central Statistical Office |
| Ethiopia | 2005 National Labour Force Survey (NLFS), (230680); 2013 NLFS, (240660) | Central Statistical Agency Of Ethiopia |
| Fiji | 2005 Employment and Unemployment Survey (EUS), (13971); 2011 EUS, (18249); 2016 EUS, (23258) | Fiji Bureau of Statistics |
| Finland | 1995 EULFS, (18675); 1996 EULFS, (17751); 1997 EULFS, (17213); 1998 EULFS, (17063); 1999 EULFS, (35071); 2000 EULFS, (42146); 2001 EULFS, (42712); 2002 EULFS, (42743); 2003 EULFS, (34347); 2004 EULFS, (33979); 2005 EULFS, (33507); 2006 EULFS, (32389); 2007 EULFS, (37364); 2008 EULFS, (37545); 2009 EULFS, (36690); 2010 EULFS, (35796); 2011 EULFS, (34640); 2012 EULFS, (34380); 2013 EULFS, (33466); 2014 EULFS, (33438); 2015 EULFS, (32978); 2016 EULFS, (32420); 2017 EULFS, (31531) | Statistics Finland |
| France | 1983 EULFS, (171130); 1984 EULFS, (170789); 1985 EULFS, (169651); 1986 EULFS, (169549); 1987 EULFS, (170471); 1988 EULFS, (171439); 1989 EULFS, (171365); 1990 EULFS, (168882); 1991 EULFS, (170407); 1992 EULFS, (174797); 1993 EULFS, (181762); 1994 EULFS, (187326); 1995 EULFS, (186482); 1996 EULFS, (185590); 1997 EULFS, (183417); 1998 EULFS, (183072); 1999 EULFS, (182155); 2000 EULFS, (182066); 2001 EULFS, (178143); 2002 EULFS, (175939); 2003 EULFS, (86892); 2004 EULFS, (87773); 2005 EULFS, (342098); 2006 EULFS, (111575); 2007 EULFS, (113696); 2008 EULFS, (115201); 2009 EULFS, (140010); 2010 EULFS, (158773); 2011 EULFS, (168876); 2012 EULFS, (168318); 2013 EULFS, (76405); 2014 EULFS, (85171); 2015 EULFS, (85848); 2016 EULFS, (87532); 2017 EULFS, (83040) | Insee |
| Gambia | 2012 LFS, (32275); 2018 LFS, (57799) | Gambia Bureau Of Statistics (Gbos) |
| Georgia | 2017 LFS, (54824); 2018 LFS, (78150) | National Statistics Office of Georgia |
| Ghana | 2015 LFS, (9604); 2006 Living Standards Survey (LSS), (37128); 2013 LSS, (72372); 2017 LSS, (59864) | Ghana Statistical Service |
| Greece | 1983 EULFS, (126774); 1984 EULFS, (142141); 1985 EULFS, (141713); 1986 EULFS, (141998); 1987 EULFS, (142734); 1988 EULFS, (143139); 1989 EULFS, (140794); 1990 EULFS, (139764); 1991 EULFS, (138129); 1992 EULFS, (135941); 1993 EULFS, (170386); 1994 EULFS, (167225); 1995 EULFS, (165221); 1996 EULFS, (165305); 1997 EULFS, (163893); 1998 EULFS, (84007); 1999 EULFS, (82921); 2000 EULFS, (81264); 2001 EULFS, (80282); 2002 EULFS, (77451); 2003 EULFS, (73017); 2004 EULFS, (80992); 2005 EULFS, (314576); 2006 EULFS, (303621); 2007 EULFS, (296383); 2008 EULFS, (293618); 2009 EULFS, (297460); 2010 EULFS, (302136); 2011 EULFS, (271770); 2012 EULFS, (242672); 2013 EULFS, (245180); 2014 EULFS, (237701); 2015 EULFS, (232211); 2016 EULFS, (249731); 2017 EULFS, (247680); 1998 LFS, (335722); 1999 LFS, (331600); 2000 LFS, (325043); 2001 LFS, (321441); 2002 LFS, (308669); 2003 LFS, (292863); 2004 LFS, (322499); 2005 LFS, (314576); 2006 LFS, (303621); 2007 LFS, (296383); 2008 LFS, (293618); 2009 LFS, (297460); 2010 LFS, (302136); 2011 LFS, (271770); 2012 LFS, (242672); 2013 LFS, (245180); 2014 LFS, (237701); 2015 LFS, (232211); 2016 LFS, (249731); 2017 LFS, (247680); 2018 LFS, (61505) | Hellenic Statistical Authority |
| Guatemala | 2006 Living Standards Survey (ENCOVI), (68739); 2011 ENCOVI, (66523); 2014 ENCOVI, (54819); 2002 National Employment and Income Survey (ENEI), (31361); 2003 ENEI, (10607); 2004 ENEI, (64639); 2010 ENEI, (17919); 2011 ENEI, (17861); 2012 ENEI, (17686); 2013 ENEI, (36169); 2014 ENEI, (35335); 2015 ENEI, (47838); 2016 ENEI, (70999); 2017 ENEI, (69050); 2018 ENEI, (44829) | Ine Guatemala |
| Guinea | 2010 National Survey on Child Labour and Trafficking (ENTE), (26553) | National Institute of Statistics and Census |
| Guyana | 2017 LFS, (15112); | Bureau Of Statistics - Guyana |
| Haiti | 2012 Living Conditions of Households After Earthquake (ECVMAS), (23775) | Haitian Institute of Statistics and Informatics |
| Honduras | 2005 Permanent Household Survey (EPHPM), (34994); 2006 EPHPM, (99645); 2007 EPHPM, (99700); 2008 EPHPM, (99771); 2009 EPHPM, (98028); 2010 EPHPM, (32419); 2011 EPHPM, (32430); 2012 EPHPM, (32830); 2013 EPHPM, (32686); 2014 EPHPM, (24023); 2015 EPHPM, (26539); 2016 EPHPM, (27297); 2017 EPHPM, (23597); 2018 EPHPM, (25943) | Ine Honduras |
| Hungary | 1996 EULFS, (64611); 1997 EULFS, (63443); 1998 EULFS, (84036); 1999 EULFS, (87541); 2000 EULFS, (85080); 2001 EULFS, (84671); 2002 EULFS, (82904); 2003 EULFS, (88212); 2004 EULFS, (82310); 2005 EULFS, (312190); 2006 EULFS, (311397); 2007 EULFS, (305048); 2008 EULFS, (290431); 2009 EULFS, (289049); 2010 EULFS, (282935); 2011 EULFS, (282740); 2012 EULFS, (274633); 2013 EULFS, (259318); 2014 EULFS, (255230); 2015 EULFS, (245064); 2016 EULFS, (234165); 2017 EULFS, (226927) | Hungarian Central Statistical Office |
| Iceland | 1995 EULFS, (3933); 1996 EULFS, (3845); 1997 EULFS, (3759); 1998 EULFS, (3755); 1999 EULFS, (3656); 2000 EULFS, (3697); 2001 EULFS, (3685); 2002 EULFS, (3646); 2003 EULFS, (3186); 2004 EULFS, (3110); 2005 EULFS, (12424); 2006 EULFS, (12550); 2007 EULFS, (12435); 2008 EULFS, (12356); 2009 EULFS, (12370); 2010 EULFS, (12491); 2011 EULFS, (12730); 2012 EULFS, (12681); 2013 EULFS, (12399); 2014 EULFS, (12153); 2015 EULFS, (11910); 2016 EULFS, (11220); 2017 EULFS, (10488) | Statistics Iceland |
| India | 2011 GALLUP, (3518); 2012 GALLUP, (10080); 2013 GALLUP, (5540) | Gallup |
| Indonesia | 2009 National Child Labour Survey (CLS), (47661); 2011 GALLUP, (1000); 2012 GALLUP, (3000); 2013 GALLUP, (1000); 2014 GALLUP, (1000); 1990 LFS, (291095); 1991 LFS, (282334); 1992 LFS, (282542); 1993 LFS, (279784); 1994 LFS, (245206); 1996 LFS, (247199); 1997 LFS, (219439); 1998 LFS, (163517); 1999 LFS, (155572); 2000 LFS, (98952); 2001 LFS, (119935); 2002 LFS, (275353); 2003 LFS, (232466); 2004 LFS, (237290); 2005 LFS, (202633); 2006 LFS, (193696); 2007 LFS, (910277); 2008 LFS, (1151143); 2009 LFS, (1145371); 2010 LFS, (1166189); 2011 LFS, (658411); 2012 LFS, (633531); 2013 LFS, (617677); 2014 LFS, (611436); 2015 LFS, (659946); 2016 LFS, (262491); 2017 LFS, (672010); 2018 LFS, (637998) | Statistics Indonesia; Gallup |
| Iraq | 2007 HSES, (127189); 2012 HSES, (176041) | Central Statistical Organization |
| Ireland | 1983 EULFS, (146954); 1984 EULFS, (153972); 1985 EULFS, (155855); 1986 EULFS, (153692); 1987 EULFS, (156448); 1988 EULFS, (154580); 1989 EULFS, (154882); 1990 EULFS, (151027); 1991 EULFS, (150228); 1992 EULFS, (153913); 1993 EULFS, (151820); 1994 EULFS, (149566); 1995 EULFS, (148090); 1996 EULFS, (144674); 1997 EULFS, (147933); 1998 EULFS, (111342); 1999 EULFS, (109768); 2000 EULFS, (106306); 2001 EULFS, (105405); 2002 EULFS, (105569); 2003 EULFS, (101500); 2004 EULFS, (86545); 2005 EULFS, (91174); 2006 EULFS, (85734); 2007 EULFS, (319039); 2008 EULFS, (278293); 2009 EULFS, (273122); 2010 EULFS, (246328); 2011 EULFS, (228205); 2012 EULFS, (234162); 2013 EULFS, (209237); 2014 EULFS, (208574); 2015 EULFS, (192293); 2016 EULFS, (170246); 2017 EULFS, (153381) | Central Statistics Office Ireland |
| Israel | 2012 LFS, (255453); 2013 LFS, (257010); 2014 LFS, (244278); 2015 LFS, (245270); 2016 LFS, (247855) | Israel Central Bureau of Statistics |
| Italy | 1983 EULFS, (343716); 1984 EULFS, (345484); 1985 EULFS, (331073); 1986 EULFS, (329044); 1987 EULFS, (351592); 1988 EULFS, (349734); 1989 EULFS, (384472); 1990 EULFS, (383682); 1991 EULFS, (192597); 1992 EULFS, (201007); 1993 EULFS, (200550); 1994 EULFS, (198935); 1995 EULFS, (203434); 1996 EULFS, (202432); 1997 EULFS, (201541); 1998 EULFS, (201835); 1999 EULFS, (200625); 2000 EULFS, (199367); 2001 EULFS, (196236); 2002 EULFS, (194041); 2003 EULFS, (192359); 2004 EULFS, (172264); 2005 EULFS, (704372); 2006 EULFS, (684303); 2007 EULFS, (677746); 2008 EULFS, (671939); 2009 EULFS, (659561); 2010 EULFS, (662986); 2011 EULFS, (657569); 2012 EULFS, (606972); 2013 EULFS, (611255); 2014 EULFS, (604580); 2015 EULFS, (597872); 2016 EULFS, (584571); 2017 EULFS, (581449); 2014 LFS, (382989); 2015 LFS, (400747); 2016 LFS, (379592); 2017 LFS, (381796); 2018 LFS, (374790) | Istat |
| Jamaica | 2014 LFS, (14319); 2016 LFS, (17900); 2013 SWTS, (2584); 2015 SWTS, (3666) | Statistical Institute Of Jamaica |
| Japan | 2011 GALLUP, (1000); 2012 GALLUP, (2000); 2013 GALLUP, (1001); 2014 GALLUP, (2006); 2000 LFS, (1600000); 2001 LFS, (1600000); 2002 LFS, (1600000); 2003 LFS, (1600000); 2004 LFS, (1600000); 2005 LFS, (1600000); 2006 LFS, (1600000); 2007 LFS, (1600000); 2008 LFS, (1600000); 2009 LFS, (1600000); 2010 LFS, (1600000); 2012 LFS, (1600000); 2013 LFS, (1600000); 2014 LFS, (1600000); 2015 LFS, (1600000); 2016 LFS, (1600000); 2017 LFS, (1600000); 2018 LFS, (1600000) | Statistics Bureau; Gallup |
| Jordan | 2012 SWTS, (5405); 2015 SWTS, (3749) | Department Of Statistics Of Jordan |
| Kenya | 2006 Household Budget Survey (HBS), (57102); 2016 HBS, (92846); 1999 LFS, (52016) | Kenya National Bureau Of Statistics |
| Kyrgyzstan | 2014 CLS, (24013); 2013 SWTS, (3930) | National Statistical Committee Of The Kyrgyz Republic |
| Lao People's Democratic Republic | 2011 GALLUP, (1000); 2012 GALLUP, (1000); 2010 LFS, (58654); 2017 LFS, (52167) | Lao Statistics Bureau; Gallup |
| Latvia | 1998 EULFS, (18756); 1999 EULFS, (18701); 2000 EULFS, (19193); 2001 EULFS, (18834); 2002 EULFS, (5943); 2003 EULFS, (6022); 2004 EULFS, (6020); 2005 EULFS, (21919); 2006 EULFS, (18640); 2007 EULFS, (37822); 2008 EULFS, (38597); 2009 EULFS, (40424); 2010 EULFS, (37433); 2011 EULFS, (35856); 2012 EULFS, (34887); 2013 EULFS, (39011); 2014 EULFS, (42685); 2015 EULFS, (41362); 2016 EULFS, (41070); 2017 EULFS, (10107) | Central Statistical Bureau of Latvia |
| Lebanon | 2014 SWTS, (2616) | Central Administration Of Statistics |
| Liberia | 2015 HIES, (18089); 2016 HIES, (36308); 2010 LFS, (31809); 2012 SWTS, (1876); 2014 SWTS, (2416) | Liberia Institute Of Statistics And Geo-Information Services |
| Lithuania | 1998 EULFS, (7543); 1999 EULFS, (7558); 2000 EULFS, (7583); 2001 EULFS, (7788); 2002 EULFS, (12993); 2003 EULFS, (12219); 2004 EULFS, (12149); 2005 EULFS, (47947); 2006 EULFS, (44958); 2007 EULFS, (63022); 2008 EULFS, (61774); 2009 EULFS, (66803); 2010 EULFS, (66867); 2011 EULFS, (65166); 2012 EULFS, (62838); 2013 EULFS, (61126); 2014 EULFS, (59789); 2015 EULFS, (56511); 2016 EULFS, (56046); 2017 EULFS, (59156) | Statistics Lithuania |
| Luxembourg | 1983 EULFS, (27727); 1984 EULFS, (27661); 1985 EULFS, (26005); 1986 EULFS, (24660); 1987 EULFS, (24586); 1988 EULFS, (25031); 1989 EULFS, (24629); 1990 EULFS, (24629); 1991 EULFS, (24448); 1992 EULFS, (15202); 1993 EULFS, (14149); 1994 EULFS, (13949); 1995 EULFS, (18731); 1996 EULFS, (18382); 1997 EULFS, (17769); 1998 EULFS, (17326); 1999 EULFS, (16095); 2000 EULFS, (15257); 2001 EULFS, (14814); 2002 EULFS, (13429); 2003 EULFS, (16394); 2004 EULFS, (21189); 2005 EULFS, (90024); 2006 EULFS, (85080); 2007 EULFS, (21178); 2008 EULFS, (14288); 2009 EULFS, (19229); 2010 EULFS, (18781); 2011 EULFS, (19942); 2012 EULFS, (23219); 2013 EULFS, (16139); 2014 EULFS, (13831); 2015 EULFS, (10706); 2016 EULFS, (7126); 2017 EULFS, (8660) | Statec |
| Madagascar | 2012 ENESI, (49670); 2015 ENESI, (15641); 2013 SWTS, (3295); 2015 SWTS, (5044) | National Institute Of Statistics Of Madagascar (Instat) |
| Malawi | 2013 LFS, (29978); 2012 SWTS, (3102); 2014 SWTS, (3097) | Malawi National Statistical Office |
| Malaysia | 2011 GALLUP, (1000); 2012 GALLUP, (1000); 2013 GALLUP, (1000); 2014 GALLUP, (2008) | Gallup |
| Maldives | 2016 HIES, (17448) | National Bureau Of Statistics Of Maldives |
| Mali | 2014 Modulare and Permanent Household Survey (EMOP), (51799); 2015 EMOP, (48462); 2016 EMOP, (46158) | National Institute Of Statistics |
| Malta | 2009 EULFS, (26399); 2010 EULFS, (26183); 2011 EULFS, (23969); 2012 EULFS, (24656); 2013 EULFS, (25597); 2014 EULFS, (25589); 2015 EULFS, (25683); 2016 EULFS, (25513); 2017 EULFS, (24479) | National Statistics Office |
| Marshall Islands | 2018 HIES, (3594) | Rmi Statistics |
| Mauritania | 2012 ENESI, (33547); 2017 ENESI, (47085); | ONS Mauritania |
| Mauritius | 2001 Continuous Multi-Purpose Household Survey (CMPHS), (19738); 2002 CMPHS, (19909); 2003 CMPHS, (20356); 2004 CMPHS, (24281); 2005 CMPHS, (30799); 2006 CMPHS, (30934); 2007 CMPHS, (31363); 2008 CMPHS, (31054); 2009 CMPHS, (30651); 2010 CMPHS, (31016); 2011 CMPHS, (31601); 2012 CMPHS, (31801); 2013 CMPHS, (31189); 2014 CMPHS, (39010); 2015 CMPHS, (38914); 2016 CMPHS, (37946); 2017 CMPHS, (37533); 2018 CMPHS, (37424) | Statistics Mauritius |
| Mexico | 1995 ENE, (151739); 1996 ENE, (499737); 1997 ENE, (161135); 1998 ENE, (511094); 1999 ENE, (226488); 2000 ENE, (1818895); 2002 ENE, (2372167); 2003 ENE, (2098044); 2004 ENE, (1604731); 2005 National Survey of Occupation and Employment (ENOE), (1649291); 2006 ENOE, (1663902); 2007 ENOE, (1643644); 2008 ENOE, (1619352); 2009 ENOE, (1590077); 2010 ENOE, (1593114); 2011 ENOE, (1574013); 2012 ENOE, (1563171); 2013 ENOE, (1552325); 2014 ENOE, (1590748); 2015 ENOE, (1579514); 2016 ENOE, (1548185); 2017 ENOE, (1543995); 2018 ENOE, (1535217) | Inegi |
| Micronesia (Federated States of) | 2014 HIES, (10010) | Fsm Division Of National Statistic |
| Mongolia | 2011 GALLUP, (1000); 2012 GALLUP, (1000); 2013 GALLUP, (1000); 2014 GALLUP, (1000); 2008 LFS, (25300); 2009 LFS, (24940); 2010 LFS, (47493); 2011 LFS, (45358); 2012 LFS, (45445); 2013 LFS, (44678); 2014 LFS, (43664); 2015 LFS, (30607); 2016 LFS, (43680); 2017 LFS, (44414); 2018 LFS, (44260) | National Statistical Office Of Mongolia; Gallup |
| Montenegro | 2011 LFS, (24616); 2012 LFS, (24584); 2013 LFS, (23325); 2014 LFS, (23378); 2015 LFS, (23425); 2016 LFS, (22816); 2017 LFS, (24532); 2018 LFS, (23616); 2015 SWTS, (2998) | Statistical Office Montenegro |
| Mozambique | 2015 Household Budget Survey (IOF), (48930) | National Statistical Office - Mozambique |
| Myanmar | 2012 GALLUP, (1020); 2013 GALLUP, (1020); 2015 LFS, (101278); 2017 LFS, (120598); 2018 LFS, (117070) | Myanmar Statistics; Gallup |
| Namibia | 2012 LFS, (34531); 2013 LFS, (33744); 2014 LFS, (40212); 2016 LFS, (47320); 2018 LFS, (40993) | Namibia Statistics Agency |
| Nauru | 2013 HIES, (3150) | Nauru Statistics |
| Nepal | 2011 GALLUP, (1000); 2012 GALLUP, (2000); 2013 GALLUP, (1050); 2014 GALLUP, (1050); 2008 LFS, (76208); 2017 LFS, (77638); 2013 SWTS, (3584) | Central Bureau Of Statistics, Nepal; Gallup |
| Netherlands | 1983 EULFS, (170910); 1985 EULFS, (163616); 1987 EULFS, (52632); 1988 EULFS, (78284); 1989 EULFS, (79578); 1990 EULFS, (77210); 1991 EULFS, (76260); 1992 EULFS, (77350); 1993 EULFS, (74968); 1994 EULFS, (82084); 1995 EULFS, (87311); 1996 EULFS, (77808); 1997 EULFS, (89842); 1998 EULFS, (55959); 1999 EULFS, (51929); 2000 EULFS, (66992); 2001 EULFS, (90264); 2002 EULFS, (97594); 2003 EULFS, (96740); 2004 EULFS, (112913); 2005 EULFS, (469514); 2006 EULFS, (108545); 2007 EULFS, (106472); 2008 EULFS, (109585); 2009 EULFS, (93491); 2010 EULFS, (82389); 2011 EULFS, (87876); 2012 EULFS, (81733); 2013 EULFS, (83840); 2014 EULFS, (76990); 2015 EULFS, (73768); 2016 EULFS, (74078); 2017 EULFS, (71557) | Statistics Netherlands |
| New Zealand | 2011 GALLUP, (1000); 2012 GALLUP, (1008); 2013 GALLUP, (500); 2014 GALLUP, (2001) | Gallup |
| Nicaragua | 2012 Continuous Employment Survey (ECH), (123958); 2014 Nicaraguan Living Standards Measurement Survey (EMNV), (29381) | National Institute Of Information Development Of Nicaragua |
| Niger | 2011 ECVM, (24791); 2014 ECVM, (23231); 2012 ENESI, (23343); 2009 ENTE, (20108) | INS - Niger |
| Nigeria | 2011 General household survey (GHS), (28253); 2013 GHS, (29032); 2016 GHS, (32863) | National Bureau Of Statistics, Nigeria |
| Norway | 1995 EULFS, (30673); 1996 EULFS, (21551); 1997 EULFS, (21362); 1998 EULFS, (20664); 1999 EULFS, (20717); 2000 EULFS, (21095); 2001 EULFS, (20213); 2002 EULFS, (20838); 2003 EULFS, (20931); 2004 EULFS, (21306); 2005 EULFS, (85331); 2006 EULFS, (20663); 2007 EULFS, (20785); 2008 EULFS, (20163); 2009 EULFS, (20422); 2010 EULFS, (19985); 2011 EULFS, (19737); 2012 EULFS, (19254); 2013 EULFS, (23669); 2014 EULFS, (19138); 2015 EULFS, (19328); 2016 EULFS, (19743); 2017 EULFS, (20450) | Statistics Norway |
| Pakistan | 2011 GALLUP, (1000); 2012 GALLUP, (3012); 2013 GALLUP, (1000); 2014 GALLUP, (1000); 2006 LFS, (219969); 2007 LFS, (224280); 2008 LFS, (250820); 2009 LFS, (249216); 2010 LFS, (245521); 2011 LFS, (243659); 2012 LFS, (114805); 2013 LFS, (228224); 2014 LFS, (258490); 2015 LFS, (264136); 2017 LFS, (135455); 2018 LFS, (272490) | Pakistan Bureau Of Statistics; Gallup |
| Palau | 2000 Census (CENSUS), (15163); 2014 HIES, (2785) | Bureau Of Budget And Planning |
| Panama | 2010 Labor Market Survey (EML), (48881); 2011 EML, (46612); 2012 EML, (45636); 2013 EML, (44237); 2014 EML, (43719); 2015 EML, (42396); 2016 EML, (42234); 2017 EML, (40136); 2018 EML, (39218); 2010 ETI, (47438); 2012 ETI, (44425); 2014 ETI, (46066) | National Statistics And Censuses Institute |
| Papua New Guinea | 2010 HIES, (22550) | National Statistical Office, Papua New Guinea |
| Paraguay | 2011 EANNA, (8016); 2010 ECE, (11333); 2011 ECE, (11385); 2012 ECE, (11213); 2013 ECE, (11719); 2014 ECE, (11233); 2015 ECE, (11041); 2016 ECE, (10224); 2002 EPH, (17600); 2003 EPH, (43161); 2004 EPH, (34636); 2005 EPH, (19579); 2006 EPH, (22733); 2007 EPH, (21053); 2008 EPH, (19416); 2010 EPH, (20475); 2011 EPH, (19740); 2012 EPH, (21151); 2013 EPH, (21207); 2014 EPH, (20272); 2015 EPH, (30898); 2016 EPH, (37814); 2017 EPH, (35215); 2017 Household Survey (EPHC), (73643); 2018 EPHC, (55453) | General Directorate Of Statistics, Surveys And Censuses |
| Peru | 2002 ENAHO, (78396); 2003 ENAHO, (53230); 2004 ENAHO, (81743); 2005 ENAHO, (81188); 2006 ENAHO, (83697); 2007 ENAHO, (87924); 2008 ENAHO, (84703); 2009 ENAHO, (85331); 2010 ENAHO, (83373); 2011 ENAHO, (94697); 2012 ENAHO, (98828); 2013 ENAHO, (112307); 2014 ENAHO, (111741); 2015 ENAHO, (113605); 2016 ENAHO, (134235); 2017 ENAHO, (119188); 2001 Permanent Employment Survey (EPE), (61155); 2002 EPE, (86228); 2003 EPE, (78949); 2004 EPE, (79311); 2005 EPE, (79869); 2006 EPE, (73221); 2007 EPE, (37537); 2008 EPE, (37332); 2009 EPE, (36737); 2010 EPE, (35485); 2011 EPE, (55712); 2012 EPE, (53993); 2013 EPE, (53125); 2014 EPE, (51326); 2015 EPE, (51933); 2016 EPE, (51874); 2017 EPE, (49551); 2018 EPE, (49086); 2013 SWTS, (2464) | Instituto Nacional De Estadística E Informática |
| Philippines | 2011 GALLUP, (1000); 2012 GALLUP, (2000); 2013 GALLUP, (1000); 2014 GALLUP, (1000); 2001 LFS, (805548); 2002 LFS, (744852); 2003 LFS, (828408); 2004 LFS, (843973); 2005 LFS, (826148); 2006 LFS, (801161); 2007 LFS, (787319); 2008 LFS, (784460); 2009 LFS, (789999); 2010 LFS, (781809); 2011 LFS, (792937); 2012 LFS, (801983); 2013 LFS, (802941); 2014 LFS, (789164); 2015 LFS, (804150); 2016 LFS, (738225); 2017 LFS, (708288); 2018 LFS, (709342) | Philippine Statistics Authority; Gallup |
| Poland | 1997 EULFS, (54583); 1998 EULFS, (54545); 1999 EULFS, (53300); 2000 EULFS, (46295); 2001 EULFS, (57575); 2002 EULFS, (58623); 2003 EULFS, (58303); 2004 EULFS, (57433); 2005 EULFS, (185148); 2006 EULFS, (215383); 2007 EULFS, (205051); 2008 EULFS, (203974); 2009 EULFS, (208559); 2010 EULFS, (403423); 2011 EULFS, (410077); 2012 EULFS, (408094); 2013 EULFS, (378531); 2014 EULFS, (352437); 2015 EULFS, (330223); 2016 EULFS, (309894); 2017 EULFS, (290507) | Central Statistical office of Poland |
| Portugal | 1986 EULFS, (92655); 1987 EULFS, (90904); 1988 EULFS, (89414); 1989 EULFS, (84800); 1990 EULFS, (83746); 1991 EULFS, (84289); 1992 EULFS, (48566); 1993 EULFS, (48776); 1994 EULFS, (47029); 1995 EULFS, (43933); 1996 EULFS, (43770); 1997 EULFS, (44044); 1998 EULFS, (50067); 1999 EULFS, (47315); 2000 EULFS, (45626); 2001 EULFS, (45681); 2002 EULFS, (45617); 2003 EULFS, (46385); 2004 EULFS, (50714); 2005 EULFS, (190128); 2006 EULFS, (179534); 2007 EULFS, (171960); 2008 EULFS, (167970); 2009 EULFS, (164964); 2010 EULFS, (161168); 2011 EULFS, (159736); 2012 EULFS, (159948); 2013 EULFS, (159659); 2014 EULFS, (167243); 2015 EULFS, (166495); 2016 EULFS, (162885); 2017 EULFS, (158200); 1998 Employment Survey (IE), (196867); 1999 IE, (187397); 2000 IE, (180699); 2001 IE, (180413); 2002 IE, (179218); 2003 IE, (188508); 2004 IE, (199780); 2005 IE, (190128); 2006 IE, (179534); 2007 IE, (171960); 2008 IE, (167970); 2009 IE, (164964); 2010 IE, (161168); 2011 IE, (159736); 2012 IE, (159948); 2013 IE, (159659); 2014 IE, (167243); 2015 IE, (166495); 2016 IE, (162885); 2017 IE, (158200) | National Statistics Institute |
| Republic of Korea | 2015 Economically Active Population Survey (EAPS), (733868); 2016 EAPS, (716758); 2017 EAPS, (726967); 2018 EAPS, (731852); 2011 GALLUP, (1001); 2012 GALLUP, (2000); 2013 GALLUP, (1000); 2014 GALLUP, (2000); 2016 Local Area Labour Force Survey (LALFS), (780574) | Statistics Korea; Gallup |
| Republic of Moldova | 2009 CAS, (19193); 2010 LFS, (35683); 2011 LFS, (35369); 2012 LFS, (32937); 2013 LFS, (33092); 2014 LFS, (32775); 2015 LFS, (25146); 2016 LFS, (23708); 2017 LFS, (23648); 2018 LFS, (23386); 2018 NLFS, (52560); 2015 SWTS, (1189) | NBS Moldova |
| Romania | 1997 EULFS, (49473); 1998 EULFS, (47718); 1999 EULFS, (46261); 2000 EULFS, (44805); 2001 EULFS, (43742); 2002 EULFS, (41757); 2003 EULFS, (41556); 2004 EULFS, (68596); 2005 EULFS, (269408); 2006 EULFS, (257982); 2007 EULFS, (249521); 2008 EULFS, (240785); 2009 EULFS, (239426); 2010 EULFS, (240404); 2011 EULFS, (238807); 2012 EULFS, (230669); 2013 EULFS, (226205); 2014 EULFS, (225202); 2015 EULFS, (239124); 2016 EULFS, (231785); 2017 EULFS, (230482); 2000 LFS, (177649); 2001 LFS, (173740); 2002 LFS, (168942); 2003 LFS, (164430); 2004 LFS, (270742); 2005 LFS, (265018); 2006 LFS, (252797); 2007 LFS, (243397); 2008 LFS, (234156); 2009 LFS, (233993); 2010 LFS, (240404); 2011 LFS, (238807); 2012 LFS, (230669); 2013 LFS, (226205); 2014 LFS, (225202); 2015 LFS, (239124); 2016 LFS, (231785); 2017 LFS, (230482); 2018 LFS, (228114) | NSI Romania |
| Russian Federation | 2010 LFS, (831020); 2011 LFS, (820578); 2012 LFS, (820536); 2013 LFS, (820614); 2014 LFS, (821471); 2015 LFS, (837401); 2016 LFS, (836632); 2017 LFS, (924761); 2018 LFS, (924880); 2012 SWTS, (3890); 2015 SWTS, (3415) | Russian Federal State Statistics Service |
| Rwanda | 2014 Integrated Household Living Conditions Survey (EICV), (66081); 2017 LFS, (77761); 2018 LFS, (76670); 2008 NCLS, (28038) | National Institute Of Statistics Rwanda |
| Samoa | 2012 LFS, (16004); 2017 LFS, (16942); 2012 SWTS, (2914) | Samoa Bureau Of Statistics |
| Senegal | 2015 Quarterly National Employment Survey (ENES), (36446); 2016 ENES, (80947); 2017 ENES, (63265) | National Agency Of Statistics And Demography |
| Serbia | 2008 LFS, (41771); 2009 LFS, (40329); 2010 LFS, (43139); 2011 LFS, (44831); 2012 LFS, (44113); 2013 LFS, (47844); 2014 LFS, (95026); 2015 LFS, (113890); 2016 LFS, (133704); 2017 LFS, (130284); 2018 LFS, (131447); 2015 SWTS, (3508) | Statistical Office Of The Republic Of Serbia |
| Seychelles | 2014 LFS, (12594); 2015 LFS, (16165); 2016 LFS, (12405); 2017 LFS, (13662); 2018 LFS, (11506) | National Bureau Of Statistics Seychelles |
| Sierra Leone | 2014 LFS, (25641); 2015 SWTS, (2707) | Statistics Sierra Leone |
| Singapore | 2011 GALLUP, (1000); 2013 GALLUP, (1000) | Gallup |
| Slovakia | 1998 EULFS, (31301); 1999 EULFS, (30442); 2000 EULFS, (30846); 2001 EULFS, (30554); 2002 EULFS, (29420); 2003 EULFS, (28889); 2004 EULFS, (28772); 2005 EULFS, (116250); 2006 EULFS, (113417); 2007 EULFS, (110377); 2008 EULFS, (109840); 2009 EULFS, (105014); 2010 EULFS, (104685); 2011 EULFS, (104274); 2012 EULFS, (101469); 2013 EULFS, (100810); 2014 EULFS, (97958); 2015 EULFS, (93459); 2016 EULFS, (92759); 2017 EULFS, (89462); 2010 LFS, (97106); 2011 LFS, (96965); 2012 LFS, (93811); 2013 LFS, (93232); 2014 LFS, (90567); 2015 LFS, (86225); 2016 LFS, (85087) | Statistical Office Of The Slovak Republic |
| Slovenia | 1996 EULFS, (24640); 1997 EULFS, (18318); 1998 EULFS, (17976); 1999 EULFS, (19627); 2000 EULFS, (18751); 2001 EULFS, (19607); 2002 EULFS, (19766); 2003 EULFS, (19881); 2004 EULFS, (18871); 2005 EULFS, (71406); 2006 EULFS, (69813); 2007 EULFS, (67877); 2008 EULFS, (65600); 2009 EULFS, (65919); 2010 EULFS, (64599); 2011 EULFS, (61888); 2012 EULFS, (58415); 2013 EULFS, (61223); 2014 EULFS, (62543); 2015 EULFS, (60979); 2016 EULFS, (62887); 2017 EULFS, (64849) | Statistical Office Of The Republic of Slovenia |
| Solomon Islands | 2013 HIES, (25732) | Solomon Islands National Statistics Office |
| South Africa | 2000 Quarterly Labour Force Survey (QLFS), (143886); 2001 QLFS, (214147); 2002 QLFS, (211850); 2003 QLFS, (199567); 2004 QLFS, (208087); 2005 QLFS, (219622); 2006 QLFS, (215194); 2007 QLFS, (215447); 2008 QLFS, (375918); 2009 QLFS, (359465); 2010 QLFS, (342466); 2011 QLFS, (333939); 2012 QLFS, (339858); 2013 QLFS, (346687); 2014 QLFS, (341320); 2015 QLFS, (286782); 2016 QLFS, (275306); 2017 QLFS, (275968); 2018 QLFS, (275382) | Statistics South Africa |
| Spain | 1999 Labour Force Survey (EPA), (775331); 2000 EPA, (719612); 2001 EPA, (692188); 2002 EPA, (693004); 2003 EPA, (698682); 2004 EPA, (694869); 2005 EPA, (610851); 2006 EPA, (637480); 2007 EPA, (663556); 2008 EPA, (668794); 2009 EPA, (683852); 2010 EPA, (689204); 2011 EPA, (678593); 2012 EPA, (682911); 2013 EPA, (684398); 2014 EPA, (677373); 2015 EPA, (658968); 2016 EPA, (641170); 2017 EPA, (637152); 2018 EPA, (646763); 1986 EULFS, (199257); 1987 EULFS, (184972); 1988 EULFS, (198310); 1989 EULFS, (199553); 1990 EULFS, (201640); 1991 EULFS, (199613); 1992 EULFS, (196715); 1993 EULFS, (190708); 1994 EULFS, (190737); 1995 EULFS, (192743); 1996 EULFS, (191097); 1997 EULFS, (189898); 1998 EULFS, (190911); 1999 EULFS, (196532); 2000 EULFS, (180853); 2001 EULFS, (173643); 2002 EULFS, (172552); 2003 EULFS, (175209); 2004 EULFS, (175159); 2005 EULFS, (610851); 2006 EULFS, (100506); 2007 EULFS, (101753); 2008 EULFS, (103144); 2009 EULFS, (104354); 2010 EULFS, (108253); 2011 EULFS, (103644); 2012 EULFS, (107969); 2013 EULFS, (105786); 2014 EULFS, (105914); 2015 EULFS, (106381); 2016 EULFS, (100156); 2017 EULFS, (98743) | National Statistics Institute |
| Sri Lanka | 2009 CAS, (65866); 2011 GALLUP, (1000); 2012 GALLUP, (2031); 2013 GALLUP, (1030); 2014 GALLUP, (1062); 2010 LFS, (69201); 2011 LFS, (56172); 2013 LFS, (79297); 2014 LFS, (81376); 2015 LFS, (82800); 2016 LFS, (85082) | Department Of Census And Statistics-Sri Lanka; Gallup |
| Sweden | 1995 EULFS, (16650); 1996 EULFS, (16244); 1997 EULFS, (16069); 1998 EULFS, (15810); 1999 EULFS, (17909); 2000 EULFS, (17321); 2001 EULFS, (50512); 2002 EULFS, (55699); 2003 EULFS, (56316); 2004 EULFS, (52185); 2005 EULFS, (148124); 2006 EULFS, (209871); 2007 EULFS, (208058); 2008 EULFS, (205262); 2009 EULFS, (201336); 2010 EULFS, (260251); 2011 EULFS, (252642); 2012 EULFS, (248236); 2013 EULFS, (241152); 2014 EULFS, (227392); 2015 EULFS, (212943); 2016 EULFS, (202839); 2017 EULFS, (202127) | Statistics Sweden |
| Switzerland | 1991 Swiss Labor Force Survey (ESPA), (15965); 1992 ESPA, (16892); 1993 ESPA, (18069); 1994 ESPA, (17869); 1995 ESPA, (31754); 1996 ESPA, (16218); 1997 ESPA, (16189); 1998 ESPA, (16306); 1999 ESPA, (17720); 2000 ESPA, (17733); 2001 ESPA, (18738); 2002 ESPA, (41263); 2003 ESPA, (57679); 2004 ESPA, (54229); 2005 ESPA, (51791); 2006 ESPA, (48262); 2007 ESPA, (48485); 2008 ESPA, (47899); 2009 ESPA, (49390); 2010 ESPA, (67088); 2011 ESPA, (71872); 2012 ESPA, (74193); 2013 ESPA, (71705); 2014 ESPA, (68892); 2015 ESPA, (66608); 2016 ESPA, (62732); 2017 ESPA, (63495); 2018 ESPA, (64727); 1996 EULFS, (16186); 1997 EULFS, (16188); 1998 EULFS, (16306); 1999 EULFS, (17720); 2000 EULFS, (17733); 2001 EULFS, (18738); 2002 EULFS, (41263); 2003 EULFS, (57679); 2004 EULFS, (54229); 2005 EULFS, (51791); 2006 EULFS, (48262); 2007 EULFS, (48485); 2008 EULFS, (47899); 2009 EULFS, (49390); 2010 EULFS, (67088); 2011 EULFS, (71872); 2012 EULFS, (74193); 2013 EULFS, (71705); 2014 EULFS, (68892); 2015 EULFS, (66608); 2016 EULFS, (62732); 2017 EULFS, (63495) | Federal Statistical Office |
| Tajikistan | 2009 LSS, (10069); | Agency On Statistics Under The President Of The Republic Of Tajikistan |
| Thailand | 2011 GALLUP, (1000); 2012 GALLUP, (2000); 2013 GALLUP, (1000); 2014 Informal Employment Survey (IES), (232710); 2015 IES, (229332); 2016 IES, (230798); 2017 IES, (227229); 2018 IES, (222537); 2010 LFS, (815818); 2013 LFS, (899961); 2014 LFS, (889721); 2015 LFS, (876636); 2016 LFS, (877066); 2017 LFS, (867713); 2018 LFS, (849683); 2014 Labour Force Survey (LFSMS) (LFSMS), (233048); 2015 LFSMS, (219433); 2016 LFSMS, (215997); 2017 LFSMS, (213746); 2018 LFSMS, (209747) | National Statistical Office Of Thailand; Gallup |
| Timor-Leste | 2010 LFS, (24059); 2013 LFS, (33136); 2016 LFS, (9936); | General Directorate Of Statistics, Timor-Leste Ministry Of Finance |
| Togo | 2009 ENTE, (5448); 2017 ERIESI, (22205); 2011 QUIBB, (29781); 2015 QUIBB, (11513); 2012 SWTS, (2033); 2014 SWTS, (2708) | National Institute Of Statistics And Economic And Demographic Studies |
| Tonga | 2018 LFS, (12089); | Tonga Statistics Department |
| Tunisia | 2010 National survey on population and employment (ENPE), (548244); 2013 SWTS, (3000) | National Institute Of Statistics - Tunisia |
| Turkey | 2004 LFS, (472837); 2005 LFS, (490040); 2006 LFS, (497137); 2007 LFS, (481605); 2008 LFS, (481154); 2009 LFS, (503329); 2010 LFS, (522171); 2011 LFS, (517076); 2012 LFS, (510807); 2013 LFS, (502426); 2014 LFS, (393822); 2015 LFS, (389035); 2016 LFS, (380709); 2017 LFS, (378691) | Turkish Statistical Institute |
| Tuvalu | 2016 HIES, (4418) | Tuvalu Statistics |
| Uganda | 2012 LFS, (31779); 2013 SWTS, (3811); 2015 SWTS, (3049) | Uganda Bureau Of Statistics |
| Ukraine | 2013 SWTS, (3526); 2015 SWTS, (3202) | State Statistics Service Of Ukraine |
| United Arab Emirates | 2017 LFS, (71284); 2018 LFS, (64392) | Federal Competitiveness And Statistics Authority |
| United Kingdom of Great Britain and Northern Ireland | 1983 EULFS, (217772); 1984 EULFS, (164692); 1985 EULFS, (167188); 1986 EULFS, (167245); 1987 EULFS, (163886); 1988 EULFS, (166456); 1989 EULFS, (166433); 1990 EULFS, (161772); 1991 EULFS, (159129); 1992 EULFS, (159601); 1993 EULFS, (163164); 1994 EULFS, (159445); 1995 EULFS, (153761); 1996 EULFS, (152116); 1997 EULFS, (148091); 1998 EULFS, (144979); 1999 EULFS, (143058); 2000 EULFS, (140067); 2001 EULFS, (135887); 2002 EULFS, (136155); 2003 EULFS, (130417); 2004 EULFS, (125610); 2005 EULFS, (123141); 2006 EULFS, (120651); 2007 EULFS, (120379); 2008 EULFS, (100136); 2009 EULFS, (95254); 2010 EULFS, (93215); 2011 EULFS, (88824); 2012 EULFS, (86284); 2013 EULFS, (84041); 2014 EULFS, (86405); 2015 EULFS, (82229); 2016 EULFS, (80193); 2017 EULFS, (81073); 1992 LFS, (458194); 1993 LFS, (605737); 1994 LFS, (602628); 1995 LFS, (621110); 1996 LFS, (614386); 1997 LFS, (601001); 1998 LFS, (590204); 1999 LFS, (580659); 2000 LFS, (564088); 2001 LFS, (551406); 2002 LFS, (538343); 2003 LFS, (516825); 2004 LFS, (374072); 2005 LFS, (489846); 2006 LFS, (479604); 2007 LFS, (479382); 2008 LFS, (471586); 2009 LFS, (454114); 2010 LFS, (428779); 2011 LFS, (404533); 2012 LFS, (403325); 2013 LFS, (396809); 2014 LFS, (389637); 2015 LFS, (375702); 2016 LFS, (361663); 2017 LFS, (353954); 2018 LFS, (350706) | Office For National Statistics |
| United Republic of Tanzania | 2001 LFS, (43558); 2006 LFS, (74371); 2014 LFS, (47199); 2013 SWTS, (1988) | Tanzania National Bureau Of Statistics |
| United States of America | 1994 Current Population Survey (CPS), (1847463); 1995 CPS, (1823354); 1996 CPS, (1616150); 1997 CPS, (1620976); 1998 CPS, (1621980); 1999 CPS, (1630445); 2000 CPS, (1622588); 2001 CPS, (1742243); 2002 CPS, (1904485); 2003 CPS, (1893968); 2004 CPS, (1863921); 2005 CPS, (1852282); 2006 CPS, (1840362); 2007 CPS, (1830401); 2008 CPS, (1819125); 2009 CPS, (1830865); 2010 CPS, (1838262); 2011 CPS, (1822867); 2012 CPS, (1814235); 2013 CPS, (1808433); 2014 CPS, (1821449); 2015 CPS, (1811993); 2016 CPS, (1809232); 2017 CPS, (1780227); 2018 CPS, (1727277) | Census Bureau |
| Uruguay | 1998 ECH, (56854); 1999 ECH, (57674); 2000 ECH, (57982); 2001 ECH, (57410); 2002 ECH, (56333); 2003 ECH, (55369); 2004 ECH, (55587); 2005 ECH, (54330); 2006 ECH, (256861); 2007 ECH, (143185); 2008 ECH, (144582); 2009 ECH, (132599); 2010 ECH, (132010); 2011 ECH, (130804); 2012 ECH, (120462); 2013 ECH, (127925); 2014 ECH, (131857); 2015 ECH, (121461); 2016 ECH, (118591); 2017 ECH, (118268); 2018 ECH, (108608); 2010 ENTI, (10209) | Ine Uruguay |
| Venezuela (Bolivarian Republic of) | 2005 Household Survey (EHM), (330293); 2006 EHM, (333902); 2007 EHM, (339556); 2008 EHM, (331616); 2009 EHM, (318378); 2010 EHM, (304885); 2011 EHM, (307198); 2012 EHM, (309760) | Instituto Nacional De Estadística |
| Viet Nam | 2011 GALLUP, (1000); 2012 GALLUP, (2000); 2013 GALLUP, (1017); 2007 LFS, (661321); 2009 LFS, (66187); 2010 LFS, (916894); 2011 LFS, (1110451); 2012 LFS, (746768); 2013 LFS, (747807); 2014 LFS, (746978); 2015 LFS, (819537); 2016 LFS, (814611); 2017 LFS, (825102); 2018 LFS, (824143); 2012 NCLS, (41459); 2013 SWTS, (2722); 2015 SWTS, (2229) | General Statistics Office Of Vietnam; Gallup |
| Yemen | 2014 LFS, (85850); 2010 NCLS, (67617) | Central Statistical Office, Government Of Yemen |
| Zambia | 2017 LFS, (45685); 2012 SWTS, (3206); 2014 SWTS, (3225) | Central Statistical Office Of Zambia |
| Zimbabwe | 2011 LFS, (39798); 2014 LFS, (40527) | Zimbabwe National Statistics |

Footnote: LFS = Labour Force Survey

**Tables S4 and S5: Detailed description and meta-data on the WHO/ILO Longitudinal Working Hours Database**

The WHO/ILO Longitudinal Working Hours Database contains information on 46 countries for 444 country/year pair observations (Table S4). Each of these country/year pairs contain 36 individual transition probabilities for the 36 working hours transitions in principle. Moreover, additional breakdowns by sex and 5-year wide age groups are available. Due to insufficient sample overlap, however, from 76 country/year pairs it is impossible to calculate transition probabilities. This resulted in 47,282 useful individual observations. In total, 155 million observations from 1,742 Labour Force Survey (LFS) pooled pseudo-longitudinal samples conducted in 46 countries between 1 January 2010 and 31 December 2018 were used. This database captured 56.1% of the global population with at least one survey.

**Table S4: Coverage of survey waves and countries in the WHO/ILO Longitudinal Global Working Hours Database**

|  | **Region** (defined as per WHO classification) | | | | | | **World** |
| --- | --- | --- | --- | --- | --- | --- | --- |
|  | **Africa** | **Americas** | **South-East Asia** | **Europe** | **Eastern Mediter-ranean** | **Western Pacific** |  |
| Survey waves (N) | 66 | 428 | 45 | 1150 | 40 | 113 | 1724 |
| Countries with ≥1 survey wave (N) (% of countries) | 2 (4.3%) | 8 (24.2%) | 2 (18.2%) | 30 (60.0%) | 1 (4.6%) | 3 (14.3%) | 46  (23.6%) |

**Table S5: Estimates based on pooled pseudo-longitudinal transition data were derived from overlapping Labour Force Survey samples from the following countries and years**

| **Country** | **LFS pooled pseudo-longitudinal samples** | **Pooled sample size** | **Number of underlying quarterly datasets used** |
| --- | --- | --- | --- |
| Argentina | 2003–2004, 2004–2005, 2005–2006, 2006–2007, 2007–2008, 2008–2009, 2009–2010, 2010–2011, 2011–2012, 2012–2013, 2013–2014, 2014–2015, 2015–2016, 2016–2017, 2017–2018 | 3 288 964 | 58 |
| Austria | 2010–2011, 2011–2012, 2012–2013, 2013–2014, 2014–2015, 2015–2016, 2016–2017, 2017–2018 | 196 725 | 36 |
| Brazil | 2012–2013, 2013–2014, 2014–2015, 2015–2016, 2016–2017, 2017–2018, 2018–2019 | 16 444 204 | 29 |
| Bulgaria | 2010–2011, 2011–2012, 2012–2013, 2013–2014, 2014–2015, 2015–2016, 2016–2017, 2017–2018 | 354 072 | 36 |
| Cambodia | 2007–2008, 2010–2011, 2011–2012, 2012–2013, 2013–2014, 2014–2015, 2015–2016 | 190 278 | 9 |
| Chile | 2010–2011, 2011–2012, 2012–2013, 2013–2014, 2014–2015, 2015–2016, 2016–2017, 2017–2018, 2018–2019 | 4 072 879 | 38 |
| Costa Rica | 2010–2011, 2011–2012, 2012–2013, 2013–2014, 2014–2015, 2015–2016, 2016–2017, 2017–2018, 2018–2019 | 911 456 | 35 |
| Croatia | 2010–2011, 2011–2012, 2012–2013, 2014–2015, 2015–2016, 2016–2017, 2017–2018 | 84 703 | 32 |
| Cyprus | 2010–2011, 2011–2012, 2012–2013, 2013–2014, 2014–2015, 2015–2016, 2016–2017, 2017–2018 | 74 759 | 36 |
| Czechia | 2010–2011, 2011–2012, 2012–2013, 2013–2014, 2014–2015, 2015–2016, 2016–2017, 2017–2018 | 249 533 | 36 |
| Denmark | 2010–2011, 2011–2012, 2012–2013, 2013–2014, 2014–2015, 2015–2016, 2016–2017, 2017–2018 | 251 717 | 36 |
| Ecuador | 2003–2004, 2004–2005, 2005–2006, 2006–2007, 2007–2008, 2008–2009, 2009–2010, 2010–2011, 2011–2012, 2012–2013, 2013–2014, 2014–2015, 2015–2016, 2016–2017, 2017–2018, 2018–2019 | 2 958 630 | 40 |
| Egypt | 2008–2009, 2009–2010, 2010–2011, 2011–2012, 2012–2013, 2013–2014, 2014–2015, 2015–2016, 2016–2017 | 3 500 944 | 40 |
| Estonia | 2010–2011, 2011–2012, 2012–2013, 2013–2014, 2014–2015, 2015–2016, 2016–2017, 2017–2018 | 65 527 | 36 |
| Finland | 2010–2011, 2011–2012, 2012–2013, 2013–2014, 2014–2015, 2015–2016, 2016–2017, 2017–2018 | 294 204 | 36 |
| France | 2010–2011, 2011–2012, 2012–2013, 2013–2014, 2014–2015, 2015–2016, 2016–2017, 2017–2018 | 797 215 | 36 |
| Germany | 2010–2011, 2011–2012, 2012–2013, 2013–2014, 2014–2015, 2016–2017, 2017–2018 | 1 848 640 | 32 |
| Greece | 2010–2011, 2011–2012, 2012–2013, 2013–2014, 2014–2015, 2015–2016, 2016–2017, 2017–2018 | 437 673 | 36 |
| Hungary | 2010–2011, 2011–2012, 2012–2013, 2013–2014, 2014–2015, 2015–2016, 2016–2017, 2017–2018 | 412 342 | 36 |
| Indonesia | 2008–2009, 2009–2010, 2010–2011, 2011–2012, 2012–2013, 2013–2014, 2014–2015, 2015–2016, 2016–2017, 2017–2018 | 7 707 743 | 21 |
| Ireland | 2010–2011, 2011–2012, 2012–2013, 2013–2014, 2014–2015, 2015–2016, 2016–2017, 2017–2018 | 141 984 | 32 |
| Island | 2010–2011, 2011–2012, 2012–2013, 2013–2014, 2014–2015, 2015–2016, 2016–2017, 2017–2018 | 33 143 | 36 |
| Italy | 2010–2011, 2011–2012, 2012–2013, 2013–2014, 2014–2015, 2015–2016, 2016–2017, 2017–2018 | 1 523 421 | 36 |
| Latvia | 2010–2011, 2011–2012, 2012–2013, 2013–2014, 2014–2015, 2015–2016, 2016–2017, 2017–2018 | 92 909 | 36 |
| Lithuania | 2010–2011, 2011–2012, 2012–2013, 2013–2014, 2014–2015, 2015–2016, 2016–2017, 2017–2018 | 68 551 | 36 |
| Mexico | 2005–2006, 2006–2007, 2007–2008, 2008–2009, 2009–2010, 2010–2011, 2011–2012, 2012–2013, 2013–2014, 2014–2015, 2015–2016, 2016–2017, 2017–2018, 2018–2019 | 22 644 438 | 57 |
| Netherlands | 2010–2011, 2011–2012, 2012–2013, 2013–2014, 2014–2015, 2015–2016, 2016–2017, 2017–2018 | 357 594 | 34 |
| Norway | 2010–2011, 2011–2012, 2012–2013, 2013–2014, 2014–2015, 2015–2016, 2016–2017, 2017–2018 | 198 842 | 36 |
| Peru | 2001–2002, 2002–2003, 2003–2004, 2004–2005, 2005–2006, 2006–2007, 2007–2008, 2008–2009, 2009–2010, 2010–2011, 2011–2012, 2012–2013, 2013–2014, 2014–2015, 2015–2016, 2016–2017, 2017–2018, 2018–2019 | 1 247 116 | 73 |
| Philippines | 2001, 2001, 2002, 2002, 2003, 2003, 2004, 2004, 2005, 2005, 2006, 2006, 2007, 2007, 2008, 2008, 2009, 2009, 2010, 2010, 2011, 2011, 2012, 2012, 2013, 2013, 2014, 2014, 2015, 2015, 2016, 2016, 2017, 2017, 2018, 2018 | 14 187 929 | 71 |
| Poland | 2010–2011, 2011–2012, 2012–2013, 2013–2014, 2014–2015, 2015–2016, 2016–2017, 2017–2018 | 964 287 | 36 |
| Portugal | 2011–2012, 2012–2013, 2013–2014, 2014–2015, 2015–2016, 2016–2017, 2017–2018 | 68 551 | 32 |
| Romania | 2010–2011, 2011–2012, 2012–2013, 2013–2014, 2015–2016, 2016–2017, 2017–2018 | 628 227 | 32 |
| Rwanda | 2017–2018 | 154 431 | 4 |
| Serbia | 2008–2009, 2009–2010, 2010–2011, 2011–2012, 2012–2013, 2013–2014, 2014–2015, 2015–2016, 2016–2017, 2017–2018 | 866 378 | 32 |
| Slovakia | 2010–2011, 2011–2012, 2012–2013, 2013–2014, 2014–2015, 2015–2016, 2016–2017, 2017–2018 | 114 164 | 36 |
| Slovenia | 2010–2011, 2011–2012, 2012–2013, 2013–2014, 2014–2015, 2015–2016, 2016–2017, 2017–2018 | 114 967 | 36 |
| South Africa | 2000–2001, 2001–2002, 2002–2003, 2003–2004, 2004–2005, 2005–2006, 2006–2007, 2007–2008, 2008–2009, 2009–2010, 2010–2011, 2011–2012, 2012–2013, 2013–2014, 2014–2015, 2015–2016, 2016–2017, 2017–2018, 2018–2019 | 5 315 997 | 62 |
| Spain | 2010–2011, 2011–2012, 2012–2013, 2013–2014, 2014–2015, 2015–2016, 2016–2017, 2017–2018 | 1 133 880 | 36 |
| Sweden | 2010–2011, 2011–2012, 2012–2013, 2013–2014, 2014–2015, 2015–2016, 2016–2017, 2017–2018 | 763 748 | 36 |
| Switzerland | 2010–2011, 2011–2012, 2012–2013, 2013–2014, 2014–2015, 2015–2016, 2016–2017, 2017–2018 | 429 317 | 36 |
| Thailand | 2013–202014, 2014–202015, 2015–202016, 2016–202017, 2017–2018 | 6 101 231 | 24 |
| Turkey | 2010–2011, 2011–2012, 2012–2013, 2014–2015, 2015–2016, 2016–2017, 2017–2018 | 993 315 | 32 |
| United Kingdom of Great Britain and Northern Ireland | 2010–2011, 2011–2012, 2012–2013, 2013–2014, 2014–2015, 2015–2016, 2016–2017, 2017–2018 | 271 456 | 36 |
| United States of America | 1994–1995, 1995–1996, 1996–1997, 1997–1998, 1998–1999, 1999–2000, 2000–2001, 2001–2002, 2002–2003, 2003–2004, 2004–2005, 2005–2006, 2006–2007, 2007–2008, 2008–2009, 2009–2010, 2010–2011, 2011–2012, 2012–2013, 2013–2014, 2014–2015, 2015–2016, 2016–2017, 2017–2018, 2018–2019 | 45 437 674 | 98 |
| Viet Nam | 2011–2012, 2012–2013, 2013–2014, 2014–2015, 2015–2016, 2016–2017, 2017–2018, 2018–2019 | 6 838 494 | 33 |
| **Total** |  | **154 834 252** | **1742** |

**Table S6: Proportion of population exposed to long working hours (≥55 hours/week), 2000, 2010, and 2016, and mean percentage change for 2000–2010, 2010–2016, and 2000–2016, by region and sex, 194 countries**

| **Region** | **Both sexes** | | | | | |
| --- | --- | --- | --- | --- | --- | --- |
|  | **Exposure (%) (UR)** | | | **Percent change (UR)** | | |
|  | **2000** | **2010** | **2016** | **2000–2010** | **2010–2016** | **2000–2016** |
| Africa | 17.3% (16.9–17.7) | 13.5% (13.3–13.6) | 11.4% (11.2–11.5) | -22.3% (-24.2–-20.4) | -15.6% (-17.3–-13.9) | -34.4% (-36.1–-32.6) |
| Americas | 8.8% (8.7–8.9) | 7.3% (7.2–7.4) | 6.4% (6.3–6.6) | -17.2% (-19.0–-15.4) | -12.1% (-14.9–-9.2) | -27.2% (-29.3–-25.1) |
| Eastern Mediterranean | 9.8% (9.6–10.0) | 10.7% (10.5–10.9) | 11.4% (11.1–11.6) | 9.4% (5.9–13.0) | 5.9% (2.8–9.2) | 15.9% (12.0–20.0) |
| Europe | 4.6% (4.6–4.6) | 3.9% (3.9–4.0) | 3.5% (3.5–3.6) | -14.8% (-16.1–-13.6) | -9.8% (-11.4–-8.1) | -23.1% (-24.5–-21.8) |
| South-East Asia | 11.5% (10.5–12.5) | 11.7% (11.0–12.3) | 11.7% (10.8–12.5) | 1.4% (-8.6–12.8) | 0.1% (-9.2–10.0) | 1.5% (-9.5–13.9) |
| Western Pacific | 3.9% (3.5– 4.4) | 5.9% (5.5– 6.4) | 8.4% (7.8– 9.0) | 51.7% (32.1–74.9) | 42.0% 27.8–57.9) | 115.4% (88.7–148.3) |

| **Region** | **Males** | | | | | | **Females** | | | | | |
| --- | --- | --- | --- | --- | --- | --- | --- | --- | --- | --- | --- | --- |
|  | **Exposure (%) (UR)** | | | **Percent change (UR)** | | | **Exposure (%) (UR)** | | | **Percent change (UR)** | | |
|  | **2000** | **2010** | **2016** | **2000–2010** | **2010–2016** | **2000–2016** | **2000** | **2010** | **2016** | **2000–2010** | **2010–2016** | **2000–2016** |
| Africa | 21.5% (20.9–22.1) | 17.4% (17.1–17.7) | 15.3% (15.0–15.6) | -18.9%  (-21.4–  -16.2) | -12.4% (-14.6–  -10.1) | -28.9% (-31.3–  -26.5) | 13.3% (12.8–13.7) | 9.6%  (9.4–  9.8) | 7.5% (7.3–7.7) | -27.7%  (-30.4–  -24.7) | -21.5%  (-24.1–  -18.8) | -43.2%  (-45.6–  -40.8) |
| Americas | 13.2% (13.1–13.4) | 10.8% (10.6–11.0) | 9.4% (9.1–9.7) | -18.4%  (-20.2–  -16.5) | -12.9% (-16.0–  -9.8) | -28.9% (-31.1–  -26.7) | 4.6% (4.5–  4.8) | 4.0% (3.8–  4.1) | 3.6% (3.4–3.8) | -14.3%  (-18.5–  -10.0) | -9.8%  (-15.9–  -3.6) | -22.7%  (-27.3–  -17.9) |
| Eastern Mediterranean | 17.4% (17.0–17.9) | 19.5% (19.1–20.0) | 21.0% (20.5–21.5) | 12.1% (8.4–16.0) | 7.5% (4.0–11.0) | 20.4% (16.2–24.8) | 1.8% (1.7–  1.9) | 1.3% (1.3–  1.4) | 1.0% (1.0–1.1) | -26.4%  (-32.5–  -19.5) | -21.3%  (-27.3–  -15.0) | -42.1%  (-47.3–  -36.3) |
| Europe | 7.5% (7.4–7.5) | 6.2% (6.1–6.2) | 5.4% (5.3–5.5) | -17.5%  (-18.9–  -15.9) | -11.9% (-13.9–  -9.9) | -27.3% (-28.8–  -25.7) | 2.0% (2.0–  2.0) | 1.9% (1.8–  1.9) | 1.8% (1.8–1.9) | -6.5%  (-8.8–  -4.1) | -3.5%  (-6.3–  -0.7) | -9.8%  (-12.3–  -7.3) |
| South-East Asia | 17.9% (16.2–19.6) | 18.2% (17.0–19.3) | 18.1% (16.5–19.6) | 1.5%  (-9.2–14.1) | -0.4% (-10.8–10.7) | 1.1%  (-11.0–14.8) | 4.9% (3.9–  5.9) | 4.9% (4.2–  5.6) | 5.0% (4.2–5.8) | 0.8%  (-21.2–  31.3) | 2.2%  (-18.0–  26.8) | 3.0%  (-19.7–  35.1) |
| Western Pacific | 4.5% (3.9, 5.2) | 7.4% (6.6, 8.1) | 11.1% (10.1, 12.1) | 62.8% (37.6–95.8) | 50.6% (32.1–72.0) | 145.2% (107.9–192.5) | 3.3% (2.6–  3.9) | 4.4% (3.9–  5.0) | 5.7% (5.0–6.3) | 36.0%  (8.6–  74.9) | 27.6%  (7.7–  51.7) | 73.5%  (39.9–  120.4) |

Footnote: UR = 95% uncertainty range

**Table S7: Population-attributable fractions for ischemic heart disease attributable to exposure to long working hours (≥55 hours/week), by region and sex, 183 countries**

|  | **Region** | **Both sexes** | | | | | |
| --- | --- | --- | --- | --- | --- | --- | --- |
|  |  | **PAF (%) (UR)** | | | **Percent change (UR)** | | |
|  |  | **2000** | **2010** | **2016** | **2000–2010** | **2010–2016** | **2000–2016** |
| Deaths | Africa | 3.4 (3.2–3.6) | 3.3 (3.1–3.6) | 3.3 (3.1–3.5) | -2.4 (-10.8–6.7) | -0.6 (-9.7–9.4) | -2.9 (-11.7–6.9) |
|  | Americas | 2.2 (2.0–2.3) | 2.3 (2.1–2.5) | 2.3 (2.1–2.5) | 6.0 (-5.8–18.9) | 0.5 (-10.4–12.4) | 6.4 (-5.2–19.5) |
|  | Eastern Mediterranean | 6.0 (5.5–6.5) | 5.7 (5.2–6.2) | 5.5 (5.0–6.1) | -5.1 (-16.5–7.8) | -2.5 (-14.9–11.4) | -7.5 (-19.2–5.4) |
|  | Europe | 1.8 (1.6–1.9) | 1.6 (1.4–1.7) | 1.4 (1.3–1.5) | -11.5 (-21.0–-1.2) | -8.2 (-18.0–2.5) | -18.7 (-27.1–-9.6) |
|  | South-East Asia | 7.2 (6.2–8.4) | 7.2 (6.1–8.3) | 7.2 (6.0–8.4) | -0.2 (-19.9–24.5) | -0.7 (-20.4–24.9) | -0.9 (-20.9–23.7) |
|  | Western Pacific | 3.3 (2.8–3.8) | 2.8 (2.4–3.3) | 2.7 (2.3–3.2) | -14.5 (-30.9–5.6) | -2.9 (-23.2–22.9) | -17.0 (-33.9–3.2) |
| DALYs | Africa | 3.8 (3.6–4.0) | 3.8 (3.6–4.0) | 3.9 (3.7–4.1) | 0.1 (-6.7–7.4) | 1.0 (-5.7–8.3) | 1.2 (-5.5–8.3) |
|  | Americas | 3.3 (3.0–3.6) | 3.4 (3.2–3.7) | 3.4 (3.1–3.7) | 4.8 (-6.2–17.6) | -1.4 (-11.4–9.9) | 3.4 (-7.9–15.7) |
|  | Eastern Mediterranean | 6.9 (6.4–7.4) | 6.8 (6.3–7.4) | 6.9 (6.3–7.4) | -0.7 (-10.8–10.5) | 0.2 (-10.7–12.5) | -0.5 (-10.9–10.9) |
|  | Europe | 2.6 (2.4–2.8) | 2.4 (2.2–2.6) | 2.3 (2.1–2.5) | -8.9 (-18.7–1.7) | -4.1 (-13.7–6.8) | -12.7 (-21.4–-2.4) |
|  | South-East Asia | 8.3 (7.2–9.4) | 8.4 (7.3–9.6) | 8.5 (7.3–9.7) | 1.6 (-15.8–23.0) | 0.6 (-16.9–21.7) | 2.2 (-15.9–23.6) |
|  | Western Pacific | 4.8 (4.2–5.3) | 4.3 (3.8–4.9) | 4.3 (3.7–4.9) | -9.4 (-24.5–7.6) | -1.3 (-19.2–20.2) | -10.6 (-25.8–7.6) |

|  | **Region** | **Males** | | | | | | **Females** | | | | | |
| --- | --- | --- | --- | --- | --- | --- | --- | --- | --- | --- | --- | --- | --- |
|  |  | **PAF (%) (UR)** | | | **Percent change (UR)** | | | **PAF (%) (UR)** | | | **Percent change (UR)** | | |
|  |  | **2000** | **2010** | **2016** | **2000**–**2010** | **2010**–**2016** | **2000**–**2016** | **2000** | **2010** | **2016** | **2000**–**2010** | **2010**–**2016** | **2000**–**2016** |
| Deaths | Africa | 4.6 (4.2– 4.9) | 4.6 (4.2– 4.9) | 4.6 (4.2– 5.0) | 0.0  (-11.0–12.6) | 0.6  (-11.0–13.5) | 0.6  (-11.3–13.7) | 2.3 (2.1–2.5) | 2.1 (1.9–2.3) | 2.1 (1.8–2.3) | -5.8  (-17.8–8.4) | -3.5  (-16.9–11.8) | -9.1  (-21.6–  5.0) |
|  | Americas | 3.6 (3.3– 4.0) | 3.7 (3.4– 4.1) | 3.6 (3.3– 3.9) | 1.7  (-11.2–16.0) | -2.9  (-14.8–10.4) | -1.2  (-13.6–13.4) | 0.6 (0.5–0.7) | 0.7 (0.6–0.7) | 0.7 (0.6–0.8) | 10.5  (-3.6–26.9) | 4.8  (-8.0–19.3) | 15.8 (1.2–33.1) |
|  | Eastern Mediterranean | 8.4 (7.5– 9.3) | 8.1 (7.2– 9.0) | 7.9 (7.0– 8.9) | -3.6  (-17.9–12.9) | -1.7  (-16.6–15.9) | -5.3  (-19.7–11.4) | 2.9 (2.5–3.2) | 2.6 (2.3–2.9) | 2.4 (2.1–2.8) | -10.7  (-24.7–5.5) | -4.8  (-20.4–13.5) | -15.0  (-28.6–0.7) |
|  | Europe | 3.1 (2.8– 3.4) | 2.7 (2.4– 2.9) | 2.5 (2.3– 2.7) | -12.6  (-23.6–  -0.3) | -8.0  (-19.5–4.9) | -19.6  (-29.4–  -8.4) | 0.6 (0.5–0.6) | 0.6 (0.5–0.6) | 0.5 (0.5–0.6) | -6.1  (-20.5–10.4) | -7.9  (-21.7–8.9) | -13.5  (-26.0–1.4) |
|  | South-East Asia | 9.3 (7.5–11.2) | 9.3 (7.6–11.2) | 9.2 (7.4–11.1) | 0.0  (-24.7–33.2) | -1.0  (-25.8–32.0) | -1.0  (-26.2–32.8) | 4.4 (3.5–5.3) | 4.3 (3.4–5.1) | 4.1 (3.3–5.0) | -2.4  (-26.5–29.9) | -3.9  (-28.9–28.7) | -6.2  (-30.7–26.1) |
|  | Western Pacific | 4.4 (3.6– 5.2) | 3.7 (3.0– 4.5) | 3.6 (2.8– 4.4) | -14.6  (-35.6–13.8) | -3.8  (-30.4–30.8) | -17.8  (-39.8–9.8) | 2.2 (1.7–2.6) | 1.9 (1.5–2.4) | 1.9 (1.4–2.4) | -11.5  (-35.7–20.1) | -1.4  (-30.9–39.1) | -12.7  (-38.4–20.2) |
| DALYs | Africa | 5.0 (4.7–5.3) | 5.1 (4.8–5.4) | 5.1 (4.8–5.5) | 1.6  (-7.0–11.1) | 1.6  (-6.7–10.9) | 3.3  (-5.2–12.7) | 2.4 (2.3–2.6) | 2.4 (2.2–2.5) | 2.3 (2.2–2.5) | -2.3  (-11.8–8.3) | -1.3  (-11.1–9.3) | -3.6  (-12.8–6.9) |
|  | Americas | 4.9 (4.4–5.3) | 5.0 (4.5–5.4) | 4.8 (4.4–5.2) | 2.1  (-10.3–16.6) | -3.3  (-14.7–9.6) | -1.2  (-13.4–12.5) | 1.0 (0.9–1.1) | 1.1 (1.0–1.2) | 1.1 (1.0–1.2) | 7.2  (-5.3–21.9) | 1.3  (-10.1–14.1) | 8.5  (-4.2–23.2) |
|  | Eastern Mediterranean | 9.1 (8.3–9.9) | 9.1 (8.2–10.0) | 9.2 (8.3–10.0) | -0.1  (-12.7–13.8) | 0.6  (-12.7–15.7) | 0.6  (-12.1–15.0) | 3.6 (3.2–3.9) | 3.4 (3.0–3.7) | 3.3 (3.0–3.7) | -5.1  (-17.6–9.0) | -1.5  (-15.5–14.5) | -6.6  (-19.5–7.9) |
|  | Europe | 3.9 (3.5–4.2) | 3.5 (3.2–3.8) | 3.4 (3.1–3.7) | -9.2  (-20.0–3.3) | -4.2  (-15.3–8.5) | -13.0  (-23.3–  -1.3) | 0.9 (0.8–1.0) | 0.9 (0.8–1.0) | 0.8 (0.8–0.9) | -4.1  (-17.2–11.3) | -3.3  (-16.2–12.1) | -7.2  (-19.4–7.1) |
|  | South-East Asia | 10.1 (8.4–11.8) | 10.2 (8.5–11.9) | 10.2 (8.4–12.0) | 0.9  (-20.6–27.8) | 0.0  (-22.1–28.0) | 0.9  (-21.5–29.3) | 5.5 (4.5–6.4) | 5.6 (4.6–6.6) | 5.5 (4.5–6.5) | 1.5  (-20.8–29.7) | -1.2  (-22.9–27.4) | 0.3  (-22.1–28.4) |
|  | Western Pacific | 6.0 (5.1–6.9) | 5.5 (4.6–6.4) | 5.4 (4.4–6.4) | -8.6  (-27.7–15.1) | -1.7  (-23.7–25.5) | -10.1  (-30.1–13.6) | 3.3 (2.7–3.8) | 3.0 (2.4–3.6) | 3.0 (2.4–3.6) | -8.2  (-30.1–18.4) | -0.1  (-24.9–33.5) | -8.3  (-30.2–19.8) |

Footnote: UR = 95% uncertainty range

**Table S8: Number of deaths from ischemic heart disease attributable to exposure to long working hours (≥55 hours/week), 2000, 2010, and 2016, and mean percentage change for 2000–2010, 2010–2016, and 2000–2016, by region and sex, 183 countries**

| **Region** | **Both sexes** | | | | | |
| --- | --- | --- | --- | --- | --- | --- |
|  | **Deaths (UR)** | | | **Percent change (UR)** | | |
|  | **2000** | **2010** | **2016** | **2000–2010** | **2010–2016** | **2000–2016** |
| Africa | 12,671 (11,941–13,402) | 14,662 (13,818–15,505) | 16,942 (15,878–18,005) | 15.7 (6.7–25.6) | 15.6 (6.0–25.9) | 33.7 (22.5–45.2) |
| Americas | 23,378 (21,434–25,321) | 23,329 (21,530–25,128) | 25,079 (23,095–27,063) | -0.2 (-10.7–11.7) | 7.5 (-4.0–20.1) | 7.3 (-4.5–20.6) |
| Eastern Mediterranean | 34,260 (31,363–37,158) | 40,997 (37,372–44,623) | 46,111 (41,876–50,346) | 19.7 (5.8–35.1) | 12.5 (-0.9–27.8) | 34.6 (18.2–53.0) |
| Europe | 45,452 (41,907–48,997) | 38,610 (35,598–41,623) | 33,452 (30,939–35,965) | -15.1 (-24.1–-5.1) | -13.4 (-22.3–-3.5) | -26.4 (-34.0–-17.9) |
| South-East Asia | 93,097 (79,515–106,680) | 132,711 (113,352–152,070) | 159,832 (135,422–184,242) | 42.6 (15.8–75.2) | 20.4 (-3.0–48.4) | 71.7 (37.8–112.1) |
| Western Pacific | 36,125 (31,376–40,873) | 54,036 (46,192–61,879) | 65,337 (54,898–75,776) | 49.6 (22.0–81.5) | 20.9 (-2.9–49.7) | 80.9 (46.8–122.1) |

| **Region** | **Males** | | | | | | **Females** | | | | | |
| --- | --- | --- | --- | --- | --- | --- | --- | --- | --- | --- | --- | --- |
|  | **Deaths (UR)** | | | **Percent change (UR)** | | | **Deaths (UR)** | | | **Percent change (UR)** | | |
|  | **2000** | **2010** | **2016** | **2000–2010** | **2010–2016** | **2000–2016** | **2000** | **2010** | **2016** | **2000–2010** | **2010–2016** | **2000–2016** |
| Africa | 8,484 (7,855–9,112) | 9,922 (9,195–10,649) | 11,651 (10,731–12,572) | 17.0 (5.5–30.0) | 17.4 (5.2–30.9) | 37.3 (22.8–53.1) | 4,188  (3,816–  4,559) | 4,740  (4,313–  5,167) | 5,290  (4,757–  5,823) | 13.2  (-0.4–28.4) | 11.6  (-2.2–28.0) | 26.3 (10.2–44.3) |
| Americas | 20,198 (18,280–22,116) | 20,170 (18,392–21,947) | 21,670 (19,709–23,632) | -0.1  (-12.1–14.1) | 7.4  (-5.4–22.0) | 7.3  (-6.1–22.6) | 3,180  (2,867–  3,493) | 3,159  (2,883–  3,435) | 3,409  (3,111–  3,706) | -0.7  (-13.1–13.3) | 7.9  (-4.8–22.2) | 7.2  (-5.8–22.2) |
| Eastern Mediterranean | 27,160 (24,371–29,949) | 33,012 (29,506–36,519) | 37,302 (33,205–41,400) | 21.6 (4.8–40.3) | 13.0  (-3.5–31.9) | 37.3 (18.0–60.0) | 7,100  (6,315–  7,886) | 7,985  (7,064–  8,906) | 8,809  (7,740–  9,878) | 12.5  (-4.4–31.8) | 10.3  (-6.7–30.3) | 24.1 (5.1–45.2) |
| Europe | 37,520 (34,079–40,961) | 31,449 (28,557–34,342) | 27,217 (24,801–29,634) | -16.2  (-26.3–  -4.3) | -13.5  (-23.7–  -1.5) | -27.5  (-36.1–  -17.5) | 7,932  (7,078–  8,786) | 7,161  (6,319–  8,003) | 6,235  (5,546–  6,924) | -9.7  (-23.0–6.0) | -12.9  (-25.9–2.5) | -21.4  (-32.5–  -8.6) |
| South-East Asia | 69,006 (56,201–81,810) | 99,620 (81,301–117,939) | 122,516 (99,242–145,791) | 44.4 (11.0–88.3) | 23.0  (-5.6–60.3) | 77.6 (35.1–132.8) | 24,092  (19,561–  28,622) | 33,091  (26,831–  39,352) | 37,316 (29,958–44,674) | 37.4 (5.5–80.3) | 12.8  (-14.4–48.6) | 54.9 (17.2–103.7) |
| Western Pacific | 24,424 (20,224–28,625) | 35,347 (28,596–42,097) | 42,396 (33,417–51,376) | 44.7 (11.2–86.9) | 20.0  (-10.7–59.1) | 73.6 (31.0–126.7) | 11,700  (9,487–  13,914) | 18,689  (14,694–  22,684) | 22,940 (17,617–28,264) | 59.7 (19.5–111.8) | 22.8  (-11.8–67.4) | 96.1 (41.9–162.6) |

Footnote: UR = 95% uncertainty range

**Table S9: Population-attributable fractions for stroke attributable to exposure to long working hours (≥55 hours/week), by country and sex, 2000, 2010 and 2016, 183 countries**

|  | **Region** | **Both sexes** | | | | | |
| --- | --- | --- | --- | --- | --- | --- | --- |
|  |  | **PAF (%) (UR)** | | | **Percent change (UR)** | | |
|  |  | **2000** | **2010** | **2016** | **2000–2010** | **2010–2016** | **2000–2016** |
| Deaths | Africa | 6.2 (5.9– 6.5) | 6.3 (5.9– 6.6) | 6.3 (5.9– 6.7) | 0.9 (-6.5–9.0) | 0.6 (-7.4–8.9) | 1.5 (-6.4–10.2) |
|  | Americas | 4.5 (4.2– 4.8) | 4.4 (4.1– 4.7) | 4.2 (3.9– 4.5) | -2.7 (-11.7–7.1) | -3.6 (-12.0–5.7) | -6.2 (-14.7–3.1) |
|  | Eastern Mediterranean | 10.4 (9.5–11.4) | 9.8 (8.9–10.6) | 9.5 (8.6–10.4) | -6.2 (-17.1–6.1) | -2.3 (-14.1–11.1) | -8.4 (-19.7–4.5) |
|  | Europe | 2.8 (2.6– 3.0) | 2.7 (2.5– 2.9) | 2.5 (2.3– 2.6) | -5.2 (-14.7–5.3) | -7.5 (-16.1–1.9) | -12.4 (-20.9–-2.5) |
|  | South-East Asia | 12.6 (11.1–14.1) | 12.7 (11.3–14.1) | 12.8 (11.3–14.3) | 0.7 (-14.7–18.9) | 0.6 (-14.5–18.3) | 1.3 (-14.9–20.4) |
|  | Western Pacific | 6.1 (5.2– 7.2) | 6.0 (5.0– 6.9) | 6.0 (5.0– 7.1) | -3.1 (-23.3–22.3) | 0.7 (-20.8–27.0) | -2.4 (-24.0–24.1) |
| DALYs | Africa | 6.8 (6.5–7.1) | 7.0 (6.7–7.2) | 7.1 (6.8–7.3) | 2.6 (-2.7–8.1) | 1.3 (-4.0–6.9) | 4.0 (-1.5–9.8) |
|  | Americas | 6.2 (5.8–6.6) | 5.9 (5.6–6.3) | 5.8 (5.4–6.1) | -3.9 (-12.0–4.8) | -2.9 (-10.6–5.5) | -6.7 (-14.3–1.9) |
|  | Eastern Mediterranean | 11.7 (10.9–12.5) | 11.5 (10.7–12.2) | 11.5 (10.7–12.2) | -2.0 (-10.5–7.4) | 0.2 (-8.7–9.8) | -1.8 (-10.5–8.0) |
|  | Europe | 4.1 (3.8–4.4) | 4.0 (3.7–4.2) | 3.8 (3.6–4.1) | -2.6 (-11.8–7.7) | -3.8 (-12.2–5.6) | -6.3 (-14.9–3.3) |
|  | South-East Asia | 14.4 (13.1–15.7) | 14.7 (13.5–16.0) | 14.9 (13.6–16.2) | 2.1 (-10.2–15.9) | 1.2 (-10.5–14.6) | 3.3 (-9.2–17.4) |
|  | Western Pacific | 8.6 (7.5–9.6) | 8.5 (7.4–9.5) | 8.6 (7.4–9.8) | -1.1 (-16.9–17.9) | 1.3 (-16.5–22.0) | 0.1 (-17.1–20.6) |

|  | **Region** | **Males** | | | | | | **Females** | | | | | |
| --- | --- | --- | --- | --- | --- | --- | --- | --- | --- | --- | --- | --- | --- |
|  |  | **PAF (%) (UR)** | | | **Percent change (UR)** | | | **PAF (%) (UR)** | | | **Percent change (UR)** | | |
|  |  | **2000** | **2010** | **2016** | **2000–2010** | **2010–2016** | **2000–2016** | **2000** | **2010** | **2016** | **2000–2010** | **2010–2016** | **2000–2016** |
| Deaths | Africa | 8.6  (8.0– 9.3) | 8.8 (8.2– 9.5) | 9.0 (8.3– 9.7) | 2.6  (-7.7–13.9) | 1.4  (-9.4–13.1) | 4.0  (-7.1–16.1) | 4.4  (4.0–  4.7) | 4.3  (4.0–  4.7) | 4.2 (3.9–4.6) | -1.3  (-12.0–10.6) | -1.7  (-13.2–10.9) | -3.0  (-14.4–9.6) |
|  | Americas | 8.0  (7.3– 8.6) | 7.5 (6.9– 8.1) | 7.2 (6.6– 7.7) | -5.2  (-15.4–6.4) | -5.0  (-14.8–6.3) | -9.9  (-19.7–1.3) | 1.6  (1.5–  1.8) | 1.6  (1.5–  1.8) | 1.6 (1.5–1.7) | -0.1  (-11.5–12.5) | -2.6  (-13.1–9.2) | -2.7  (-13.6–9.5) |
|  | Eastern Mediterranean | 15.1 (13.4–16.9) | 14.5 (12.9–16.2) | 14.2 (12.6–15.9) | -4.4  (-18.4–12.7) | -1.8  (-16.9–15.0) | -6.1  (-20.6–11.1) | 5.7  (5.0–  6.3) | 5.1  (4.5–  5.8) | 5.0 (4.3–5.6) | -9.1  (-23.6–7.5) | -3.5  (-19.7–16.0) | -12.3  (-26.8–4.5) |
|  | Europe | 5.2  (4.7– 5.8) | 4.9 (4.5– 5.3) | 4.5 (4.1– 4.9) | -7.1  (-19.0–6.4) | -7.9  (-18.5–3.8) | -14.5  (-25.0–  -2.2) | 1.2  (1.1–  1.3) | 1.1  (1.0–  1.2) | 1.0 (0.9–1.1) | -8.0  (-20.3–6.3) | -9.2  (-20.3–3.7) | -16.5  (-27.2–  -3.4) |
|  | South-East Asia | 17.0 (14.4–19.8) | 17.1 (14.7–19.6) | 17.1 (14.5–19.8) | 0.5  (-18.7–24.4) | -0.2  (-19.5–23.2) | 0.3  (-19.8–25.7) | 7.9  (6.5–  9.3) | 7.7  (6.4–  9.1) | 7.6 (6.4–9.0) | -2.1  (-24.2–26.3) | -1.0  (-23.0–25.8) | -3.0  (-24.0–24.5) |
|  | Western Pacific | 7.6  (6.0– 9.4) | 7.2 (5.7– 8.8) | 7.2 (5.6– 9.0) | -5.4  (-31.2–30.5) | -0.6  (-29.2–38.6) | -6.0  (-33.0–30.6) | 4.5  (3.6–  5.6) | 4.6  (3.6–  5.6) | 4.7 (3.6–5.8) | 0.3  (-27.5–37.6) | 2.1  (-27.5–41.6) | 2.4  (-26.9–42.6) |
| DALYs | Africa | 9.3  (8.8–  9.7) | 9.6 (9.1–10.0) | 9.8 (9.3–10.2) | 3.3  (-3.7–10.9) | 2.0  (-5.0–9.3) | 5.4  (-1.9–13.2) | 4.7 (4.5–5.0) | 4.8  (4.5–5.1) | 4.8 (4.5–5.0) | 1.4  (-6.1–  9.3) | -0.5  (-8.3–7.9) | 0.9  (-7.0–9.5) |
|  | Americas | 9.9  (9.1–10.7) | 9.4 (8.8–10.1) | 9.1 (8.4–9.7) | -4.8  (-14.4–6.2) | -4.0  (-13.2–6.3) | -8.6  (-17.7–1.7) | 2.5 (2.3–2.7) | 2.4  (2.3–2.6) | 2.4 (2.2–2.5) | -3.2  (-13.0–7.8) | -2.7  (-12.0–7.5) | -5.9  (-15.0–4.1) |
|  | Eastern Mediterranean | 16.3 (14.9–17.6) | 16.2 (14.9–17.6) | 16.3 (14.9–17.7) | -0.5  (-11.8–12.0) | 0.6  (-10.7–13.6) | 0.1  (-11.0–12.9) | 6.9 (6.3–7.6) | 6.6 (6.0–7.2) | 6.6 (6.0–7.2) | -4.6  (-16.4–8.6) | -0.8  (-12.8–13.3) | -5.4  (-16.9–7.6) |
|  | Europe | 6.7  (6.0–  7.3) | 6.4 (5.8–6.9) | 6.1 (5.6–6.5) | -4.5  (-15.4–8.4) | -4.5  (-15.0–7.2) | -8.8  (-19.3–3.1) | 1.9 (1.7–2.1) | 1.8 (1.6–1.9) | 1.7 (1.6–1.9) | -4.0  (-15.9–9.7) | -3.3  (-14.3–8.9) | -7.2  (-18.4–6.1) |
|  | South-East Asia | 18.1 (16.0–20.3) | 18.4 (16.3–20.4) | 18.4 (16.4–20.5) | 1.3  (-13.9–19.2) | 0.5  (-14.5–18.2) | 1.8  (-13.8–19.2) | 10.1 (8.7–11.5) | 10.2 (8.8–11.5) | 10.2 (8.9–11.5) | 0.9  (-17.0–23.0) | 0.1  (-17.1–19.9) | 1.1  (-16.7–21.5) |
|  | Western Pacific | 10.4 (8.6–12.2) | 10.1 (8.4–11.8) | 10.1 (8.3–12.0) | -2.4  (-23.5–24.3) | 0.3  (-22.2–29.0) | -2.0  (-24.4–26.3) | 6.5 (5.3–7.7) | 6.6 (5.3–7.8) | 6.7 (5.4–8.1) | 1.0  (-21.8–31.0) | 2.6  (-21.9–34.9) | 3.6  (-20.8–35.0) |

Footnote: UR = 95% uncertainty range

**Table S10: Number of deaths from stroke attributable to exposure to long working hours (≥55 hours/week), 2000, 2010, and 2016, and mean percentage change for 2000–2010, 2010–2016, and 2000–2016, by country and sex, 183 countries**

| **Region** | **Both sexes** | | | | | |
| --- | --- | --- | --- | --- | --- | --- |
|  | **Deaths (UR)** | | | **Percent change (UR)** | | |
|  | **2000** | **2010** | **2016** | **2000–2010** | **2010–2016** | **2000–2016** |
| Africa | 17,906 (17,042–18,771) | 20,331 (19,368–21,294) | 23,003 (21,822–24,183) | 13.5 (6.0–21.6) | 13.1 (5.4–21.2) | 28.5 (19.5–37.9) |
| Americas | 19,591 (18,279–20,902) | 17,833 (16,702–18,965) | 18,285 (17,162–19,409) | -9.0 (-16.8–0.1) | 2.5 (-6.2–12.3) | -6.7 (-14.8–2.1) |
| Eastern Mediterranean | 25,484 (23,393–27,576) | 28,593 (26,239–30,947) | 30,807 (28,188–33,426) | 12.2 (-0.3–26.1) | 7.7 (-4.5–21.5) | 20.9 (7.3–36.1) |
| Europe | 37,264 (34,397–40,132) | 29,469 (27,455–31,483) | 24,218 (22,611–25,825) | -20.9 (-28.7–-12.3) | -17.8 (-25.2–-9.6) | -35.0 (-41.3–-28.0) |
| South-East Asia | 119,868 (106,664–133,072) | 143,542 (128,725–158,359) | 158,987 (141,968–176,006) | 19.8 (3.1–39.5) | 10.8 (-4.4–28.3) | 32.6 (14.3–54.7) |
| Western Pacific | 114,741 (97,365–132,116) | 126,917 (108,361–145,472) | 143,140 (120,401–165,879) | 10.6 (-10.8–36.5) | 12.8 (-9.7–39.9) | 24.8 (-0.4–55.8) |

| **Region** | **Males** | | | | | | **Females** | | | | | |
| --- | --- | --- | --- | --- | --- | --- | --- | --- | --- | --- | --- | --- |
|  | **Deaths (UR)** | | | **Percent change (UR)** | | | **Deaths (UR)** | | | **Percent change (UR)** | | |
|  | **2000** | **2010** | **2016** | **2000–2010** | **2010–**  **2016** | **2000–2016** | **2000** | **2010** | **2016** | **2000–2010** | **2010–**  **2016** | **2000–2016** |
| Africa | 10,723 (10,034–11,412) | 12,324 (11,547–13,101) | 14,224 (13,266–15,182) | 14.9 (5.0–25.7) | 15.4 (5.3–26.6) | 32.7 (20.8–45.5) | 7,184 (6,662–7,705) | 8,007 (7,438–8,576) | 8,779 (8,089–9,468) | 11.5 (0.8–23.3) | 9.6  (-1.4–21.7) | 22.2 (9.8–35.9) |
| Americas | 15,651 (14,380–16,922) | 14,227 (13,130–15,324) | 14,599 (13,511–15,687) | -9.1  (-18.5–  1.5) | 2.6  (-7.6–14.0) | -6.7  (-16.4–4.2) | 3,939 (3,616–4,263) | 3,606 (3,328–3,885) | 3,687 (3,406–3,967) | -8.5  (-18.0–2.3) | 2.2  (-8.4–13.9) | -6.4  (-16.3–4.6) |
| Eastern Mediterranean | 18,566 (16,613–20,519) | 20,973 (18,799–23,148) | 22,685 (20,266–25,105) | 13.0  (-3.0–  31.0) | 8.2  (-6.9–25.7) | 22.2 (5.2–42.0) | 6,919 (6,170–7,668) | 7,620 (6,718–8,521) | 8,122 (7,120–9,123) | 10.1  (-6.4–29.1) | 6.6  (-10.0–26.6) | 17.4  (-0.9–38.3) |
| Europe | 27,511 (24,831–30,192) | 22,184 (20,293–24,076) | 18,389 (16,867–19,911) | -19.4  (-29.0–  -8.2) | -17.1  (-26.6–  -6.6) | -33.2  (-41.1–  -23.7) | 9,753 (8,736–10,770) | 7,285 (6,593–7,976) | 5,829 (5,314–6,344) | -25.3  (-35.2–  -13.8) | -20.0  (-29.8–  -8.8) | -40.2  (-47.9–  -31.3) |
| South-East Asia | 83,516 (71,752–95,281) | 102,309 (88,972–115,646) | 115,341 (99,690–130,993) | 22.5 (1.0–48.4) | 12.7  (-6.8–36.5) | 38.1 (13.6–67.8) | 36,352 (30,356–42,347) | 41,233 (34,777–47,689) | 43,646 (36,961–50,331) | 13.4  (-9.4–43.9) | 5.9  (-15.2–31.6) | 20.1  (-4.3–51.8) |
| Western Pacific | 73,628 (58,484–88,773) | 80,505 (64,531–96,479) | 90,859 (71,210–110,508) | 9.3  (-18.1–45.6) | 12.9  (-16.9–51.3) | 23.4  (-9.9–66.6) | 41,112 (32,595–49,629) | 46,411 (36,970–55,853) | 52,281 (40,837–63,725) | 12.9  (-15.4–50.9) | 12.7  (-17.1–51.0) | 27.2  (-6.8–74.4) |

Footnote: UR = 95% uncertainty range

**Table S11: Results from sensitivity analyses for deaths and DALYs (and 95% uncertainty ranges), 2016, all countries**

| **Sensitivity analysis** | **Ischemic heart disease** | | **Stroke** | |
| --- | --- | --- | --- | --- |
|  | **Deaths (UR)** | **DALYs (in ‘000) (UR)** | **Deaths (UR)** | **DALYs (in ‘000) (UR)** |
| Main analysis | 346,753 (319,658–373,848) | 10,655 (9,874–11,437) | 398,441 (369,826–427,056) | 12,603 (11,817–13,390) |
| Reduced lag time to 8 years (2003–2012) | 324,346 (298,508–350,185) | 10,216 (9,461–10,971) | 368,317 (342,619–394,015) | 11,947 (11,218–12,676) |
| Increased lag time to 12 years (1999–2008) | 376,814 (347,892–405,735) | 11,182 (10,372–11,992) | 439,169 (406,739–471,600) | 13,428 (12,574–14,282) |
| Reduced time window of exposure to 8 years (2002–2009) | 307,329 (282,867–331,792) | 9,511 (8,801–10,221) | 351,911 (326,800–377,022) | 11,208 (10,507–11,909) |
| Increased time window of exposure to 12 years (2000–2011) | 386,339 (356,810–415,868) | 11,713 (10,884–12,542) | 446,171 (414,058–478,284) | 13,932 (13,067–14,796) |
| Assigned most common exposure category over time window (restricted to years in labour market activity) | 179,360 (166,755–191,966) | 5,006 (4,702–5,311) | 219,517 (203,999–235,034) | 6,470 (6,064–6,876) |

Footnote: UR = 95% uncertainty range

**Table S12. GATHER checklist**


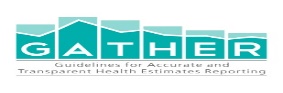
**Checklist of information that should be included in new reports of global health estimates**

| Item # | Checklist item | Reported on page # |  |
| --- | --- | --- | --- |
| Objectives and funding | | | |
| 1 | Define the indicator(s), populations (including age, sex, and geographic entities), and time period(s) for which estimates were made. | Abstract - 3 |  |
| 2 | List the funding sources for the work. | Abstract - 3 |  |
| Data Inputs | | | |
| *For all data inputs from multiple sources that are synthesized as part of the study:* | | | |
| 3 | Describe how the data were identified and how the data were accessed. | 8 and Supplementary Tables S3-S8 |  |
| 4 | Specify the inclusion and exclusion criteria. Identify all ad-hoc exclusions. |  |  |
| 5 | Provide information on all included data sources and their main characteristics. For each data source used, report reference information or contact name/institution, population represented, data collection method, year(s) of data collection, sex and age range, diagnostic criteria or measurement method, and sample size, as relevant. | 8 and Supplementary Tables S3-S8 |  |
| 6 | Identify and describe any categories of input data that have potentially important biases (e.g., based on characteristics listed in item 5). | 10 |  |
| *For data inputs that contribute, the analysis but were not synthesized as part of the study:* | | | |
| 7 | Describe and give sources for any other data inputs. | Table S3 (Input data 3-5) |  |
| *For all data inputs:* | | | |
| 8 | Provide all data inputs in a file format from which data can be efficiently extracted (e.g., a spreadsheet rather than a PDF), including all relevant meta-data listed in item 5. For any data inputs that cannot be shared because of ethical or legal reasons, such as third-party ownership, provide a contact name or the name of the institution that retains the right, the data. | www.who.int/groups/who-ilo-joint-estimates |  |
| Data analysis | | | |
| 9 | Provide a conceptual overview of the data analysis method. A diagram may be helpful. | 7-10, Supplementary Methods |  |
| 10 | Provide a detailed description of all steps of the analysis, including mathematical formulae. This description should cover, as relevant, data cleaning, data pre-processing, data adjustments and weighting of data sources, and mathematical or statistical model(s). | 8-9, Supplementary Methods |  |
| 11 | Describe how candidate models were evaluated and how the final model(s) were selected. | 8-9, Supplementary Methods |  |
| 12 | Provide the results of an evaluation of model performance, if done, as well as the results of any relevant sensitivity analysis. | 10, Table S13 |  |
| 13 | Describe methods for calculating uncertainty of the estimates. State which sources of uncertainty were, and were not, accounted for in the uncertainty analysis. | 9-10, Supplementary Methods |  |
| 14 | State how analytic or statistical source code used to generate estimates can be accessed. | Supplementary Methods |  |
| Results and Discussion | | | |
| 15 | Provide published estimates in a file format from which data can be efficiently extracted. | www.who.int/groups/who-ilo-joint-estimates |  |
| 16 | Report a quantitative measure of the uncertainty of the estimates (e.g. uncertainty intervals). | 10-13 |  |
| 17 | Interpret results in light of existing evidence. If updating a previous set of estimates, describe the reasons for changes in estimates. | 14 |  |
| 18 | Discuss limitations of the estimates. Include a discussion of any modelling assumptions or data limitations that affect interpretation of the estimates. | 14-15, Supplementary Methods |  |
