# Supplementary material for: Global, regional, and national burdens of ischemic heart disease and stroke attributable to exposure to long working hours for 194 countries, 2000–2016: A systematic analysis from the WHO/ILO Joint Estimates of the Work-related Burden of Disease and Injury
Source: Environ Int. 2021 Sep;154:106595. doi: 10.1016/j.envint.2021.106595 (PMC8204267; doi:10.1016/j.envint.2021.106595)
Supplement: Supplementary data file 2 [file mmc2.docx]

**Global, regional and national burdens of ischaemic heart disease and stroke attributable exposure to long working hours for 194 countries, 2000-2016: a systematic analysis from the WHO/ILO Joint Estimates of the Work-related Burden of Disease and Injury**

**Supplementary data file 2 –** Script

March 29, 2021

**Models & Functions**

######################################
#
# multilevel model
#
#####################################

whoilo_fit_hours <- function(df){

 my_fit_hours <- function(df_tmp3, hours_band){
 df_tmp <- df_tmp3 %>%
 filter(hours_band == !!hours_band)
 lmerfit_1 <- lme4::lmer(prop ~ y * poly(a,5) + ((y * poly(a,5)) | country_abbrev),
 data = df_tmp,
 weights = wgt,
 control = lme4::lmerControl(check.conv.singular = lme4::.makeCC(action = "ignore", tol = 1e-3)))
 preds <- merTools::predictInterval(lmerfit_1,
 newdata = df_tmp,
 which = "full",
 n.sims = 1000,
 include.resid.var = FALSE,
 level=0.95,
 stat="median")
 tmp_dta <- bind_cols(df_tmp, preds) %>%
 mutate(fit = case_when(fit < 0 ~ 0,
 fit > 1 ~ 1,
 TRUE ~ fit),
 lwr = case_when(lwr < 0 ~ 0,
 lwr > 1 ~ 1,
 TRUE ~ lwr),
 upr = case_when(upr < 0 ~ 0,
 upr > 1 ~ 1,
 TRUE ~ upr))
 return(tmp_dta)
 }
 out <- NULL
 for (region in unique(who_ilo_long_hours_fit$region)){
 for (sex in unique(who_ilo_long_hours_fit$sex)) {
 df_tmp2 <- df %>%
 filter(region == !!region,
 sex == !!sex)
 tmp <- foreach(i=c(1:5)) %dopar% {
 my_fit_hours(df = df_tmp2,
 hours_band = i)}
 tmp <- data.table::rbindlist(tmp)
 out <- bind_rows(out, tmp)
 }
 }
 out <- out %>%
 select(region, country_abbrev, year, survey, sex, ilo_age_bands_5yr, hours_band, starts_with("prop"), fit, upr, lwr) %>%
 pivot_wider(names_from = hours_band, values_from = c("prop", "prop_lwr", "prop_upr", "fit", "lwr", "upr")) %>%
 mutate(sd_1 = (upr_1 - lwr_1) / 2 / 1.96,
 sd_2 = (upr_2 - lwr_2) / 2 / 1.96,
 sd_3 = (upr_3 - lwr_3) / 2 / 1.96,
 sd_4 = (upr_4 - lwr_4) / 2 / 1.96,
 sd_5 = (upr_5 - lwr_5) / 2 / 1.96,
 sd_0 = sqrt(sd_1^2 + sd_2^2 + sd_3^2 + sd_4^2 + sd_5^2),
 fit_0 = 1 - fit_1 - fit_2 - fit_3 - fit_4 - fit_5) %>%
 rowwise() %>%
 mutate(lwr_0 = qnorm(0.025, fit_0, sd_0),
 upr_0 = qnorm(0.975, fit_0, sd_0)) %>%
 ungroup() %>%
 select(region, country_abbrev, year, sex, ilo_age_bands_5yr, starts_with("fit"), starts_with("lwr"), starts_with("upr")) %>%
 distinct() %>%
 pivot_longer(cols = fit_1:upr_0) %>%
 separate(name, into = c("name", "hours_band")) %>%
 pivot_wider() %>%
 rename(fit_lwr = lwr, fit_upr = upr) %>%
 arrange(region, country_abbrev, year, sex, ilo_age_bands_5yr, hours_band) %>%
 ungroup() %>%
 distinct()
 return(out)
}

#####################################
#
# microsimulation
#
#####################################

MC_calculate_exposure_country_ci <- function(df_fit,
 df_turnover,
 country_abbrev,
 year_now,
 lag_time,
 exposure_window,
 df_population,
 total_population,
 df_death,
 full,
 metric,
 n){
 out <- NULL
 sexes <- c("m", "f")
 ages <- c("15_19", "20_24", "25_29", "30_34", "35_39", "40_44", "45_49", "50_54", "55_59", "60_64", "65_69", "70_74", "75_79", "80_84", "85_89", "90_94", "95_")
 start_year <- year_now - lag_time - exposure_window - 1
 end_year <- year_now + 1
 for (sex in sexes){
 tmp_fit <- df_fit %>%
 filter(country_abbrev == !!country_abbrev,
 sex == sex,
 year >= start_year,
 year <= end_year) %>%
 unique
 tmp_turnover <- df_turnover %>%
 filter(country_abbrev == !!country_abbrev,
 sex == sex) %>%
 unique
 tmp_populations <- df_population %>%
 filter(country_abbrev == !!country_abbrev,
 sex == sex,
 year >= start_year,
 year <= end_year) %>%
 unique
 tmp_death <- df_death %>%
 filter(country_abbrev == !!country_abbrev,
 sex == sex,
 year >= start_year,
 year <= end_year)
 for (age in ages){
 tmp_out <- whoilo_MC_calculate_exposure(df_fit = tmp_fit,
 df_turnover = tmp_turnover,
 country_abbrev = country_abbrev,
 sex = sex,
 ilo_age_bands_5yr = age,
 year_now = year_now,
 lag_time = lag_time,
 exposure_window = exposure_window,
 df_population = tmp_populations,
 total_population = total_population,
 df_death = tmp_death,
 full = full,
 metric = metric,
 n = n)
 tmp_out$sex <- sex
 tmp_out$ilo_age_bands_5yr <- age
 tmp_out$country_abbrev <- country_abbrev
 tmp_out$year <- year_now
 out <- bind_rows(out, tmp_out)
 }
 }
 return(out)
}

MC_calculate_exposure_country_ci <- possibly(MC_calculate_exposure_country_ci,
 otherwise = tibble(country_abbrev = NA_character_, sex = NA_character_, ilo_age_bands_5yr = NA_character_, year = NA_real_, h0 = NA_real_, h1 = NA_real_, h2 = NA_real_, h3 = NA_real_, h4 = NA_real_, h5 = NA_real_),
 quiet = TRUE)

whoilo_MC_calculate_exposure <- function(df_fit,
 df_turnover,
 country_abbrev,
 sex,
 ilo_age_bands_5yr,
 year_now,
 lag_time,
 exposure_window,
 df_population,
 total_population,
 df_death,
 full = FALSE,
 metric,
 n){
 if (n == 0) {
 MC_calculate_exposure_pe(df_fit = df_fit,
 df_turnover = df_turnover,
 country_abbrev = country_abbrev,
 sex = sex,
 ilo_age_bands_5yr = ilo_age_bands_5yr,
 year_now = year_now,
 lag_time = lag_time,
 exposure_window = exposure_window,
 df_population = df_population,
 total_population = total_population,
 df_death = df_death,
 full = full,
 metric = metric)
 } else if (is.numeric(n)) {
 out <- foreach::foreach(i = c(1:n),
 .packages = c('tidyverse')) %dopar% {
 MC_calculate_exposure_ci(df_fit = df_fit,
 df_turnover = df_turnover,
 country_abbrev = country_abbrev,
 sex = sex,
 ilo_age_bands_5yr = ilo_age_bands_5yr,
 year_now = year_now,
 lag_time = lag_time,
 exposure_window = exposure_window,
 df_population = df_population,
 total_population = total_population,
 df_death = df_death,
 full = full,
 metric = metric)
 }

 out = data.table::rbindlist(out)
 saveRDS(out, str_c("export/model3_exposure_boot/",year_now,"/dist_", country_abbrev,"_",sex,"_",ilo_age_bands_5yr,".RData"))

 out = out %>%
 group_by(country_abbrev, sex, ilo_age_bands_5yr, year) %>%
 summarise(h0_median = quantile(h0, probs = c(0.5), na.rm = TRUE),
 h0_quantile_lwr = quantile(h0, probs = c(0.025), na.rm = TRUE),
 h0_quantile_upr = quantile(h0, probs = c(0.975), na.rm = TRUE),
 h0_mean = Rmisc::CI(h0)[[2]],
 h0_ci_lwr = Rmisc::CI(h0)[[3]],
 h0_ci_upr = Rmisc::CI(h0)[[1]],
 h1_median = quantile(h1, probs = c(0.5), na.rm = TRUE),
 h1_quantile_lwr = quantile(h1, probs = c(0.025), na.rm = TRUE),
 h1_quantile_upr = quantile(h1, probs = c(0.975), na.rm = TRUE),
 h1_mean = Rmisc::CI(h1)[[2]],
 h1_ci_lwr = Rmisc::CI(h1)[[3]],
 h1_ci_upr = Rmisc::CI(h1)[[1]],
 h2_median = quantile(h2, probs = c(0.5), na.rm = TRUE),
 h2_quantile_lwr = quantile(h2, probs = c(0.025), na.rm = TRUE),
 h2_quantile_upr = quantile(h2, probs = c(0.975), na.rm = TRUE),
 h2_mean = Rmisc::CI(h2)[[2]],
 h2_ci_lwr = Rmisc::CI(h2)[[3]],
 h2_ci_upr = Rmisc::CI(h2)[[1]],
 h3_median = quantile(h3, probs = c(0.5), na.rm = TRUE),
 h3_quantile_lwr = quantile(h3, probs = c(0.025), na.rm = TRUE),
 h3_quantile_upr = quantile(h3, probs = c(0.975), na.rm = TRUE),
 h3_mean = Rmisc::CI(h3)[[2]],
 h3_ci_lwr = Rmisc::CI(h3)[[3]],
 h3_ci_upr = Rmisc::CI(h3)[[1]],
 h4_median = quantile(h4, probs = c(0.5), na.rm = TRUE),
 h4_quantile_lwr = quantile(h4, probs = c(0.025), na.rm = TRUE),
 h4_quantile_upr = quantile(h4, probs = c(0.975), na.rm = TRUE),
 h4_mean = Rmisc::CI(h4)[[2]],
 h4_ci_lwr = Rmisc::CI(h4)[[3]],
 h4_ci_upr = Rmisc::CI(h4)[[1]],
 h5_median = quantile(h5, probs = c(0.5), na.rm = TRUE),
 h5_quantile_lwr = quantile(h5, probs = c(0.025), na.rm = TRUE),
 h5_quantile_upr = quantile(h5, probs = c(0.975), na.rm = TRUE),
 h5_mean = Rmisc::CI(h5)[[2]],
 h5_ci_lwr = Rmisc::CI(h5)[[3]],
 h5_ci_upr = Rmisc::CI(h5)[[1]]

 ) %>%
 ungroup
 return(out)
 }
}

MC_calculate_exposure_ci <- function(df_fit,
 df_turnover,
 country_abbrev,
 sex,
 ilo_age_bands_5yr,
 year_now,
 lag_time,
 exposure_window,
 df_population,
 total_population,
 df_death,
 full = FALSE,
 metric){
 turnover_df_rnd <- whoilo_randomize_turnover(df_turnover,
 country_abbrev = country_abbrev,
 sex = sex)
 fit_df_rnd <- whoilo_randomize_1parameter(df_fit, "fit")
 death_df_rnd <- whoilo_randomize_1parameter(df_death, "death_rate")
 MC_calculate_exposure_pe(df_fit = fit_df_rnd,
 df_turnover = turnover_df_rnd,
 country_abbrev = country_abbrev,
 sex = sex,
 ilo_age_bands_5yr = ilo_age_bands_5yr,
 year_now = year_now,
 lag_time = lag_time,
 exposure_window = exposure_window,
 df_population = df_population,
 total_population = total_population,
 df_death = death_df_rnd,
 full = full,
 metric = metric)
}

MC_calculate_exposure_pe <- function(df_fit,
 df_turnover,
 country_abbrev,
 sex,
 ilo_age_bands_5yr,
 year_now,
 lag_time,
 exposure_window,
 df_population,
 total_population,
 df_death,
 full = FALSE,
 metric,
 left_side = TRUE){

 pattern <- "[[:digit:]]+$"

 out <- tibble(country_abbrev = !!country_abbrev,
 sex = !!sex,
 ilo_age_bands_5yr = !!ilo_age_bands_5yr,
 year = !!year_now,
 h0 = double(1),
 h1 = double(1),
 h2 = double(1),
 h3 = double(1),
 h4 = double(1),
 h5 = double(1))

 full_out <- list()

 age_now_df <- whoilo_select_cohort(year_now = year_now,
 age_now = 15:100,
 lag_time = lag_time,
 exposure_window = exposure_window) %>%

 filter(!is.na(start_age)) %>%

 filter(start_age < 100) %>%

 mutate(ilo_age_bands_5yr = whoilo_age_to_bands(age = age_now, age_type = "UN"),
 age_bands_5yr = ilo_age_bands_5yr)


 age_now_df <- left_join(age_now_df,
 age_now_df %>%
 group_by(ilo_age_bands_5yr) %>%
 summarise(n = n()) %>%
 ungroup,
 by = "ilo_age_bands_5yr") %>%
 filter(age_bands_5yr == !!ilo_age_bands_5yr)

 if (nrow(age_now_df) == 0) {
 return(out)
 }

 for (i in 1:nrow(age_now_df)) {
 tmp <- whoilo_follow_cohort(year_now = year_now,
 lag_time = lag_time,
 exposure_window = exposure_window,
 age_now = age_now_df$age_now[i])

 tmp <- left_join(tmp, tmp %>%
 group_by(age_bands_5yr) %>%
 summarise(start_year = min(years),
 end_year = max(years)),
 by = "age_bands_5yr")

 years <- tmp$years[1]

 tmp <- tmp %>%
 select(age_bands_5yr, end_year) %>%
 mutate(ilo_age_bands_5yr = age_bands_5yr) %>%
 unique %>%
 mutate(end_year =
 case_when(
 end_year >= year_now - lag_time + exposure_window ~ end_year - 1,
 TRUE ~ as.double(end_year)
 )
 )

 for (j in 1:nrow(tmp)) {

 if (j == 1){

 if (!is.null(df_population)){

 population <- df_population %>%

 filter(country_abbrev == !!country_abbrev,
 year == years,
 sex == !!sex,
 age_bands_5yr == tmp$age_bands_5yr[j]) %>%

 select(population)


 population <- population$population * 200

 if (!is.null(total_population)){

 national_population <- df_population %>%

 filter(country_abbrev == !!country_abbrev,
 year == years,
 sex == !!sex,
 age_bands_5yr == "national") %>%

 select(population)

 national_population <- national_population$population
 }

 population <- population / national_population * total_population /10

 } else {

 try(if (is.null(total_population)) stop("population size is missing"))
 population <- total_population /81

 }

 cohort <- whoilo_start_year(df = df_fit,
 country_abbrev = !!country_abbrev,
 year = years,
 age = tmp$age_bands_5yr[j],
 sex = !!sex,
 population = population)

 }

 turnover_rate <- whoilo_select_turnover_rate(df = df_turnover,
 year = years,
 country_abbrev = !!country_abbrev,
 age = tmp$ilo_age_bands_5yr[j],
 sex = !!sex)


 years <- as.double(str_extract(tail(names(cohort),n=1),pattern))


 if (years < (year_now - lag_time + exposure_window)) {

 for (k in years:tmp$end_year[j]){

 cohort <- whoilo_next_year(cohort, turnover_rate)

 if (!is.null(df_death)) {

 death_rate <- whoilo_select_death_rate(df = df_death,
 year = k,
 country_abbrev = !!country_abbrev,
 age_bands_5yr = tmp$age_bands_5yr[j],
 sex = !!sex)

 cohort <- whoilo_censor_year(df = cohort,
 new_year = FALSE,
 death_rate = death_rate)
 }
 }
 }


 years <- as.double(str_extract(tail(names(cohort), n=1), pattern))


 if (years > (year_now - lag_time + exposure_window)) {

 message(" cleaning cohort ", years)

 years <- (year_now - lag_time + exposure_window)

 cohort <- cohort[,1:ncol(cohort)-1]

 break

 }

 }


 if (!is.null(df_death) & (years < year_now)){

 for (k in years:(year_now-1)){

 death_rate <- whoilo_select_death_rate(df = df_death,
 year = k,
 country_abbrev = !!country_abbrev,
 age_bands_5yr = tmp$age_bands_5yr[j],
 sex = !!sex)


 cohort <- whoilo_censor_year(df = cohort,
 new_year = TRUE,
 death_rate = death_rate)
 }

 }

 if (left_side == TRUE){

 year1 <- paste0("y_", (year_now - lag_time + exposure_window-1))

 if (year1 %in% names(cohort)) {

 n0 <- which(names(cohort) == year1)

 for (cohort_years in c((n0+1):length(names(cohort)))) {

 year2 <- names(cohort[cohort_years])

 cohort[[year2]] <- ifelse(test = is.na(cohort[[year2]]), yes = NA, no = cohort[[year1]])

 }

 } else {

 cohort[cohort !=0 ] <- 0
 cohort[is.na(cohort)] <- 0

 }

 }


 if (full){
 full_out <- append(full_out, list(age_now_df[i,], cohort))
 }

 if (metric == "MAX"){
 ## everyone is exposed as the maximum category visited
 exposure <- matrixStats::rowMaxs(as.matrix(cohort))
 } else if (metric == "MAX2") {
 max2 <- function(x) {
 x <- as.numeric(x)
 if(sum(is.na(x)) > 0) {
 return(NA)
 }
 max <- 0
 for(i in 2:length(x)) {
 if ((x[i] == x[i-1]) & (x[i] > max)) {
 max <- x[i]
 }
 }
 return(max)
 }
 exposure <- apply(cohort, 1, max2)
 } else if (metric == "MAX3") {

 year_range <- (year_now - lag_time - exposure_window):(year_now - lag_time + exposure_window-1)
 bb <- table(row(cohort), as.matrix(cohort), useNA = "always")

 cohort <- cohort %>%

 select(num_range(prefix = "y_", range = year_range))

 cc <- table(row(cohort), as.matrix(cohort), useNA = "always")

 exposure <- double((nrow(bb)-1))

 for (i in 1:(nrow(bb)-1)){

 if (last(bb[i,]) != 0){exposure[i] = NA

 } else {
 exposure[i] = max(which(cc[i,] == max(cc[i,])))-1
 }

 }

 } else if (metric == "MAX4") {
 year_range <- (year_now - lag_time - exposure_window):(year_now - lag_time + exposure_window-1)

 bb <- table(row(cohort), as.matrix(cohort), useNA = "always")

 cohort <- cohort %>%

 select(num_range(prefix = "y_", range = year_range))

 cc <- table(row(cohort), as.matrix(cohort), useNA = "always")

 exposure <- double((nrow(bb)-1))

 if (ncol(cohort) == 0) {
 exposure <-0
 } else {
 for (i in 1:(nrow(bb)-1)){
 if (last(bb[i,]) != 0){
 exposure[i] = NA
 } else if (sum(cc[i,2:7] != 0) == 0) {
 exposure[i] = 0
 } else {exposure[i] = max(which(cc[i,2:7] == max(cc[i,2:7])))}
 }
 }
 } else if (metric[1] == "CUM") {

 year_range <- (year_now - lag_time - exposure_window):(year_now - lag_time + exposure_window-1)
 cohort <- cohort %>%
 select(num_range(prefix = "y_", range = year_range))
 cohort[cohort == 1] <- 0.046
 cohort[cohort == 2] <- 0.162
 cohort[cohort == 3] <- 0.036
 if (ncol(cohort) == 0) {
 exposure <-0
 } else {
 exposure <- rowSums(cohort)
 }
 } else {
 stop("Unknown metric")
 }


 if (metric[1] == "CUM") {

 tmp_out <- tibble(country_abbrev = !!country_abbrev,
 sex = !!sex,
 ilo_age_bands_5yr = !!ilo_age_bands_5yr,
 year = !!year_now,
 h0 = exposure,
 h1 = double(1),
 h2 = double(1),
 h3 = double(1),
 h4 = double(1),
 h5 = double(1))

 }
 if (metric[1] != "CUM") {
 if (sum(!is.na(exposure)) == 0) {

 aa <- tibble(country_abbrev = !!country_abbrev,
 sex = !!sex,
 ilo_age_bands_5yr = !!ilo_age_bands_5yr,
 year = !!year_now,
 h0 = 0,
 h1 = 0,
 h2 = 0,
 h3 = 0,
 h4 = 0,
 h5 = 0)

 } else {

 aa <- table(exposure)

 aa <- as_tibble(list(x = aa, n = names(aa))) %>%
 mutate(n = str_c("h", n)) %>%
 spread(n ,x)

 aa <- aa %>%
 mutate(h0 = ifelse(test = ("h0" %in% names(aa)),
 yes = h0,
 no = 0),
 h1 = ifelse(test = ("h1" %in% names(aa)),
 yes = h1,
 no = 0),
 h2 = ifelse(test = ("h2" %in% names(aa)),
 yes = h2,
 no = 0),
 h3 = ifelse(test = ("h3" %in% names(aa)),
 yes = h3,
 no = 0),
 h4 = ifelse(test = ("h4" %in% names(aa)),
 yes = h4,
 no = 0),
 h5 = ifelse(test = ("h5" %in% names(aa)),
 yes = h5,
 no = 0))

 }


 out$h0 <- out$h0 + aa$h0
 out$h1 <- out$h1 + aa$h1
 out$h2 <- out$h2 + aa$h2
 out$h3 <- out$h3 + aa$h3
 out$h4 <- out$h4 + aa$h4
 out$h5 <- out$h5 + aa$h5

 }

 }

 #End of Age Band loop

 if (metric[1] != "CUM") {

 out <- out %>%

 mutate(sum = h0+h1+h2+h3+h4+h5,
 h0 = h0/sum,
 h1 = h1/sum,
 h2 = h2/sum,
 h3 = h3/sum,
 h4 = h4/sum,
 h5 = h5/sum) %>%

 select(-sum)
 }
 if (metric[1] == "CUM") {
 out <- bind_rows(out, tmp_out)
 }


 if (full){
 out <- list(out, full_out)
 }
 return(out)

}


whoilo_select_cohort <- function(year_now,
 age_now,
 lag_time,
 exposure_window,
 legal_age = 15){

 if (!is.numeric(year_now)) {warning("year_now is not a number", call. = FALSE)}
 if (!is.numeric(age_now)) {warning("age_now is not a number", call. = FALSE)}
 if (!is.numeric(lag_time)) {warning("lag_time is not a number", call. = FALSE)}
 if (!is.numeric(exposure_window)) {warning("exposure_window is not a number", call. = FALSE)}
 if (!is.numeric(legal_age)) {warning("legal_age is not a number", call. = FALSE)}

 start_year <- (year_now - lag_time - exposure_window)

 end_year <- (year_now - lag_time + exposure_window)
 if (end_year > year_now) {warning("end_year is in the future", call. = FALSE)}

 tibble(age_now = age_now) %>%
 mutate(start_year = case_when(
 age_now - (year_now - end_year) < legal_age ~ NA_real_,
 age_now - (year_now - !!start_year) >= legal_age ~ !!start_year,
 TRUE ~ year_now - (age_now - legal_age)
 )) %>%
 mutate(start_age = case_when(
 age_now - (year_now - end_year) < legal_age ~ NA_real_,
 age_now - (year_now - !!start_year) > legal_age ~ age_now - (year_now - start_year),
 TRUE ~ 15
 ))
}

whoilo_age_to_bands <- function(age,
 age_type = "UN"){
 if (age_type == "ILO"){
 age <- dplyr::case_when(age %in% 15:19 ~ "15_19",
 age %in% 20:24 ~ "20_24",
 age %in% 25:29 ~ "25_29",
 age %in% 30:34 ~ "30_34",
 age %in% 35:39 ~ "35_39",
 age %in% 40:44 ~ "40_44",
 age %in% 45:49 ~ "45_49",
 age %in% 50:54 ~ "50_54",
 age %in% 55:59 ~ "55_59",
 age %in% 60:64 ~ "60_64",
 age %in% 65:200 ~ "65_",
 TRUE ~ NA_character_)
 } else if (age_type == "UN"){
 age <- dplyr::case_when(age %in% 15:19 ~ "15_19",
 age %in% 20:24 ~ "20_24",
 age %in% 25:29 ~ "25_29",
 age %in% 30:34 ~ "30_34",
 age %in% 35:39 ~ "35_39",
 age %in% 40:44 ~ "40_44",
 age %in% 45:49 ~ "45_49",
 age %in% 50:54 ~ "50_54",
 age %in% 55:59 ~ "55_59",
 age %in% 60:64 ~ "60_64",
 age %in% 65:69 ~ "65_69",
 age %in% 70:74 ~ "70_74",
 age %in% 75:79 ~ "75_79",
 age %in% 80:84 ~ "80_84",
 age %in% 85:89 ~ "85_89",
 age %in% 90:94 ~ "90_94",
 age %in% 95:200 ~ "95_",
 TRUE ~ NA_character_)
 } else if (age_type == "UN10"){
 age <- dplyr::case_when(age %in% 15:24 ~ "15_24",
 age %in% 25:34 ~ "25_34",
 age %in% 35:44 ~ "35_44",
 age %in% 45:54 ~ "45_54",
 age %in% 55:64 ~ "55_64",
 age %in% 65:74 ~ "65_74",
 age %in% 75:84 ~ "75_84",
 age %in% 85:94 ~ "85_94",
 age %in% 95:200 ~ "95_",
 TRUE ~ NA_character_)
 } else {stop("Unknow age_type")}

 return(age)
}

whoilo_follow_cohort <- function(year_now,
 age_now,
 lag_time,
 exposure_window){
 year_start <- year_now - lag_time - exposure_window
 year_end <- year_now - lag_time + exposure_window
 years <- year_start:year_end

 age_now <- enquo(age_now)
 year_now <- enquo(year_now)

 tibble(years = !!years) %>%
 mutate(age = !!age_now - (!!year_now - !!years),
 age_bands_5yr = whoilo_age_to_bands(age = age, age_type = "UN")) %>%
 filter(age >=15)
}

whoilo_start_year <- function(df,
 country_abbrev,
 year,
 age,
 sex,
 population = 1000) {


 if ((age %in% c("65_69", "70_74", "75_79", "80_84", "85_89", "90_94", "95_")) & !(age %in% df$ilo_age_bands_5yr)) { age <- "65_"}


 country_abbrev <- enquo(country_abbrev)
 y_ <- str_c("y_", as.character(year))
 year <- enquo(year)
 age <- enquo(age)
 sex <- enquo(sex)


 cohort <- df %>%
 filter(country_abbrev == !!country_abbrev,
 sex == !!sex,
 year == !!year,
 ilo_age_bands_5yr == !!age) %>%
 select(hours_band, fit) %>%
 mutate(fit = round(fit * !!population))

 cohort$fit[1] <- cohort$fit[1]+(population - sum(cohort$fit))

 cohort %>%
 group_by(hours_band) %>%
 expand(temp = 1:fit) %>%
 select(-temp) %>%
 ungroup %>%
 select(!!y_ := hours_band) %>%
 mutate_all(as.numeric)
}

whoilo_select_turnover_rate <-function(df,
 year,
 country_abbrev,
 age,
 sex){

 country_abbrev <- enquo(country_abbrev)
 age <- enquo(age)
 sex <- enquo(sex)
 year <- enquo(year)


 if (has_element(names(df), "year")) {

 df %>%
 filter(country_abbrev == !!country_abbrev,
 sex == !!sex,
 ilo_age_bands_5yr == !!age,
 year == !!year) %>%
 separate(transition, c("from", "to"), sep = "_") %>%
 group_by(from) %>%
 mutate(cum_sum_p = cumsum(prop),
 cum_sum_n = cumsum(n)) %>%
 ungroup %>%
 unite(transition, from, to, sep = "_")
 } else {

 df %>%
 filter(country_abbrev == !!country_abbrev,
 sex == !!sex,
 ilo_age_bands_5yr == !!age) %>%
 separate(transition, c("from", "to"), sep = "_") %>%
 group_by(from) %>%
 mutate(cum_sum_p = cumsum(prop),
 cum_sum_n = cumsum(n)) %>%
 ungroup %>%
 unite(transition, from, to, sep = "_")
 }
}

whoilo_select_death_rate <-function(df,
 year,
 country_abbrev,
 age_bands_5yr,
 sex){


 if ((age_bands_5yr %in% c("85_89", "90_94", "95_")) & !(age_bands_5yr %in% df$age_bands_5yr)) { age_bands_5yr <- "85_"}

 country_abbrev <- enquo(country_abbrev)
 age_bands_5yr <- enquo(age_bands_5yr)
 sex <- enquo(sex)
 year <- enquo(year)

 df <- df %>%
 filter(country_abbrev == !!country_abbrev,
 sex == !!sex,
 age_bands_5yr == !!age_bands_5yr,
 year == !!year) %>%
 select(death_rate)

 df$death_rate
}

whoilo_censor_year <- function(df,
 new_year = TRUE,
 death_rate) {

 if (new_year){

 pattern <- "[[:digit:]]+$"
 last_year <- tail(names(df), n=1)

 new_year <- str_replace(last_year,
 pattern,
 as.character(as.numeric(str_extract(last_year, pattern)) + 1))


 df <- bind_cols(prop = runif(nrow(df)), df)

 names(df)[length(names(df))] <- "last_year"

 df <- df %>%
 mutate(new_year = case_when(
 prop > !!death_rate ~ last_year,
 TRUE ~ NA_real_
 )) %>%
 select(-prop)

 names(df)[(length(names(df))-1)] <- last_year
 names(df)[length(names(df))] <- new_year
 } else{

 pattern <- "[[:digit:]]+$"

 last_year <- tail(names(df), n=1)


 df <- bind_cols(prop = runif(nrow(df)), df)

 names(df)[length(names(df))] <- "last_year"

 df <- df %>%
 mutate(last_year = case_when(
 prop > !!death_rate ~ last_year,
 TRUE ~ NA_real_
 )) %>%
 select(-prop)

 names(df)[length(names(df))] <- last_year
 }

 return(df)
}

whoilo_next_year <- function(df,
 turnover_rate) {

 pattern <- "[[:digit:]]+$"
 last_year <- tail(names(df), n=1)
 new_year <- str_replace(last_year,
 pattern,
 as.character(as.numeric(str_extract(last_year, pattern)) + 1))


 p_0_0 <- filter(turnover_rate, transition == "0_0")$cum_sum_p
 p_0_1 <- filter(turnover_rate, transition == "0_1")$cum_sum_p
 p_0_2 <- filter(turnover_rate, transition == "0_2")$cum_sum_p
 p_0_3 <- filter(turnover_rate, transition == "0_3")$cum_sum_p
 p_0_4 <- filter(turnover_rate, transition == "0_4")$cum_sum_p
 p_0_5 <- filter(turnover_rate, transition == "0_5")$cum_sum_p
 p_1_0 <- filter(turnover_rate, transition == "1_0")$cum_sum_p
 p_1_1 <- filter(turnover_rate, transition == "1_1")$cum_sum_p
 p_1_2 <- filter(turnover_rate, transition == "1_2")$cum_sum_p
 p_1_3 <- filter(turnover_rate, transition == "1_3")$cum_sum_p
 p_1_4 <- filter(turnover_rate, transition == "1_4")$cum_sum_p
 p_1_5 <- filter(turnover_rate, transition == "1_5")$cum_sum_p
 p_2_0 <- filter(turnover_rate, transition == "2_0")$cum_sum_p
 p_2_1 <- filter(turnover_rate, transition == "2_1")$cum_sum_p
 p_2_2 <- filter(turnover_rate, transition == "2_2")$cum_sum_p
 p_2_3 <- filter(turnover_rate, transition == "2_3")$cum_sum_p
 p_2_4 <- filter(turnover_rate, transition == "2_4")$cum_sum_p
 p_2_5 <- filter(turnover_rate, transition == "2_5")$cum_sum_p
 p_3_0 <- filter(turnover_rate, transition == "3_0")$cum_sum_p
 p_3_1 <- filter(turnover_rate, transition == "3_1")$cum_sum_p
 p_3_2 <- filter(turnover_rate, transition == "3_2")$cum_sum_p
 p_3_3 <- filter(turnover_rate, transition == "3_3")$cum_sum_p
 p_3_4 <- filter(turnover_rate, transition == "3_4")$cum_sum_p
 p_3_5 <- filter(turnover_rate, transition == "3_5")$cum_sum_p
 p_4_0 <- filter(turnover_rate, transition == "4_0")$cum_sum_p
 p_4_1 <- filter(turnover_rate, transition == "4_1")$cum_sum_p
 p_4_2 <- filter(turnover_rate, transition == "4_2")$cum_sum_p
 p_4_3 <- filter(turnover_rate, transition == "4_3")$cum_sum_p
 p_4_4 <- filter(turnover_rate, transition == "4_4")$cum_sum_p
 p_4_5 <- filter(turnover_rate, transition == "4_5")$cum_sum_p
 p_5_0 <- filter(turnover_rate, transition == "5_0")$cum_sum_p
 p_5_1 <- filter(turnover_rate, transition == "5_1")$cum_sum_p
 p_5_2 <- filter(turnover_rate, transition == "5_2")$cum_sum_p
 p_5_3 <- filter(turnover_rate, transition == "5_3")$cum_sum_p
 p_5_4 <- filter(turnover_rate, transition == "5_4")$cum_sum_p
 p_5_5 <- filter(turnover_rate, transition == "5_5")$cum_sum_p


 df <- bind_cols(prop = runif(nrow(df)), df)


 names(df)[length(names(df))] <- "last_year"


 df$new_year <- NA
 df$new_year[(df$last_year == 0 & df$prop < p_0_0)] <- 0
 df$new_year[(df$last_year == 0 & df$prop > p_0_0 & df$prop < p_0_1)] <- 1
 df$new_year[(df$last_year == 0 & df$prop > p_0_1 & df$prop < p_0_2)] <- 2
 df$new_year[(df$last_year == 0 & df$prop > p_0_2 & df$prop < p_0_3)] <- 3
 df$new_year[(df$last_year == 0 & df$prop > p_0_3 & df$prop < p_0_4)] <- 4
 df$new_year[(df$last_year == 0 & df$prop > p_0_4 & df$prop < p_0_5)] <- 5

 df$new_year[(df$last_year == 1 & df$prop < p_1_0)] <- 0
 df$new_year[(df$last_year == 1 & df$prop > p_1_0 & df$prop < p_1_1)] <- 1
 df$new_year[(df$last_year == 1 & df$prop > p_1_1 & df$prop < p_1_2)] <- 2
 df$new_year[(df$last_year == 1 & df$prop > p_1_2 & df$prop < p_1_3)] <- 3
 df$new_year[(df$last_year == 1 & df$prop > p_1_3 & df$prop < p_1_4)] <- 4
 df$new_year[(df$last_year == 1 & df$prop > p_1_4 & df$prop < p_1_5)] <- 5

 df$new_year[(df$last_year == 2 & df$prop < p_2_0)] <- 0
 df$new_year[(df$last_year == 2 & df$prop > p_2_0 & df$prop < p_2_1)] <- 1
 df$new_year[(df$last_year == 2 & df$prop > p_2_1 & df$prop < p_2_2)] <- 2
 df$new_year[(df$last_year == 2 & df$prop > p_2_2 & df$prop < p_2_3)] <- 3
 df$new_year[(df$last_year == 2 & df$prop > p_2_3 & df$prop < p_2_4)] <- 4
 df$new_year[(df$last_year == 2 & df$prop > p_2_4 & df$prop < p_2_5)] <- 5

 df$new_year[(df$last_year == 3 & df$prop < p_3_0)] <- 0
 df$new_year[(df$last_year == 3 & df$prop > p_3_0 & df$prop < p_3_1)] <- 1
 df$new_year[(df$last_year == 3 & df$prop > p_3_1 & df$prop < p_3_2)] <- 2
 df$new_year[(df$last_year == 3 & df$prop > p_3_2 & df$prop < p_3_3)] <- 3
 df$new_year[(df$last_year == 3 & df$prop > p_3_3 & df$prop < p_3_4)] <- 4
 df$new_year[(df$last_year == 3 & df$prop > p_3_4 & df$prop < p_3_5)] <- 5

 df$new_year[(df$last_year == 4 & df$prop < p_4_0)] <- 0
 df$new_year[(df$last_year == 4 & df$prop > p_4_0 & df$prop < p_4_1)] <- 1
 df$new_year[(df$last_year == 4 & df$prop > p_4_1 & df$prop < p_4_2)] <- 2
 df$new_year[(df$last_year == 4 & df$prop > p_4_2 & df$prop < p_4_3)] <- 3
 df$new_year[(df$last_year == 4 & df$prop > p_4_3 & df$prop < p_4_4)] <- 4
 df$new_year[(df$last_year == 4 & df$prop > p_4_4 & df$prop < p_4_5)] <- 5

 df$new_year[(df$last_year == 5 & df$prop < p_5_0)] <- 0
 df$new_year[(df$last_year == 5 & df$prop > p_5_0 & df$prop < p_5_1)] <- 1
 df$new_year[(df$last_year == 5 & df$prop > p_5_1 & df$prop < p_5_2)] <- 2
 df$new_year[(df$last_year == 5 & df$prop > p_5_2 & df$prop < p_5_3)] <- 3
 df$new_year[(df$last_year == 5 & df$prop > p_5_3 & df$prop < p_5_4)] <- 4
 df$new_year[(df$last_year == 5 & df$prop > p_5_4 & df$prop < p_5_5)] <- 5

 df <- select(df, -prop)

 names(df)[(length(names(df))-1)] <- last_year
 names(df)[length(names(df))] <- new_year

 return(df)
}

whoilo_randomize_turnover <- function(df,
 country_abbrev,
 sex){

 country_abbrev <- enquo(country_abbrev)
 sex <- enquo(sex)

 apply_rnorm <- function(x, c1, c2, c3) rnorm(n = 1,
 mean = x[c1],
 sd = 0.25 * (x[c2] -x[c3]))

 df <- df %>%
 filter(country_abbrev == !!country_abbrev,
 sex == !!sex)

 df$prop <- apply(df[,c("prop", "prop_lwr", "prop_upr")],
 1,
 apply_rnorm,
 c1 = "prop",
 c2 = "prop_upr",
 c3 = "prop_lwr")

 if (has_element(names(df), "year")) {
 df <- df %>%
 separate(transition, c("from", "to"), "_") %>%
 group_by(region, country_abbrev, year, sex, ilo_age_bands_5yr, from) %>%
 mutate(prop = ifelse(to == 0, NA, prop),
 pp = sum(prop, na.rm = TRUE),
 prop = ifelse(to == 0, 1-pp, prop)
 ) %>%
 unite(transition, from, to) %>%
 select(-pp) %>%
 mutate(prop = case_when(prop < 0 ~ 0,
 prop > 1 ~ 1,
 TRUE ~ prop)) %>%
 ungroup
 } else{
 df <- df %>%
 separate(transition, c("from", "to"), "_") %>%
 group_by(region, country_abbrev, sex, ilo_age_bands_5yr, from) %>%
 mutate(prop = ifelse(to == 0, NA, prop),
 pp = sum(prop, na.rm = TRUE),
 prop = ifelse(to == 0, 1-pp, prop)
 ) %>%
 unite(transition, from, to) %>%
 select(-pp) %>%
 mutate(prop = case_when(prop < 0 ~ 0,
 prop > 1 ~ 1,
 TRUE ~ prop)) %>%
 ungroup
 }

 return(df)
}

whoilo_randomize_1parameter <- function(df,
 variable,
 constrain = FALSE){

apply_rnorm <- function(x, c1, c2, c3, norm = "r") {

 #Get mean
 m = x[c1]

 #Calculate average distance
 dist = ((x[c2] -x[c3])) / 2

 #Calculate Standard deviation
 se = dist / qnorm(0.975)

 #Get distribution (Norm or LogNorm)
 if (norm == "r") {

 #Get Normal Distribution
 out = rnorm(n = 1, m, se)

 } else if (norm == "rl") {

 #Calculate shape parameters
 location <- log(m^2 / sqrt(se^2 + m^2))
 shape <- sqrt(log(1 + (se^2 / m^2)))

 #Get LogNormal distribution
 out = rlnorm(n = 1, location, shape)

 }

 out

 }

 variable_lwr <- paste0(variable, "_lwr")
 variable_upr <- paste0(variable, "_upr")
 df[[variable]] <- apply(df[,c(variable, variable_lwr, variable_upr)],
 1,
 apply_rnorm,
 c1 = variable,
 c2 = variable_upr,
 c3 = variable_lwr)

 if (constrain == TRUE){

 df[[variable]][df[[variable]] < 0] <- 0
 df[[variable]][df[[variable]] > 1] <- 1
 }

 return(df)
}


whoilo_calculate_deaths <- function(exposure,
 year,
 ghe_code,
 reference,
 rr_df) {


 exposure <- exposure %>%
 mutate(h0p = ifelse(test = (reference == 0), yes = 1, no = 0),
 h1p = ifelse(test = (reference == 1), yes = 1, no = 0),
 h2p = ifelse(test = (reference == 2), yes = 1, no = 0),
 h3p = ifelse(test = (reference == 3), yes = 1, no = 0),
 h4p = ifelse(test = (reference == 4), yes = 1, no = 0),
 h5p = ifelse(test = (reference == 5), yes = 1, no = 0))

 exposure <- exposure %>%

 mutate(paf = ( (h0 * (rr_df$rr[1] - 1)) +
 (h1 * (rr_df$rr[2] - 1)) +
 (h2 * (rr_df$rr[3] - 1)) +
 (h3 * (rr_df$rr[4] - 1)) +
 (h4 * (rr_df$rr[5] - 1)) +
 (h5 * (rr_df$rr[6] - 1)) ) /
 ( (h0 * (rr_df$rr[1] - 1) + 1) * h0p +
 (h1 * (rr_df$rr[2] - 1) + 1) * h1p +
 (h2 * (rr_df$rr[3] - 1) + 1) * h2p +
 (h3 * (rr_df$rr[4] - 1) + 1) * h3p +
 (h4 * (rr_df$rr[5] - 1) + 1) * h4p +
 (h5 * (rr_df$rr[6] - 1) + 1) * h5p )
 ) %>%

 select(-h0p, -h1p, -h2p, -h3p, -h4p, -h5p) %>%
 mutate(paf = case_when(is.infinite(paf) ~ 0,
 TRUE ~ paf),
 paf = case_when(paf < 0 ~ 0,
 paf > 1 ~ 1,
 TRUE ~ paf))

 calculate_paf_CI <- function(i,
 reference){

 rnd_rr <- whoilo_randomize_1parameter(rr_df, "rr", norm = "rl")$rr

 rnd_h <- whoilo_randomize_exposure(exposure)

 tmp <- rnd_h %>%
 mutate(h0p = ifelse(test = (reference == 0), yes = 1, no = 0),
 h1p = ifelse(test = (reference == 1), yes = 1, no = 0),
 h2p = ifelse(test = (reference == 2), yes = 1, no = 0),
 h3p = ifelse(test = (reference == 3), yes = 1, no = 0),
 h4p = ifelse(test = (reference == 4), yes = 1, no = 0),
 h5p = ifelse(test = (reference == 5), yes = 1, no = 0)) %>%

 mutate(paf = ( (h0 * (rnd_rr[1] - 1)) +
 (h1 * (rnd_rr[2] - 1)) +
 (h2 * (rnd_rr[3] - 1)) +
 (h3 * (rnd_rr[4] - 1)) +
 (h4 * (rnd_rr[5] - 1)) +
 (h5 * (rnd_rr[6] - 1)) ) /
 ( (h0 * (rnd_rr[1] - 1) + 1) * h0p +
 (h1 * (rnd_rr[2] - 1) + 1) * h1p +
 (h2 * (rnd_rr[3] - 1) + 1) * h2p +
 (h3 * (rnd_rr[4] - 1) + 1) * h3p +
 (h4 * (rnd_rr[5] - 1) + 1) * h4p +
 (h5 * (rnd_rr[6] - 1) + 1) * h5p )
 ) %>%

 select(-h0p, -h1p, -h2p, -h3p, -h4p, -h5p) %>%
 mutate(paf =
 case_when(
 paf < 0 ~ 0,
 paf > 1 ~ 1,
 TRUE ~ paf),
 i = !!i)

 return(tmp)
 }

 out <- foreach::foreach(i = c(1:1000)) %dopar% {
 calculate_paf_CI(i, reference)
 }

 out <- data.table::rbindlist(out)

 out_ci <- out %>%
 group_by(country_abbrev, sex, age_bands_5yr) %>%
 summarise(paf_median = quantile(paf, probs = 0.5, na.rm = TRUE),
 paf_lwr = quantile(paf, probs = 0.025, na.rm = TRUE),
 paf_upr = quantile(paf, probs = 0.975, na.rm = TRUE)) %>%
 ungroup %>%
 transmute(country_abbrev, sex, age_bands_5yr,
 paf_median =
 case_when(age_bands_5yr == "15_19" ~ 0,
 paf_median < 0 ~ 0,
 paf_median > 1 ~ 1,
 TRUE ~ paf_median),
 paf_lwr =
 case_when(age_bands_5yr == "15_19" ~ 0,
 paf_lwr < 0 ~ 0,
 paf_lwr > 1 ~ 1,
 TRUE ~ paf_lwr),
 paf_upr =
 case_when(age_bands_5yr == "15_19" ~ 0,
 paf_upr < 0 ~ 0,
 paf_upr > 1 ~ 1,
 TRUE ~ paf_upr))

 exposure <- left_join(exposure, out_ci, by = c("country_abbrev", "sex", "age_bands_5yr"))


 dths_df <- case_when(!!ghe_code == 0691 ~ "who_ilo_long_ghe_0691",
 !!ghe_code == 0692 ~ "who_ilo_long_ghe_0692",
 !!ghe_code == 1130 ~ "who_ilo_long_ghe_1130",
 !!ghe_code == 1140 ~ "who_ilo_long_ghe_1140",
 TRUE ~ NA_character_)
 dths_df <- get(dths_df)

 dths_df <- dths_df %>%
 filter(year == !!year,
 age_bands_5yr != "0_4",
 age_bands_5yr != "5_9",
 age_bands_5yr != "10_14")

 calculate_deats_CI <- function(i,
 exposure,
 dths_df,
 ghe_code,
 year){

 rnd_dths <- whoilo_randomize_1parameter(dths_df, "dths") %>%
 mutate(dths = case_when(
 dths < 0 ~ 0,
 TRUE ~ dths))
 rnd_exposure <- whoilo_randomize_1parameter(exposure, "paf") %>%
 mutate(paf = case_when(
 paf < 0 ~ 0,
 paf >1 ~ 1,
 TRUE ~ paf))

 tmp <- rnd_exposure %>%
 mutate(ghe_code = !!ghe_code,
 year = !!year) %>%
 left_join(rnd_dths, by = c("region", "country_abbrev", "year", "sex", "age_bands_5yr", "ghe_code")) %>%
 select(-ghe_code, -ghe_name) %>%
 mutate(pad = paf * dths,
 i = !!i)
 return(tmp)
 }

 out <- foreach::foreach(i = c(1:1000)) %dopar% {
 calculate_deats_CI(i, exposure, dths_df, ghe_code, year)
 }

 out <- data.table::rbindlist(out)

 out_ci <- out %>%
 group_by(country_abbrev, sex, age_bands_5yr) %>%
 summarise(pad_median = quantile(pad, probs = 0.5, na.rm = TRUE),
 pad_lwr = quantile(pad, probs = 0.025, na.rm = TRUE),
 pad_upr = quantile(pad, probs = 0.975, na.rm = TRUE)) %>%
 ungroup %>%
 transmute(country_abbrev, sex, age_bands_5yr,
 pad_median =
 case_when(age_bands_5yr == "15_19" ~ 0,
 pad_median < 0 ~ 0,
 TRUE ~ pad_median),
 pad_lwr =
 case_when(age_bands_5yr == "15_19" ~ 0,
 pad_lwr < 0 ~ 0,
 TRUE ~ pad_lwr),
 pad_upr =
 case_when(age_bands_5yr == "15_19" ~ 0,
 pad_upr < 0 ~ 0,
 TRUE ~ pad_upr))

 exposure <- exposure %>%
 mutate(ghe_code = !!ghe_code,
 year = !!year) %>%
 left_join(dths_df, by = c("region", "country_abbrev", "year", "sex", "age_bands_5yr", "ghe_code")) %>%
 select(-ghe_code, -ghe_name) %>%
 mutate(pad = paf * dths)

 exposure <- left_join(exposure, out_ci, by = c("country_abbrev", "sex", "age_bands_5yr")) %>%
 mutate(year = !!year)

 exposure <- exposure %>%
 select(-contains("h0"), -contains("h1"), -contains("h2"), -contains("h3"), -contains("h4"), -contains("median"))
 return(exposure)
}

whoilo_calculate_dalys <- function(exposure,
 year,
 ghe_code,
 reference,
 rr_df){
 exposure[is.na(exposure)] <- 0

 exposure <- exposure %>%
 mutate(h0p = ifelse(test = (reference == 0), yes = 1, no = 0),
 h1p = ifelse(test = (reference == 1), yes = 1, no = 0),
 h2p = ifelse(test = (reference == 2), yes = 1, no = 0),
 h3p = ifelse(test = (reference == 3), yes = 1, no = 0),
 h4p = ifelse(test = (reference == 4), yes = 1, no = 0),
 h5p = ifelse(test = (reference == 5), yes = 1, no = 0))

 exposure <- exposure %>%

 mutate(paf = ( (h0 * (rr_df$rr[1] - 1)) +
 (h1 * (rr_df$rr[2] - 1)) +
 (h2 * (rr_df$rr[3] - 1)) +
 (h3 * (rr_df$rr[4] - 1)) +
 (h4 * (rr_df$rr[5] - 1)) +
 (h5 * (rr_df$rr[6] - 1)) ) /
 ( (h0 * (rr_df$rr[1] - 1) + 1) * h0p +
 (h1 * (rr_df$rr[2] - 1) + 1) * h1p +
 (h2 * (rr_df$rr[3] - 1) + 1) * h2p +
 (h3 * (rr_df$rr[4] - 1) + 1) * h3p +
 (h4 * (rr_df$rr[5] - 1) + 1) * h4p +
 (h5 * (rr_df$rr[6] - 1) + 1) * h5p )
 ) %>%

 select(-h0p, -h1p, -h2p, -h3p, -h4p, -h5p) %>%
 mutate(paf = case_when(is.infinite(paf) ~ 0,
 TRUE ~ paf),
 paf = case_when(paf < 0 ~ 0,
 paf > 1 ~ 1,
 TRUE ~ paf))

 calculate_paf_CI <- function(i,
 reference){


 rnd_rr <- whoilo_randomize_1parameter(rr_df, "rr", norm = "rl")$rr

 rnd_h <- whoilo_randomize_exposure(exposure)

 tmp <- rnd_h %>%
 mutate(h0p = ifelse(test = (reference == 0), yes = 1, no = 0),
 h1p = ifelse(test = (reference == 1), yes = 1, no = 0),
 h2p = ifelse(test = (reference == 2), yes = 1, no = 0),
 h3p = ifelse(test = (reference == 3), yes = 1, no = 0),
 h4p = ifelse(test = (reference == 4), yes = 1, no = 0),
 h5p = ifelse(test = (reference == 5), yes = 1, no = 0)) %>%

 mutate(paf = ( (h0 * (rnd_rr[1] - 1)) +
 (h1 * (rnd_rr[2] - 1)) +
 (h2 * (rnd_rr[3] - 1)) +
 (h3 * (rnd_rr[4] - 1)) +
 (h4 * (rnd_rr[5] - 1)) +
 (h5 * (rnd_rr[6] - 1)) ) /
 ( (h0 * (rnd_rr[1] - 1) + 1) * h0p +
 (h1 * (rnd_rr[2] - 1) + 1) * h1p +
 (h2 * (rnd_rr[3] - 1) + 1) * h2p +
 (h3 * (rnd_rr[4] - 1) + 1) * h3p +
 (h4 * (rnd_rr[5] - 1) + 1) * h4p +
 (h5 * (rnd_rr[6] - 1) + 1) * h5p )
 ) %>%

 select(-h0p, -h1p, -h2p, -h3p, -h4p, -h5p) %>%
 mutate(paf =
 case_when(
 paf < 0 ~ 0,
 paf > 1 ~ 1,
 TRUE ~ paf),
 i = !!i)
 return(tmp)
 }

 out <- foreach::foreach(i = c(1:1000)) %dopar% {
 calculate_paf_CI(i, reference)
 }
 out <- data.table::rbindlist(out)


 out_ci <- out %>%
 group_by(country_abbrev, sex, age_bands_5yr) %>%
 summarise(paf_median = quantile(paf, probs = 0.5, na.rm = TRUE),
 paf_lwr = quantile(paf, probs = 0.025, na.rm = TRUE),
 paf_upr = quantile(paf, probs = 0.975, na.rm = TRUE)) %>%
 ungroup %>%
 transmute(country_abbrev, sex, age_bands_5yr,
 paf_median =
 case_when(age_bands_5yr == "15_19" ~ 0,
 paf_median < 0 ~ 0,
 paf_median > 1 ~ 1,
 TRUE ~ paf_median),
 paf_lwr =
 case_when(age_bands_5yr == "15_19" ~ 0,
 paf_lwr < 0 ~ 0,
 paf_lwr > 1 ~ 1,
 TRUE ~ paf_lwr),
 paf_upr =
 case_when(age_bands_5yr == "15_19" ~ 0,
 paf_upr < 0 ~ 0,
 paf_upr > 1 ~ 1,
 TRUE ~ paf_upr))

 exposure <- left_join(exposure, out_ci, by = c("country_abbrev", "sex", "age_bands_5yr"))

 daly_df <- case_when(!!ghe_code == 0691 ~ "who_ilo_long_daly_0691",
 !!ghe_code == 0692 ~ "who_ilo_long_daly_0692",
 !!ghe_code == 1130 ~ "who_ilo_long_daly_1130",
 !!ghe_code == 1140 ~ "who_ilo_long_daly_1140",
 TRUE ~ NA_character_)
 daly_df <- get(daly_df)

 daly_df <- daly_df %>%
 filter(year == !!year)

 calculate_incidences_CI <- function(i,
 exposure,
 daly_df,
 ghe_code,
 year){
 rnd_exposure <- whoilo_randomize_1parameter(exposure, "paf") %>%
 mutate(paf = case_when(
 paf < 0 ~ 0,
 paf >1 ~ 1,
 TRUE ~ paf))

 tmp <- rnd_exposure %>%
 mutate(ghe_code = !!ghe_code,
 year = !!year) %>%
 left_join(daly_df, by = c("region", "country_abbrev", "year", "sex", "age_bands_5yr", "ghe_code")) %>%
 select(-ghe_code, -ghe_name) %>%
 mutate(pad = paf * daly,
 i = !!i)
 return(tmp)
 }

 out <- foreach(i = c(1:1000)) %dopar% {
 calculate_incidences_CI(i, exposure, daly_df, ghe_code, year)
 }

 out <- data.table::rbindlist(out)

 out_ci <- out %>%
 group_by(country_abbrev, sex, age_bands_5yr) %>%
 summarise(pad_median = quantile(pad, probs = 0.5, na.rm = TRUE),
 pad_lwr = quantile(pad, probs = 0.025, na.rm = TRUE),
 pad_upr = quantile(pad, probs = 0.975, na.rm = TRUE)) %>%
 ungroup %>%
 transmute(country_abbrev, sex, age_bands_5yr,
 pad_median =
 case_when(age_bands_5yr == "15_19" ~ 0,
 pad_median < 0 ~ 0,
 TRUE ~ pad_median),
 pad_lwr =
 case_when(age_bands_5yr == "15_19" ~ 0,
 pad_lwr < 0 ~ 0,
 TRUE ~ pad_lwr),
 pad_upr =
 case_when(age_bands_5yr == "15_19" ~ 0,
 pad_upr < 0 ~ 0,
 TRUE ~ pad_upr))

 exposure <- exposure %>%
 mutate(ghe_code = !!ghe_code,
 year = !!year) %>%
 left_join(daly_df, by = c("region", "country_abbrev", "year", "sex", "age_bands_5yr", "ghe_code")) %>%
 select(-ghe_code, -ghe_name) %>%
 mutate(pad = paf * daly)


 exposure <- left_join(exposure, out_ci, by = c("country_abbrev", "sex", "age_bands_5yr")) %>%
 mutate(year = !!year)

 exposure <- exposure %>%
 select(-contains("h0"), -contains("h1"), -contains("h2"), -contains("h3"), -contains("h4"), -contains("median"))
 return(exposure)
}

whoilo_randomize_exposure <- function(df){
 apply_rnorm <- function(x, c1, c2, c3) rnorm(n =1,
 mean = x[c1],
 sd = 0.255102 * (x[c2] -x[c3]))
 for (i in c("h1", "h2", "h3", "h4", "h5")){
 i_lwr <- paste0(i, "_lwr")
 i_upr <- paste0(i, "_upr")
 df[[i]] <- apply(df[,c(i, i_lwr, i_upr)],
 1,
 apply_rnorm,
 c1 = i,
 c2 = i_upr,
 c3 = i_lwr)
 }
 df <- df %>%
 mutate(
 h1 = case_when(
 h1 <0 ~ 0,
 h1 >1 ~ 1,
 TRUE ~ h1),
 h2 = case_when(
 h2 <0 ~ 0,
 h2 >1 ~ 1,
 TRUE ~ h2),
 h3 = case_when(
 h3 <0 ~ 0,
 h3 >1 ~ 1,
 TRUE ~ h3),
 h4 = case_when(
 h4 <0 ~ 0,
 h4 >1 ~ 1,
 TRUE ~ h4),
 h5 = case_when(
 h5 <0 ~ 0,
 h5 >1 ~ 1,
 TRUE ~ h5)
 )

 df$h0 <- 1 - df$h1 - df$h2 - df$h3 - df$h4 - df$h5

 df <- df %>%
 mutate(
 h0 = case_when(
 h0 <0 ~ 0,
 h0 >1 ~ 1,
 TRUE ~ h0))

 return(df)
}

format_DALY <- function(df_in) {
 df_in %>%
 mutate(DALYenvelope_lwr = NA,
 DALYenvelope_upr = NA) %>%
 select(region, country_abbrev, year, sex, ilo_age_bands_5yr = age_bands_5yr, cause,
 everything(), -starts_with("rr"),
 DALYpaf = paf, DALYpaf_lwr = paf_lwr, DALYpaf_upr = paf_upr,
 DALYenvelope = daly,
 DALYenvelope_lwr = DALYenvelope_lwr,
 DALYenvelope_upr = DALYenvelope_upr,
 DALY = pad,
 DALY_lwr = pad_lwr,
 DALY_upr = pad_upr,
 -source) %>%
 pivot_longer(cols = h5:DALYenvelope_upr, names_to = "outcome", values_to = "result") %>%
 separate(col = outcome, into = c("outcome", "type"), sep = "_") %>%
 pivot_wider(names_from = type, values_from = result, names_sep = "_") %>%
 rename(result = "NA",
 result_lwr = lwr,
 result_upr = upr) %>%
 mutate(cause = case_when(
 outcome %in% c("h0", "h1", "h2", "h3", "h4", "h5") ~ "exposure",
 TRUE ~ cause))
}


format_DTHS <- function(df_in) {
 df_in %>%
 select(-source) %>%
 mutate(ilo_age_bands_5yr = age_bands_5yr) %>%
 select(-age_bands_5yr) %>%
 group_by(country_abbrev, sex, region, ilo_age_bands_5yr, year, cause) %>%
 summarise_all(sum) %>%
 ungroup %>%

 select(region, country_abbrev, year, sex, ilo_age_bands_5yr, cause,
 everything(), -starts_with("rr"),
 DTHpaf = paf, DTHpaf_lwr = paf_lwr, DTHpaf_upr = paf_upr,
 DTHenvelope = dths,
 DTHenvelope_lwr = dths_lwr,
 DTHenvelope_upr = dths_upr,
 DTH = pad,
 DTH_lwr = pad_lwr,
 DTH_upr = pad_upr) %>%
 pivot_longer(cols = h5:DTH_upr, names_to = "outcome", values_to = "result") %>%
 separate(col = outcome, into = c("outcome", "type"), sep = "_") %>%
 pivot_wider(names_from = type, values_from = result, names_sep = "_") %>%
 rename(result = "NA",
 result_lwr = lwr,
 result_upr = upr) %>%
 mutate(cause = case_when(
 outcome %in% c("h0", "h1", "h2", "h3", "h4", "h5") ~ "exposure",
 TRUE ~ cause))
}


whoilo_BOD_to_excel <- function(df_in,
 year,
 outcome,
 cause,
 wb,
 sheet,
 format = "print"){
 tmp1 <- my_func2(df_in = df_in,
 year = year,
 outcome = outcome,
 cause = cause,
 breakdown = c("total", "sex", "age", "sexage"),
 level = "global",
 format = format) %>%
 mutate(region = "global",
 country_abbrev = NA) %>%
 select(year, region, country_abbrev , everything())
 tmp1[nrow(tmp1)+1,] <- NA
 tmp2 <- my_func2(df_in = df_in,
 year = year,
 outcome = outcome,
 cause = cause,
 breakdown = c("total", "sex", "age", "sexage"),
 level = "regional",
 format = format) %>%
 mutate(country_abbrev = NA) %>%
 select(year, region, country_abbrev , everything())
 tmp2[nrow(tmp2)+1,] <- NA
 tmp3 <- my_func2(df_in = df_in,
 year = year,
 outcome = outcome,
 cause = cause,
 breakdown = c("total", "sex", "age", "sexage"),
 level = "national",
 format = format)
 tmp <- tmp1 %>%
 bind_rows(tmp2) %>%
 bind_rows(tmp3) %>%
 select(-contains("sd"))

 country_names <- who_ilo_long_countries %>%
 select(country_name_who, country_abbrev)
 tmp <- tmp %>%
 left_join(country_names, by = c("country_abbrev")) %>%
 select(year, region, country_abbrev, country_name_who, everything()) %>%
 filter(!(country_abbrev %in% c("PSE", "HKG", "TWN")))

 if (outcome == "DALYenvelope"){
 scale2 <- function(x) (x*NA)
 tmp <- tmp %>%
 mutate_at(vars(contains("_lwr")), scale2) %>%
 mutate_at(vars(contains("_upr")), scale2)
 }

 addWorksheet(wb = wb,
 sheetName = sheet)
 writeData(wb = wb,
 sheet = sheet,
 x = tmp,
 startCol = 1,
 startRow = 1)


 freezePane(wb = wb,
 sheet = sheet,
 firstActiveRow = 2,
 firstActiveCol = 5)
}
my_func2 <- function(df_in,
 year,
 outcome,
 cause,
 breakdown,
 level,
 format = NULL){
 if (level == "global"){
 df_out <- my_func_global(df_in = df_in,
 year = year,
 outcome = outcome,
 cause = cause,
 breakdown = breakdown)
 }
 if (level == "regional"){
 df_out <- my_func_regional(df_in = df_in,
 year = year,
 outcome = outcome,
 cause = cause,
 breakdown = breakdown)
 }
 if (level == "national"){
 df_out <- my_func_national(df_in = df_in,
 year = year,
 outcome = outcome,
 cause = cause,
 breakdown = breakdown)
 }
 if (format == "print"){
 if (outcome %in% c("DTH", "DALY", "DTHenvelope", "DALYenvelope", "DTHS", "DALYS", "h0_number", "h1_number", "h2_number", "h3_number", "h4_number", "h5_number")) {
 scale2 <- function(x) (case_when(x <0 ~0, TRUE ~ x))
 df_out <- df_out %>%
 mutate_if(is.numeric, round) %>%
 mutate(year = !!year) %>%
 mutate_at(vars(contains("_lwr")), scale2)
 }
 if (outcome %in% c("DTHrate", "DALYrate")) {
 scale2 <- function(x) (case_when(x <0 ~0, TRUE ~ x))
 df_out <- df_out %>%
 mutate_if(is.numeric, round, digits = 2) %>%
 mutate(year = !!year) %>%
 mutate_at(vars(contains("_lwr")), scale2)
 }
 if (outcome %in% c("PAF", "DTHpaf", "DALYpaf", "h0", "h1", "h2", "h3", "h4", "h5", "h45")) {
 scale2 <- function(x) (case_when(x <0 ~0, TRUE ~ x))
 df_out <- df_out %>%
 mutate_at(vars(contains("_lwr")), scale2) %>%
 mutate_if(is.numeric, function(x){x = format(round(x*100, 2), nsmall = 2)}) %>%
 mutate(year = !!year)
 }
 if (outcome %in% c("population")) {
 df_out <- df_out %>%
 mutate_if(is.numeric, round) %>%
 mutate(year = !!year)
 }
 }
 return(df_out)
}
my_func_global <- function(df_in,
 year,
 outcome,
 cause,
 breakdown,
 shape = "wide"){
 # total
 if ("total" %in% breakdown){
 if (outcome %in% c("PAF", "DTHpaf")){
 envelope_stroke_global <- df_in %>%
 filter(outcome == "DTHenvelope",
 year == !!year,
 cause == !!cause) %>%
 arrange(region, country_abbrev, year, sex, ilo_age_bands_5yr) %>%
 drop_na() %>%
 mutate(sd = (result_upr - result_lwr)/2/1.96) %>%
 summarise(result = sum(result),
 sd = sqrt(sum((sd)^2))) %>%
 mutate(result_lwr = qnorm(p = 0.025, mean = result, sd = sd),
 result_upr = qnorm(p = 0.975, mean = result, sd = sd)) %>%
 ungroup() %>%

 select_all(.funs = funs(str_c(., "1")))

 dths_stroke_global <- df_in %>%
 filter(outcome == "DTH",
 year == !!year,
 cause == !!cause) %>%
 arrange(region, country_abbrev, year, sex, ilo_age_bands_5yr) %>%
 drop_na() %>%
 mutate(sd = (result_upr - result_lwr)/2/1.96) %>%
 summarise(result = sum(result),
 sd = sqrt(sum((sd)^2))) %>%
 mutate(result_lwr = qnorm(p = 0.025, mean = result, sd = sd),
 result_upr = qnorm(p = 0.975, mean = result, sd = sd)) %>%
 ungroup
 dths_stroke_global <- bind_cols(dths_stroke_global, envelope_stroke_global) %>%
 rowwise() %>%
 transmute(result_lwr_b = whoilo_paf_summary(result, result1, sd, sd1, CI = 0.025),
 result_upr_b = whoilo_paf_summary(result, result1, sd, sd1, CI = 0.975),
 result_b = whoilo_paf_summary(result, result1, CI = NULL)) %>%
 mutate(sd_b = (result_upr_b - result_lwr_b)/2/1.96) %>%
 ungroup() %>%
 select(result_b, sd_b, result_lwr_b, result_upr_b)
 if (shape == "long") {
 dths_stroke_global <- dths_stroke_global %>%
 mutate(sex = "b",
 age = "15_") %>%
 select(sex, age, result = result_b, sd = sd_b, result_lwr = result_lwr_b, result_upr = result_upr_b)
 }
 }
 if (outcome %in% c("DALYpaf")){
 envelope_stroke_global <- df_in %>%
 filter(outcome == "DALYenvelope",
 year == !!year,
 cause == !!cause) %>%
 mutate(result_lwr = case_when(is.na(result_lwr) ~ result,
 TRUE ~ result_lwr),
 result_upr = case_when(is.na(result_upr) ~ result,
 TRUE ~ result_upr)) %>%
 arrange(region, country_abbrev, year, sex, ilo_age_bands_5yr) %>%
 drop_na(region, result) %>%
 mutate(sd = (result_upr - result_lwr)/2/1.96) %>%
 summarise(result = sum(result),
 sd = sqrt(sum((sd)^2))) %>%
 mutate(result_lwr = qnorm(p = 0.025, mean = result, sd = sd),
 result_upr = qnorm(p = 0.975, mean = result, sd = sd)) %>%
 ungroup() %>%

 select_all(.funs = funs(str_c(., "1")))

 dths_stroke_global <- df_in %>%
 filter(outcome == "DALY",
 year == !!year,
 cause == !!cause) %>%
 arrange(region, country_abbrev, year, sex, ilo_age_bands_5yr) %>%
 drop_na(region, result) %>%
 mutate(sd = (result_upr - result_lwr)/2/1.96) %>%
 summarise(result = sum(result),
 sd = sqrt(sum((sd)^2))) %>%
 mutate(result_lwr = qnorm(p = 0.025, mean = result, sd = sd),
 result_upr = qnorm(p = 0.975, mean = result, sd = sd)) %>%
 ungroup
 dths_stroke_global <- bind_cols(dths_stroke_global, envelope_stroke_global) %>%
 rowwise() %>%
 transmute(result_lwr_b = whoilo_paf_summary(result, result1, sd, sd1, CI = 0.025),
 result_upr_b = whoilo_paf_summary(result, result1, sd, sd1, CI = 0.975),
 result_b = whoilo_paf_summary(result, result1, CI = NULL)) %>%
 mutate(sd_b = (result_upr_b - result_lwr_b)/2/1.96) %>%
 ungroup() %>%
 select(result_b, sd_b, result_lwr_b, result_upr_b)
 if (shape == "long") {
 dths_stroke_global <- dths_stroke_global %>%
 mutate(sex = "b",
 age = "15_") %>%
 select(sex, age, result = result_b, sd = sd_b, result_lwr = result_lwr_b, result_upr = result_upr_b)
 }
 }
 if (outcome %in% c("h0", "h1", "h2", "h3", "h4", "h5")){
 dths_stroke_global <- df_in %>%
 filter(outcome == !!outcome,
 year == !!year,
 cause == !!cause) %>%
 arrange(region, country_abbrev, year, sex, ilo_age_bands_5yr) %>%
 mutate(sd = (result_upr - result_lwr)/2/1.96) %>%
 left_join(who_ilo_long_populations, by = c("region", "country_abbrev", "year", "sex", "ilo_age_bands_5yr" = "age_bands_5yr")) %>%
 mutate(result = result*population,
 sd = sd*population) %>%
 drop_na() %>%
 summarise(result = sum(result),
 sd = sqrt(sum((sd)^2)),
 population = sum(population)) %>%
 mutate(result_lwr = qnorm(p = 0.025, mean = result, sd = sd)/ population,
 result_upr = qnorm(p = 0.975, mean = result, sd = sd)/ population,
 result = result / population,
 sd = sd/population) %>%
 select(-population) %>%
 ungroup
 dths_stroke_global[dths_stroke_global < 0] <- 0
 if (shape == "wide") {
 dths_stroke_global <- dths_stroke_global %>%
 mutate(sex = "b") %>%
 pivot_wider(names_from = sex, values_from = result:result_upr)
 }
 }
 if (outcome %in% c("h0_number", "h1_number", "h2_number", "h3_number", "h4_number", "h5_number")){
 outcome2 <- str_sub(outcome, end = -8L)
 dths_stroke_global <- df_in %>%
 filter(outcome == !!outcome2,
 year == !!year,
 cause == !!cause) %>%
 arrange(region, country_abbrev, year, sex, ilo_age_bands_5yr) %>%
 mutate(sd = (result_upr - result_lwr)/2/1.96) %>%
 left_join(who_ilo_long_populations, by = c("region", "country_abbrev", "year", "sex", "ilo_age_bands_5yr" = "age_bands_5yr")) %>%
 mutate(population = population * 1000) %>%
 mutate(result = result*population,
 sd = sd*population) %>%
 drop_na() %>%
 summarise(result_b = sum(result),
 sd_b = sqrt(sum((sd)^2)),
 population = sum(population)) %>%
 mutate(result_lwr_b = qnorm(p = 0.025, mean = result_b, sd = sd_b),
 result_upr_b = qnorm(p = 0.975, mean = result_b, sd = sd_b),
 result_b = result_b,
 sd_b = sd_b) %>%
 select(-population) %>%
 ungroup
 dths_stroke_global[dths_stroke_global < 0] <- 0
 if (shape == "long") {
 dths_stroke_global <- dths_stroke_global %>%
 mutate(sex = "b",
 age = "15_") %>%
 select(sex, age, result = result_b, sd = sd_b, result_lwr = result_lwr_b, result_upr = result_upr_b)
 }
 }
 if (outcome %in% c("DTH", "DALY", "DTHenvelope", "DALYenvelope")) {
 dths_stroke_global <- df_in %>%
 filter(outcome == !!outcome,
 year == !!year,
 cause == !!cause) %>%
 mutate(result_lwr = case_when(is.na(result_lwr) ~ result,
 TRUE ~ result_lwr),
 result_upr = case_when(is.na(result_upr) ~ result,
 TRUE ~ result_upr)) %>%
 arrange(region, country_abbrev, year, sex, ilo_age_bands_5yr) %>%
 mutate(sd = (result_upr - result_lwr)/2/1.96) %>%
 drop_na(region, result) %>%
 summarise(result = sum(result),
 sd = sqrt(sum((sd)^2))) %>%
 mutate(result_lwr = qnorm(p = 0.025, mean = result, sd = sd),
 result_upr = qnorm(p = 0.975, mean = result, sd = sd)) %>%
 ungroup
 if (shape == "wide") {
 dths_stroke_global <- dths_stroke_global %>%
 mutate(sex = "b") %>%
 pivot_wider(names_from = sex, values_from = result:result_upr)
 }
 }
 if (outcome %in% c("DTHS", "DALYS")) {
 outcome2 <- str_sub(outcome, end = -2L)
 dths_stroke_global <- df_in %>%
 filter(outcome == !!outcome2,
 year == !!year,
 cause %in% c("stroke_h5", "ihd_h5")) %>%
 arrange(region, country_abbrev, year, sex, ilo_age_bands_5yr) %>%
 mutate(sd = (result_upr - result_lwr)/2/1.96) %>%
 drop_na() %>%
 summarise(result = sum(result),
 sd = sqrt(sum((sd)^2))) %>%
 mutate(result_lwr = qnorm(p = 0.025, mean = result, sd = sd),
 result_upr = qnorm(p = 0.975, mean = result, sd = sd)) %>%
 ungroup
 if (shape == "wide") {
 dths_stroke_global <- dths_stroke_global %>%
 mutate(sex = "b") %>%
 pivot_wider(names_from = sex, values_from = result:result_upr)
 }
 }
 if (outcome %in% c("h45")){
 dths_stroke_global <- df_in %>%
 filter(outcome %in% c("h4", "h5"),
 year == !!year,
 cause == !!cause) %>%
 arrange(region, country_abbrev, year, sex, ilo_age_bands_5yr) %>%
 mutate(sd = (result_upr - result_lwr)/2/1.96) %>%
 left_join(who_ilo_long_populations, by = c("region", "country_abbrev", "year", "sex", "ilo_age_bands_5yr" = "age_bands_5yr")) %>%
 mutate(result = result*population,
 sd = sd*population) %>%
 drop_na() %>%
 summarise(result = sum(result),
 sd = sqrt(sum((sd)^2)),
 population = sum(population)/2) %>%
 mutate(result_lwr = qnorm(p = 0.025, mean = result, sd = sd)/ population,
 result_upr = qnorm(p = 0.975, mean = result, sd = sd)/ population,
 result = result / population,
 sd = sd/population) %>%
 select(-population) %>%
 ungroup
 dths_stroke_global[dths_stroke_global < 0] <- 0
 if (shape == "wide") {
 dths_stroke_global <- dths_stroke_global %>%
 mutate(sex = "b") %>%
 pivot_wider(names_from = sex, values_from = result:result_upr)
 }
 }
 if (outcome %in% c("DTHrate")){
 outcome2 <- "DTH"
 dths_stroke_global <- df_in %>%
 filter(outcome == !!outcome2,
 year == !!year,
 cause == !!cause) %>%
 arrange(region, country_abbrev, year, sex, ilo_age_bands_5yr) %>%
 mutate(sd = (result_upr - result_lwr)/2/1.96) %>%
 left_join(who_ilo_long_populations, by = c("region", "country_abbrev", "year", "sex", "ilo_age_bands_5yr" = "age_bands_5yr")) %>%
 drop_na() %>%
 summarise(result = sum(result),
 sd = sqrt(sum((sd)^2)),
 population = sum(population)) %>%
 mutate(population = population / 100,
 result = result / population,
 sd = sd / population,
 result_lwr = qnorm(p = 0.025, mean = result, sd = sd),
 result_upr = qnorm(p = 0.975, mean = result, sd = sd)) %>%
 select(-population) %>%
 ungroup
 if (shape == "wide") {
 dths_stroke_global <- dths_stroke_global %>%
 mutate(sex = "b") %>%
 pivot_wider(names_from = sex, values_from = result:result_upr)
 }
 }
 if (outcome %in% c("DALYrate")){
 outcome2 <- "DALY"
 dths_stroke_global <- df_in %>%
 filter(outcome == !!outcome2,
 year == !!year,
 cause == !!cause) %>%
 arrange(region, country_abbrev, year, sex, ilo_age_bands_5yr) %>%
 mutate(sd = (result_upr - result_lwr)/2/1.96) %>%
 left_join(who_ilo_long_populations, by = c("region", "country_abbrev", "year", "sex", "ilo_age_bands_5yr" = "age_bands_5yr")) %>%
 drop_na() %>%
 summarise(result = sum(result),
 sd = sqrt(sum((sd)^2)),
 population = sum(population)) %>%
 mutate(population = population,
 result = result / population * 100000,
 sd = sd / population * 100000,
 result_lwr = qnorm(p = 0.025, mean = result, sd = sd),
 result_upr = qnorm(p = 0.975, mean = result, sd = sd)) %>%
 select(-population) %>%
 ungroup
 if (shape == "wide") {
 dths_stroke_global <- dths_stroke_global %>%
 mutate(sex = "b") %>%
 pivot_wider(names_from = sex, values_from = result:result_upr)
 }
 }
 if (outcome %in% c("population")){
 dths_stroke_global <- who_ilo_long_populations %>%
 filter(age_bands_5yr %in% c("15_19", "20_24", "25_29", "30_34", "35_39", "40_44", "45_49", "50_54", "55_59", "60_64", "65_69", "70_74", "75_79", "80_84", "85_89", "90_94", "95_"),
 year == !!year) %>%
 arrange(region, country_abbrev, year, sex, age_bands_5yr) %>%
 drop_na() %>%
 summarise(result_b = sum(population)) %>%
 ungroup %>%
 mutate(sd_b = NA,
 result_lwr_b = NA,
 result_upr_b = NA)
 if (shape == "long") {
 dths_stroke_global <- dths_stroke_global %>%
 mutate(sex = "b",
 age = "15_") %>%
 select(sex, age, result = result_b, sd = sd_b, result_lwr = result_lwr_b, result_upr = result_upr_b)
 }
 }
 if (outcome %in% c("DTHratio")){
 envelope_stroke_global <- df_in %>%
 filter(outcome == "DTHparent",
 year == !!year,
 cause == !!cause) %>%
 arrange(region, country_abbrev, year, sex, ilo_age_bands_5yr) %>%
 drop_na() %>%
 mutate(sd = (result_upr - result_lwr)/2/1.96) %>%
 summarise(result = sum(result),
 sd = sqrt(sum((sd)^2))) %>%
 mutate(result_lwr = qnorm(p = 0.025, mean = result, sd = sd),
 result_upr = qnorm(p = 0.975, mean = result, sd = sd)) %>%
 ungroup()

 dths_stroke_global <- df_in %>%
 filter(outcome == "DTHenvelope",
 year == !!year,
 cause == !!cause) %>%
 arrange(region, country_abbrev, year, sex, ilo_age_bands_5yr) %>%
 drop_na() %>%
 mutate(sd = (result_upr - result_lwr)/2/1.96) %>%
 summarise(result = sum(result),
 sd = sqrt(sum((sd)^2))) %>%
 mutate(result_lwr = qnorm(p = 0.025, mean = result, sd = sd),
 result_upr = qnorm(p = 0.975, mean = result, sd = sd)) %>%
 ungroup() %>%

 select_all(.funs = funs(str_c(., "1")))

 dths_stroke_global <- bind_cols(dths_stroke_global, envelope_stroke_global) %>%
 rowwise() %>%
 transmute(result_lwr_b = whoilo_paf_summary(result, result1, sd, sd1, CI = 0.025),
 result_upr_b = whoilo_paf_summary(result, result1, sd, sd1, CI = 0.975),
 result_b = whoilo_paf_summary(result, result1, CI = NULL)) %>%
 mutate(sd_b = (result_upr_b - result_lwr_b)/2/1.96) %>%
 ungroup() %>%
 select(result_b, sd_b, result_lwr_b, result_upr_b)
 if (shape == "long") {
 dths_stroke_global <- dths_stroke_global %>%
 mutate(sex = "b",
 age = "15_") %>%
 select(sex, age, result = result_b, sd = sd_b, result_lwr = result_lwr_b, result_upr = result_upr_b)
 }
 }
 if (outcome %in% c("DALYratio")){
 envelope_stroke_global <- df_in %>%
 filter(outcome == "DALYparent",
 year == !!year,
 cause == !!cause) %>%
 arrange(region, country_abbrev, year, sex, ilo_age_bands_5yr) %>%
 drop_na() %>%
 mutate(sd = (result_upr - result_lwr)/2/1.96) %>%
 summarise(result = sum(result),
 sd = sqrt(sum((sd)^2))) %>%
 mutate(result_lwr = qnorm(p = 0.025, mean = result, sd = sd),
 result_upr = qnorm(p = 0.975, mean = result, sd = sd)) %>%
 ungroup()

 dths_stroke_global <- df_in %>%
 filter(outcome == "DALYenvelope",
 year == !!year,
 cause == !!cause) %>%
 mutate(result_lwr = case_when(is.na(result_lwr) ~ result,
 TRUE ~ result_lwr),
 result_upr = case_when(is.na(result_upr) ~ result,
 TRUE ~ result_upr)) %>%
 arrange(region, country_abbrev, year, sex, ilo_age_bands_5yr) %>%
 drop_na() %>%
 mutate(sd = (result_upr - result_lwr)/2/1.96) %>%
 summarise(result = sum(result),
 sd = sqrt(sum((sd)^2))) %>%
 mutate(result_lwr = qnorm(p = 0.025, mean = result, sd = sd),
 result_upr = qnorm(p = 0.975, mean = result, sd = sd)) %>%
 ungroup() %>%

 select_all(.funs = funs(str_c(., "1")))

 dths_stroke_global <- bind_cols(dths_stroke_global, envelope_stroke_global) %>%
 rowwise() %>%
 transmute(result_lwr_b = whoilo_paf_summary(result, result1, sd, sd1, CI = 0.025),
 result_upr_b = whoilo_paf_summary(result, result1, sd, sd1, CI = 0.975),
 result_b = whoilo_paf_summary(result, result1, CI = NULL)) %>%
 mutate(sd_b = (result_upr_b - result_lwr_b)/2/1.96) %>%
 ungroup() %>%
 select(result_b, sd_b, result_lwr_b, result_upr_b)
 if (shape == "long") {
 dths_stroke_global <- dths_stroke_global %>%
 mutate(sex = "b",
 age = "15_") %>%
 select(sex, age, result = result_b, sd = sd_b, result_lwr = result_lwr_b, result_upr = result_upr_b)
 }
 }
 }
 # by sex
 if ("sex" %in% breakdown){
 if (outcome %in% c("PAF", "DTHpaf")){
 envelope_stroke_sex_global <- df_in %>%
 filter(outcome == "DTHenvelope",
 year == !!year,
 cause == !!cause) %>%
 arrange(region, country_abbrev, year, sex, ilo_age_bands_5yr) %>%
 drop_na() %>%
 mutate(sd = (result_upr - result_lwr)/2/1.96) %>%
 group_by(sex) %>%
 summarise(result = sum(result),
 sd = sqrt(sum((sd)^2))) %>%
 ungroup %>%
 pivot_wider(names_from = sex, values_from = result:sd) %>%

 select_all(.funs = funs(str_c(., "1")))

 dths_stroke_sex_global <- df_in %>%
 filter(outcome == "DTH",
 year == !!year,
 cause == !!cause) %>%
 arrange(region, country_abbrev, year, sex, ilo_age_bands_5yr) %>%
 drop_na() %>%
 mutate(sd = (result_upr - result_lwr)/2/1.96) %>%
 group_by(sex) %>%
 summarise(result = sum(result),
 sd = sqrt(sum((sd)^2))) %>%
 ungroup %>%
 pivot_wider(names_from = sex, values_from = result:sd)
 dths_stroke_sex_global <- bind_cols(dths_stroke_sex_global, envelope_stroke_sex_global) %>%
 rowwise() %>%
 transmute(result_lwr_f = whoilo_paf_summary(result_f, result_f1, sd_f, sd_f1, CI = 0.025),
 result_upr_f = whoilo_paf_summary(result_f, result_f1, sd_f, sd_f1, CI = 0.975),
 result_f = whoilo_paf_summary(result_f, result_f1, CI = NULL),
 result_lwr_m = whoilo_paf_summary(result_m, result_m1, sd_m, sd_m1, CI = 0.025),
 result_upr_m = whoilo_paf_summary(result_m, result_m1, sd_m, sd_m1, CI = 0.975),
 result_m = whoilo_paf_summary(result_m, result_m1, CI = NULL)) %>%
 ungroup() %>%
 mutate(sd_f = (result_upr_f - result_lwr_f)/2/1.96,
 sd_m = (result_upr_m - result_lwr_m)/2/1.96) %>%
 select(result_f, sd_f, result_lwr_f, result_upr_f, result_m, sd_m, result_lwr_m, result_upr_m)
 }
 if (outcome %in% c("DALYpaf")){
 envelope_stroke_sex_global <- df_in %>%
 filter(outcome == "DALYenvelope",
 year == !!year,
 cause == !!cause) %>%
 mutate(result_lwr = case_when(is.na(result_lwr) ~ result,
 TRUE ~ result_lwr),
 result_upr = case_when(is.na(result_upr) ~ result,
 TRUE ~ result_upr)) %>%
 arrange(region, country_abbrev, year, sex, ilo_age_bands_5yr) %>%
 drop_na() %>%
 mutate(sd = (result_upr - result_lwr)/2/1.96) %>%
 group_by(sex) %>%
 summarise(result = sum(result),
 sd = sqrt(sum((sd)^2))) %>%
 ungroup %>%
 pivot_wider(names_from = sex, values_from = result:sd) %>%

 select_all(.funs = funs(str_c(., "1")))

 dths_stroke_sex_global <- df_in %>%
 filter(outcome == "DALY",
 year == !!year,
 cause == !!cause) %>%
 arrange(region, country_abbrev, year, sex, ilo_age_bands_5yr) %>%
 drop_na(region, result) %>%
 mutate(sd = (result_upr - result_lwr)/2/1.96) %>%
 group_by(sex) %>%
 summarise(result = sum(result),
 sd = sqrt(sum((sd)^2))) %>%
 ungroup %>%
 pivot_wider(names_from = sex, values_from = result:sd)
 dths_stroke_sex_global <- bind_cols(dths_stroke_sex_global, envelope_stroke_sex_global) %>%
 rowwise() %>%
 transmute(result_lwr_f = whoilo_paf_summary(result_f, result_f1, sd_f, sd_f1, CI = 0.025),
 result_upr_f = whoilo_paf_summary(result_f, result_f1, sd_f, sd_f1, CI = 0.975),
 result_f = whoilo_paf_summary(result_f, result_f1, CI = NULL),
 result_lwr_m = whoilo_paf_summary(result_m, result_m1, sd_m, sd_m1, CI = 0.025),
 result_upr_m = whoilo_paf_summary(result_m, result_m1, sd_m, sd_m1, CI = 0.975),
 result_m = whoilo_paf_summary(result_m, result_m1, CI = NULL)) %>%
 ungroup() %>%
 mutate(sd_f = (result_upr_f - result_lwr_f)/2/1.96,
 sd_m = (result_upr_m - result_lwr_m)/2/1.96) %>%
 select(result_f, sd_f, result_lwr_f, result_upr_f, result_m, sd_m, result_lwr_m, result_upr_m)
 }
 if (outcome %in% c("h0", "h1", "h2", "h3", "h4", "h5")){
 dths_stroke_sex_global <- df_in %>%
 filter(outcome == !!outcome,
 year == !!year,
 cause == !!cause) %>%
 arrange(region, country_abbrev, year, sex, ilo_age_bands_5yr) %>%
 mutate(sd = (result_upr - result_lwr)/2/1.96) %>%
 group_by(sex) %>%
 left_join(who_ilo_long_populations, by = c("region", "country_abbrev", "year", "sex", "ilo_age_bands_5yr" = "age_bands_5yr")) %>%
 mutate(result = result*population,
 sd = sd*population) %>%
 drop_na() %>%
 summarise(result = sum(result),
 sd = sqrt(sum((sd)^2)),
 population = sum(population)) %>%
 mutate(result_lwr = qnorm(p = 0.025, mean = result, sd = sd)/ population,
 result_upr = qnorm(p = 0.975, mean = result, sd = sd)/ population,
 result = result / population,
 sd = sd/population) %>%
 select(-population) %>%
 ungroup
 if (shape == "wide") {
 dths_stroke_sex_global <- dths_stroke_sex_global %>%
 pivot_wider(names_from = sex, values_from = result:result_upr) %>%
 select(contains("_f"), contains("_m"))
 }
 }
 if (outcome %in% c("h0_number", "h1_number", "h2_number", "h3_number", "h4_number", "h5_number")){
 outcome2 <- str_sub(outcome, end = -8L)
 dths_stroke_sex_global <- df_in %>%
 filter(outcome == !!outcome2,
 year == !!year,
 cause == !!cause) %>%
 arrange(region, country_abbrev, year, sex, ilo_age_bands_5yr) %>%
 mutate(sd = (result_upr - result_lwr)/2/1.96) %>%
 group_by(sex) %>%
 left_join(who_ilo_long_populations, by = c("region", "country_abbrev", "year", "sex", "ilo_age_bands_5yr" = "age_bands_5yr")) %>%
 mutate(population = population * 1000) %>%
 mutate(result = result*population,
 sd = sd*population) %>%
 drop_na() %>%
 summarise(result = sum(result),
 sd = sqrt(sum((sd)^2)),
 population = sum(population)) %>%
 mutate(result_lwr = qnorm(p = 0.025, mean = result, sd = sd),
 result_upr = qnorm(p = 0.975, mean = result, sd = sd),
 result = result,
 sd = sd) %>%
 select(-population) %>%
 ungroup
 if (shape == "wide") {
 dths_stroke_sex_global <- dths_stroke_sex_global %>%
 pivot_wider(names_from = sex, values_from = result:result_upr) %>%
 select(contains("_f"), contains("_m"))
 }
 }
 if (outcome %in% c("DTH", "DALY", "DTHenvelope", "DALYenvelope")) {
 dths_stroke_sex_global <- df_in %>%
 filter(outcome == !!outcome,
 year == !!year,
 cause == !!cause) %>%
 arrange(region, country_abbrev, year, sex, ilo_age_bands_5yr) %>%
 mutate(sd = (result_upr - result_lwr)/2/1.96) %>%
 drop_na(region, result) %>%
 group_by(sex) %>%
 summarise(result = sum(result),
 sd = sqrt(sum((sd)^2))) %>%
 mutate(result_lwr = qnorm(p = 0.025, mean = result, sd = sd),
 result_upr = qnorm(p = 0.975, mean = result, sd = sd)) %>%
 ungroup
 if (shape == "wide") {
 dths_stroke_sex_global <- dths_stroke_sex_global %>%
 pivot_wider(names_from = sex, values_from = result:result_upr) %>%
 select(contains("_f"), contains("_m"))
 }
 }
 if (outcome %in% c("DTHS", "DALYS")) {
 outcome2 <- str_sub(outcome, end = -2L)
 dths_stroke_sex_global <- df_in %>%
 filter(outcome == !!outcome2,
 year == !!year,
 cause %in% c("stroke_h5", "ihd_h5")) %>%
 arrange(region, country_abbrev, year, sex, ilo_age_bands_5yr) %>%
 mutate(sd = (result_upr - result_lwr)/2/1.96) %>%
 drop_na() %>%
 group_by(sex) %>%
 summarise(result = sum(result),
 sd = sqrt(sum((sd)^2))) %>%
 mutate(result_lwr = qnorm(p = 0.025, mean = result, sd = sd),
 result_upr = qnorm(p = 0.975, mean = result, sd = sd)) %>%
 ungroup
 if (shape == "wide") {
 dths_stroke_sex_global <- dths_stroke_sex_global %>%
 pivot_wider(names_from = sex, values_from = result:result_upr) %>%
 select(contains("_f"), contains("_m"))
 }
 }
 if (outcome %in% c("h45")){
 dths_stroke_sex_global <- df_in %>%
 filter(outcome %in% c("h4", "h5"),
 year == !!year,
 cause == !!cause) %>%
 arrange(region, country_abbrev, year, sex, ilo_age_bands_5yr) %>%
 mutate(sd = (result_upr - result_lwr)/2/1.96) %>%
 group_by(sex) %>%
 left_join(who_ilo_long_populations, by = c("region", "country_abbrev", "year", "sex", "ilo_age_bands_5yr" = "age_bands_5yr")) %>%
 mutate(result = result*population,
 sd = sd*population) %>%
 drop_na() %>%
 summarise(result = sum(result),
 sd = sqrt(sum((sd)^2)),
 population = sum(population)/2) %>%
 mutate(result_lwr = qnorm(p = 0.025, mean = result, sd = sd)/ population,
 result_upr = qnorm(p = 0.975, mean = result, sd = sd)/ population,
 result = result / population,
 sd = sd/population) %>%
 select(-population) %>%
 ungroup
 if (shape == "wide") {
 dths_stroke_sex_global <- dths_stroke_sex_global %>%
 pivot_wider(names_from = sex, values_from = result:result_upr) %>%
 select(contains("_f"), contains("_m"))
 }
 }
 if (outcome %in% c("DTHrate")){
 outcome2 <- "DTH"
 dths_stroke_sex_global <- df_in %>%
 filter(outcome == !!outcome2,
 year == !!year,
 cause == !!cause) %>%
 arrange(region, country_abbrev, year, sex, ilo_age_bands_5yr) %>%
 mutate(sd = (result_upr - result_lwr)/2/1.96) %>%
 group_by(sex) %>%
 left_join(who_ilo_long_populations, by = c("region", "country_abbrev", "year", "sex", "ilo_age_bands_5yr" = "age_bands_5yr")) %>%
 drop_na() %>%
 summarise(result = sum(result),
 sd = sqrt(sum((sd)^2)),
 population = sum(population)) %>%
 mutate(population = population / 100,
 result = result / population,
 sd = sd / population,
 result_lwr = qnorm(p = 0.025, mean = result, sd = sd),
 result_upr = qnorm(p = 0.975, mean = result, sd = sd)) %>%
 select(-population) %>%
 ungroup
 if (shape == "wide") {
 dths_stroke_sex_global <- dths_stroke_sex_global %>%
 pivot_wider(names_from = sex, values_from = result:result_upr) %>%
 select(contains("_f"), contains("_m"))
 }
 }
 if (outcome %in% c("DALYrate")){
 outcome2 <- "DALY"
 dths_stroke_sex_global <- df_in %>%
 filter(outcome == !!outcome2,
 year == !!year,
 cause == !!cause) %>%
 arrange(region, country_abbrev, year, sex, ilo_age_bands_5yr) %>%
 mutate(sd = (result_upr - result_lwr)/2/1.96) %>%
 group_by(sex) %>%
 left_join(who_ilo_long_populations, by = c("region", "country_abbrev", "year", "sex", "ilo_age_bands_5yr" = "age_bands_5yr")) %>%
 drop_na() %>%
 summarise(result = sum(result),
 sd = sqrt(sum((sd)^2)),
 population = sum(population)) %>%
 mutate(population = population,
 result = result / population * 100000,
 sd = sd / population * 100000,
 result_lwr = qnorm(p = 0.025, mean = result, sd = sd),
 result_upr = qnorm(p = 0.975, mean = result, sd = sd)) %>%
 select(-population) %>%
 ungroup
 if (shape == "wide") {
 dths_stroke_sex_global <- dths_stroke_sex_global %>%
 pivot_wider(names_from = sex, values_from = result:result_upr) %>%
 select(contains("_f"), contains("_m"))
 }
 }
 if (outcome %in% c("population")){
 dths_stroke_sex_global <- who_ilo_long_populations %>%
 filter(age_bands_5yr %in% c("15_19", "20_24", "25_29", "30_34", "35_39", "40_44", "45_49", "50_54", "55_59", "60_64", "65_69", "70_74", "75_79", "80_84", "85_89", "90_94", "95_"),
 year == !!year) %>%
 arrange(region, country_abbrev, year, sex, age_bands_5yr) %>%
 drop_na() %>%
 group_by(sex) %>%
 summarise(result = sum(population)) %>%
 mutate(sd = NA,
 result_lwr = NA,
 result_upr = NA) %>%
 ungroup
 if (shape == "wide") {
 dths_stroke_sex_global <- dths_stroke_sex_global %>%
 pivot_wider(names_from = sex, values_from = result:result_upr) %>%
 select(contains("_f"), contains("_m"))
 }
 }
 if (outcome %in% c("DTHratio")){
 envelope_stroke_sex_global <- df_in %>%
 filter(outcome == "DTHparent",
 year == !!year,
 cause == !!cause) %>%
 arrange(region, country_abbrev, year, sex, ilo_age_bands_5yr) %>%
 drop_na() %>%
 mutate(sd = (result_upr - result_lwr)/2/1.96) %>%
 group_by(sex) %>%
 summarise(result = sum(result),
 sd = sqrt(sum((sd)^2))) %>%
 ungroup %>%
 pivot_wider(names_from = sex, values_from = result:sd)

 dths_stroke_sex_global <- df_in %>%
 filter(outcome == "DTHenvelope",
 year == !!year,
 cause == !!cause) %>%
 arrange(region, country_abbrev, year, sex, ilo_age_bands_5yr) %>%
 drop_na() %>%
 mutate(sd = (result_upr - result_lwr)/2/1.96) %>%
 group_by(sex) %>%
 summarise(result = sum(result),
 sd = sqrt(sum((sd)^2))) %>%
 ungroup %>%
 pivot_wider(names_from = sex, values_from = result:sd) %>%

 select_all(.funs = funs(str_c(., "1")))

 dths_stroke_sex_global <- bind_cols(dths_stroke_sex_global, envelope_stroke_sex_global) %>%
 rowwise() %>%
 transmute(result_lwr_f = whoilo_paf_summary(result_f, result_f1, sd_f, sd_f1, CI = 0.025),
 result_upr_f = whoilo_paf_summary(result_f, result_f1, sd_f, sd_f1, CI = 0.975),
 result_f = whoilo_paf_summary(result_f, result_f1, CI = NULL),
 result_lwr_m = whoilo_paf_summary(result_m, result_m1, sd_m, sd_m1, CI = 0.025),
 result_upr_m = whoilo_paf_summary(result_m, result_m1, sd_m, sd_m1, CI = 0.975),
 result_m = whoilo_paf_summary(result_m, result_m1, CI = NULL)) %>%
 ungroup() %>%
 mutate(sd_f = (result_upr_f - result_lwr_f)/2/1.96,
 sd_m = (result_upr_m - result_lwr_m)/2/1.96) %>%
 select(result_f, sd_f, result_lwr_f, result_upr_f, result_m, sd_m, result_lwr_m, result_upr_m)
 }
 if (outcome %in% c("DALYratio")){
 envelope_stroke_sex_global <- df_in %>%
 filter(outcome == "DALYparent",
 year == !!year,
 cause == !!cause) %>%
 arrange(region, country_abbrev, year, sex, ilo_age_bands_5yr) %>%
 drop_na() %>%
 mutate(sd = (result_upr - result_lwr)/2/1.96) %>%
 group_by(sex) %>%
 summarise(result = sum(result),
 sd = sqrt(sum((sd)^2))) %>%
 ungroup %>%
 pivot_wider(names_from = sex, values_from = result:sd)

 dths_stroke_sex_global <- df_in %>%
 filter(outcome == "DALYenvelope",
 year == !!year,
 cause == !!cause) %>%
 mutate(result_lwr = case_when(is.na(result_lwr) ~ result,
 TRUE ~ result_lwr),
 result_upr = case_when(is.na(result_upr) ~ result,
 TRUE ~ result_upr)) %>%
 arrange(region, country_abbrev, year, sex, ilo_age_bands_5yr) %>%
 drop_na() %>%
 mutate(sd = (result_upr - result_lwr)/2/1.96) %>%
 group_by(sex) %>%
 summarise(result = sum(result),
 sd = sqrt(sum((sd)^2))) %>%
 ungroup %>%
 pivot_wider(names_from = sex, values_from = result:sd) %>%

 select_all(.funs = funs(str_c(., "1")))

 dths_stroke_sex_global <- bind_cols(dths_stroke_sex_global, envelope_stroke_sex_global) %>%
 rowwise() %>%
 transmute(result_lwr_f = whoilo_paf_summary(result_f, result_f1, sd_f, sd_f1, CI = 0.025),
 result_upr_f = whoilo_paf_summary(result_f, result_f1, sd_f, sd_f1, CI = 0.975),
 result_f = whoilo_paf_summary(result_f, result_f1, CI = NULL),
 result_lwr_m = whoilo_paf_summary(result_m, result_m1, sd_m, sd_m1, CI = 0.025),
 result_upr_m = whoilo_paf_summary(result_m, result_m1, sd_m, sd_m1, CI = 0.975),
 result_m = whoilo_paf_summary(result_m, result_m1, CI = NULL)) %>%
 ungroup() %>%
 mutate(sd_f = (result_upr_f - result_lwr_f)/2/1.96,
 sd_m = (result_upr_m - result_lwr_m)/2/1.96) %>%
 select(result_f, sd_f, result_lwr_f, result_upr_f, result_m, sd_m, result_lwr_m, result_upr_m)
 }
 }
 # by age
 if ("age" %in% breakdown){
 if (outcome %in% c("PAF", "DTHpaf")){
 envelope_stroke_age_global <- df_in %>%
 filter(outcome == "DTHenvelope",
 year == !!year,
 cause == !!cause) %>%
 arrange(region, country_abbrev, year, sex, ilo_age_bands_5yr) %>%
 drop_na() %>%
 mutate(sd = (result_upr - result_lwr)/2/1.96) %>%
 group_by(ilo_age_bands_5yr) %>%
 summarise(result = sum(result),
 sd = sqrt(sum((sd)^2))) %>%
 ungroup %>%
 pivot_wider(names_from = ilo_age_bands_5yr, values_from = result:sd) %>%

 select_all(.funs = funs(str_c(., "1")))

 dths_stroke_age_global <- df_in %>%
 filter(outcome == "DTH",
 year == !!year,
 cause == !!cause) %>%
 arrange(region, country_abbrev, year, sex, ilo_age_bands_5yr) %>%
 drop_na() %>%
 mutate(sd = (result_upr - result_lwr)/2/1.96) %>%
 group_by(ilo_age_bands_5yr) %>%
 summarise(result = sum(result),
 sd = sqrt(sum((sd)^2))) %>%
 ungroup %>%
 pivot_wider(names_from = ilo_age_bands_5yr, values_from = result:sd)
 dths_stroke_age_global <- bind_cols(dths_stroke_age_global, envelope_stroke_age_global) %>%
 rowwise() %>%
 transmute(result_b_15_19 = whoilo_paf_summary(result_15_19, result_15_191, CI = NULL),
 result_lwr_b_15_19 = whoilo_paf_summary(result_15_19, result_15_191, sd_15_19, sd_15_191, CI = 0.025),
 result_upr_b_15_19 = whoilo_paf_summary(result_15_19, result_15_191, sd_15_19, sd_15_191, CI = 0.975),
 result_b_20_24 = whoilo_paf_summary(result_20_24, result_20_241, CI = NULL),
 result_lwr_b_20_24 = whoilo_paf_summary(result_20_24, result_20_241, sd_20_24, sd_20_241, CI = 0.025),
 result_upr_b_20_24 = whoilo_paf_summary(result_20_24, result_20_241, sd_20_24, sd_20_241, CI = 0.975),
 result_b_25_29 = whoilo_paf_summary(result_25_29, result_25_291, CI = NULL),
 result_lwr_b_25_29 = whoilo_paf_summary(result_25_29, result_25_291, sd_25_29, sd_25_291, CI = 0.025),
 result_upr_b_25_29 = whoilo_paf_summary(result_25_29, result_25_291, sd_25_29, sd_25_291, CI = 0.975),
 result_b_30_34 = whoilo_paf_summary(result_30_34, result_30_341, CI = NULL),
 result_lwr_b_30_34 = whoilo_paf_summary(result_30_34, result_30_341, sd_30_34, sd_30_341, CI = 0.025),
 result_upr_b_30_34 = whoilo_paf_summary(result_30_34, result_30_341, sd_30_34, sd_30_341, CI = 0.975),
 result_b_35_39 = whoilo_paf_summary(result_35_39, result_35_391, CI = NULL),
 result_lwr_b_35_39 = whoilo_paf_summary(result_35_39, result_35_391, sd_35_39, sd_35_391, CI = 0.025),
 result_upr_b_35_39 = whoilo_paf_summary(result_35_39, result_35_391, sd_35_39, sd_35_391, CI = 0.975),
 result_b_40_44 = whoilo_paf_summary(result_40_44, result_40_441, CI = NULL),
 result_lwr_b_40_44 = whoilo_paf_summary(result_40_44, result_40_441, sd_40_44, sd_40_441, CI = 0.025),
 result_upr_b_40_44 = whoilo_paf_summary(result_40_44, result_40_441, sd_40_44, sd_40_441, CI = 0.975),
 result_b_45_49 = whoilo_paf_summary(result_45_49, result_45_491, CI = NULL),
 result_lwr_b_45_49 = whoilo_paf_summary(result_45_49, result_45_491, sd_45_49, sd_45_491, CI = 0.025),
 result_upr_b_45_49 = whoilo_paf_summary(result_45_49, result_45_491, sd_45_49, sd_45_491, CI = 0.975),
 result_b_50_54 = whoilo_paf_summary(result_50_54, result_50_541, CI = NULL),
 result_lwr_b_50_54 = whoilo_paf_summary(result_50_54, result_50_541, sd_50_54, sd_50_541, CI = 0.025),
 result_upr_b_50_54 = whoilo_paf_summary(result_50_54, result_50_541, sd_50_54, sd_50_541, CI = 0.975),
 result_b_55_59 = whoilo_paf_summary(result_55_59, result_55_591, CI = NULL),
 result_lwr_b_55_59 = whoilo_paf_summary(result_55_59, result_55_591, sd_55_59, sd_55_591, CI = 0.025),
 result_upr_b_55_59 = whoilo_paf_summary(result_55_59, result_55_591, sd_55_59, sd_55_591, CI = 0.975),
 result_b_60_64 = whoilo_paf_summary(result_60_64, result_60_641, CI = NULL),
 result_lwr_b_60_64 = whoilo_paf_summary(result_60_64, result_60_641, sd_60_64, sd_60_641, CI = 0.025),
 result_upr_b_60_64 = whoilo_paf_summary(result_60_64, result_60_641, sd_60_64, sd_60_641, CI = 0.975),
 result_b_65_69 = whoilo_paf_summary(result_65_69, result_65_691, CI = NULL),
 result_lwr_b_65_69 = whoilo_paf_summary(result_65_69, result_65_691, sd_65_69, sd_65_691, CI = 0.025),
 result_upr_b_65_69 = whoilo_paf_summary(result_65_69, result_65_691, sd_65_69, sd_65_691, CI = 0.975),
 result_b_70_74 = whoilo_paf_summary(result_70_74, result_70_741, CI = NULL),
 result_lwr_b_70_74 = whoilo_paf_summary(result_70_74, result_70_741, sd_70_74, sd_70_741, CI = 0.025),
 result_upr_b_70_74 = whoilo_paf_summary(result_70_74, result_70_741, sd_70_74, sd_70_741, CI = 0.975),
 result_b_75_79 = whoilo_paf_summary(result_75_79, result_75_791, CI = NULL),
 result_lwr_b_75_79 = whoilo_paf_summary(result_75_79, result_75_791, sd_75_79, sd_75_791, CI = 0.025),
 result_upr_b_75_79 = whoilo_paf_summary(result_75_79, result_75_791, sd_75_79, sd_75_791, CI = 0.975),
 result_b_80_84 = whoilo_paf_summary(result_80_84, result_80_841, CI = NULL),
 result_lwr_b_80_84 = whoilo_paf_summary(result_80_84, result_80_841, sd_80_84, sd_80_841, CI = 0.025),
 result_upr_b_80_84 = whoilo_paf_summary(result_80_84, result_80_841, sd_80_84, sd_80_841, CI = 0.975),
 result_b_85_89 = whoilo_paf_summary(result_85_89, result_85_891, CI = NULL),
 result_lwr_b_85_89 = whoilo_paf_summary(result_85_89, result_85_891, sd_85_89, sd_85_891, CI = 0.025),
 result_upr_b_85_89 = whoilo_paf_summary(result_85_89, result_85_891, sd_85_89, sd_85_891, CI = 0.975),
 result_b_90_94 = whoilo_paf_summary(result_90_94, result_90_941, CI = NULL),
 result_lwr_b_90_94 = whoilo_paf_summary(result_90_94, result_90_941, sd_90_94, sd_90_941, CI = 0.025),
 result_upr_b_90_94 = whoilo_paf_summary(result_90_94, result_90_941, sd_90_94, sd_90_941, CI = 0.975),
 result_b_95_ = whoilo_paf_summary(result_95_, result_95_1, CI = NULL),
 result_lwr_b_95_ = whoilo_paf_summary(result_95_, result_95_1, sd_95_, sd_95_1, CI = 0.025),
 result_upr_b_95_ = whoilo_paf_summary(result_95_, result_95_1, sd_95_, sd_95_1, CI = 0.975)) %>%
 ungroup() %>%
 mutate(sd_b_15_19 = (result_upr_b_15_19 - result_lwr_b_15_19)/2/1.96,
 sd_b_20_24 = (result_upr_b_20_24 - result_lwr_b_20_24)/2/1.96,
 sd_b_25_29 = (result_upr_b_25_29 - result_lwr_b_25_29)/2/1.96,
 sd_b_30_34 = (result_upr_b_30_34 - result_lwr_b_30_34)/2/1.96,
 sd_b_35_39 = (result_upr_b_35_39 - result_lwr_b_35_39)/2/1.96,
 sd_b_40_44 = (result_upr_b_40_44 - result_lwr_b_40_44)/2/1.96,
 sd_b_45_49 = (result_upr_b_45_49 - result_lwr_b_45_49)/2/1.96,
 sd_b_50_54 = (result_upr_b_50_54 - result_lwr_b_50_54)/2/1.96,
 sd_b_55_59 = (result_upr_b_55_59 - result_lwr_b_55_59)/2/1.96,
 sd_b_60_64 = (result_upr_b_60_64 - result_lwr_b_60_64)/2/1.96,
 sd_b_65_69 = (result_upr_b_65_69 - result_lwr_b_65_69)/2/1.96,
 sd_b_70_74 = (result_upr_b_70_74 - result_lwr_b_70_74)/2/1.96,
 sd_b_75_79 = (result_upr_b_75_79 - result_lwr_b_75_79)/2/1.96,
 sd_b_80_84 = (result_upr_b_80_84 - result_lwr_b_80_84)/2/1.96,
 sd_b_85_89 = (result_upr_b_85_89 - result_lwr_b_85_89)/2/1.96,
 sd_b_90_94 = (result_upr_b_90_94 - result_lwr_b_90_94)/2/1.96,
 sd_b_95_ = (result_upr_b_95_ - result_lwr_b_95_)/2/1.96) %>%
 select(result_b_15_19, sd_b_15_19, result_lwr_b_15_19, result_upr_b_15_19,
 result_b_20_24, sd_b_20_24, result_lwr_b_20_24, result_upr_b_20_24,
 result_b_25_29, sd_b_25_29, result_lwr_b_25_29, result_upr_b_25_29,
 result_b_30_34, sd_b_30_34, result_lwr_b_30_34, result_upr_b_30_34,
 result_b_35_39, sd_b_35_39, result_lwr_b_35_39, result_upr_b_35_39,
 result_b_40_44, sd_b_40_44, result_lwr_b_40_44, result_upr_b_40_44,
 result_b_45_49, sd_b_45_49, result_lwr_b_45_49, result_upr_b_45_49,
 result_b_50_54, sd_b_50_54, result_lwr_b_50_54, result_upr_b_50_54,
 result_b_55_59, sd_b_55_59, result_lwr_b_55_59, result_upr_b_55_59,
 result_b_60_64, sd_b_60_64, result_lwr_b_60_64, result_upr_b_60_64,
 result_b_65_69, sd_b_65_69, result_lwr_b_65_69, result_upr_b_65_69,
 result_b_70_74, sd_b_70_74, result_lwr_b_70_74, result_upr_b_70_74,
 result_b_75_79, sd_b_75_79, result_lwr_b_75_79, result_upr_b_75_79,
 result_b_80_84, sd_b_80_84, result_lwr_b_80_84, result_upr_b_80_84,
 result_b_85_89, sd_b_85_89, result_lwr_b_85_89, result_upr_b_85_89,
 result_b_90_94, sd_b_90_94, result_lwr_b_90_94, result_upr_b_90_94,
 result_b_95_, sd_b_95_, result_lwr_b_95_, result_upr_b_95_)
 }
 if (outcome %in% c("DALYpaf")){
 envelope_stroke_age_global <- df_in %>%
 filter(outcome == "DALYenvelope",
 year == !!year,
 cause == !!cause) %>%
 mutate(result_lwr = case_when(is.na(result_lwr) ~ result,
 TRUE ~ result_lwr),
 result_upr = case_when(is.na(result_upr) ~ result,
 TRUE ~ result_upr)) %>%
 arrange(region, country_abbrev, year, sex, ilo_age_bands_5yr) %>%
 drop_na() %>%
 mutate(sd = (result_upr - result_lwr)/2/1.96) %>%
 group_by(ilo_age_bands_5yr) %>%
 summarise(result = sum(result),
 sd = sqrt(sum((sd)^2))) %>%
 ungroup %>%
 pivot_wider(names_from = ilo_age_bands_5yr, values_from = result:sd) %>%

 select_all(.funs = funs(str_c(., "1")))

 dths_stroke_age_global <- df_in %>%
 filter(outcome == "DALY",
 year == !!year,
 cause == !!cause) %>%
 arrange(region, country_abbrev, year, sex, ilo_age_bands_5yr) %>%
 drop_na() %>%
 mutate(sd = (result_upr - result_lwr)/2/1.96) %>%
 group_by(ilo_age_bands_5yr) %>%
 summarise(result = sum(result),
 sd = sqrt(sum((sd)^2))) %>%
 ungroup %>%
 pivot_wider(names_from = ilo_age_bands_5yr, values_from = result:sd)
 dths_stroke_age_global <- bind_cols(dths_stroke_age_global, envelope_stroke_age_global) %>%
 rowwise() %>%
 transmute(result_b_15_19 = whoilo_paf_summary(result_15_19, result_15_191, CI = NULL),
 result_lwr_b_15_19 = whoilo_paf_summary(result_15_19, result_15_191, sd_15_19, sd_15_191, CI = 0.025),
 result_upr_b_15_19 = whoilo_paf_summary(result_15_19, result_15_191, sd_15_19, sd_15_191, CI = 0.975),
 result_b_20_24 = whoilo_paf_summary(result_20_24, result_20_241, CI = NULL),
 result_lwr_b_20_24 = whoilo_paf_summary(result_20_24, result_20_241, sd_20_24, sd_20_241, CI = 0.025),
 result_upr_b_20_24 = whoilo_paf_summary(result_20_24, result_20_241, sd_20_24, sd_20_241, CI = 0.975),
 result_b_25_29 = whoilo_paf_summary(result_25_29, result_25_291, CI = NULL),
 result_lwr_b_25_29 = whoilo_paf_summary(result_25_29, result_25_291, sd_25_29, sd_25_291, CI = 0.025),
 result_upr_b_25_29 = whoilo_paf_summary(result_25_29, result_25_291, sd_25_29, sd_25_291, CI = 0.975),
 result_b_30_34 = whoilo_paf_summary(result_30_34, result_30_341, CI = NULL),
 result_lwr_b_30_34 = whoilo_paf_summary(result_30_34, result_30_341, sd_30_34, sd_30_341, CI = 0.025),
 result_upr_b_30_34 = whoilo_paf_summary(result_30_34, result_30_341, sd_30_34, sd_30_341, CI = 0.975),
 result_b_35_39 = whoilo_paf_summary(result_35_39, result_35_391, CI = NULL),
 result_lwr_b_35_39 = whoilo_paf_summary(result_35_39, result_35_391, sd_35_39, sd_35_391, CI = 0.025),
 result_upr_b_35_39 = whoilo_paf_summary(result_35_39, result_35_391, sd_35_39, sd_35_391, CI = 0.975),
 result_b_40_44 = whoilo_paf_summary(result_40_44, result_40_441, CI = NULL),
 result_lwr_b_40_44 = whoilo_paf_summary(result_40_44, result_40_441, sd_40_44, sd_40_441, CI = 0.025),
 result_upr_b_40_44 = whoilo_paf_summary(result_40_44, result_40_441, sd_40_44, sd_40_441, CI = 0.975),
 result_b_45_49 = whoilo_paf_summary(result_45_49, result_45_491, CI = NULL),
 result_lwr_b_45_49 = whoilo_paf_summary(result_45_49, result_45_491, sd_45_49, sd_45_491, CI = 0.025),
 result_upr_b_45_49 = whoilo_paf_summary(result_45_49, result_45_491, sd_45_49, sd_45_491, CI = 0.975),
 result_b_50_54 = whoilo_paf_summary(result_50_54, result_50_541, CI = NULL),
 result_lwr_b_50_54 = whoilo_paf_summary(result_50_54, result_50_541, sd_50_54, sd_50_541, CI = 0.025),
 result_upr_b_50_54 = whoilo_paf_summary(result_50_54, result_50_541, sd_50_54, sd_50_541, CI = 0.975),
 result_b_55_59 = whoilo_paf_summary(result_55_59, result_55_591, CI = NULL),
 result_lwr_b_55_59 = whoilo_paf_summary(result_55_59, result_55_591, sd_55_59, sd_55_591, CI = 0.025),
 result_upr_b_55_59 = whoilo_paf_summary(result_55_59, result_55_591, sd_55_59, sd_55_591, CI = 0.975),
 result_b_60_64 = whoilo_paf_summary(result_60_64, result_60_641, CI = NULL),
 result_lwr_b_60_64 = whoilo_paf_summary(result_60_64, result_60_641, sd_60_64, sd_60_641, CI = 0.025),
 result_upr_b_60_64 = whoilo_paf_summary(result_60_64, result_60_641, sd_60_64, sd_60_641, CI = 0.975),
 result_b_65_69 = whoilo_paf_summary(result_65_69, result_65_691, CI = NULL),
 result_lwr_b_65_69 = whoilo_paf_summary(result_65_69, result_65_691, sd_65_69, sd_65_691, CI = 0.025),
 result_upr_b_65_69 = whoilo_paf_summary(result_65_69, result_65_691, sd_65_69, sd_65_691, CI = 0.975),
 result_b_70_74 = whoilo_paf_summary(result_70_74, result_70_741, CI = NULL),
 result_lwr_b_70_74 = whoilo_paf_summary(result_70_74, result_70_741, sd_70_74, sd_70_741, CI = 0.025),
 result_upr_b_70_74 = whoilo_paf_summary(result_70_74, result_70_741, sd_70_74, sd_70_741, CI = 0.975),
 result_b_75_79 = whoilo_paf_summary(result_75_79, result_75_791, CI = NULL),
 result_lwr_b_75_79 = whoilo_paf_summary(result_75_79, result_75_791, sd_75_79, sd_75_791, CI = 0.025),
 result_upr_b_75_79 = whoilo_paf_summary(result_75_79, result_75_791, sd_75_79, sd_75_791, CI = 0.975),
 result_b_80_84 = whoilo_paf_summary(result_80_84, result_80_841, CI = NULL),
 result_lwr_b_80_84 = whoilo_paf_summary(result_80_84, result_80_841, sd_80_84, sd_80_841, CI = 0.025),
 result_upr_b_80_84 = whoilo_paf_summary(result_80_84, result_80_841, sd_80_84, sd_80_841, CI = 0.975),
 result_b_85_89 = whoilo_paf_summary(result_85_89, result_85_891, CI = NULL),
 result_lwr_b_85_89 = whoilo_paf_summary(result_85_89, result_85_891, sd_85_89, sd_85_891, CI = 0.025),
 result_upr_b_85_89 = whoilo_paf_summary(result_85_89, result_85_891, sd_85_89, sd_85_891, CI = 0.975),
 result_b_90_94 = whoilo_paf_summary(result_90_94, result_90_941, CI = NULL),
 result_lwr_b_90_94 = whoilo_paf_summary(result_90_94, result_90_941, sd_90_94, sd_90_941, CI = 0.025),
 result_upr_b_90_94 = whoilo_paf_summary(result_90_94, result_90_941, sd_90_94, sd_90_941, CI = 0.975),
 result_b_95_ = whoilo_paf_summary(result_95_, result_95_1, CI = NULL),
 result_lwr_b_95_ = whoilo_paf_summary(result_95_, result_95_1, sd_95_, sd_95_1, CI = 0.025),
 result_upr_b_95_ = whoilo_paf_summary(result_95_, result_95_1, sd_95_, sd_95_1, CI = 0.975)) %>%
 ungroup() %>%
 mutate(sd_b_15_19 = (result_upr_b_15_19 - result_lwr_b_15_19)/2/1.96,
 sd_b_20_24 = (result_upr_b_20_24 - result_lwr_b_20_24)/2/1.96,
 sd_b_25_29 = (result_upr_b_25_29 - result_lwr_b_25_29)/2/1.96,
 sd_b_30_34 = (result_upr_b_30_34 - result_lwr_b_30_34)/2/1.96,
 sd_b_35_39 = (result_upr_b_35_39 - result_lwr_b_35_39)/2/1.96,
 sd_b_40_44 = (result_upr_b_40_44 - result_lwr_b_40_44)/2/1.96,
 sd_b_45_49 = (result_upr_b_45_49 - result_lwr_b_45_49)/2/1.96,
 sd_b_50_54 = (result_upr_b_50_54 - result_lwr_b_50_54)/2/1.96,
 sd_b_55_59 = (result_upr_b_55_59 - result_lwr_b_55_59)/2/1.96,
 sd_b_60_64 = (result_upr_b_60_64 - result_lwr_b_60_64)/2/1.96,
 sd_b_65_69 = (result_upr_b_65_69 - result_lwr_b_65_69)/2/1.96,
 sd_b_70_74 = (result_upr_b_70_74 - result_lwr_b_70_74)/2/1.96,
 sd_b_75_79 = (result_upr_b_75_79 - result_lwr_b_75_79)/2/1.96,
 sd_b_80_84 = (result_upr_b_80_84 - result_lwr_b_80_84)/2/1.96,
 sd_b_85_89 = (result_upr_b_85_89 - result_lwr_b_85_89)/2/1.96,
 sd_b_90_94 = (result_upr_b_90_94 - result_lwr_b_90_94)/2/1.96,
 sd_b_95_ = (result_upr_b_95_ - result_lwr_b_95_)/2/1.96) %>%
 select(result_b_15_19, sd_b_15_19, result_lwr_b_15_19, result_upr_b_15_19,
 result_b_20_24, sd_b_20_24, result_lwr_b_20_24, result_upr_b_20_24,
 result_b_25_29, sd_b_25_29, result_lwr_b_25_29, result_upr_b_25_29,
 result_b_30_34, sd_b_30_34, result_lwr_b_30_34, result_upr_b_30_34,
 result_b_35_39, sd_b_35_39, result_lwr_b_35_39, result_upr_b_35_39,
 result_b_40_44, sd_b_40_44, result_lwr_b_40_44, result_upr_b_40_44,
 result_b_45_49, sd_b_45_49, result_lwr_b_45_49, result_upr_b_45_49,
 result_b_50_54, sd_b_50_54, result_lwr_b_50_54, result_upr_b_50_54,
 result_b_55_59, sd_b_55_59, result_lwr_b_55_59, result_upr_b_55_59,
 result_b_60_64, sd_b_60_64, result_lwr_b_60_64, result_upr_b_60_64,
 result_b_65_69, sd_b_65_69, result_lwr_b_65_69, result_upr_b_65_69,
 result_b_70_74, sd_b_70_74, result_lwr_b_70_74, result_upr_b_70_74,
 result_b_75_79, sd_b_75_79, result_lwr_b_75_79, result_upr_b_75_79,
 result_b_80_84, sd_b_80_84, result_lwr_b_80_84, result_upr_b_80_84,
 result_b_85_89, sd_b_85_89, result_lwr_b_85_89, result_upr_b_85_89,
 result_b_90_94, sd_b_90_94, result_lwr_b_90_94, result_upr_b_90_94,
 result_b_95_, sd_b_95_, result_lwr_b_95_, result_upr_b_95_)
 }
 if (outcome %in% c("h0", "h1", "h2", "h3", "h4", "h5")){
 dths_stroke_age_global <- df_in %>%
 filter(outcome == !!outcome,
 year == !!year,
 cause == !!cause) %>%
 arrange(region, country_abbrev, year, sex, ilo_age_bands_5yr) %>%
 mutate(sd = (result_upr - result_lwr)/2/1.96) %>%
 group_by(ilo_age_bands_5yr) %>%
 left_join(who_ilo_long_populations, by = c("region", "country_abbrev", "year", "sex", "ilo_age_bands_5yr" = "age_bands_5yr")) %>%
 mutate(result = result*population,
 sd = sd*population) %>%
 drop_na() %>%
 summarise(result = sum(result),
 sd = sqrt(sum((sd)^2)),
 population = sum(population)) %>%
 mutate(result_lwr = qnorm(p = 0.025, mean = result, sd = sd)/ population,
 result_upr = qnorm(p = 0.975, mean = result, sd = sd)/ population,
 result = result / population,
 sd = sd/population) %>%
 select(-population) %>%
 ungroup %>%
 pivot_wider(names_from = ilo_age_bands_5yr, values_from = result:result_upr)
 names(dths_stroke_age_global) <- str_replace_all(names(dths_stroke_age_global), pattern = "result_", replacement = "result_b_")
 names(dths_stroke_age_global) <- str_replace_all(names(dths_stroke_age_global), pattern = "sd_", replacement = "sd_b_")
 names(dths_stroke_age_global) <- str_replace_all(names(dths_stroke_age_global), pattern = "result_b_lwr", replacement = "result_lwr_b")
 names(dths_stroke_age_global) <- str_replace_all(names(dths_stroke_age_global), pattern = "result_b_upr", replacement = "result_upr_b")
 dths_stroke_age_global <- dths_stroke_age_global %>%
 select(result_b_15_19, sd_b_15_19, result_lwr_b_15_19, result_upr_b_15_19,
 result_b_20_24, sd_b_20_24, result_lwr_b_20_24, result_upr_b_20_24,
 result_b_25_29, sd_b_25_29, result_lwr_b_25_29, result_upr_b_25_29,
 result_b_30_34, sd_b_30_34, result_lwr_b_30_34, result_upr_b_30_34,
 result_b_35_39, sd_b_35_39, result_lwr_b_35_39, result_upr_b_35_39,
 result_b_40_44, sd_b_40_44, result_lwr_b_40_44, result_upr_b_40_44,
 result_b_45_49, sd_b_45_49, result_lwr_b_45_49, result_upr_b_45_49,
 result_b_50_54, sd_b_50_54, result_lwr_b_50_54, result_upr_b_50_54,
 result_b_55_59, sd_b_55_59, result_lwr_b_55_59, result_upr_b_55_59,
 result_b_60_64, sd_b_60_64, result_lwr_b_60_64, result_upr_b_60_64,
 result_b_65_69, sd_b_65_69, result_lwr_b_65_69, result_upr_b_65_69,
 result_b_70_74, sd_b_70_74, result_lwr_b_70_74, result_upr_b_70_74,
 result_b_75_79, sd_b_75_79, result_lwr_b_75_79, result_upr_b_75_79,
 result_b_80_84, sd_b_80_84, result_lwr_b_80_84, result_upr_b_80_84,
 result_b_85_89, sd_b_85_89, result_lwr_b_85_89, result_upr_b_85_89,
 result_b_90_94, sd_b_90_94, result_lwr_b_90_94, result_upr_b_90_94,
 result_b_95_, sd_b_95_, result_lwr_b_95_, result_upr_b_95_)
 }
 if (outcome %in% c("h0_number", "h1_number", "h2_number", "h3_number", "h4_number", "h5_number")){
 outcome2 <- str_sub(outcome, end = -8L)
 dths_stroke_age_global <- df_in %>%
 filter(outcome == !!outcome2,
 year == !!year,
 cause == !!cause) %>%
 arrange(region, country_abbrev, year, sex, ilo_age_bands_5yr) %>%
 mutate(sd = (result_upr - result_lwr)/2/1.96) %>%
 group_by(ilo_age_bands_5yr) %>%
 left_join(who_ilo_long_populations, by = c("region", "country_abbrev", "year", "sex", "ilo_age_bands_5yr" = "age_bands_5yr")) %>%
 mutate(population = population * 1000) %>%
 mutate(result = result*population,
 sd = sd*population) %>%
 drop_na() %>%
 summarise(result = sum(result),
 sd = sqrt(sum((sd)^2)),
 population = sum(population)) %>%
 mutate(result_lwr = qnorm(p = 0.025, mean = result, sd = sd),
 result_upr = qnorm(p = 0.975, mean = result, sd = sd),
 result = result,
 sd = sd) %>%
 select(-population) %>%
 ungroup %>%
 pivot_wider(names_from = ilo_age_bands_5yr, values_from = result:result_upr)
 names(dths_stroke_age_global) <- str_replace_all(names(dths_stroke_age_global), pattern = "result_", replacement = "result_b_")
 names(dths_stroke_age_global) <- str_replace_all(names(dths_stroke_age_global), pattern = "sd_", replacement = "sd_b_")
 names(dths_stroke_age_global) <- str_replace_all(names(dths_stroke_age_global), pattern = "result_b_lwr", replacement = "result_lwr_b")
 names(dths_stroke_age_global) <- str_replace_all(names(dths_stroke_age_global), pattern = "result_b_upr", replacement = "result_upr_b")
 dths_stroke_age_global <- dths_stroke_age_global %>%
 select(result_b_15_19, sd_b_15_19, result_lwr_b_15_19, result_upr_b_15_19,
 result_b_20_24, sd_b_20_24, result_lwr_b_20_24, result_upr_b_20_24,
 result_b_25_29, sd_b_25_29, result_lwr_b_25_29, result_upr_b_25_29,
 result_b_30_34, sd_b_30_34, result_lwr_b_30_34, result_upr_b_30_34,
 result_b_35_39, sd_b_35_39, result_lwr_b_35_39, result_upr_b_35_39,
 result_b_40_44, sd_b_40_44, result_lwr_b_40_44, result_upr_b_40_44,
 result_b_45_49, sd_b_45_49, result_lwr_b_45_49, result_upr_b_45_49,
 result_b_50_54, sd_b_50_54, result_lwr_b_50_54, result_upr_b_50_54,
 result_b_55_59, sd_b_55_59, result_lwr_b_55_59, result_upr_b_55_59,
 result_b_60_64, sd_b_60_64, result_lwr_b_60_64, result_upr_b_60_64,
 result_b_65_69, sd_b_65_69, result_lwr_b_65_69, result_upr_b_65_69,
 result_b_70_74, sd_b_70_74, result_lwr_b_70_74, result_upr_b_70_74,
 result_b_75_79, sd_b_75_79, result_lwr_b_75_79, result_upr_b_75_79,
 result_b_80_84, sd_b_80_84, result_lwr_b_80_84, result_upr_b_80_84,
 result_b_85_89, sd_b_85_89, result_lwr_b_85_89, result_upr_b_85_89,
 result_b_90_94, sd_b_90_94, result_lwr_b_90_94, result_upr_b_90_94,
 result_b_95_, sd_b_95_, result_lwr_b_95_, result_upr_b_95_)
 }
 if (outcome %in% c("DTH", "DALY", "DTHenvelope", "DALYenvelope")){
 dths_stroke_age_global <- df_in %>%
 filter(outcome == !!outcome,
 year == !!year,
 cause == !!cause) %>%
 arrange(region, country_abbrev, year, sex, ilo_age_bands_5yr) %>%
 mutate(sd = (result_upr - result_lwr)/2/1.96) %>%
 drop_na(region, result) %>%
 group_by(ilo_age_bands_5yr) %>%
 summarise(result = sum(result),
 sd = sqrt(sum((sd)^2))) %>%
 mutate(result_lwr = qnorm(p = 0.025, mean = result, sd = sd),
 result_upr = qnorm(p = 0.975, mean = result, sd = sd)) %>%
 ungroup %>%
 pivot_wider(names_from = ilo_age_bands_5yr, values_from = result:result_upr)
 names(dths_stroke_age_global) <- str_replace_all(names(dths_stroke_age_global), pattern = "result_", replacement = "result_b_")
 names(dths_stroke_age_global) <- str_replace_all(names(dths_stroke_age_global), pattern = "sd_", replacement = "sd_b_")
 names(dths_stroke_age_global) <- str_replace_all(names(dths_stroke_age_global), pattern = "result_b_lwr", replacement = "result_lwr_b")
 names(dths_stroke_age_global) <- str_replace_all(names(dths_stroke_age_global), pattern = "result_b_upr", replacement = "result_upr_b")
 dths_stroke_age_global <- dths_stroke_age_global %>%
 select(result_b_15_19, sd_b_15_19, result_lwr_b_15_19, result_upr_b_15_19,
 result_b_20_24, sd_b_20_24, result_lwr_b_20_24, result_upr_b_20_24,
 result_b_25_29, sd_b_25_29, result_lwr_b_25_29, result_upr_b_25_29,
 result_b_30_34, sd_b_30_34, result_lwr_b_30_34, result_upr_b_30_34,
 result_b_35_39, sd_b_35_39, result_lwr_b_35_39, result_upr_b_35_39,
 result_b_40_44, sd_b_40_44, result_lwr_b_40_44, result_upr_b_40_44,
 result_b_45_49, sd_b_45_49, result_lwr_b_45_49, result_upr_b_45_49,
 result_b_50_54, sd_b_50_54, result_lwr_b_50_54, result_upr_b_50_54,
 result_b_55_59, sd_b_55_59, result_lwr_b_55_59, result_upr_b_55_59,
 result_b_60_64, sd_b_60_64, result_lwr_b_60_64, result_upr_b_60_64,
 result_b_65_69, sd_b_65_69, result_lwr_b_65_69, result_upr_b_65_69,
 result_b_70_74, sd_b_70_74, result_lwr_b_70_74, result_upr_b_70_74,
 result_b_75_79, sd_b_75_79, result_lwr_b_75_79, result_upr_b_75_79,
 result_b_80_84, sd_b_80_84, result_lwr_b_80_84, result_upr_b_80_84,
 result_b_85_89, sd_b_85_89, result_lwr_b_85_89, result_upr_b_85_89,
 result_b_90_94, sd_b_90_94, result_lwr_b_90_94, result_upr_b_90_94,
 result_b_95_, sd_b_95_, result_lwr_b_95_, result_upr_b_95_)
 }
 if (outcome %in% c("DTHS", "DALYS")) {
 outcome2 <- str_sub(outcome, end = -2L)
 dths_stroke_age_global <- df_in %>%
 filter(outcome == !!outcome2,
 year == !!year,
 cause %in% c("stroke_h5", "ihd_h5")) %>%
 arrange(region, country_abbrev, year, sex, ilo_age_bands_5yr) %>%
 mutate(sd = (result_upr - result_lwr)/2/1.96) %>%
 drop_na() %>%
 group_by(ilo_age_bands_5yr) %>%
 summarise(result = sum(result),
 sd = sqrt(sum((sd)^2))) %>%
 mutate(result_lwr = qnorm(p = 0.025, mean = result, sd = sd),
 result_upr = qnorm(p = 0.975, mean = result, sd = sd)) %>%
 ungroup %>%
 pivot_wider(names_from = ilo_age_bands_5yr, values_from = result:result_upr)
 names(dths_stroke_age_global) <- str_replace_all(names(dths_stroke_age_global), pattern = "result_", replacement = "result_b_")
 names(dths_stroke_age_global) <- str_replace_all(names(dths_stroke_age_global), pattern = "sd_", replacement = "sd_b_")
 names(dths_stroke_age_global) <- str_replace_all(names(dths_stroke_age_global), pattern = "result_b_lwr", replacement = "result_lwr_b")
 names(dths_stroke_age_global) <- str_replace_all(names(dths_stroke_age_global), pattern = "result_b_upr", replacement = "result_upr_b")
 dths_stroke_age_global <- dths_stroke_age_global %>%
 select(result_b_15_19, sd_b_15_19, result_lwr_b_15_19, result_upr_b_15_19,
 result_b_20_24, sd_b_20_24, result_lwr_b_20_24, result_upr_b_20_24,
 result_b_25_29, sd_b_25_29, result_lwr_b_25_29, result_upr_b_25_29,
 result_b_30_34, sd_b_30_34, result_lwr_b_30_34, result_upr_b_30_34,
 result_b_35_39, sd_b_35_39, result_lwr_b_35_39, result_upr_b_35_39,
 result_b_40_44, sd_b_40_44, result_lwr_b_40_44, result_upr_b_40_44,
 result_b_45_49, sd_b_45_49, result_lwr_b_45_49, result_upr_b_45_49,
 result_b_50_54, sd_b_50_54, result_lwr_b_50_54, result_upr_b_50_54,
 result_b_55_59, sd_b_55_59, result_lwr_b_55_59, result_upr_b_55_59,
 result_b_60_64, sd_b_60_64, result_lwr_b_60_64, result_upr_b_60_64,
 result_b_65_69, sd_b_65_69, result_lwr_b_65_69, result_upr_b_65_69,
 result_b_70_74, sd_b_70_74, result_lwr_b_70_74, result_upr_b_70_74,
 result_b_75_79, sd_b_75_79, result_lwr_b_75_79, result_upr_b_75_79,
 result_b_80_84, sd_b_80_84, result_lwr_b_80_84, result_upr_b_80_84,
 result_b_85_89, sd_b_85_89, result_lwr_b_85_89, result_upr_b_85_89,
 result_b_90_94, sd_b_90_94, result_lwr_b_90_94, result_upr_b_90_94,
 result_b_95_, sd_b_95_, result_lwr_b_95_, result_upr_b_95_)
 }
 if (outcome %in% c("h45")){
 dths_stroke_age_global <- df_in %>%
 filter(outcome %in% c("h4", "h5"),
 year == !!year,
 cause == !!cause) %>%
 arrange(region, country_abbrev, year, sex, ilo_age_bands_5yr) %>%
 mutate(sd = (result_upr - result_lwr)/2/1.96) %>%
 group_by(ilo_age_bands_5yr) %>%
 left_join(who_ilo_long_populations, by = c("region", "country_abbrev", "year", "sex", "ilo_age_bands_5yr" = "age_bands_5yr")) %>%
 mutate(result = result*population,
 sd = sd*population) %>%
 drop_na() %>%
 summarise(result = sum(result),
 sd = sqrt(sum((sd)^2)),
 population = sum(population)/2) %>%
 mutate(result_lwr = qnorm(p = 0.025, mean = result, sd = sd)/ population,
 result_upr = qnorm(p = 0.975, mean = result, sd = sd)/ population,
 result = result / population,
 sd = sd/population) %>%
 select(-population) %>%
 ungroup %>%
 pivot_wider(names_from = ilo_age_bands_5yr, values_from = result:result_upr)
 names(dths_stroke_age_global) <- str_replace_all(names(dths_stroke_age_global), pattern = "result_", replacement = "result_b_")
 names(dths_stroke_age_global) <- str_replace_all(names(dths_stroke_age_global), pattern = "sd_", replacement = "sd_b_")
 names(dths_stroke_age_global) <- str_replace_all(names(dths_stroke_age_global), pattern = "result_b_lwr", replacement = "result_lwr_b")
 names(dths_stroke_age_global) <- str_replace_all(names(dths_stroke_age_global), pattern = "result_b_upr", replacement = "result_upr_b")
 dths_stroke_age_global <- dths_stroke_age_global %>%
 select(result_b_15_19, sd_b_15_19, result_lwr_b_15_19, result_upr_b_15_19,
 result_b_20_24, sd_b_20_24, result_lwr_b_20_24, result_upr_b_20_24,
 result_b_25_29, sd_b_25_29, result_lwr_b_25_29, result_upr_b_25_29,
 result_b_30_34, sd_b_30_34, result_lwr_b_30_34, result_upr_b_30_34,
 result_b_35_39, sd_b_35_39, result_lwr_b_35_39, result_upr_b_35_39,
 result_b_40_44, sd_b_40_44, result_lwr_b_40_44, result_upr_b_40_44,
 result_b_45_49, sd_b_45_49, result_lwr_b_45_49, result_upr_b_45_49,
 result_b_50_54, sd_b_50_54, result_lwr_b_50_54, result_upr_b_50_54,
 result_b_55_59, sd_b_55_59, result_lwr_b_55_59, result_upr_b_55_59,
 result_b_60_64, sd_b_60_64, result_lwr_b_60_64, result_upr_b_60_64,
 result_b_65_69, sd_b_65_69, result_lwr_b_65_69, result_upr_b_65_69,
 result_b_70_74, sd_b_70_74, result_lwr_b_70_74, result_upr_b_70_74,
 result_b_75_79, sd_b_75_79, result_lwr_b_75_79, result_upr_b_75_79,
 result_b_80_84, sd_b_80_84, result_lwr_b_80_84, result_upr_b_80_84,
 result_b_85_89, sd_b_85_89, result_lwr_b_85_89, result_upr_b_85_89,
 result_b_90_94, sd_b_90_94, result_lwr_b_90_94, result_upr_b_90_94,
 result_b_95_, sd_b_95_, result_lwr_b_95_, result_upr_b_95_)
 }
 if (outcome %in% c("DTHrate")){
 outcome2 <- "DTH"
 dths_stroke_age_global <- df_in %>%
 filter(outcome == !!outcome2,
 year == !!year,
 cause == !!cause) %>%
 arrange(region, country_abbrev, year, sex, ilo_age_bands_5yr) %>%
 mutate(sd = (result_upr - result_lwr)/2/1.96) %>%
 group_by(ilo_age_bands_5yr) %>%
 left_join(who_ilo_long_populations, by = c("region", "country_abbrev", "year", "sex", "ilo_age_bands_5yr" = "age_bands_5yr")) %>%
 drop_na() %>%
 summarise(result = sum(result),
 sd = sqrt(sum((sd)^2)),
 population = sum(population)) %>%
 mutate(population = population / 100,
 result = result / population,
 sd = sd / population,
 result_lwr = qnorm(p = 0.025, mean = result, sd = sd),
 result_upr = qnorm(p = 0.975, mean = result, sd = sd)) %>%
 select(-population) %>%
 ungroup %>%
 pivot_wider(names_from = ilo_age_bands_5yr, values_from = result:result_upr)
 names(dths_stroke_age_global) <- str_replace_all(names(dths_stroke_age_global), pattern = "result_", replacement = "result_b_")
 names(dths_stroke_age_global) <- str_replace_all(names(dths_stroke_age_global), pattern = "sd_", replacement = "sd_b_")
 names(dths_stroke_age_global) <- str_replace_all(names(dths_stroke_age_global), pattern = "result_b_lwr", replacement = "result_lwr_b")
 names(dths_stroke_age_global) <- str_replace_all(names(dths_stroke_age_global), pattern = "result_b_upr", replacement = "result_upr_b")
 dths_stroke_age_global <- dths_stroke_age_global %>%
 select(result_b_15_19, sd_b_15_19, result_lwr_b_15_19, result_upr_b_15_19,
 result_b_20_24, sd_b_20_24, result_lwr_b_20_24, result_upr_b_20_24,
 result_b_25_29, sd_b_25_29, result_lwr_b_25_29, result_upr_b_25_29,
 result_b_30_34, sd_b_30_34, result_lwr_b_30_34, result_upr_b_30_34,
 result_b_35_39, sd_b_35_39, result_lwr_b_35_39, result_upr_b_35_39,
 result_b_40_44, sd_b_40_44, result_lwr_b_40_44, result_upr_b_40_44,
 result_b_45_49, sd_b_45_49, result_lwr_b_45_49, result_upr_b_45_49,
 result_b_50_54, sd_b_50_54, result_lwr_b_50_54, result_upr_b_50_54,
 result_b_55_59, sd_b_55_59, result_lwr_b_55_59, result_upr_b_55_59,
 result_b_60_64, sd_b_60_64, result_lwr_b_60_64, result_upr_b_60_64,
 result_b_65_69, sd_b_65_69, result_lwr_b_65_69, result_upr_b_65_69,
 result_b_70_74, sd_b_70_74, result_lwr_b_70_74, result_upr_b_70_74,
 result_b_75_79, sd_b_75_79, result_lwr_b_75_79, result_upr_b_75_79,
 result_b_80_84, sd_b_80_84, result_lwr_b_80_84, result_upr_b_80_84,
 result_b_85_89, sd_b_85_89, result_lwr_b_85_89, result_upr_b_85_89,
 result_b_90_94, sd_b_90_94, result_lwr_b_90_94, result_upr_b_90_94,
 result_b_95_, sd_b_95_, result_lwr_b_95_, result_upr_b_95_)
 }
 if (outcome %in% c("DALYrate")){
 outcome2 <- "DALY"
 dths_stroke_age_global <- df_in %>%
 filter(outcome == !!outcome2,
 year == !!year,
 cause == !!cause) %>%
 arrange(region, country_abbrev, year, sex, ilo_age_bands_5yr) %>%
 mutate(sd = (result_upr - result_lwr)/2/1.96) %>%
 group_by(ilo_age_bands_5yr) %>%
 left_join(who_ilo_long_populations, by = c("region", "country_abbrev", "year", "sex", "ilo_age_bands_5yr" = "age_bands_5yr")) %>%
 drop_na() %>%
 summarise(result = sum(result),
 sd = sqrt(sum((sd)^2)),
 population = sum(population)) %>%
 mutate(population = population,
 result = result / population * 100000,
 sd = sd / population * 100000,
 result_lwr = qnorm(p = 0.025, mean = result, sd = sd),
 result_upr = qnorm(p = 0.975, mean = result, sd = sd)) %>%
 select(-population) %>%
 ungroup %>%
 pivot_wider(names_from = ilo_age_bands_5yr, values_from = result:result_upr)
 names(dths_stroke_age_global) <- str_replace_all(names(dths_stroke_age_global), pattern = "result_", replacement = "result_b_")
 names(dths_stroke_age_global) <- str_replace_all(names(dths_stroke_age_global), pattern = "sd_", replacement = "sd_b_")
 names(dths_stroke_age_global) <- str_replace_all(names(dths_stroke_age_global), pattern = "result_b_lwr", replacement = "result_lwr_b")
 names(dths_stroke_age_global) <- str_replace_all(names(dths_stroke_age_global), pattern = "result_b_upr", replacement = "result_upr_b")
 dths_stroke_age_global <- dths_stroke_age_global %>%
 select(result_b_15_19, sd_b_15_19, result_lwr_b_15_19, result_upr_b_15_19,
 result_b_20_24, sd_b_20_24, result_lwr_b_20_24, result_upr_b_20_24,
 result_b_25_29, sd_b_25_29, result_lwr_b_25_29, result_upr_b_25_29,
 result_b_30_34, sd_b_30_34, result_lwr_b_30_34, result_upr_b_30_34,
 result_b_35_39, sd_b_35_39, result_lwr_b_35_39, result_upr_b_35_39,
 result_b_40_44, sd_b_40_44, result_lwr_b_40_44, result_upr_b_40_44,
 result_b_45_49, sd_b_45_49, result_lwr_b_45_49, result_upr_b_45_49,
 result_b_50_54, sd_b_50_54, result_lwr_b_50_54, result_upr_b_50_54,
 result_b_55_59, sd_b_55_59, result_lwr_b_55_59, result_upr_b_55_59,
 result_b_60_64, sd_b_60_64, result_lwr_b_60_64, result_upr_b_60_64,
 result_b_65_69, sd_b_65_69, result_lwr_b_65_69, result_upr_b_65_69,
 result_b_70_74, sd_b_70_74, result_lwr_b_70_74, result_upr_b_70_74,
 result_b_75_79, sd_b_75_79, result_lwr_b_75_79, result_upr_b_75_79,
 result_b_80_84, sd_b_80_84, result_lwr_b_80_84, result_upr_b_80_84,
 result_b_85_89, sd_b_85_89, result_lwr_b_85_89, result_upr_b_85_89,
 result_b_90_94, sd_b_90_94, result_lwr_b_90_94, result_upr_b_90_94,
 result_b_95_, sd_b_95_, result_lwr_b_95_, result_upr_b_95_)
 }
 if (outcome %in% c("population")){
 dths_stroke_age_global <- who_ilo_long_populations %>%
 filter(age_bands_5yr %in% c("15_19", "20_24", "25_29", "30_34", "35_39", "40_44", "45_49", "50_54", "55_59", "60_64", "65_69", "70_74", "75_79", "80_84", "85_89", "90_94", "95_"),
 year == !!year) %>%
 arrange(region, country_abbrev, year, sex, age_bands_5yr) %>%
 drop_na() %>%
 group_by(age_bands_5yr) %>%
 summarise(result_b = sum(population)) %>%
 mutate(sd_b = NA,
 result_lwr_b = NA,
 result_upr_b = NA) %>%
 ungroup %>%
 pivot_wider(names_from = age_bands_5yr, values_from = result_b:result_upr_b) %>%
 select(result_b_15_19, sd_b_15_19, result_lwr_b_15_19, result_upr_b_15_19,
 result_b_20_24, sd_b_20_24, result_lwr_b_20_24, result_upr_b_20_24,
 result_b_25_29, sd_b_25_29, result_lwr_b_25_29, result_upr_b_25_29,
 result_b_30_34, sd_b_30_34, result_lwr_b_30_34, result_upr_b_30_34,
 result_b_35_39, sd_b_35_39, result_lwr_b_35_39, result_upr_b_35_39,
 result_b_40_44, sd_b_40_44, result_lwr_b_40_44, result_upr_b_40_44,
 result_b_45_49, sd_b_45_49, result_lwr_b_45_49, result_upr_b_45_49,
 result_b_50_54, sd_b_50_54, result_lwr_b_50_54, result_upr_b_50_54,
 result_b_55_59, sd_b_55_59, result_lwr_b_55_59, result_upr_b_55_59,
 result_b_60_64, sd_b_60_64, result_lwr_b_60_64, result_upr_b_60_64,
 result_b_65_69, sd_b_65_69, result_lwr_b_65_69, result_upr_b_65_69,
 result_b_70_74, sd_b_70_74, result_lwr_b_70_74, result_upr_b_70_74,
 result_b_75_79, sd_b_75_79, result_lwr_b_75_79, result_upr_b_75_79,
 result_b_80_84, sd_b_80_84, result_lwr_b_80_84, result_upr_b_80_84,
 result_b_85_89, sd_b_85_89, result_lwr_b_85_89, result_upr_b_85_89,
 result_b_90_94, sd_b_90_94, result_lwr_b_90_94, result_upr_b_90_94,
 result_b_95_, sd_b_95_, result_lwr_b_95_, result_upr_b_95_)
 }
 if (outcome %in% c("DTHratio")){
 envelope_stroke_age_global <- df_in %>%
 filter(outcome == "DTHparent",
 year == !!year,
 cause == !!cause) %>%
 arrange(region, country_abbrev, year, sex, ilo_age_bands_5yr) %>%
 drop_na() %>%
 mutate(sd = (result_upr - result_lwr)/2/1.96) %>%
 group_by(ilo_age_bands_5yr) %>%
 summarise(result = sum(result),
 sd = sqrt(sum((sd)^2))) %>%
 ungroup %>%
 pivot_wider(names_from = ilo_age_bands_5yr, values_from = result:sd)

 dths_stroke_age_global <- df_in %>%
 filter(outcome == "DTHenvelope",
 year == !!year,
 cause == !!cause) %>%
 arrange(region, country_abbrev, year, sex, ilo_age_bands_5yr) %>%
 drop_na() %>%
 mutate(sd = (result_upr - result_lwr)/2/1.96) %>%
 group_by(ilo_age_bands_5yr) %>%
 summarise(result = sum(result),
 sd = sqrt(sum((sd)^2))) %>%
 ungroup %>%
 pivot_wider(names_from = ilo_age_bands_5yr, values_from = result:sd) %>%

 select_all(.funs = funs(str_c(., "1")))

 dths_stroke_age_global <- bind_cols(dths_stroke_age_global, envelope_stroke_age_global) %>%
 rowwise() %>%
 transmute(result_b_15_19 = whoilo_paf_summary(result_15_19, result_15_191, CI = NULL),
 result_lwr_b_15_19 = whoilo_paf_summary(result_15_19, result_15_191, sd_15_19, sd_15_191, CI = 0.025),
 result_upr_b_15_19 = whoilo_paf_summary(result_15_19, result_15_191, sd_15_19, sd_15_191, CI = 0.975),
 result_b_20_24 = whoilo_paf_summary(result_20_24, result_20_241, CI = NULL),
 result_lwr_b_20_24 = whoilo_paf_summary(result_20_24, result_20_241, sd_20_24, sd_20_241, CI = 0.025),
 result_upr_b_20_24 = whoilo_paf_summary(result_20_24, result_20_241, sd_20_24, sd_20_241, CI = 0.975),
 result_b_25_29 = whoilo_paf_summary(result_25_29, result_25_291, CI = NULL),
 result_lwr_b_25_29 = whoilo_paf_summary(result_25_29, result_25_291, sd_25_29, sd_25_291, CI = 0.025),
 result_upr_b_25_29 = whoilo_paf_summary(result_25_29, result_25_291, sd_25_29, sd_25_291, CI = 0.975),
 result_b_30_34 = whoilo_paf_summary(result_30_34, result_30_341, CI = NULL),
 result_lwr_b_30_34 = whoilo_paf_summary(result_30_34, result_30_341, sd_30_34, sd_30_341, CI = 0.025),
 result_upr_b_30_34 = whoilo_paf_summary(result_30_34, result_30_341, sd_30_34, sd_30_341, CI = 0.975),
 result_b_35_39 = whoilo_paf_summary(result_35_39, result_35_391, CI = NULL),
 result_lwr_b_35_39 = whoilo_paf_summary(result_35_39, result_35_391, sd_35_39, sd_35_391, CI = 0.025),
 result_upr_b_35_39 = whoilo_paf_summary(result_35_39, result_35_391, sd_35_39, sd_35_391, CI = 0.975),
 result_b_40_44 = whoilo_paf_summary(result_40_44, result_40_441, CI = NULL),
 result_lwr_b_40_44 = whoilo_paf_summary(result_40_44, result_40_441, sd_40_44, sd_40_441, CI = 0.025),
 result_upr_b_40_44 = whoilo_paf_summary(result_40_44, result_40_441, sd_40_44, sd_40_441, CI = 0.975),
 result_b_45_49 = whoilo_paf_summary(result_45_49, result_45_491, CI = NULL),
 result_lwr_b_45_49 = whoilo_paf_summary(result_45_49, result_45_491, sd_45_49, sd_45_491, CI = 0.025),
 result_upr_b_45_49 = whoilo_paf_summary(result_45_49, result_45_491, sd_45_49, sd_45_491, CI = 0.975),
 result_b_50_54 = whoilo_paf_summary(result_50_54, result_50_541, CI = NULL),
 result_lwr_b_50_54 = whoilo_paf_summary(result_50_54, result_50_541, sd_50_54, sd_50_541, CI = 0.025),
 result_upr_b_50_54 = whoilo_paf_summary(result_50_54, result_50_541, sd_50_54, sd_50_541, CI = 0.975),
 result_b_55_59 = whoilo_paf_summary(result_55_59, result_55_591, CI = NULL),
 result_lwr_b_55_59 = whoilo_paf_summary(result_55_59, result_55_591, sd_55_59, sd_55_591, CI = 0.025),
 result_upr_b_55_59 = whoilo_paf_summary(result_55_59, result_55_591, sd_55_59, sd_55_591, CI = 0.975),
 result_b_60_64 = whoilo_paf_summary(result_60_64, result_60_641, CI = NULL),
 result_lwr_b_60_64 = whoilo_paf_summary(result_60_64, result_60_641, sd_60_64, sd_60_641, CI = 0.025),
 result_upr_b_60_64 = whoilo_paf_summary(result_60_64, result_60_641, sd_60_64, sd_60_641, CI = 0.975),
 result_b_65_69 = whoilo_paf_summary(result_65_69, result_65_691, CI = NULL),
 result_lwr_b_65_69 = whoilo_paf_summary(result_65_69, result_65_691, sd_65_69, sd_65_691, CI = 0.025),
 result_upr_b_65_69 = whoilo_paf_summary(result_65_69, result_65_691, sd_65_69, sd_65_691, CI = 0.975),
 result_b_70_74 = whoilo_paf_summary(result_70_74, result_70_741, CI = NULL),
 result_lwr_b_70_74 = whoilo_paf_summary(result_70_74, result_70_741, sd_70_74, sd_70_741, CI = 0.025),
 result_upr_b_70_74 = whoilo_paf_summary(result_70_74, result_70_741, sd_70_74, sd_70_741, CI = 0.975),
 result_b_75_79 = whoilo_paf_summary(result_75_79, result_75_791, CI = NULL),
 result_lwr_b_75_79 = whoilo_paf_summary(result_75_79, result_75_791, sd_75_79, sd_75_791, CI = 0.025),
 result_upr_b_75_79 = whoilo_paf_summary(result_75_79, result_75_791, sd_75_79, sd_75_791, CI = 0.975),
 result_b_80_84 = whoilo_paf_summary(result_80_84, result_80_841, CI = NULL),
 result_lwr_b_80_84 = whoilo_paf_summary(result_80_84, result_80_841, sd_80_84, sd_80_841, CI = 0.025),
 result_upr_b_80_84 = whoilo_paf_summary(result_80_84, result_80_841, sd_80_84, sd_80_841, CI = 0.975),
 result_b_85_89 = whoilo_paf_summary(result_85_89, result_85_891, CI = NULL),
 result_lwr_b_85_89 = whoilo_paf_summary(result_85_89, result_85_891, sd_85_89, sd_85_891, CI = 0.025),
 result_upr_b_85_89 = whoilo_paf_summary(result_85_89, result_85_891, sd_85_89, sd_85_891, CI = 0.975),
 result_b_90_94 = whoilo_paf_summary(result_90_94, result_90_941, CI = NULL),
 result_lwr_b_90_94 = whoilo_paf_summary(result_90_94, result_90_941, sd_90_94, sd_90_941, CI = 0.025),
 result_upr_b_90_94 = whoilo_paf_summary(result_90_94, result_90_941, sd_90_94, sd_90_941, CI = 0.975),
 result_b_95_ = whoilo_paf_summary(result_95_, result_95_1, CI = NULL),
 result_lwr_b_95_ = whoilo_paf_summary(result_95_, result_95_1, sd_95_, sd_95_1, CI = 0.025),
 result_upr_b_95_ = whoilo_paf_summary(result_95_, result_95_1, sd_95_, sd_95_1, CI = 0.975)) %>%
 ungroup() %>%
 mutate(sd_b_15_19 = (result_upr_b_15_19 - result_lwr_b_15_19)/2/1.96,
 sd_b_20_24 = (result_upr_b_20_24 - result_lwr_b_20_24)/2/1.96,
 sd_b_25_29 = (result_upr_b_25_29 - result_lwr_b_25_29)/2/1.96,
 sd_b_30_34 = (result_upr_b_30_34 - result_lwr_b_30_34)/2/1.96,
 sd_b_35_39 = (result_upr_b_35_39 - result_lwr_b_35_39)/2/1.96,
 sd_b_40_44 = (result_upr_b_40_44 - result_lwr_b_40_44)/2/1.96,
 sd_b_45_49 = (result_upr_b_45_49 - result_lwr_b_45_49)/2/1.96,
 sd_b_50_54 = (result_upr_b_50_54 - result_lwr_b_50_54)/2/1.96,
 sd_b_55_59 = (result_upr_b_55_59 - result_lwr_b_55_59)/2/1.96,
 sd_b_60_64 = (result_upr_b_60_64 - result_lwr_b_60_64)/2/1.96,
 sd_b_65_69 = (result_upr_b_65_69 - result_lwr_b_65_69)/2/1.96,
 sd_b_70_74 = (result_upr_b_70_74 - result_lwr_b_70_74)/2/1.96,
 sd_b_75_79 = (result_upr_b_75_79 - result_lwr_b_75_79)/2/1.96,
 sd_b_80_84 = (result_upr_b_80_84 - result_lwr_b_80_84)/2/1.96,
 sd_b_85_89 = (result_upr_b_85_89 - result_lwr_b_85_89)/2/1.96,
 sd_b_90_94 = (result_upr_b_90_94 - result_lwr_b_90_94)/2/1.96,
 sd_b_95_ = (result_upr_b_95_ - result_lwr_b_95_)/2/1.96) %>%
 select(result_b_15_19, sd_b_15_19, result_lwr_b_15_19, result_upr_b_15_19,
 result_b_20_24, sd_b_20_24, result_lwr_b_20_24, result_upr_b_20_24,
 result_b_25_29, sd_b_25_29, result_lwr_b_25_29, result_upr_b_25_29,
 result_b_30_34, sd_b_30_34, result_lwr_b_30_34, result_upr_b_30_34,
 result_b_35_39, sd_b_35_39, result_lwr_b_35_39, result_upr_b_35_39,
 result_b_40_44, sd_b_40_44, result_lwr_b_40_44, result_upr_b_40_44,
 result_b_45_49, sd_b_45_49, result_lwr_b_45_49, result_upr_b_45_49,
 result_b_50_54, sd_b_50_54, result_lwr_b_50_54, result_upr_b_50_54,
 result_b_55_59, sd_b_55_59, result_lwr_b_55_59, result_upr_b_55_59,
 result_b_60_64, sd_b_60_64, result_lwr_b_60_64, result_upr_b_60_64,
 result_b_65_69, sd_b_65_69, result_lwr_b_65_69, result_upr_b_65_69,
 result_b_70_74, sd_b_70_74, result_lwr_b_70_74, result_upr_b_70_74,
 result_b_75_79, sd_b_75_79, result_lwr_b_75_79, result_upr_b_75_79,
 result_b_80_84, sd_b_80_84, result_lwr_b_80_84, result_upr_b_80_84,
 result_b_85_89, sd_b_85_89, result_lwr_b_85_89, result_upr_b_85_89,
 result_b_90_94, sd_b_90_94, result_lwr_b_90_94, result_upr_b_90_94,
 result_b_95_, sd_b_95_, result_lwr_b_95_, result_upr_b_95_)
 }
 if (outcome %in% c("DALYratio")){
 envelope_stroke_age_global <- df_in %>%
 filter(outcome == "DALYparent",
 year == !!year,
 cause == !!cause) %>%
 arrange(region, country_abbrev, year, sex, ilo_age_bands_5yr) %>%
 drop_na() %>%
 mutate(sd = (result_upr - result_lwr)/2/1.96) %>%
 group_by(ilo_age_bands_5yr) %>%
 summarise(result = sum(result),
 sd = sqrt(sum((sd)^2))) %>%
 ungroup %>%
 pivot_wider(names_from = ilo_age_bands_5yr, values_from = result:sd)

 dths_stroke_age_global <- df_in %>%
 filter(outcome == "DALYenvelope",
 year == !!year,
 cause == !!cause) %>%
 mutate(result_lwr = case_when(is.na(result_lwr) ~ result,
 TRUE ~ result_lwr),
 result_upr = case_when(is.na(result_upr) ~ result,
 TRUE ~ result_upr)) %>%
 arrange(region, country_abbrev, year, sex, ilo_age_bands_5yr) %>%
 drop_na() %>%
 mutate(sd = (result_upr - result_lwr)/2/1.96) %>%
 group_by(ilo_age_bands_5yr) %>%
 summarise(result = sum(result),
 sd = sqrt(sum((sd)^2))) %>%
 ungroup %>%
 pivot_wider(names_from = ilo_age_bands_5yr, values_from = result:sd) %>%

 select_all(.funs = funs(str_c(., "1")))

 dths_stroke_age_global <- bind_cols(dths_stroke_age_global, envelope_stroke_age_global) %>%
 rowwise() %>%
 transmute(result_b_15_19 = whoilo_paf_summary(result_15_19, result_15_191, CI = NULL),
 result_lwr_b_15_19 = whoilo_paf_summary(result_15_19, result_15_191, sd_15_19, sd_15_191, CI = 0.025),
 result_upr_b_15_19 = whoilo_paf_summary(result_15_19, result_15_191, sd_15_19, sd_15_191, CI = 0.975),
 result_b_20_24 = whoilo_paf_summary(result_20_24, result_20_241, CI = NULL),
 result_lwr_b_20_24 = whoilo_paf_summary(result_20_24, result_20_241, sd_20_24, sd_20_241, CI = 0.025),
 result_upr_b_20_24 = whoilo_paf_summary(result_20_24, result_20_241, sd_20_24, sd_20_241, CI = 0.975),
 result_b_25_29 = whoilo_paf_summary(result_25_29, result_25_291, CI = NULL),
 result_lwr_b_25_29 = whoilo_paf_summary(result_25_29, result_25_291, sd_25_29, sd_25_291, CI = 0.025),
 result_upr_b_25_29 = whoilo_paf_summary(result_25_29, result_25_291, sd_25_29, sd_25_291, CI = 0.975),
 result_b_30_34 = whoilo_paf_summary(result_30_34, result_30_341, CI = NULL),
 result_lwr_b_30_34 = whoilo_paf_summary(result_30_34, result_30_341, sd_30_34, sd_30_341, CI = 0.025),
 result_upr_b_30_34 = whoilo_paf_summary(result_30_34, result_30_341, sd_30_34, sd_30_341, CI = 0.975),
 result_b_35_39 = whoilo_paf_summary(result_35_39, result_35_391, CI = NULL),
 result_lwr_b_35_39 = whoilo_paf_summary(result_35_39, result_35_391, sd_35_39, sd_35_391, CI = 0.025),
 result_upr_b_35_39 = whoilo_paf_summary(result_35_39, result_35_391, sd_35_39, sd_35_391, CI = 0.975),
 result_b_40_44 = whoilo_paf_summary(result_40_44, result_40_441, CI = NULL),
 result_lwr_b_40_44 = whoilo_paf_summary(result_40_44, result_40_441, sd_40_44, sd_40_441, CI = 0.025),
 result_upr_b_40_44 = whoilo_paf_summary(result_40_44, result_40_441, sd_40_44, sd_40_441, CI = 0.975),
 result_b_45_49 = whoilo_paf_summary(result_45_49, result_45_491, CI = NULL),
 result_lwr_b_45_49 = whoilo_paf_summary(result_45_49, result_45_491, sd_45_49, sd_45_491, CI = 0.025),
 result_upr_b_45_49 = whoilo_paf_summary(result_45_49, result_45_491, sd_45_49, sd_45_491, CI = 0.975),
 result_b_50_54 = whoilo_paf_summary(result_50_54, result_50_541, CI = NULL),
 result_lwr_b_50_54 = whoilo_paf_summary(result_50_54, result_50_541, sd_50_54, sd_50_541, CI = 0.025),
 result_upr_b_50_54 = whoilo_paf_summary(result_50_54, result_50_541, sd_50_54, sd_50_541, CI = 0.975),
 result_b_55_59 = whoilo_paf_summary(result_55_59, result_55_591, CI = NULL),
 result_lwr_b_55_59 = whoilo_paf_summary(result_55_59, result_55_591, sd_55_59, sd_55_591, CI = 0.025),
 result_upr_b_55_59 = whoilo_paf_summary(result_55_59, result_55_591, sd_55_59, sd_55_591, CI = 0.975),
 result_b_60_64 = whoilo_paf_summary(result_60_64, result_60_641, CI = NULL),
 result_lwr_b_60_64 = whoilo_paf_summary(result_60_64, result_60_641, sd_60_64, sd_60_641, CI = 0.025),
 result_upr_b_60_64 = whoilo_paf_summary(result_60_64, result_60_641, sd_60_64, sd_60_641, CI = 0.975),
 result_b_65_69 = whoilo_paf_summary(result_65_69, result_65_691, CI = NULL),
 result_lwr_b_65_69 = whoilo_paf_summary(result_65_69, result_65_691, sd_65_69, sd_65_691, CI = 0.025),
 result_upr_b_65_69 = whoilo_paf_summary(result_65_69, result_65_691, sd_65_69, sd_65_691, CI = 0.975),
 result_b_70_74 = whoilo_paf_summary(result_70_74, result_70_741, CI = NULL),
 result_lwr_b_70_74 = whoilo_paf_summary(result_70_74, result_70_741, sd_70_74, sd_70_741, CI = 0.025),
 result_upr_b_70_74 = whoilo_paf_summary(result_70_74, result_70_741, sd_70_74, sd_70_741, CI = 0.975),
 result_b_75_79 = whoilo_paf_summary(result_75_79, result_75_791, CI = NULL),
 result_lwr_b_75_79 = whoilo_paf_summary(result_75_79, result_75_791, sd_75_79, sd_75_791, CI = 0.025),
 result_upr_b_75_79 = whoilo_paf_summary(result_75_79, result_75_791, sd_75_79, sd_75_791, CI = 0.975),
 result_b_80_84 = whoilo_paf_summary(result_80_84, result_80_841, CI = NULL),
 result_lwr_b_80_84 = whoilo_paf_summary(result_80_84, result_80_841, sd_80_84, sd_80_841, CI = 0.025),
 result_upr_b_80_84 = whoilo_paf_summary(result_80_84, result_80_841, sd_80_84, sd_80_841, CI = 0.975),
 result_b_85_89 = whoilo_paf_summary(result_85_89, result_85_891, CI = NULL),
 result_lwr_b_85_89 = whoilo_paf_summary(result_85_89, result_85_891, sd_85_89, sd_85_891, CI = 0.025),
 result_upr_b_85_89 = whoilo_paf_summary(result_85_89, result_85_891, sd_85_89, sd_85_891, CI = 0.975),
 result_b_90_94 = whoilo_paf_summary(result_90_94, result_90_941, CI = NULL),
 result_lwr_b_90_94 = whoilo_paf_summary(result_90_94, result_90_941, sd_90_94, sd_90_941, CI = 0.025),
 result_upr_b_90_94 = whoilo_paf_summary(result_90_94, result_90_941, sd_90_94, sd_90_941, CI = 0.975),
 result_b_95_ = whoilo_paf_summary(result_95_, result_95_1, CI = NULL),
 result_lwr_b_95_ = whoilo_paf_summary(result_95_, result_95_1, sd_95_, sd_95_1, CI = 0.025),
 result_upr_b_95_ = whoilo_paf_summary(result_95_, result_95_1, sd_95_, sd_95_1, CI = 0.975)) %>%
 ungroup() %>%
 mutate(sd_b_15_19 = (result_upr_b_15_19 - result_lwr_b_15_19)/2/1.96,
 sd_b_20_24 = (result_upr_b_20_24 - result_lwr_b_20_24)/2/1.96,
 sd_b_25_29 = (result_upr_b_25_29 - result_lwr_b_25_29)/2/1.96,
 sd_b_30_34 = (result_upr_b_30_34 - result_lwr_b_30_34)/2/1.96,
 sd_b_35_39 = (result_upr_b_35_39 - result_lwr_b_35_39)/2/1.96,
 sd_b_40_44 = (result_upr_b_40_44 - result_lwr_b_40_44)/2/1.96,
 sd_b_45_49 = (result_upr_b_45_49 - result_lwr_b_45_49)/2/1.96,
 sd_b_50_54 = (result_upr_b_50_54 - result_lwr_b_50_54)/2/1.96,
 sd_b_55_59 = (result_upr_b_55_59 - result_lwr_b_55_59)/2/1.96,
 sd_b_60_64 = (result_upr_b_60_64 - result_lwr_b_60_64)/2/1.96,
 sd_b_65_69 = (result_upr_b_65_69 - result_lwr_b_65_69)/2/1.96,
 sd_b_70_74 = (result_upr_b_70_74 - result_lwr_b_70_74)/2/1.96,
 sd_b_75_79 = (result_upr_b_75_79 - result_lwr_b_75_79)/2/1.96,
 sd_b_80_84 = (result_upr_b_80_84 - result_lwr_b_80_84)/2/1.96,
 sd_b_85_89 = (result_upr_b_85_89 - result_lwr_b_85_89)/2/1.96,
 sd_b_90_94 = (result_upr_b_90_94 - result_lwr_b_90_94)/2/1.96,
 sd_b_95_ = (result_upr_b_95_ - result_lwr_b_95_)/2/1.96) %>%
 select(result_b_15_19, sd_b_15_19, result_lwr_b_15_19, result_upr_b_15_19,
 result_b_20_24, sd_b_20_24, result_lwr_b_20_24, result_upr_b_20_24,
 result_b_25_29, sd_b_25_29, result_lwr_b_25_29, result_upr_b_25_29,
 result_b_30_34, sd_b_30_34, result_lwr_b_30_34, result_upr_b_30_34,
 result_b_35_39, sd_b_35_39, result_lwr_b_35_39, result_upr_b_35_39,
 result_b_40_44, sd_b_40_44, result_lwr_b_40_44, result_upr_b_40_44,
 result_b_45_49, sd_b_45_49, result_lwr_b_45_49, result_upr_b_45_49,
 result_b_50_54, sd_b_50_54, result_lwr_b_50_54, result_upr_b_50_54,
 result_b_55_59, sd_b_55_59, result_lwr_b_55_59, result_upr_b_55_59,
 result_b_60_64, sd_b_60_64, result_lwr_b_60_64, result_upr_b_60_64,
 result_b_65_69, sd_b_65_69, result_lwr_b_65_69, result_upr_b_65_69,
 result_b_70_74, sd_b_70_74, result_lwr_b_70_74, result_upr_b_70_74,
 result_b_75_79, sd_b_75_79, result_lwr_b_75_79, result_upr_b_75_79,
 result_b_80_84, sd_b_80_84, result_lwr_b_80_84, result_upr_b_80_84,
 result_b_85_89, sd_b_85_89, result_lwr_b_85_89, result_upr_b_85_89,
 result_b_90_94, sd_b_90_94, result_lwr_b_90_94, result_upr_b_90_94,
 result_b_95_, sd_b_95_, result_lwr_b_95_, result_upr_b_95_)
 }
 }
 # by sexage
 if ("sexage" %in% breakdown){
 if (outcome %in% c("PAF", "DTHpaf")){
 envelope_stroke_sexage_global <- df_in %>%
 filter(outcome == "DTHenvelope",
 year == !!year,
 cause == !!cause) %>%
 arrange(region, country_abbrev, year, sex, ilo_age_bands_5yr) %>%
 drop_na() %>%
 mutate(sd = (result_upr - result_lwr)/2/1.96) %>%
 group_by(sex, ilo_age_bands_5yr) %>%
 summarise(result = sum(result),
 sd = sqrt(sum((sd)^2))) %>%
 ungroup %>%
 pivot_wider(names_from = sex:ilo_age_bands_5yr, values_from = result:sd) %>%

 select_all(.funs = funs(str_c(., "1")))

 dths_stroke_sexage_global <- df_in %>%
 filter(outcome == "DTH",
 year == !!year,
 cause == !!cause) %>%
 arrange(region, country_abbrev, year, sex, ilo_age_bands_5yr) %>%
 drop_na() %>%
 mutate(sd = (result_upr - result_lwr)/2/1.96) %>%
 group_by(sex, ilo_age_bands_5yr) %>%
 summarise(result = sum(result),
 sd = sqrt(sum((sd)^2))) %>%
 ungroup %>%
 pivot_wider(names_from = sex:ilo_age_bands_5yr, values_from = result:sd)
 dths_stroke_sexage_global <- bind_cols(dths_stroke_sexage_global, envelope_stroke_sexage_global) %>%
 rowwise() %>%
 transmute(result_lwr_f_15_19 = whoilo_paf_summary(result_f_15_19, result_f_15_191, sd_f_15_19, sd_f_15_191, CI = 0.025),
 result_upr_f_15_19 = whoilo_paf_summary(result_f_15_19, result_f_15_191, sd_f_15_19, sd_f_15_191, CI = 0.975),
 result_f_15_19 = whoilo_paf_summary(result_f_15_19, result_f_15_191, CI = NULL),
 result_lwr_f_20_24 = whoilo_paf_summary(result_f_20_24, result_f_20_241, sd_f_20_24, sd_f_20_241, CI = 0.025),
 result_upr_f_20_24 = whoilo_paf_summary(result_f_20_24, result_f_20_241, sd_f_20_24, sd_f_20_241, CI = 0.975),
 result_f_20_24 = whoilo_paf_summary(result_f_20_24, result_f_20_241, CI = NULL),
 result_lwr_f_25_29 = whoilo_paf_summary(result_f_25_29, result_f_25_291, sd_f_25_29, sd_f_25_291, CI = 0.025),
 result_upr_f_25_29 = whoilo_paf_summary(result_f_25_29, result_f_25_291, sd_f_25_29, sd_f_25_291, CI = 0.975),
 result_f_25_29 = whoilo_paf_summary(result_f_25_29, result_f_25_291, CI = NULL),
 result_lwr_f_30_34 = whoilo_paf_summary(result_f_30_34, result_f_30_341, sd_f_30_34, sd_f_30_341, CI = 0.025),
 result_upr_f_30_34 = whoilo_paf_summary(result_f_30_34, result_f_30_341, sd_f_30_34, sd_f_30_341, CI = 0.975),
 result_f_30_34 = whoilo_paf_summary(result_f_30_34, result_f_30_341, CI = NULL),
 result_lwr_f_35_39 = whoilo_paf_summary(result_f_35_39, result_f_35_391, sd_f_35_39, sd_f_35_391, CI = 0.025),
 result_upr_f_35_39 = whoilo_paf_summary(result_f_35_39, result_f_35_391, sd_f_35_39, sd_f_35_391, CI = 0.975),
 result_f_35_39 = whoilo_paf_summary(result_f_35_39, result_f_35_391, CI = NULL),
 result_lwr_f_40_44 = whoilo_paf_summary(result_f_40_44, result_f_40_441, sd_f_40_44, sd_f_40_441, CI = 0.025),
 result_upr_f_40_44 = whoilo_paf_summary(result_f_40_44, result_f_40_441, sd_f_40_44, sd_f_40_441, CI = 0.975),
 result_f_40_44 = whoilo_paf_summary(result_f_40_44, result_f_40_441, CI = NULL),
 result_lwr_f_45_49 = whoilo_paf_summary(result_f_45_49, result_f_45_491, sd_f_45_49, sd_f_45_491, CI = 0.025),
 result_upr_f_45_49 = whoilo_paf_summary(result_f_45_49, result_f_45_491, sd_f_45_49, sd_f_45_491, CI = 0.975),
 result_f_45_49 = whoilo_paf_summary(result_f_45_49, result_f_45_491, CI = NULL),
 result_lwr_f_50_54 = whoilo_paf_summary(result_f_50_54, result_f_50_541, sd_f_50_54, sd_f_50_541, CI = 0.025),
 result_upr_f_50_54 = whoilo_paf_summary(result_f_50_54, result_f_50_541, sd_f_50_54, sd_f_50_541, CI = 0.975),
 result_f_50_54 = whoilo_paf_summary(result_f_50_54, result_f_50_541, CI = NULL),
 result_lwr_f_55_59 = whoilo_paf_summary(result_f_55_59, result_f_55_591, sd_f_55_59, sd_f_55_591, CI = 0.025),
 result_upr_f_55_59 = whoilo_paf_summary(result_f_55_59, result_f_55_591, sd_f_55_59, sd_f_55_591, CI = 0.975),
 result_f_55_59 = whoilo_paf_summary(result_f_55_59, result_f_55_591, CI = NULL),
 result_lwr_f_60_64 = whoilo_paf_summary(result_f_60_64, result_f_60_641, sd_f_60_64, sd_f_60_641, CI = 0.025),
 result_upr_f_60_64 = whoilo_paf_summary(result_f_60_64, result_f_60_641, sd_f_60_64, sd_f_60_641, CI = 0.975),
 result_f_60_64 = whoilo_paf_summary(result_f_60_64, result_f_60_641, CI = NULL),
 result_lwr_f_65_69 = whoilo_paf_summary(result_f_65_69, result_f_65_691, sd_f_65_69, sd_f_65_691, CI = 0.025),
 result_upr_f_65_69 = whoilo_paf_summary(result_f_65_69, result_f_65_691, sd_f_65_69, sd_f_65_691, CI = 0.975),
 result_f_65_69 = whoilo_paf_summary(result_f_65_69, result_f_65_691, CI = NULL),
 result_lwr_f_70_74 = whoilo_paf_summary(result_f_70_74, result_f_70_741, sd_f_70_74, sd_f_70_741, CI = 0.025),
 result_upr_f_70_74 = whoilo_paf_summary(result_f_70_74, result_f_70_741, sd_f_70_74, sd_f_70_741, CI = 0.975),
 result_f_70_74 = whoilo_paf_summary(result_f_70_74, result_f_70_741, CI = NULL),
 result_lwr_f_75_79 = whoilo_paf_summary(result_f_75_79, result_f_75_791, sd_f_75_79, sd_f_75_791, CI = 0.025),
 result_upr_f_75_79 = whoilo_paf_summary(result_f_75_79, result_f_75_791, sd_f_75_79, sd_f_75_791, CI = 0.975),
 result_f_75_79 = whoilo_paf_summary(result_f_75_79, result_f_75_791, CI = NULL),
 result_lwr_f_80_84 = whoilo_paf_summary(result_f_80_84, result_f_80_841, sd_f_80_84, sd_f_80_841, CI = 0.025),
 result_upr_f_80_84 = whoilo_paf_summary(result_f_80_84, result_f_80_841, sd_f_80_84, sd_f_80_841, CI = 0.975),
 result_f_80_84 = whoilo_paf_summary(result_f_80_84, result_f_80_841, CI = NULL),
 result_lwr_f_85_89 = whoilo_paf_summary(result_f_85_89, result_f_85_891, sd_f_85_89, sd_f_85_891, CI = 0.025),
 result_upr_f_85_89 = whoilo_paf_summary(result_f_85_89, result_f_85_891, sd_f_85_89, sd_f_85_891, CI = 0.975),
 result_f_85_89 = whoilo_paf_summary(result_f_85_89, result_f_85_891, CI = NULL),
 result_lwr_f_90_94 = whoilo_paf_summary(result_f_90_94, result_f_90_941, sd_f_90_94, sd_f_90_941, CI = 0.025),
 result_upr_f_90_94 = whoilo_paf_summary(result_f_90_94, result_f_90_941, sd_f_90_94, sd_f_90_941, CI = 0.975),
 result_f_90_94 = whoilo_paf_summary(result_f_90_94, result_f_90_941, CI = NULL),
 result_lwr_f_95_ = whoilo_paf_summary(result_f_95_, result_f_95_1, sd_f_95_, sd_f_95_1, CI = 0.025),
 result_upr_f_95_ = whoilo_paf_summary(result_f_95_, result_f_95_1, sd_f_95_, sd_f_95_1, CI = 0.975),
 result_f_95_ = whoilo_paf_summary(result_f_95_, result_f_95_1, CI = NULL),
 result_lwr_m_15_19 = whoilo_paf_summary(result_m_15_19, result_m_15_191, sd_m_15_19, sd_m_15_191, CI = 0.025),
 result_upr_m_15_19 = whoilo_paf_summary(result_m_15_19, result_m_15_191, sd_m_15_19, sd_m_15_191, CI = 0.975),
 result_m_15_19 = whoilo_paf_summary(result_m_15_19, result_m_15_191, CI = NULL),
 result_lwr_m_20_24 = whoilo_paf_summary(result_m_20_24, result_m_20_241, sd_m_20_24, sd_m_20_241, CI = 0.025),
 result_upr_m_20_24 = whoilo_paf_summary(result_m_20_24, result_m_20_241, sd_m_20_24, sd_m_20_241, CI = 0.975),
 result_m_20_24 = whoilo_paf_summary(result_m_20_24, result_m_20_241, CI = NULL),
 result_lwr_m_25_29 = whoilo_paf_summary(result_m_25_29, result_m_25_291, sd_m_25_29, sd_m_25_291, CI = 0.025),
 result_upr_m_25_29 = whoilo_paf_summary(result_m_25_29, result_m_25_291, sd_m_25_29, sd_m_25_291, CI = 0.975),
 result_m_25_29 = whoilo_paf_summary(result_m_25_29, result_m_25_291, CI = NULL),
 result_lwr_m_30_34 = whoilo_paf_summary(result_m_30_34, result_m_30_341, sd_m_30_34, sd_m_30_341, CI = 0.025),
 result_upr_m_30_34 = whoilo_paf_summary(result_m_30_34, result_m_30_341, sd_m_30_34, sd_m_30_341, CI = 0.975),
 result_m_30_34 = whoilo_paf_summary(result_m_30_34, result_m_30_341, CI = NULL),
 result_lwr_m_35_39 = whoilo_paf_summary(result_m_35_39, result_m_35_391, sd_m_35_39, sd_m_35_391, CI = 0.025),
 result_upr_m_35_39 = whoilo_paf_summary(result_m_35_39, result_m_35_391, sd_m_35_39, sd_m_35_391, CI = 0.975),
 result_m_35_39 = whoilo_paf_summary(result_m_35_39, result_m_35_391, CI = NULL),
 result_lwr_m_40_44 = whoilo_paf_summary(result_m_40_44, result_m_40_441, sd_m_40_44, sd_m_40_441, CI = 0.025),
 result_upr_m_40_44 = whoilo_paf_summary(result_m_40_44, result_m_40_441, sd_m_40_44, sd_m_40_441, CI = 0.975),
 result_m_40_44 = whoilo_paf_summary(result_m_40_44, result_m_40_441, CI = NULL),
 result_lwr_m_45_49 = whoilo_paf_summary(result_m_45_49, result_m_45_491, sd_m_45_49, sd_m_45_491, CI = 0.025),
 result_upr_m_45_49 = whoilo_paf_summary(result_m_45_49, result_m_45_491, sd_m_45_49, sd_m_45_491, CI = 0.975),
 result_m_45_49 = whoilo_paf_summary(result_m_45_49, result_m_45_491, CI = NULL),
 result_lwr_m_50_54 = whoilo_paf_summary(result_m_50_54, result_m_50_541, sd_m_50_54, sd_m_50_541, CI = 0.025),
 result_upr_m_50_54 = whoilo_paf_summary(result_m_50_54, result_m_50_541, sd_m_50_54, sd_m_50_541, CI = 0.975),
 result_m_50_54 = whoilo_paf_summary(result_m_50_54, result_m_50_541, CI = NULL),
 result_lwr_m_55_59 = whoilo_paf_summary(result_m_55_59, result_m_55_591, sd_m_55_59, sd_m_55_591, CI = 0.025),
 result_upr_m_55_59 = whoilo_paf_summary(result_m_55_59, result_m_55_591, sd_m_55_59, sd_m_55_591, CI = 0.975),
 result_m_55_59 = whoilo_paf_summary(result_m_55_59, result_m_55_591, CI = NULL),
 result_lwr_m_60_64 = whoilo_paf_summary(result_m_60_64, result_m_60_641, sd_m_60_64, sd_m_60_641, CI = 0.025),
 result_upr_m_60_64 = whoilo_paf_summary(result_m_60_64, result_m_60_641, sd_m_60_64, sd_m_60_641, CI = 0.975),
 result_m_60_64 = whoilo_paf_summary(result_m_60_64, result_m_60_641, CI = NULL),
 result_lwr_m_65_69 = whoilo_paf_summary(result_m_65_69, result_m_65_691, sd_m_65_69, sd_m_65_691, CI = 0.025),
 result_upr_m_65_69 = whoilo_paf_summary(result_m_65_69, result_m_65_691, sd_m_65_69, sd_m_65_691, CI = 0.975),
 result_m_65_69 = whoilo_paf_summary(result_m_65_69, result_m_65_691, CI = NULL),
 result_lwr_m_70_74 = whoilo_paf_summary(result_m_70_74, result_m_70_741, sd_m_70_74, sd_m_70_741, CI = 0.025),
 result_upr_m_70_74 = whoilo_paf_summary(result_m_70_74, result_m_70_741, sd_m_70_74, sd_m_70_741, CI = 0.975),
 result_m_70_74 = whoilo_paf_summary(result_m_70_74, result_m_70_741, CI = NULL),
 result_lwr_m_75_79 = whoilo_paf_summary(result_m_75_79, result_m_75_791, sd_m_75_79, sd_m_75_791, CI = 0.025),
 result_upr_m_75_79 = whoilo_paf_summary(result_m_75_79, result_m_75_791, sd_m_75_79, sd_m_75_791, CI = 0.975),
 result_m_75_79 = whoilo_paf_summary(result_m_75_79, result_m_75_791, CI = NULL),
 result_lwr_m_80_84 = whoilo_paf_summary(result_m_80_84, result_m_80_841, sd_m_80_84, sd_m_80_841, CI = 0.025),
 result_upr_m_80_84 = whoilo_paf_summary(result_m_80_84, result_m_80_841, sd_m_80_84, sd_m_80_841, CI = 0.975),
 result_m_80_84 = whoilo_paf_summary(result_m_80_84, result_m_80_841, CI = NULL),
 result_lwr_m_85_89 = whoilo_paf_summary(result_m_85_89, result_m_85_891, sd_m_85_89, sd_m_85_891, CI = 0.025),
 result_upr_m_85_89 = whoilo_paf_summary(result_m_85_89, result_m_85_891, sd_m_85_89, sd_m_85_891, CI = 0.975),
 result_m_85_89 = whoilo_paf_summary(result_m_85_89, result_m_85_891, CI = NULL),
 result_lwr_m_90_94 = whoilo_paf_summary(result_m_90_94, result_m_90_941, sd_m_90_94, sd_m_90_941, CI = 0.025),
 result_upr_m_90_94 = whoilo_paf_summary(result_m_90_94, result_m_90_941, sd_m_90_94, sd_m_90_941, CI = 0.975),
 result_m_90_94 = whoilo_paf_summary(result_m_90_94, result_m_90_941, CI = NULL),
 result_lwr_m_95_ = whoilo_paf_summary(result_m_95_, result_m_95_1, sd_m_95_, sd_m_95_1, CI = 0.025),
 result_upr_m_95_ = whoilo_paf_summary(result_m_95_, result_m_95_1, sd_m_95_, sd_m_95_1, CI = 0.975),
 result_m_95_ = whoilo_paf_summary(result_m_95_, result_m_95_1, CI = NULL)) %>%
 ungroup() %>%
 mutate(sd_f_15_19 = (result_upr_f_15_19 - result_lwr_f_15_19)/2/1.96,
 sd_f_20_24 = (result_upr_f_20_24 - result_lwr_f_20_24)/2/1.96,
 sd_f_25_29 = (result_upr_f_25_29 - result_lwr_f_25_29)/2/1.96,
 sd_f_30_34 = (result_upr_f_30_34 - result_lwr_f_30_34)/2/1.96,
 sd_f_35_39 = (result_upr_f_35_39 - result_lwr_f_35_39)/2/1.96,
 sd_f_40_44 = (result_upr_f_40_44 - result_lwr_f_40_44)/2/1.96,
 sd_f_45_49 = (result_upr_f_45_49 - result_lwr_f_45_49)/2/1.96,
 sd_f_50_54 = (result_upr_f_50_54 - result_lwr_f_50_54)/2/1.96,
 sd_f_55_59 = (result_upr_f_55_59 - result_lwr_f_55_59)/2/1.96,
 sd_f_60_64 = (result_upr_f_60_64 - result_lwr_f_60_64)/2/1.96,
 sd_f_65_69 = (result_upr_f_65_69 - result_lwr_f_65_69)/2/1.96,
 sd_f_70_74 = (result_upr_f_70_74 - result_lwr_f_70_74)/2/1.96,
 sd_f_75_79 = (result_upr_f_75_79 - result_lwr_f_75_79)/2/1.96,
 sd_f_80_84 = (result_upr_f_80_84 - result_lwr_f_80_84)/2/1.96,
 sd_f_85_89 = (result_upr_f_85_89 - result_lwr_f_85_89)/2/1.96,
 sd_f_90_94 = (result_upr_f_90_94 - result_lwr_f_90_94)/2/1.96,
 sd_f_95_ = (result_upr_f_95_ - result_lwr_f_95_)/2/1.96,
 sd_m_15_19 = (result_upr_m_15_19 - result_lwr_m_15_19)/2/1.96,
 sd_m_20_24 = (result_upr_m_20_24 - result_lwr_m_20_24)/2/1.96,
 sd_m_25_29 = (result_upr_m_25_29 - result_lwr_m_25_29)/2/1.96,
 sd_m_30_34 = (result_upr_m_30_34 - result_lwr_m_30_34)/2/1.96,
 sd_m_35_39 = (result_upr_m_35_39 - result_lwr_m_35_39)/2/1.96,
 sd_m_40_44 = (result_upr_m_40_44 - result_lwr_m_40_44)/2/1.96,
 sd_m_45_49 = (result_upr_m_45_49 - result_lwr_m_45_49)/2/1.96,
 sd_m_50_54 = (result_upr_m_50_54 - result_lwr_m_50_54)/2/1.96,
 sd_m_55_59 = (result_upr_m_55_59 - result_lwr_m_55_59)/2/1.96,
 sd_m_60_64 = (result_upr_m_60_64 - result_lwr_m_60_64)/2/1.96,
 sd_m_65_69 = (result_upr_m_65_69 - result_lwr_m_65_69)/2/1.96,
 sd_m_70_74 = (result_upr_m_70_74 - result_lwr_m_70_74)/2/1.96,
 sd_m_75_79 = (result_upr_m_75_79 - result_lwr_m_75_79)/2/1.96,
 sd_m_80_84 = (result_upr_m_80_84 - result_lwr_m_80_84)/2/1.96,
 sd_m_85_89 = (result_upr_m_85_89 - result_lwr_m_85_89)/2/1.96,
 sd_m_90_94 = (result_upr_m_90_94 - result_lwr_m_90_94)/2/1.96,
 sd_m_95_ = (result_upr_m_95_ - result_lwr_m_95_)/2/1.96) %>%
 select(result_f_15_19, sd_f_15_19, result_lwr_f_15_19, result_upr_f_15_19,
 result_f_20_24, sd_f_20_24, result_lwr_f_20_24, result_upr_f_20_24,
 result_f_25_29, sd_f_25_29, result_lwr_f_25_29, result_upr_f_25_29,
 result_f_30_34, sd_f_30_34, result_lwr_f_30_34, result_upr_f_30_34,
 result_f_35_39, sd_f_35_39, result_lwr_f_35_39, result_upr_f_35_39,
 result_f_40_44, sd_f_40_44, result_lwr_f_40_44, result_upr_f_40_44,
 result_f_45_49, sd_f_45_49, result_lwr_f_45_49, result_upr_f_45_49,
 result_f_50_54, sd_f_50_54, result_lwr_f_50_54, result_upr_f_50_54,
 result_f_55_59, sd_f_55_59, result_lwr_f_55_59, result_upr_f_55_59,
 result_f_60_64, sd_f_60_64, result_lwr_f_60_64, result_upr_f_60_64,
 result_f_65_69, sd_f_65_69, result_lwr_f_65_69, result_upr_f_65_69,
 result_f_70_74, sd_f_70_74, result_lwr_f_70_74, result_upr_f_70_74,
 result_f_75_79, sd_f_75_79, result_lwr_f_75_79, result_upr_f_75_79,
 result_f_80_84, sd_f_80_84, result_lwr_f_80_84, result_upr_f_80_84,
 result_f_85_89, sd_f_85_89, result_lwr_f_85_89, result_upr_f_85_89,
 result_f_90_94, sd_f_90_94, result_lwr_f_90_94, result_upr_f_90_94,
 result_f_95_, sd_f_95_, result_lwr_f_95_, result_upr_f_95_,
 result_m_15_19, sd_m_15_19, result_lwr_m_15_19, result_upr_m_15_19,
 result_m_20_24, sd_m_20_24, result_lwr_m_20_24, result_upr_m_20_24,
 result_m_25_29, sd_m_25_29, result_lwr_m_25_29, result_upr_m_25_29,
 result_m_30_34, sd_m_30_34, result_lwr_m_30_34, result_upr_m_30_34,
 result_m_35_39, sd_m_35_39, result_lwr_m_35_39, result_upr_m_35_39,
 result_m_40_44, sd_m_40_44, result_lwr_m_40_44, result_upr_m_40_44,
 result_m_45_49, sd_m_45_49, result_lwr_m_45_49, result_upr_m_45_49,
 result_m_50_54, sd_m_50_54, result_lwr_m_50_54, result_upr_m_50_54,
 result_m_55_59, sd_m_55_59, result_lwr_m_55_59, result_upr_m_55_59,
 result_m_60_64, sd_m_60_64, result_lwr_m_60_64, result_upr_m_60_64,
 result_m_65_69, sd_m_65_69, result_lwr_m_65_69, result_upr_m_65_69,
 result_m_70_74, sd_m_70_74, result_lwr_m_70_74, result_upr_m_70_74,
 result_m_75_79, sd_m_75_79, result_lwr_m_75_79, result_upr_m_75_79,
 result_m_80_84, sd_m_80_84, result_lwr_m_80_84, result_upr_m_80_84,
 result_m_85_89, sd_m_85_89, result_lwr_m_85_89, result_upr_m_85_89,
 result_m_90_94, sd_m_90_94, result_lwr_m_90_94, result_upr_m_90_94,
 result_m_95_, sd_m_95_, result_lwr_m_95_, result_upr_m_95_)
 if (shape == "long") {
 dths_stroke_sexage_global <- dths_stroke_sexage_global

 }
 }
 if (outcome %in% c("DALYpaf")){
 envelope_stroke_sexage_global <- df_in %>%
 filter(outcome == "DALYenvelope",
 year == !!year,
 cause == !!cause) %>%
 mutate(result_lwr = case_when(is.na(result_lwr) ~ result,
 TRUE ~ result_lwr),
 result_upr = case_when(is.na(result_upr) ~ result,
 TRUE ~ result_upr)) %>%
 arrange(region, country_abbrev, year, sex, ilo_age_bands_5yr) %>%
 drop_na() %>%
 mutate(sd = (result_upr - result_lwr)/2/1.96) %>%
 group_by(sex, ilo_age_bands_5yr) %>%
 summarise(result = sum(result),
 sd = sqrt(sum((sd)^2))) %>%
 ungroup %>%
 pivot_wider(names_from = sex:ilo_age_bands_5yr, values_from = result:sd) %>%

 select_all(.funs = funs(str_c(., "1")))

 dths_stroke_sexage_global <- df_in %>%
 filter(outcome == "DALY",
 year == !!year,
 cause == !!cause) %>%
 arrange(region, country_abbrev, year, sex, ilo_age_bands_5yr) %>%
 drop_na() %>%
 mutate(sd = (result_upr - result_lwr)/2/1.96) %>%
 group_by(sex, ilo_age_bands_5yr) %>%
 summarise(result = sum(result),
 sd = sqrt(sum((sd)^2))) %>%
 ungroup %>%
 pivot_wider(names_from = sex:ilo_age_bands_5yr, values_from = result:sd)
 dths_stroke_sexage_global <- bind_cols(dths_stroke_sexage_global, envelope_stroke_sexage_global) %>%
 rowwise() %>%
 transmute(result_lwr_f_15_19 = whoilo_paf_summary(result_f_15_19, result_f_15_191, sd_f_15_19, sd_f_15_191, CI = 0.025),
 result_upr_f_15_19 = whoilo_paf_summary(result_f_15_19, result_f_15_191, sd_f_15_19, sd_f_15_191, CI = 0.975),
 result_f_15_19 = whoilo_paf_summary(result_f_15_19, result_f_15_191, CI = NULL),
 result_lwr_f_20_24 = whoilo_paf_summary(result_f_20_24, result_f_20_241, sd_f_20_24, sd_f_20_241, CI = 0.025),
 result_upr_f_20_24 = whoilo_paf_summary(result_f_20_24, result_f_20_241, sd_f_20_24, sd_f_20_241, CI = 0.975),
 result_f_20_24 = whoilo_paf_summary(result_f_20_24, result_f_20_241, CI = NULL),
 result_lwr_f_25_29 = whoilo_paf_summary(result_f_25_29, result_f_25_291, sd_f_25_29, sd_f_25_291, CI = 0.025),
 result_upr_f_25_29 = whoilo_paf_summary(result_f_25_29, result_f_25_291, sd_f_25_29, sd_f_25_291, CI = 0.975),
 result_f_25_29 = whoilo_paf_summary(result_f_25_29, result_f_25_291, CI = NULL),
 result_lwr_f_30_34 = whoilo_paf_summary(result_f_30_34, result_f_30_341, sd_f_30_34, sd_f_30_341, CI = 0.025),
 result_upr_f_30_34 = whoilo_paf_summary(result_f_30_34, result_f_30_341, sd_f_30_34, sd_f_30_341, CI = 0.975),
 result_f_30_34 = whoilo_paf_summary(result_f_30_34, result_f_30_341, CI = NULL),
 result_lwr_f_35_39 = whoilo_paf_summary(result_f_35_39, result_f_35_391, sd_f_35_39, sd_f_35_391, CI = 0.025),
 result_upr_f_35_39 = whoilo_paf_summary(result_f_35_39, result_f_35_391, sd_f_35_39, sd_f_35_391, CI = 0.975),
 result_f_35_39 = whoilo_paf_summary(result_f_35_39, result_f_35_391, CI = NULL),
 result_lwr_f_40_44 = whoilo_paf_summary(result_f_40_44, result_f_40_441, sd_f_40_44, sd_f_40_441, CI = 0.025),
 result_upr_f_40_44 = whoilo_paf_summary(result_f_40_44, result_f_40_441, sd_f_40_44, sd_f_40_441, CI = 0.975),
 result_f_40_44 = whoilo_paf_summary(result_f_40_44, result_f_40_441, CI = NULL),
 result_lwr_f_45_49 = whoilo_paf_summary(result_f_45_49, result_f_45_491, sd_f_45_49, sd_f_45_491, CI = 0.025),
 result_upr_f_45_49 = whoilo_paf_summary(result_f_45_49, result_f_45_491, sd_f_45_49, sd_f_45_491, CI = 0.975),
 result_f_45_49 = whoilo_paf_summary(result_f_45_49, result_f_45_491, CI = NULL),
 result_lwr_f_50_54 = whoilo_paf_summary(result_f_50_54, result_f_50_541, sd_f_50_54, sd_f_50_541, CI = 0.025),
 result_upr_f_50_54 = whoilo_paf_summary(result_f_50_54, result_f_50_541, sd_f_50_54, sd_f_50_541, CI = 0.975),
 result_f_50_54 = whoilo_paf_summary(result_f_50_54, result_f_50_541, CI = NULL),
 result_lwr_f_55_59 = whoilo_paf_summary(result_f_55_59, result_f_55_591, sd_f_55_59, sd_f_55_591, CI = 0.025),
 result_upr_f_55_59 = whoilo_paf_summary(result_f_55_59, result_f_55_591, sd_f_55_59, sd_f_55_591, CI = 0.975),
 result_f_55_59 = whoilo_paf_summary(result_f_55_59, result_f_55_591, CI = NULL),
 result_lwr_f_60_64 = whoilo_paf_summary(result_f_60_64, result_f_60_641, sd_f_60_64, sd_f_60_641, CI = 0.025),
 result_upr_f_60_64 = whoilo_paf_summary(result_f_60_64, result_f_60_641, sd_f_60_64, sd_f_60_641, CI = 0.975),
 result_f_60_64 = whoilo_paf_summary(result_f_60_64, result_f_60_641, CI = NULL),
 result_lwr_f_65_69 = whoilo_paf_summary(result_f_65_69, result_f_65_691, sd_f_65_69, sd_f_65_691, CI = 0.025),
 result_upr_f_65_69 = whoilo_paf_summary(result_f_65_69, result_f_65_691, sd_f_65_69, sd_f_65_691, CI = 0.975),
 result_f_65_69 = whoilo_paf_summary(result_f_65_69, result_f_65_691, CI = NULL),
 result_lwr_f_70_74 = whoilo_paf_summary(result_f_70_74, result_f_70_741, sd_f_70_74, sd_f_70_741, CI = 0.025),
 result_upr_f_70_74 = whoilo_paf_summary(result_f_70_74, result_f_70_741, sd_f_70_74, sd_f_70_741, CI = 0.975),
 result_f_70_74 = whoilo_paf_summary(result_f_70_74, result_f_70_741, CI = NULL),
 result_lwr_f_75_79 = whoilo_paf_summary(result_f_75_79, result_f_75_791, sd_f_75_79, sd_f_75_791, CI = 0.025),
 result_upr_f_75_79 = whoilo_paf_summary(result_f_75_79, result_f_75_791, sd_f_75_79, sd_f_75_791, CI = 0.975),
 result_f_75_79 = whoilo_paf_summary(result_f_75_79, result_f_75_791, CI = NULL),
 result_lwr_f_80_84 = whoilo_paf_summary(result_f_80_84, result_f_80_841, sd_f_80_84, sd_f_80_841, CI = 0.025),
 result_upr_f_80_84 = whoilo_paf_summary(result_f_80_84, result_f_80_841, sd_f_80_84, sd_f_80_841, CI = 0.975),
 result_f_80_84 = whoilo_paf_summary(result_f_80_84, result_f_80_841, CI = NULL),
 result_lwr_f_85_89 = whoilo_paf_summary(result_f_85_89, result_f_85_891, sd_f_85_89, sd_f_85_891, CI = 0.025),
 result_upr_f_85_89 = whoilo_paf_summary(result_f_85_89, result_f_85_891, sd_f_85_89, sd_f_85_891, CI = 0.975),
 result_f_85_89 = whoilo_paf_summary(result_f_85_89, result_f_85_891, CI = NULL),
 result_lwr_f_90_94 = whoilo_paf_summary(result_f_90_94, result_f_90_941, sd_f_90_94, sd_f_90_941, CI = 0.025),
 result_upr_f_90_94 = whoilo_paf_summary(result_f_90_94, result_f_90_941, sd_f_90_94, sd_f_90_941, CI = 0.975),
 result_f_90_94 = whoilo_paf_summary(result_f_90_94, result_f_90_941, CI = NULL),
 result_lwr_f_95_ = whoilo_paf_summary(result_f_95_, result_f_95_1, sd_f_95_, sd_f_95_1, CI = 0.025),
 result_upr_f_95_ = whoilo_paf_summary(result_f_95_, result_f_95_1, sd_f_95_, sd_f_95_1, CI = 0.975),
 result_f_95_ = whoilo_paf_summary(result_f_95_, result_f_95_1, CI = NULL),
 result_lwr_m_15_19 = whoilo_paf_summary(result_m_15_19, result_m_15_191, sd_m_15_19, sd_m_15_191, CI = 0.025),
 result_upr_m_15_19 = whoilo_paf_summary(result_m_15_19, result_m_15_191, sd_m_15_19, sd_m_15_191, CI = 0.975),
 result_m_15_19 = whoilo_paf_summary(result_m_15_19, result_m_15_191, CI = NULL),
 result_lwr_m_20_24 = whoilo_paf_summary(result_m_20_24, result_m_20_241, sd_m_20_24, sd_m_20_241, CI = 0.025),
 result_upr_m_20_24 = whoilo_paf_summary(result_m_20_24, result_m_20_241, sd_m_20_24, sd_m_20_241, CI = 0.975),
 result_m_20_24 = whoilo_paf_summary(result_m_20_24, result_m_20_241, CI = NULL),
 result_lwr_m_25_29 = whoilo_paf_summary(result_m_25_29, result_m_25_291, sd_m_25_29, sd_m_25_291, CI = 0.025),
 result_upr_m_25_29 = whoilo_paf_summary(result_m_25_29, result_m_25_291, sd_m_25_29, sd_m_25_291, CI = 0.975),
 result_m_25_29 = whoilo_paf_summary(result_m_25_29, result_m_25_291, CI = NULL),
 result_lwr_m_30_34 = whoilo_paf_summary(result_m_30_34, result_m_30_341, sd_m_30_34, sd_m_30_341, CI = 0.025),
 result_upr_m_30_34 = whoilo_paf_summary(result_m_30_34, result_m_30_341, sd_m_30_34, sd_m_30_341, CI = 0.975),
 result_m_30_34 = whoilo_paf_summary(result_m_30_34, result_m_30_341, CI = NULL),
 result_lwr_m_35_39 = whoilo_paf_summary(result_m_35_39, result_m_35_391, sd_m_35_39, sd_m_35_391, CI = 0.025),
 result_upr_m_35_39 = whoilo_paf_summary(result_m_35_39, result_m_35_391, sd_m_35_39, sd_m_35_391, CI = 0.975),
 result_m_35_39 = whoilo_paf_summary(result_m_35_39, result_m_35_391, CI = NULL),
 result_lwr_m_40_44 = whoilo_paf_summary(result_m_40_44, result_m_40_441, sd_m_40_44, sd_m_40_441, CI = 0.025),
 result_upr_m_40_44 = whoilo_paf_summary(result_m_40_44, result_m_40_441, sd_m_40_44, sd_m_40_441, CI = 0.975),
 result_m_40_44 = whoilo_paf_summary(result_m_40_44, result_m_40_441, CI = NULL),
 result_lwr_m_45_49 = whoilo_paf_summary(result_m_45_49, result_m_45_491, sd_m_45_49, sd_m_45_491, CI = 0.025),
 result_upr_m_45_49 = whoilo_paf_summary(result_m_45_49, result_m_45_491, sd_m_45_49, sd_m_45_491, CI = 0.975),
 result_m_45_49 = whoilo_paf_summary(result_m_45_49, result_m_45_491, CI = NULL),
 result_lwr_m_50_54 = whoilo_paf_summary(result_m_50_54, result_m_50_541, sd_m_50_54, sd_m_50_541, CI = 0.025),
 result_upr_m_50_54 = whoilo_paf_summary(result_m_50_54, result_m_50_541, sd_m_50_54, sd_m_50_541, CI = 0.975),
 result_m_50_54 = whoilo_paf_summary(result_m_50_54, result_m_50_541, CI = NULL),
 result_lwr_m_55_59 = whoilo_paf_summary(result_m_55_59, result_m_55_591, sd_m_55_59, sd_m_55_591, CI = 0.025),
 result_upr_m_55_59 = whoilo_paf_summary(result_m_55_59, result_m_55_591, sd_m_55_59, sd_m_55_591, CI = 0.975),
 result_m_55_59 = whoilo_paf_summary(result_m_55_59, result_m_55_591, CI = NULL),
 result_lwr_m_60_64 = whoilo_paf_summary(result_m_60_64, result_m_60_641, sd_m_60_64, sd_m_60_641, CI = 0.025),
 result_upr_m_60_64 = whoilo_paf_summary(result_m_60_64, result_m_60_641, sd_m_60_64, sd_m_60_641, CI = 0.975),
 result_m_60_64 = whoilo_paf_summary(result_m_60_64, result_m_60_641, CI = NULL),
 result_lwr_m_65_69 = whoilo_paf_summary(result_m_65_69, result_m_65_691, sd_m_65_69, sd_m_65_691, CI = 0.025),
 result_upr_m_65_69 = whoilo_paf_summary(result_m_65_69, result_m_65_691, sd_m_65_69, sd_m_65_691, CI = 0.975),
 result_m_65_69 = whoilo_paf_summary(result_m_65_69, result_m_65_691, CI = NULL),
 result_lwr_m_70_74 = whoilo_paf_summary(result_m_70_74, result_m_70_741, sd_m_70_74, sd_m_70_741, CI = 0.025),
 result_upr_m_70_74 = whoilo_paf_summary(result_m_70_74, result_m_70_741, sd_m_70_74, sd_m_70_741, CI = 0.975),
 result_m_70_74 = whoilo_paf_summary(result_m_70_74, result_m_70_741, CI = NULL),
 result_lwr_m_75_79 = whoilo_paf_summary(result_m_75_79, result_m_75_791, sd_m_75_79, sd_m_75_791, CI = 0.025),
 result_upr_m_75_79 = whoilo_paf_summary(result_m_75_79, result_m_75_791, sd_m_75_79, sd_m_75_791, CI = 0.975),
 result_m_75_79 = whoilo_paf_summary(result_m_75_79, result_m_75_791, CI = NULL),
 result_lwr_m_80_84 = whoilo_paf_summary(result_m_80_84, result_m_80_841, sd_m_80_84, sd_m_80_841, CI = 0.025),
 result_upr_m_80_84 = whoilo_paf_summary(result_m_80_84, result_m_80_841, sd_m_80_84, sd_m_80_841, CI = 0.975),
 result_m_80_84 = whoilo_paf_summary(result_m_80_84, result_m_80_841, CI = NULL),
 result_lwr_m_85_89 = whoilo_paf_summary(result_m_85_89, result_m_85_891, sd_m_85_89, sd_m_85_891, CI = 0.025),
 result_upr_m_85_89 = whoilo_paf_summary(result_m_85_89, result_m_85_891, sd_m_85_89, sd_m_85_891, CI = 0.975),
 result_m_85_89 = whoilo_paf_summary(result_m_85_89, result_m_85_891, CI = NULL),
 result_lwr_m_90_94 = whoilo_paf_summary(result_m_90_94, result_m_90_941, sd_m_90_94, sd_m_90_941, CI = 0.025),
 result_upr_m_90_94 = whoilo_paf_summary(result_m_90_94, result_m_90_941, sd_m_90_94, sd_m_90_941, CI = 0.975),
 result_m_90_94 = whoilo_paf_summary(result_m_90_94, result_m_90_941, CI = NULL),
 result_lwr_m_95_ = whoilo_paf_summary(result_m_95_, result_m_95_1, sd_m_95_, sd_m_95_1, CI = 0.025),
 result_upr_m_95_ = whoilo_paf_summary(result_m_95_, result_m_95_1, sd_m_95_, sd_m_95_1, CI = 0.975),
 result_m_95_ = whoilo_paf_summary(result_m_95_, result_m_95_1, CI = NULL)) %>%
 ungroup() %>%
 mutate(sd_f_15_19 = (result_upr_f_15_19 - result_lwr_f_15_19)/2/1.96,
 sd_f_20_24 = (result_upr_f_20_24 - result_lwr_f_20_24)/2/1.96,
 sd_f_25_29 = (result_upr_f_25_29 - result_lwr_f_25_29)/2/1.96,
 sd_f_30_34 = (result_upr_f_30_34 - result_lwr_f_30_34)/2/1.96,
 sd_f_35_39 = (result_upr_f_35_39 - result_lwr_f_35_39)/2/1.96,
 sd_f_40_44 = (result_upr_f_40_44 - result_lwr_f_40_44)/2/1.96,
 sd_f_45_49 = (result_upr_f_45_49 - result_lwr_f_45_49)/2/1.96,
 sd_f_50_54 = (result_upr_f_50_54 - result_lwr_f_50_54)/2/1.96,
 sd_f_55_59 = (result_upr_f_55_59 - result_lwr_f_55_59)/2/1.96,
 sd_f_60_64 = (result_upr_f_60_64 - result_lwr_f_60_64)/2/1.96,
 sd_f_65_69 = (result_upr_f_65_69 - result_lwr_f_65_69)/2/1.96,
 sd_f_70_74 = (result_upr_f_70_74 - result_lwr_f_70_74)/2/1.96,
 sd_f_75_79 = (result_upr_f_75_79 - result_lwr_f_75_79)/2/1.96,
 sd_f_80_84 = (result_upr_f_80_84 - result_lwr_f_80_84)/2/1.96,
 sd_f_85_89 = (result_upr_f_85_89 - result_lwr_f_85_89)/2/1.96,
 sd_f_90_94 = (result_upr_f_90_94 - result_lwr_f_90_94)/2/1.96,
 sd_f_95_ = (result_upr_f_95_ - result_lwr_f_95_)/2/1.96,
 sd_m_15_19 = (result_upr_m_15_19 - result_lwr_m_15_19)/2/1.96,
 sd_m_20_24 = (result_upr_m_20_24 - result_lwr_m_20_24)/2/1.96,
 sd_m_25_29 = (result_upr_m_25_29 - result_lwr_m_25_29)/2/1.96,
 sd_m_30_34 = (result_upr_m_30_34 - result_lwr_m_30_34)/2/1.96,
 sd_m_35_39 = (result_upr_m_35_39 - result_lwr_m_35_39)/2/1.96,
 sd_m_40_44 = (result_upr_m_40_44 - result_lwr_m_40_44)/2/1.96,
 sd_m_45_49 = (result_upr_m_45_49 - result_lwr_m_45_49)/2/1.96,
 sd_m_50_54 = (result_upr_m_50_54 - result_lwr_m_50_54)/2/1.96,
 sd_m_55_59 = (result_upr_m_55_59 - result_lwr_m_55_59)/2/1.96,
 sd_m_60_64 = (result_upr_m_60_64 - result_lwr_m_60_64)/2/1.96,
 sd_m_65_69 = (result_upr_m_65_69 - result_lwr_m_65_69)/2/1.96,
 sd_m_70_74 = (result_upr_m_70_74 - result_lwr_m_70_74)/2/1.96,
 sd_m_75_79 = (result_upr_m_75_79 - result_lwr_m_75_79)/2/1.96,
 sd_m_80_84 = (result_upr_m_80_84 - result_lwr_m_80_84)/2/1.96,
 sd_m_85_89 = (result_upr_m_85_89 - result_lwr_m_85_89)/2/1.96,
 sd_m_90_94 = (result_upr_m_90_94 - result_lwr_m_90_94)/2/1.96,
 sd_m_95_ = (result_upr_m_95_ - result_lwr_m_95_)/2/1.96) %>%
 select(result_f_15_19, sd_f_15_19, result_lwr_f_15_19, result_upr_f_15_19,
 result_f_20_24, sd_f_20_24, result_lwr_f_20_24, result_upr_f_20_24,
 result_f_25_29, sd_f_25_29, result_lwr_f_25_29, result_upr_f_25_29,
 result_f_30_34, sd_f_30_34, result_lwr_f_30_34, result_upr_f_30_34,
 result_f_35_39, sd_f_35_39, result_lwr_f_35_39, result_upr_f_35_39,
 result_f_40_44, sd_f_40_44, result_lwr_f_40_44, result_upr_f_40_44,
 result_f_45_49, sd_f_45_49, result_lwr_f_45_49, result_upr_f_45_49,
 result_f_50_54, sd_f_50_54, result_lwr_f_50_54, result_upr_f_50_54,
 result_f_55_59, sd_f_55_59, result_lwr_f_55_59, result_upr_f_55_59,
 result_f_60_64, sd_f_60_64, result_lwr_f_60_64, result_upr_f_60_64,
 result_f_65_69, sd_f_65_69, result_lwr_f_65_69, result_upr_f_65_69,
 result_f_70_74, sd_f_70_74, result_lwr_f_70_74, result_upr_f_70_74,
 result_f_75_79, sd_f_75_79, result_lwr_f_75_79, result_upr_f_75_79,
 result_f_80_84, sd_f_80_84, result_lwr_f_80_84, result_upr_f_80_84,
 result_f_85_89, sd_f_85_89, result_lwr_f_85_89, result_upr_f_85_89,
 result_f_90_94, sd_f_90_94, result_lwr_f_90_94, result_upr_f_90_94,
 result_f_95_, sd_f_95_, result_lwr_f_95_, result_upr_f_95_,
 result_m_15_19, sd_m_15_19, result_lwr_m_15_19, result_upr_m_15_19,
 result_m_20_24, sd_m_20_24, result_lwr_m_20_24, result_upr_m_20_24,
 result_m_25_29, sd_m_25_29, result_lwr_m_25_29, result_upr_m_25_29,
 result_m_30_34, sd_m_30_34, result_lwr_m_30_34, result_upr_m_30_34,
 result_m_35_39, sd_m_35_39, result_lwr_m_35_39, result_upr_m_35_39,
 result_m_40_44, sd_m_40_44, result_lwr_m_40_44, result_upr_m_40_44,
 result_m_45_49, sd_m_45_49, result_lwr_m_45_49, result_upr_m_45_49,
 result_m_50_54, sd_m_50_54, result_lwr_m_50_54, result_upr_m_50_54,
 result_m_55_59, sd_m_55_59, result_lwr_m_55_59, result_upr_m_55_59,
 result_m_60_64, sd_m_60_64, result_lwr_m_60_64, result_upr_m_60_64,
 result_m_65_69, sd_m_65_69, result_lwr_m_65_69, result_upr_m_65_69,
 result_m_70_74, sd_m_70_74, result_lwr_m_70_74, result_upr_m_70_74,
 result_m_75_79, sd_m_75_79, result_lwr_m_75_79, result_upr_m_75_79,
 result_m_80_84, sd_m_80_84, result_lwr_m_80_84, result_upr_m_80_84,
 result_m_85_89, sd_m_85_89, result_lwr_m_85_89, result_upr_m_85_89,
 result_m_90_94, sd_m_90_94, result_lwr_m_90_94, result_upr_m_90_94,
 result_m_95_, sd_m_95_, result_lwr_m_95_, result_upr_m_95_)
 if (shape == "long") {
 dths_stroke_sexage_global <- dths_stroke_sexage_global

 }
 }
 if (outcome %in% c("h0", "h1", "h2", "h3", "h4", "h5")){
 dths_stroke_sexage_global <- df_in %>%
 filter(outcome == !!outcome,
 year == !!year,
 cause == !!cause) %>%
 arrange(region, country_abbrev, year, sex, ilo_age_bands_5yr) %>%
 mutate(sd = (result_upr - result_lwr)/2/1.96) %>%
 group_by(sex, ilo_age_bands_5yr) %>%
 left_join(who_ilo_long_populations, by = c("region", "country_abbrev", "year", "sex", "ilo_age_bands_5yr" = "age_bands_5yr")) %>%
 mutate(result = result*population,
 sd = sd*population) %>%
 drop_na() %>%
 summarise(result = sum(result),
 sd = sqrt(sum((sd)^2)),
 population = sum(population)) %>%
 mutate(result_lwr = qnorm(p = 0.025, mean = result, sd = sd)/ population,
 result_upr = qnorm(p = 0.975, mean = result, sd = sd)/ population,
 result = result / population,
 sd = sd/population) %>%
 select(-population) %>%
 ungroup %>%
 pivot_wider(names_from = sex:ilo_age_bands_5yr, values_from = result:result_upr) %>%
 select(result_f_15_19, sd_f_15_19, result_lwr_f_15_19, result_upr_f_15_19,
 result_f_20_24, sd_f_20_24, result_lwr_f_20_24, result_upr_f_20_24,
 result_f_25_29, sd_f_25_29, result_lwr_f_25_29, result_upr_f_25_29,
 result_f_30_34, sd_f_30_34, result_lwr_f_30_34, result_upr_f_30_34,
 result_f_35_39, sd_f_35_39, result_lwr_f_35_39, result_upr_f_35_39,
 result_f_40_44, sd_f_40_44, result_lwr_f_40_44, result_upr_f_40_44,
 result_f_45_49, sd_f_45_49, result_lwr_f_45_49, result_upr_f_45_49,
 result_f_50_54, sd_f_50_54, result_lwr_f_50_54, result_upr_f_50_54,
 result_f_55_59, sd_f_55_59, result_lwr_f_55_59, result_upr_f_55_59,
 result_f_60_64, sd_f_60_64, result_lwr_f_60_64, result_upr_f_60_64,
 result_f_65_69, sd_f_65_69, result_lwr_f_65_69, result_upr_f_65_69,
 result_f_70_74, sd_f_70_74, result_lwr_f_70_74, result_upr_f_70_74,
 result_f_75_79, sd_f_75_79, result_lwr_f_75_79, result_upr_f_75_79,
 result_f_80_84, sd_f_80_84, result_lwr_f_80_84, result_upr_f_80_84,
 result_f_85_89, sd_f_85_89, result_lwr_f_85_89, result_upr_f_85_89,
 result_f_90_94, sd_f_90_94, result_lwr_f_90_94, result_upr_f_90_94,
 result_f_95_, sd_f_95_, result_lwr_f_95_, result_upr_f_95_,
 result_m_15_19, sd_m_15_19, result_lwr_m_15_19, result_upr_m_15_19,
 result_m_20_24, sd_m_20_24, result_lwr_m_20_24, result_upr_m_20_24,
 result_m_25_29, sd_m_25_29, result_lwr_m_25_29, result_upr_m_25_29,
 result_m_30_34, sd_m_30_34, result_lwr_m_30_34, result_upr_m_30_34,
 result_m_35_39, sd_m_35_39, result_lwr_m_35_39, result_upr_m_35_39,
 result_m_40_44, sd_m_40_44, result_lwr_m_40_44, result_upr_m_40_44,
 result_m_45_49, sd_m_45_49, result_lwr_m_45_49, result_upr_m_45_49,
 result_m_50_54, sd_m_50_54, result_lwr_m_50_54, result_upr_m_50_54,
 result_m_55_59, sd_m_55_59, result_lwr_m_55_59, result_upr_m_55_59,
 result_m_60_64, sd_m_60_64, result_lwr_m_60_64, result_upr_m_60_64,
 result_m_65_69, sd_m_65_69, result_lwr_m_65_69, result_upr_m_65_69,
 result_m_70_74, sd_m_70_74, result_lwr_m_70_74, result_upr_m_70_74,
 result_m_75_79, sd_m_75_79, result_lwr_m_75_79, result_upr_m_75_79,
 result_m_80_84, sd_m_80_84, result_lwr_m_80_84, result_upr_m_80_84,
 result_m_85_89, sd_m_85_89, result_lwr_m_85_89, result_upr_m_85_89,
 result_m_90_94, sd_m_90_94, result_lwr_m_90_94, result_upr_m_90_94,
 result_m_95_, sd_m_95_, result_lwr_m_95_, result_upr_m_95_)
 }
 if (outcome %in% c("h0_number", "h1_number", "h2_number", "h3_number", "h4_number", "h5_number")){
 outcome2 <- str_sub(outcome, end = -8L)
 dths_stroke_sexage_global <- df_in %>%
 filter(outcome == !!outcome2,
 year == !!year,
 cause == !!cause) %>%
 arrange(region, country_abbrev, year, sex, ilo_age_bands_5yr) %>%
 mutate(sd = (result_upr - result_lwr)/2/1.96) %>%
 group_by(sex, ilo_age_bands_5yr) %>%
 left_join(who_ilo_long_populations, by = c("region", "country_abbrev", "year", "sex", "ilo_age_bands_5yr" = "age_bands_5yr")) %>%
 mutate(population = population * 1000) %>%
 mutate(result = result*population,
 sd = sd*population) %>%
 drop_na() %>%
 summarise(result = sum(result),
 sd = sqrt(sum((sd)^2)),
 population = sum(population)) %>%
 mutate(result_lwr = qnorm(p = 0.025, mean = result, sd = sd),
 result_upr = qnorm(p = 0.975, mean = result, sd = sd),
 result = result,
 sd = sd) %>%
 select(-population) %>%
 ungroup %>%
 pivot_wider(names_from = sex:ilo_age_bands_5yr, values_from = result:result_upr) %>%
 select(result_f_15_19, sd_f_15_19, result_lwr_f_15_19, result_upr_f_15_19,
 result_f_20_24, sd_f_20_24, result_lwr_f_20_24, result_upr_f_20_24,
 result_f_25_29, sd_f_25_29, result_lwr_f_25_29, result_upr_f_25_29,
 result_f_30_34, sd_f_30_34, result_lwr_f_30_34, result_upr_f_30_34,
 result_f_35_39, sd_f_35_39, result_lwr_f_35_39, result_upr_f_35_39,
 result_f_40_44, sd_f_40_44, result_lwr_f_40_44, result_upr_f_40_44,
 result_f_45_49, sd_f_45_49, result_lwr_f_45_49, result_upr_f_45_49,
 result_f_50_54, sd_f_50_54, result_lwr_f_50_54, result_upr_f_50_54,
 result_f_55_59, sd_f_55_59, result_lwr_f_55_59, result_upr_f_55_59,
 result_f_60_64, sd_f_60_64, result_lwr_f_60_64, result_upr_f_60_64,
 result_f_65_69, sd_f_65_69, result_lwr_f_65_69, result_upr_f_65_69,
 result_f_70_74, sd_f_70_74, result_lwr_f_70_74, result_upr_f_70_74,
 result_f_75_79, sd_f_75_79, result_lwr_f_75_79, result_upr_f_75_79,
 result_f_80_84, sd_f_80_84, result_lwr_f_80_84, result_upr_f_80_84,
 result_f_85_89, sd_f_85_89, result_lwr_f_85_89, result_upr_f_85_89,
 result_f_90_94, sd_f_90_94, result_lwr_f_90_94, result_upr_f_90_94,
 result_f_95_, sd_f_95_, result_lwr_f_95_, result_upr_f_95_,
 result_m_15_19, sd_m_15_19, result_lwr_m_15_19, result_upr_m_15_19,
 result_m_20_24, sd_m_20_24, result_lwr_m_20_24, result_upr_m_20_24,
 result_m_25_29, sd_m_25_29, result_lwr_m_25_29, result_upr_m_25_29,
 result_m_30_34, sd_m_30_34, result_lwr_m_30_34, result_upr_m_30_34,
 result_m_35_39, sd_m_35_39, result_lwr_m_35_39, result_upr_m_35_39,
 result_m_40_44, sd_m_40_44, result_lwr_m_40_44, result_upr_m_40_44,
 result_m_45_49, sd_m_45_49, result_lwr_m_45_49, result_upr_m_45_49,
 result_m_50_54, sd_m_50_54, result_lwr_m_50_54, result_upr_m_50_54,
 result_m_55_59, sd_m_55_59, result_lwr_m_55_59, result_upr_m_55_59,
 result_m_60_64, sd_m_60_64, result_lwr_m_60_64, result_upr_m_60_64,
 result_m_65_69, sd_m_65_69, result_lwr_m_65_69, result_upr_m_65_69,
 result_m_70_74, sd_m_70_74, result_lwr_m_70_74, result_upr_m_70_74,
 result_m_75_79, sd_m_75_79, result_lwr_m_75_79, result_upr_m_75_79,
 result_m_80_84, sd_m_80_84, result_lwr_m_80_84, result_upr_m_80_84,
 result_m_85_89, sd_m_85_89, result_lwr_m_85_89, result_upr_m_85_89,
 result_m_90_94, sd_m_90_94, result_lwr_m_90_94, result_upr_m_90_94,
 result_m_95_, sd_m_95_, result_lwr_m_95_, result_upr_m_95_)
 }
 if (outcome %in% c("DTH", "DALY", "DTHenvelope", "DALYenvelope")) {
 dths_stroke_sexage_global <- df_in %>%
 filter(outcome == !!outcome,
 year == !!year,
 cause == !!cause) %>%
 arrange(region, country_abbrev, year, sex, ilo_age_bands_5yr) %>%
 mutate(sd = (result_upr - result_lwr)/2/1.96) %>%
 drop_na(region, result) %>%
 group_by(sex, ilo_age_bands_5yr) %>%
 summarise(result = sum(result),
 sd = sqrt(sum((sd)^2))) %>%
 mutate(result_lwr = qnorm(p = 0.025, mean = result, sd = sd),
 result_upr = qnorm(p = 0.975, mean = result, sd = sd)) %>%
 ungroup %>%
 pivot_wider(names_from = sex:ilo_age_bands_5yr, values_from = result:result_upr) %>%
 select(result_f_15_19, sd_f_15_19, result_lwr_f_15_19, result_upr_f_15_19,
 result_f_20_24, sd_f_20_24, result_lwr_f_20_24, result_upr_f_20_24,
 result_f_25_29, sd_f_25_29, result_lwr_f_25_29, result_upr_f_25_29,
 result_f_30_34, sd_f_30_34, result_lwr_f_30_34, result_upr_f_30_34,
 result_f_35_39, sd_f_35_39, result_lwr_f_35_39, result_upr_f_35_39,
 result_f_40_44, sd_f_40_44, result_lwr_f_40_44, result_upr_f_40_44,
 result_f_45_49, sd_f_45_49, result_lwr_f_45_49, result_upr_f_45_49,
 result_f_50_54, sd_f_50_54, result_lwr_f_50_54, result_upr_f_50_54,
 result_f_55_59, sd_f_55_59, result_lwr_f_55_59, result_upr_f_55_59,
 result_f_60_64, sd_f_60_64, result_lwr_f_60_64, result_upr_f_60_64,
 result_f_65_69, sd_f_65_69, result_lwr_f_65_69, result_upr_f_65_69,
 result_f_70_74, sd_f_70_74, result_lwr_f_70_74, result_upr_f_70_74,
 result_f_75_79, sd_f_75_79, result_lwr_f_75_79, result_upr_f_75_79,
 result_f_80_84, sd_f_80_84, result_lwr_f_80_84, result_upr_f_80_84,
 result_f_85_89, sd_f_85_89, result_lwr_f_85_89, result_upr_f_85_89,
 result_f_90_94, sd_f_90_94, result_lwr_f_90_94, result_upr_f_90_94,
 result_f_95_, sd_f_95_, result_lwr_f_95_, result_upr_f_95_,
 result_m_15_19, sd_m_15_19, result_lwr_m_15_19, result_upr_m_15_19,
 result_m_20_24, sd_m_20_24, result_lwr_m_20_24, result_upr_m_20_24,
 result_m_25_29, sd_m_25_29, result_lwr_m_25_29, result_upr_m_25_29,
 result_m_30_34, sd_m_30_34, result_lwr_m_30_34, result_upr_m_30_34,
 result_m_35_39, sd_m_35_39, result_lwr_m_35_39, result_upr_m_35_39,
 result_m_40_44, sd_m_40_44, result_lwr_m_40_44, result_upr_m_40_44,
 result_m_45_49, sd_m_45_49, result_lwr_m_45_49, result_upr_m_45_49,
 result_m_50_54, sd_m_50_54, result_lwr_m_50_54, result_upr_m_50_54,
 result_m_55_59, sd_m_55_59, result_lwr_m_55_59, result_upr_m_55_59,
 result_m_60_64, sd_m_60_64, result_lwr_m_60_64, result_upr_m_60_64,
 result_m_65_69, sd_m_65_69, result_lwr_m_65_69, result_upr_m_65_69,
 result_m_70_74, sd_m_70_74, result_lwr_m_70_74, result_upr_m_70_74,
 result_m_75_79, sd_m_75_79, result_lwr_m_75_79, result_upr_m_75_79,
 result_m_80_84, sd_m_80_84, result_lwr_m_80_84, result_upr_m_80_84,
 result_m_85_89, sd_m_85_89, result_lwr_m_85_89, result_upr_m_85_89,
 result_m_90_94, sd_m_90_94, result_lwr_m_90_94, result_upr_m_90_94,
 result_m_95_, sd_m_95_, result_lwr_m_95_, result_upr_m_95_)
 }
 if (outcome %in% c("DTHS", "DALYS")) {
 outcome2 <- str_sub(outcome, end = -2L)
 dths_stroke_sexage_global <- df_in %>%
 filter(outcome == !!outcome2,
 year == !!year,
 cause %in% c("stroke_h5", "ihd_h5")) %>%
 arrange(region, country_abbrev, year, sex, ilo_age_bands_5yr) %>%
 mutate(sd = (result_upr - result_lwr)/2/1.96) %>%
 drop_na() %>%
 group_by(sex, ilo_age_bands_5yr) %>%
 summarise(result = sum(result),
 sd = sqrt(sum((sd)^2))) %>%
 mutate(result_lwr = qnorm(p = 0.025, mean = result, sd = sd),
 result_upr = qnorm(p = 0.975, mean = result, sd = sd)) %>%
 ungroup %>%
 pivot_wider(names_from = sex:ilo_age_bands_5yr, values_from = result:result_upr) %>%
 select(result_f_15_19, sd_f_15_19, result_lwr_f_15_19, result_upr_f_15_19,
 result_f_20_24, sd_f_20_24, result_lwr_f_20_24, result_upr_f_20_24,
 result_f_25_29, sd_f_25_29, result_lwr_f_25_29, result_upr_f_25_29,
 result_f_30_34, sd_f_30_34, result_lwr_f_30_34, result_upr_f_30_34,
 result_f_35_39, sd_f_35_39, result_lwr_f_35_39, result_upr_f_35_39,
 result_f_40_44, sd_f_40_44, result_lwr_f_40_44, result_upr_f_40_44,
 result_f_45_49, sd_f_45_49, result_lwr_f_45_49, result_upr_f_45_49,
 result_f_50_54, sd_f_50_54, result_lwr_f_50_54, result_upr_f_50_54,
 result_f_55_59, sd_f_55_59, result_lwr_f_55_59, result_upr_f_55_59,
 result_f_60_64, sd_f_60_64, result_lwr_f_60_64, result_upr_f_60_64,
 result_f_65_69, sd_f_65_69, result_lwr_f_65_69, result_upr_f_65_69,
 result_f_70_74, sd_f_70_74, result_lwr_f_70_74, result_upr_f_70_74,
 result_f_75_79, sd_f_75_79, result_lwr_f_75_79, result_upr_f_75_79,
 result_f_80_84, sd_f_80_84, result_lwr_f_80_84, result_upr_f_80_84,
 result_f_85_89, sd_f_85_89, result_lwr_f_85_89, result_upr_f_85_89,
 result_f_90_94, sd_f_90_94, result_lwr_f_90_94, result_upr_f_90_94,
 result_f_95_, sd_f_95_, result_lwr_f_95_, result_upr_f_95_,
 result_m_15_19, sd_m_15_19, result_lwr_m_15_19, result_upr_m_15_19,
 result_m_20_24, sd_m_20_24, result_lwr_m_20_24, result_upr_m_20_24,
 result_m_25_29, sd_m_25_29, result_lwr_m_25_29, result_upr_m_25_29,
 result_m_30_34, sd_m_30_34, result_lwr_m_30_34, result_upr_m_30_34,
 result_m_35_39, sd_m_35_39, result_lwr_m_35_39, result_upr_m_35_39,
 result_m_40_44, sd_m_40_44, result_lwr_m_40_44, result_upr_m_40_44,
 result_m_45_49, sd_m_45_49, result_lwr_m_45_49, result_upr_m_45_49,
 result_m_50_54, sd_m_50_54, result_lwr_m_50_54, result_upr_m_50_54,
 result_m_55_59, sd_m_55_59, result_lwr_m_55_59, result_upr_m_55_59,
 result_m_60_64, sd_m_60_64, result_lwr_m_60_64, result_upr_m_60_64,
 result_m_65_69, sd_m_65_69, result_lwr_m_65_69, result_upr_m_65_69,
 result_m_70_74, sd_m_70_74, result_lwr_m_70_74, result_upr_m_70_74,
 result_m_75_79, sd_m_75_79, result_lwr_m_75_79, result_upr_m_75_79,
 result_m_80_84, sd_m_80_84, result_lwr_m_80_84, result_upr_m_80_84,
 result_m_85_89, sd_m_85_89, result_lwr_m_85_89, result_upr_m_85_89,
 result_m_90_94, sd_m_90_94, result_lwr_m_90_94, result_upr_m_90_94,
 result_m_95_, sd_m_95_, result_lwr_m_95_, result_upr_m_95_)
 }
 if (outcome %in% c("h45")){
 dths_stroke_sexage_global <- df_in %>%
 filter(outcome %in% c("h4", "h5"),
 year == !!year,
 cause == !!cause) %>%
 arrange(region, country_abbrev, year, sex, ilo_age_bands_5yr) %>%
 mutate(sd = (result_upr - result_lwr)/2/1.96) %>%
 group_by(sex, ilo_age_bands_5yr) %>%
 left_join(who_ilo_long_populations, by = c("region", "country_abbrev", "year", "sex", "ilo_age_bands_5yr" = "age_bands_5yr")) %>%
 mutate(result = result*population,
 sd = sd*population) %>%
 drop_na() %>%
 summarise(result = sum(result),
 sd = sqrt(sum((sd)^2)),
 population = sum(population)/2) %>%
 mutate(result_lwr = qnorm(p = 0.025, mean = result, sd = sd)/ population,
 result_upr = qnorm(p = 0.975, mean = result, sd = sd)/ population,
 result = result / population,
 sd = sd/population) %>%
 select(-population) %>%
 ungroup %>%
 pivot_wider(names_from = sex:ilo_age_bands_5yr, values_from = result:result_upr) %>%
 select(result_f_15_19, sd_f_15_19, result_lwr_f_15_19, result_upr_f_15_19,
 result_f_20_24, sd_f_20_24, result_lwr_f_20_24, result_upr_f_20_24,
 result_f_25_29, sd_f_25_29, result_lwr_f_25_29, result_upr_f_25_29,
 result_f_30_34, sd_f_30_34, result_lwr_f_30_34, result_upr_f_30_34,
 result_f_35_39, sd_f_35_39, result_lwr_f_35_39, result_upr_f_35_39,
 result_f_40_44, sd_f_40_44, result_lwr_f_40_44, result_upr_f_40_44,
 result_f_45_49, sd_f_45_49, result_lwr_f_45_49, result_upr_f_45_49,
 result_f_50_54, sd_f_50_54, result_lwr_f_50_54, result_upr_f_50_54,
 result_f_55_59, sd_f_55_59, result_lwr_f_55_59, result_upr_f_55_59,
 result_f_60_64, sd_f_60_64, result_lwr_f_60_64, result_upr_f_60_64,
 result_f_65_69, sd_f_65_69, result_lwr_f_65_69, result_upr_f_65_69,
 result_f_70_74, sd_f_70_74, result_lwr_f_70_74, result_upr_f_70_74,
 result_f_75_79, sd_f_75_79, result_lwr_f_75_79, result_upr_f_75_79,
 result_f_80_84, sd_f_80_84, result_lwr_f_80_84, result_upr_f_80_84,
 result_f_85_89, sd_f_85_89, result_lwr_f_85_89, result_upr_f_85_89,
 result_f_90_94, sd_f_90_94, result_lwr_f_90_94, result_upr_f_90_94,
 result_f_95_, sd_f_95_, result_lwr_f_95_, result_upr_f_95_,
 result_m_15_19, sd_m_15_19, result_lwr_m_15_19, result_upr_m_15_19,
 result_m_20_24, sd_m_20_24, result_lwr_m_20_24, result_upr_m_20_24,
 result_m_25_29, sd_m_25_29, result_lwr_m_25_29, result_upr_m_25_29,
 result_m_30_34, sd_m_30_34, result_lwr_m_30_34, result_upr_m_30_34,
 result_m_35_39, sd_m_35_39, result_lwr_m_35_39, result_upr_m_35_39,
 result_m_40_44, sd_m_40_44, result_lwr_m_40_44, result_upr_m_40_44,
 result_m_45_49, sd_m_45_49, result_lwr_m_45_49, result_upr_m_45_49,
 result_m_50_54, sd_m_50_54, result_lwr_m_50_54, result_upr_m_50_54,
 result_m_55_59, sd_m_55_59, result_lwr_m_55_59, result_upr_m_55_59,
 result_m_60_64, sd_m_60_64, result_lwr_m_60_64, result_upr_m_60_64,
 result_m_65_69, sd_m_65_69, result_lwr_m_65_69, result_upr_m_65_69,
 result_m_70_74, sd_m_70_74, result_lwr_m_70_74, result_upr_m_70_74,
 result_m_75_79, sd_m_75_79, result_lwr_m_75_79, result_upr_m_75_79,
 result_m_80_84, sd_m_80_84, result_lwr_m_80_84, result_upr_m_80_84,
 result_m_85_89, sd_m_85_89, result_lwr_m_85_89, result_upr_m_85_89,
 result_m_90_94, sd_m_90_94, result_lwr_m_90_94, result_upr_m_90_94,
 result_m_95_, sd_m_95_, result_lwr_m_95_, result_upr_m_95_)
 }
 if (outcome %in% c("DTHrate")){
 outcome2 <- "DTH"
 dths_stroke_sexage_global <- df_in %>%
 filter(outcome == !!outcome2,
 year == !!year,
 cause == !!cause) %>%
 arrange(region, country_abbrev, year, sex, ilo_age_bands_5yr) %>%
 mutate(sd = (result_upr - result_lwr)/2/1.96) %>%
 group_by(sex, ilo_age_bands_5yr) %>%
 left_join(who_ilo_long_populations, by = c("region", "country_abbrev", "year", "sex", "ilo_age_bands_5yr" = "age_bands_5yr")) %>%
 drop_na() %>%
 summarise(result = sum(result),
 sd = sqrt(sum((sd)^2)),
 population = sum(population)) %>%
 mutate(population = population / 100,
 result = result / population,
 sd = sd / population,
 result_lwr = qnorm(p = 0.025, mean = result, sd = sd),
 result_upr = qnorm(p = 0.975, mean = result, sd = sd)) %>%
 select(-population) %>%
 ungroup %>%
 pivot_wider(names_from = sex:ilo_age_bands_5yr, values_from = result:result_upr) %>%
 select(result_f_15_19, sd_f_15_19, result_lwr_f_15_19, result_upr_f_15_19,
 result_f_20_24, sd_f_20_24, result_lwr_f_20_24, result_upr_f_20_24,
 result_f_25_29, sd_f_25_29, result_lwr_f_25_29, result_upr_f_25_29,
 result_f_30_34, sd_f_30_34, result_lwr_f_30_34, result_upr_f_30_34,
 result_f_35_39, sd_f_35_39, result_lwr_f_35_39, result_upr_f_35_39,
 result_f_40_44, sd_f_40_44, result_lwr_f_40_44, result_upr_f_40_44,
 result_f_45_49, sd_f_45_49, result_lwr_f_45_49, result_upr_f_45_49,
 result_f_50_54, sd_f_50_54, result_lwr_f_50_54, result_upr_f_50_54,
 result_f_55_59, sd_f_55_59, result_lwr_f_55_59, result_upr_f_55_59,
 result_f_60_64, sd_f_60_64, result_lwr_f_60_64, result_upr_f_60_64,
 result_f_65_69, sd_f_65_69, result_lwr_f_65_69, result_upr_f_65_69,
 result_f_70_74, sd_f_70_74, result_lwr_f_70_74, result_upr_f_70_74,
 result_f_75_79, sd_f_75_79, result_lwr_f_75_79, result_upr_f_75_79,
 result_f_80_84, sd_f_80_84, result_lwr_f_80_84, result_upr_f_80_84,
 result_f_85_89, sd_f_85_89, result_lwr_f_85_89, result_upr_f_85_89,
 result_f_90_94, sd_f_90_94, result_lwr_f_90_94, result_upr_f_90_94,
 result_f_95_, sd_f_95_, result_lwr_f_95_, result_upr_f_95_,
 result_m_15_19, sd_m_15_19, result_lwr_m_15_19, result_upr_m_15_19,
 result_m_20_24, sd_m_20_24, result_lwr_m_20_24, result_upr_m_20_24,
 result_m_25_29, sd_m_25_29, result_lwr_m_25_29, result_upr_m_25_29,
 result_m_30_34, sd_m_30_34, result_lwr_m_30_34, result_upr_m_30_34,
 result_m_35_39, sd_m_35_39, result_lwr_m_35_39, result_upr_m_35_39,
 result_m_40_44, sd_m_40_44, result_lwr_m_40_44, result_upr_m_40_44,
 result_m_45_49, sd_m_45_49, result_lwr_m_45_49, result_upr_m_45_49,
 result_m_50_54, sd_m_50_54, result_lwr_m_50_54, result_upr_m_50_54,
 result_m_55_59, sd_m_55_59, result_lwr_m_55_59, result_upr_m_55_59,
 result_m_60_64, sd_m_60_64, result_lwr_m_60_64, result_upr_m_60_64,
 result_m_65_69, sd_m_65_69, result_lwr_m_65_69, result_upr_m_65_69,
 result_m_70_74, sd_m_70_74, result_lwr_m_70_74, result_upr_m_70_74,
 result_m_75_79, sd_m_75_79, result_lwr_m_75_79, result_upr_m_75_79,
 result_m_80_84, sd_m_80_84, result_lwr_m_80_84, result_upr_m_80_84,
 result_m_85_89, sd_m_85_89, result_lwr_m_85_89, result_upr_m_85_89,
 result_m_90_94, sd_m_90_94, result_lwr_m_90_94, result_upr_m_90_94,
 result_m_95_, sd_m_95_, result_lwr_m_95_, result_upr_m_95_)
 }
 if (outcome %in% c("DALYrate")){
 outcome2 <- "DALY"
 dths_stroke_sexage_global <- df_in %>%
 filter(outcome == !!outcome2,
 year == !!year,
 cause == !!cause) %>%
 arrange(region, country_abbrev, year, sex, ilo_age_bands_5yr) %>%
 mutate(sd = (result_upr - result_lwr)/2/1.96) %>%
 group_by(sex, ilo_age_bands_5yr) %>%
 left_join(who_ilo_long_populations, by = c("region", "country_abbrev", "year", "sex", "ilo_age_bands_5yr" = "age_bands_5yr")) %>%
 drop_na() %>%
 summarise(result = sum(result),
 sd = sqrt(sum((sd)^2)),
 population = sum(population)) %>%
 mutate(population = population,
 result = result / population * 100000,
 sd = sd / population * 100000,
 result_lwr = qnorm(p = 0.025, mean = result, sd = sd),
 result_upr = qnorm(p = 0.975, mean = result, sd = sd)) %>%
 select(-population) %>%
 ungroup %>%
 pivot_wider(names_from = sex:ilo_age_bands_5yr, values_from = result:result_upr) %>%
 select(result_f_15_19, sd_f_15_19, result_lwr_f_15_19, result_upr_f_15_19,
 result_f_20_24, sd_f_20_24, result_lwr_f_20_24, result_upr_f_20_24,
 result_f_25_29, sd_f_25_29, result_lwr_f_25_29, result_upr_f_25_29,
 result_f_30_34, sd_f_30_34, result_lwr_f_30_34, result_upr_f_30_34,
 result_f_35_39, sd_f_35_39, result_lwr_f_35_39, result_upr_f_35_39,
 result_f_40_44, sd_f_40_44, result_lwr_f_40_44, result_upr_f_40_44,
 result_f_45_49, sd_f_45_49, result_lwr_f_45_49, result_upr_f_45_49,
 result_f_50_54, sd_f_50_54, result_lwr_f_50_54, result_upr_f_50_54,
 result_f_55_59, sd_f_55_59, result_lwr_f_55_59, result_upr_f_55_59,
 result_f_60_64, sd_f_60_64, result_lwr_f_60_64, result_upr_f_60_64,
 result_f_65_69, sd_f_65_69, result_lwr_f_65_69, result_upr_f_65_69,
 result_f_70_74, sd_f_70_74, result_lwr_f_70_74, result_upr_f_70_74,
 result_f_75_79, sd_f_75_79, result_lwr_f_75_79, result_upr_f_75_79,
 result_f_80_84, sd_f_80_84, result_lwr_f_80_84, result_upr_f_80_84,
 result_f_85_89, sd_f_85_89, result_lwr_f_85_89, result_upr_f_85_89,
 result_f_90_94, sd_f_90_94, result_lwr_f_90_94, result_upr_f_90_94,
 result_f_95_, sd_f_95_, result_lwr_f_95_, result_upr_f_95_,
 result_m_15_19, sd_m_15_19, result_lwr_m_15_19, result_upr_m_15_19,
 result_m_20_24, sd_m_20_24, result_lwr_m_20_24, result_upr_m_20_24,
 result_m_25_29, sd_m_25_29, result_lwr_m_25_29, result_upr_m_25_29,
 result_m_30_34, sd_m_30_34, result_lwr_m_30_34, result_upr_m_30_34,
 result_m_35_39, sd_m_35_39, result_lwr_m_35_39, result_upr_m_35_39,
 result_m_40_44, sd_m_40_44, result_lwr_m_40_44, result_upr_m_40_44,
 result_m_45_49, sd_m_45_49, result_lwr_m_45_49, result_upr_m_45_49,
 result_m_50_54, sd_m_50_54, result_lwr_m_50_54, result_upr_m_50_54,
 result_m_55_59, sd_m_55_59, result_lwr_m_55_59, result_upr_m_55_59,
 result_m_60_64, sd_m_60_64, result_lwr_m_60_64, result_upr_m_60_64,
 result_m_65_69, sd_m_65_69, result_lwr_m_65_69, result_upr_m_65_69,
 result_m_70_74, sd_m_70_74, result_lwr_m_70_74, result_upr_m_70_74,
 result_m_75_79, sd_m_75_79, result_lwr_m_75_79, result_upr_m_75_79,
 result_m_80_84, sd_m_80_84, result_lwr_m_80_84, result_upr_m_80_84,
 result_m_85_89, sd_m_85_89, result_lwr_m_85_89, result_upr_m_85_89,
 result_m_90_94, sd_m_90_94, result_lwr_m_90_94, result_upr_m_90_94,
 result_m_95_, sd_m_95_, result_lwr_m_95_, result_upr_m_95_)
 }
 if (outcome %in% c("population")){
 dths_stroke_sexage_global <- who_ilo_long_populations %>%
 filter(age_bands_5yr %in% c("15_19", "20_24", "25_29", "30_34", "35_39", "40_44", "45_49", "50_54", "55_59", "60_64", "65_69", "70_74", "75_79", "80_84", "85_89", "90_94", "95_"),
 year == !!year) %>%
 arrange(region, country_abbrev, year, sex, age_bands_5yr) %>%
 drop_na() %>%
 group_by(sex, age_bands_5yr) %>%
 summarise(result = sum(population)) %>%
 mutate(sd = NA,
 result_lwr = NA,
 result_upr = NA) %>%
 ungroup %>%
 pivot_wider(names_from = sex:age_bands_5yr, values_from = result:result_upr) %>%
 select(result_f_15_19, sd_f_15_19, result_lwr_f_15_19, result_upr_f_15_19,
 result_f_20_24, sd_f_20_24, result_lwr_f_20_24, result_upr_f_20_24,
 result_f_25_29, sd_f_25_29, result_lwr_f_25_29, result_upr_f_25_29,
 result_f_30_34, sd_f_30_34, result_lwr_f_30_34, result_upr_f_30_34,
 result_f_35_39, sd_f_35_39, result_lwr_f_35_39, result_upr_f_35_39,
 result_f_40_44, sd_f_40_44, result_lwr_f_40_44, result_upr_f_40_44,
 result_f_45_49, sd_f_45_49, result_lwr_f_45_49, result_upr_f_45_49,
 result_f_50_54, sd_f_50_54, result_lwr_f_50_54, result_upr_f_50_54,
 result_f_55_59, sd_f_55_59, result_lwr_f_55_59, result_upr_f_55_59,
 result_f_60_64, sd_f_60_64, result_lwr_f_60_64, result_upr_f_60_64,
 result_f_65_69, sd_f_65_69, result_lwr_f_65_69, result_upr_f_65_69,
 result_f_70_74, sd_f_70_74, result_lwr_f_70_74, result_upr_f_70_74,
 result_f_75_79, sd_f_75_79, result_lwr_f_75_79, result_upr_f_75_79,
 result_f_80_84, sd_f_80_84, result_lwr_f_80_84, result_upr_f_80_84,
 result_f_85_89, sd_f_85_89, result_lwr_f_85_89, result_upr_f_85_89,
 result_f_90_94, sd_f_90_94, result_lwr_f_90_94, result_upr_f_90_94,
 result_f_95_, sd_f_95_, result_lwr_f_95_, result_upr_f_95_,
 result_m_15_19, sd_m_15_19, result_lwr_m_15_19, result_upr_m_15_19,
 result_m_20_24, sd_m_20_24, result_lwr_m_20_24, result_upr_m_20_24,
 result_m_25_29, sd_m_25_29, result_lwr_m_25_29, result_upr_m_25_29,
 result_m_30_34, sd_m_30_34, result_lwr_m_30_34, result_upr_m_30_34,
 result_m_35_39, sd_m_35_39, result_lwr_m_35_39, result_upr_m_35_39,
 result_m_40_44, sd_m_40_44, result_lwr_m_40_44, result_upr_m_40_44,
 result_m_45_49, sd_m_45_49, result_lwr_m_45_49, result_upr_m_45_49,
 result_m_50_54, sd_m_50_54, result_lwr_m_50_54, result_upr_m_50_54,
 result_m_55_59, sd_m_55_59, result_lwr_m_55_59, result_upr_m_55_59,
 result_m_60_64, sd_m_60_64, result_lwr_m_60_64, result_upr_m_60_64,
 result_m_65_69, sd_m_65_69, result_lwr_m_65_69, result_upr_m_65_69,
 result_m_70_74, sd_m_70_74, result_lwr_m_70_74, result_upr_m_70_74,
 result_m_75_79, sd_m_75_79, result_lwr_m_75_79, result_upr_m_75_79,
 result_m_80_84, sd_m_80_84, result_lwr_m_80_84, result_upr_m_80_84,
 result_m_85_89, sd_m_85_89, result_lwr_m_85_89, result_upr_m_85_89,
 result_m_90_94, sd_m_90_94, result_lwr_m_90_94, result_upr_m_90_94,
 result_m_95_, sd_m_95_, result_lwr_m_95_, result_upr_m_95_)
 }
 if (outcome %in% c("DTHratio")){
 envelope_stroke_sexage_global <- df_in %>%
 filter(outcome == "DTHparent",
 year == !!year,
 cause == !!cause) %>%
 arrange(region, country_abbrev, year, sex, ilo_age_bands_5yr) %>%
 drop_na() %>%
 mutate(sd = (result_upr - result_lwr)/2/1.96) %>%
 group_by(sex, ilo_age_bands_5yr) %>%
 summarise(result = sum(result),
 sd = sqrt(sum((sd)^2))) %>%
 ungroup %>%
 pivot_wider(names_from = sex:ilo_age_bands_5yr, values_from = result:sd)

 dths_stroke_sexage_global <- df_in %>%
 filter(outcome == "DTHenvelope",
 year == !!year,
 cause == !!cause) %>%
 arrange(region, country_abbrev, year, sex, ilo_age_bands_5yr) %>%
 drop_na() %>%
 mutate(sd = (result_upr - result_lwr)/2/1.96) %>%
 group_by(sex, ilo_age_bands_5yr) %>%
 summarise(result = sum(result),
 sd = sqrt(sum((sd)^2))) %>%
 ungroup %>%
 pivot_wider(names_from = sex:ilo_age_bands_5yr, values_from = result:sd) %>%

 select_all(.funs = funs(str_c(., "1")))

 dths_stroke_sexage_global <- bind_cols(dths_stroke_sexage_global, envelope_stroke_sexage_global) %>%
 rowwise() %>%
 transmute(result_lwr_f_15_19 = whoilo_paf_summary(result_f_15_19, result_f_15_191, sd_f_15_19, sd_f_15_191, CI = 0.025),
 result_upr_f_15_19 = whoilo_paf_summary(result_f_15_19, result_f_15_191, sd_f_15_19, sd_f_15_191, CI = 0.975),
 result_f_15_19 = whoilo_paf_summary(result_f_15_19, result_f_15_191, CI = NULL),
 result_lwr_f_20_24 = whoilo_paf_summary(result_f_20_24, result_f_20_241, sd_f_20_24, sd_f_20_241, CI = 0.025),
 result_upr_f_20_24 = whoilo_paf_summary(result_f_20_24, result_f_20_241, sd_f_20_24, sd_f_20_241, CI = 0.975),
 result_f_20_24 = whoilo_paf_summary(result_f_20_24, result_f_20_241, CI = NULL),
 result_lwr_f_25_29 = whoilo_paf_summary(result_f_25_29, result_f_25_291, sd_f_25_29, sd_f_25_291, CI = 0.025),
 result_upr_f_25_29 = whoilo_paf_summary(result_f_25_29, result_f_25_291, sd_f_25_29, sd_f_25_291, CI = 0.975),
 result_f_25_29 = whoilo_paf_summary(result_f_25_29, result_f_25_291, CI = NULL),
 result_lwr_f_30_34 = whoilo_paf_summary(result_f_30_34, result_f_30_341, sd_f_30_34, sd_f_30_341, CI = 0.025),
 result_upr_f_30_34 = whoilo_paf_summary(result_f_30_34, result_f_30_341, sd_f_30_34, sd_f_30_341, CI = 0.975),
 result_f_30_34 = whoilo_paf_summary(result_f_30_34, result_f_30_341, CI = NULL),
 result_lwr_f_35_39 = whoilo_paf_summary(result_f_35_39, result_f_35_391, sd_f_35_39, sd_f_35_391, CI = 0.025),
 result_upr_f_35_39 = whoilo_paf_summary(result_f_35_39, result_f_35_391, sd_f_35_39, sd_f_35_391, CI = 0.975),
 result_f_35_39 = whoilo_paf_summary(result_f_35_39, result_f_35_391, CI = NULL),
 result_lwr_f_40_44 = whoilo_paf_summary(result_f_40_44, result_f_40_441, sd_f_40_44, sd_f_40_441, CI = 0.025),
 result_upr_f_40_44 = whoilo_paf_summary(result_f_40_44, result_f_40_441, sd_f_40_44, sd_f_40_441, CI = 0.975),
 result_f_40_44 = whoilo_paf_summary(result_f_40_44, result_f_40_441, CI = NULL),
 result_lwr_f_45_49 = whoilo_paf_summary(result_f_45_49, result_f_45_491, sd_f_45_49, sd_f_45_491, CI = 0.025),
 result_upr_f_45_49 = whoilo_paf_summary(result_f_45_49, result_f_45_491, sd_f_45_49, sd_f_45_491, CI = 0.975),
 result_f_45_49 = whoilo_paf_summary(result_f_45_49, result_f_45_491, CI = NULL),
 result_lwr_f_50_54 = whoilo_paf_summary(result_f_50_54, result_f_50_541, sd_f_50_54, sd_f_50_541, CI = 0.025),
 result_upr_f_50_54 = whoilo_paf_summary(result_f_50_54, result_f_50_541, sd_f_50_54, sd_f_50_541, CI = 0.975),
 result_f_50_54 = whoilo_paf_summary(result_f_50_54, result_f_50_541, CI = NULL),
 result_lwr_f_55_59 = whoilo_paf_summary(result_f_55_59, result_f_55_591, sd_f_55_59, sd_f_55_591, CI = 0.025),
 result_upr_f_55_59 = whoilo_paf_summary(result_f_55_59, result_f_55_591, sd_f_55_59, sd_f_55_591, CI = 0.975),
 result_f_55_59 = whoilo_paf_summary(result_f_55_59, result_f_55_591, CI = NULL),
 result_lwr_f_60_64 = whoilo_paf_summary(result_f_60_64, result_f_60_641, sd_f_60_64, sd_f_60_641, CI = 0.025),
 result_upr_f_60_64 = whoilo_paf_summary(result_f_60_64, result_f_60_641, sd_f_60_64, sd_f_60_641, CI = 0.975),
 result_f_60_64 = whoilo_paf_summary(result_f_60_64, result_f_60_641, CI = NULL),
 result_lwr_f_65_69 = whoilo_paf_summary(result_f_65_69, result_f_65_691, sd_f_65_69, sd_f_65_691, CI = 0.025),
 result_upr_f_65_69 = whoilo_paf_summary(result_f_65_69, result_f_65_691, sd_f_65_69, sd_f_65_691, CI = 0.975),
 result_f_65_69 = whoilo_paf_summary(result_f_65_69, result_f_65_691, CI = NULL),
 result_lwr_f_70_74 = whoilo_paf_summary(result_f_70_74, result_f_70_741, sd_f_70_74, sd_f_70_741, CI = 0.025),
 result_upr_f_70_74 = whoilo_paf_summary(result_f_70_74, result_f_70_741, sd_f_70_74, sd_f_70_741, CI = 0.975),
 result_f_70_74 = whoilo_paf_summary(result_f_70_74, result_f_70_741, CI = NULL),
 result_lwr_f_75_79 = whoilo_paf_summary(result_f_75_79, result_f_75_791, sd_f_75_79, sd_f_75_791, CI = 0.025),
 result_upr_f_75_79 = whoilo_paf_summary(result_f_75_79, result_f_75_791, sd_f_75_79, sd_f_75_791, CI = 0.975),
 result_f_75_79 = whoilo_paf_summary(result_f_75_79, result_f_75_791, CI = NULL),
 result_lwr_f_80_84 = whoilo_paf_summary(result_f_80_84, result_f_80_841, sd_f_80_84, sd_f_80_841, CI = 0.025),
 result_upr_f_80_84 = whoilo_paf_summary(result_f_80_84, result_f_80_841, sd_f_80_84, sd_f_80_841, CI = 0.975),
 result_f_80_84 = whoilo_paf_summary(result_f_80_84, result_f_80_841, CI = NULL),
 result_lwr_f_85_89 = whoilo_paf_summary(result_f_85_89, result_f_85_891, sd_f_85_89, sd_f_85_891, CI = 0.025),
 result_upr_f_85_89 = whoilo_paf_summary(result_f_85_89, result_f_85_891, sd_f_85_89, sd_f_85_891, CI = 0.975),
 result_f_85_89 = whoilo_paf_summary(result_f_85_89, result_f_85_891, CI = NULL),
 result_lwr_f_90_94 = whoilo_paf_summary(result_f_90_94, result_f_90_941, sd_f_90_94, sd_f_90_941, CI = 0.025),
 result_upr_f_90_94 = whoilo_paf_summary(result_f_90_94, result_f_90_941, sd_f_90_94, sd_f_90_941, CI = 0.975),
 result_f_90_94 = whoilo_paf_summary(result_f_90_94, result_f_90_941, CI = NULL),
 result_lwr_f_95_ = whoilo_paf_summary(result_f_95_, result_f_95_1, sd_f_95_, sd_f_95_1, CI = 0.025),
 result_upr_f_95_ = whoilo_paf_summary(result_f_95_, result_f_95_1, sd_f_95_, sd_f_95_1, CI = 0.975),
 result_f_95_ = whoilo_paf_summary(result_f_95_, result_f_95_1, CI = NULL),
 result_lwr_m_15_19 = whoilo_paf_summary(result_m_15_19, result_m_15_191, sd_m_15_19, sd_m_15_191, CI = 0.025),
 result_upr_m_15_19 = whoilo_paf_summary(result_m_15_19, result_m_15_191, sd_m_15_19, sd_m_15_191, CI = 0.975),
 result_m_15_19 = whoilo_paf_summary(result_m_15_19, result_m_15_191, CI = NULL),
 result_lwr_m_20_24 = whoilo_paf_summary(result_m_20_24, result_m_20_241, sd_m_20_24, sd_m_20_241, CI = 0.025),
 result_upr_m_20_24 = whoilo_paf_summary(result_m_20_24, result_m_20_241, sd_m_20_24, sd_m_20_241, CI = 0.975),
 result_m_20_24 = whoilo_paf_summary(result_m_20_24, result_m_20_241, CI = NULL),
 result_lwr_m_25_29 = whoilo_paf_summary(result_m_25_29, result_m_25_291, sd_m_25_29, sd_m_25_291, CI = 0.025),
 result_upr_m_25_29 = whoilo_paf_summary(result_m_25_29, result_m_25_291, sd_m_25_29, sd_m_25_291, CI = 0.975),
 result_m_25_29 = whoilo_paf_summary(result_m_25_29, result_m_25_291, CI = NULL),
 result_lwr_m_30_34 = whoilo_paf_summary(result_m_30_34, result_m_30_341, sd_m_30_34, sd_m_30_341, CI = 0.025),
 result_upr_m_30_34 = whoilo_paf_summary(result_m_30_34, result_m_30_341, sd_m_30_34, sd_m_30_341, CI = 0.975),
 result_m_30_34 = whoilo_paf_summary(result_m_30_34, result_m_30_341, CI = NULL),
 result_lwr_m_35_39 = whoilo_paf_summary(result_m_35_39, result_m_35_391, sd_m_35_39, sd_m_35_391, CI = 0.025),
 result_upr_m_35_39 = whoilo_paf_summary(result_m_35_39, result_m_35_391, sd_m_35_39, sd_m_35_391, CI = 0.975),
 result_m_35_39 = whoilo_paf_summary(result_m_35_39, result_m_35_391, CI = NULL),
 result_lwr_m_40_44 = whoilo_paf_summary(result_m_40_44, result_m_40_441, sd_m_40_44, sd_m_40_441, CI = 0.025),
 result_upr_m_40_44 = whoilo_paf_summary(result_m_40_44, result_m_40_441, sd_m_40_44, sd_m_40_441, CI = 0.975),
 result_m_40_44 = whoilo_paf_summary(result_m_40_44, result_m_40_441, CI = NULL),
 result_lwr_m_45_49 = whoilo_paf_summary(result_m_45_49, result_m_45_491, sd_m_45_49, sd_m_45_491, CI = 0.025),
 result_upr_m_45_49 = whoilo_paf_summary(result_m_45_49, result_m_45_491, sd_m_45_49, sd_m_45_491, CI = 0.975),
 result_m_45_49 = whoilo_paf_summary(result_m_45_49, result_m_45_491, CI = NULL),
 result_lwr_m_50_54 = whoilo_paf_summary(result_m_50_54, result_m_50_541, sd_m_50_54, sd_m_50_541, CI = 0.025),
 result_upr_m_50_54 = whoilo_paf_summary(result_m_50_54, result_m_50_541, sd_m_50_54, sd_m_50_541, CI = 0.975),
 result_m_50_54 = whoilo_paf_summary(result_m_50_54, result_m_50_541, CI = NULL),
 result_lwr_m_55_59 = whoilo_paf_summary(result_m_55_59, result_m_55_591, sd_m_55_59, sd_m_55_591, CI = 0.025),
 result_upr_m_55_59 = whoilo_paf_summary(result_m_55_59, result_m_55_591, sd_m_55_59, sd_m_55_591, CI = 0.975),
 result_m_55_59 = whoilo_paf_summary(result_m_55_59, result_m_55_591, CI = NULL),
 result_lwr_m_60_64 = whoilo_paf_summary(result_m_60_64, result_m_60_641, sd_m_60_64, sd_m_60_641, CI = 0.025),
 result_upr_m_60_64 = whoilo_paf_summary(result_m_60_64, result_m_60_641, sd_m_60_64, sd_m_60_641, CI = 0.975),
 result_m_60_64 = whoilo_paf_summary(result_m_60_64, result_m_60_641, CI = NULL),
 result_lwr_m_65_69 = whoilo_paf_summary(result_m_65_69, result_m_65_691, sd_m_65_69, sd_m_65_691, CI = 0.025),
 result_upr_m_65_69 = whoilo_paf_summary(result_m_65_69, result_m_65_691, sd_m_65_69, sd_m_65_691, CI = 0.975),
 result_m_65_69 = whoilo_paf_summary(result_m_65_69, result_m_65_691, CI = NULL),
 result_lwr_m_70_74 = whoilo_paf_summary(result_m_70_74, result_m_70_741, sd_m_70_74, sd_m_70_741, CI = 0.025),
 result_upr_m_70_74 = whoilo_paf_summary(result_m_70_74, result_m_70_741, sd_m_70_74, sd_m_70_741, CI = 0.975),
 result_m_70_74 = whoilo_paf_summary(result_m_70_74, result_m_70_741, CI = NULL),
 result_lwr_m_75_79 = whoilo_paf_summary(result_m_75_79, result_m_75_791, sd_m_75_79, sd_m_75_791, CI = 0.025),
 result_upr_m_75_79 = whoilo_paf_summary(result_m_75_79, result_m_75_791, sd_m_75_79, sd_m_75_791, CI = 0.975),
 result_m_75_79 = whoilo_paf_summary(result_m_75_79, result_m_75_791, CI = NULL),
 result_lwr_m_80_84 = whoilo_paf_summary(result_m_80_84, result_m_80_841, sd_m_80_84, sd_m_80_841, CI = 0.025),
 result_upr_m_80_84 = whoilo_paf_summary(result_m_80_84, result_m_80_841, sd_m_80_84, sd_m_80_841, CI = 0.975),
 result_m_80_84 = whoilo_paf_summary(result_m_80_84, result_m_80_841, CI = NULL),
 result_lwr_m_85_89 = whoilo_paf_summary(result_m_85_89, result_m_85_891, sd_m_85_89, sd_m_85_891, CI = 0.025),
 result_upr_m_85_89 = whoilo_paf_summary(result_m_85_89, result_m_85_891, sd_m_85_89, sd_m_85_891, CI = 0.975),
 result_m_85_89 = whoilo_paf_summary(result_m_85_89, result_m_85_891, CI = NULL),
 result_lwr_m_90_94 = whoilo_paf_summary(result_m_90_94, result_m_90_941, sd_m_90_94, sd_m_90_941, CI = 0.025),
 result_upr_m_90_94 = whoilo_paf_summary(result_m_90_94, result_m_90_941, sd_m_90_94, sd_m_90_941, CI = 0.975),
 result_m_90_94 = whoilo_paf_summary(result_m_90_94, result_m_90_941, CI = NULL),
 result_lwr_m_95_ = whoilo_paf_summary(result_m_95_, result_m_95_1, sd_m_95_, sd_m_95_1, CI = 0.025),
 result_upr_m_95_ = whoilo_paf_summary(result_m_95_, result_m_95_1, sd_m_95_, sd_m_95_1, CI = 0.975),
 result_m_95_ = whoilo_paf_summary(result_m_95_, result_m_95_1, CI = NULL)) %>%
 ungroup() %>%
 mutate(sd_f_15_19 = (result_upr_f_15_19 - result_lwr_f_15_19)/2/1.96,
 sd_f_20_24 = (result_upr_f_20_24 - result_lwr_f_20_24)/2/1.96,
 sd_f_25_29 = (result_upr_f_25_29 - result_lwr_f_25_29)/2/1.96,
 sd_f_30_34 = (result_upr_f_30_34 - result_lwr_f_30_34)/2/1.96,
 sd_f_35_39 = (result_upr_f_35_39 - result_lwr_f_35_39)/2/1.96,
 sd_f_40_44 = (result_upr_f_40_44 - result_lwr_f_40_44)/2/1.96,
 sd_f_45_49 = (result_upr_f_45_49 - result_lwr_f_45_49)/2/1.96,
 sd_f_50_54 = (result_upr_f_50_54 - result_lwr_f_50_54)/2/1.96,
 sd_f_55_59 = (result_upr_f_55_59 - result_lwr_f_55_59)/2/1.96,
 sd_f_60_64 = (result_upr_f_60_64 - result_lwr_f_60_64)/2/1.96,
 sd_f_65_69 = (result_upr_f_65_69 - result_lwr_f_65_69)/2/1.96,
 sd_f_70_74 = (result_upr_f_70_74 - result_lwr_f_70_74)/2/1.96,
 sd_f_75_79 = (result_upr_f_75_79 - result_lwr_f_75_79)/2/1.96,
 sd_f_80_84 = (result_upr_f_80_84 - result_lwr_f_80_84)/2/1.96,
 sd_f_85_89 = (result_upr_f_85_89 - result_lwr_f_85_89)/2/1.96,
 sd_f_90_94 = (result_upr_f_90_94 - result_lwr_f_90_94)/2/1.96,
 sd_f_95_ = (result_upr_f_95_ - result_lwr_f_95_)/2/1.96,
 sd_m_15_19 = (result_upr_m_15_19 - result_lwr_m_15_19)/2/1.96,
 sd_m_20_24 = (result_upr_m_20_24 - result_lwr_m_20_24)/2/1.96,
 sd_m_25_29 = (result_upr_m_25_29 - result_lwr_m_25_29)/2/1.96,
 sd_m_30_34 = (result_upr_m_30_34 - result_lwr_m_30_34)/2/1.96,
 sd_m_35_39 = (result_upr_m_35_39 - result_lwr_m_35_39)/2/1.96,
 sd_m_40_44 = (result_upr_m_40_44 - result_lwr_m_40_44)/2/1.96,
 sd_m_45_49 = (result_upr_m_45_49 - result_lwr_m_45_49)/2/1.96,
 sd_m_50_54 = (result_upr_m_50_54 - result_lwr_m_50_54)/2/1.96,
 sd_m_55_59 = (result_upr_m_55_59 - result_lwr_m_55_59)/2/1.96,
 sd_m_60_64 = (result_upr_m_60_64 - result_lwr_m_60_64)/2/1.96,
 sd_m_65_69 = (result_upr_m_65_69 - result_lwr_m_65_69)/2/1.96,
 sd_m_70_74 = (result_upr_m_70_74 - result_lwr_m_70_74)/2/1.96,
 sd_m_75_79 = (result_upr_m_75_79 - result_lwr_m_75_79)/2/1.96,
 sd_m_80_84 = (result_upr_m_80_84 - result_lwr_m_80_84)/2/1.96,
 sd_m_85_89 = (result_upr_m_85_89 - result_lwr_m_85_89)/2/1.96,
 sd_m_90_94 = (result_upr_m_90_94 - result_lwr_m_90_94)/2/1.96,
 sd_m_95_ = (result_upr_m_95_ - result_lwr_m_95_)/2/1.96) %>%
 select(result_f_15_19, sd_f_15_19, result_lwr_f_15_19, result_upr_f_15_19,
 result_f_20_24, sd_f_20_24, result_lwr_f_20_24, result_upr_f_20_24,
 result_f_25_29, sd_f_25_29, result_lwr_f_25_29, result_upr_f_25_29,
 result_f_30_34, sd_f_30_34, result_lwr_f_30_34, result_upr_f_30_34,
 result_f_35_39, sd_f_35_39, result_lwr_f_35_39, result_upr_f_35_39,
 result_f_40_44, sd_f_40_44, result_lwr_f_40_44, result_upr_f_40_44,
 result_f_45_49, sd_f_45_49, result_lwr_f_45_49, result_upr_f_45_49,
 result_f_50_54, sd_f_50_54, result_lwr_f_50_54, result_upr_f_50_54,
 result_f_55_59, sd_f_55_59, result_lwr_f_55_59, result_upr_f_55_59,
 result_f_60_64, sd_f_60_64, result_lwr_f_60_64, result_upr_f_60_64,
 result_f_65_69, sd_f_65_69, result_lwr_f_65_69, result_upr_f_65_69,
 result_f_70_74, sd_f_70_74, result_lwr_f_70_74, result_upr_f_70_74,
 result_f_75_79, sd_f_75_79, result_lwr_f_75_79, result_upr_f_75_79,
 result_f_80_84, sd_f_80_84, result_lwr_f_80_84, result_upr_f_80_84,
 result_f_85_89, sd_f_85_89, result_lwr_f_85_89, result_upr_f_85_89,
 result_f_90_94, sd_f_90_94, result_lwr_f_90_94, result_upr_f_90_94,
 result_f_95_, sd_f_95_, result_lwr_f_95_, result_upr_f_95_,
 result_m_15_19, sd_m_15_19, result_lwr_m_15_19, result_upr_m_15_19,
 result_m_20_24, sd_m_20_24, result_lwr_m_20_24, result_upr_m_20_24,
 result_m_25_29, sd_m_25_29, result_lwr_m_25_29, result_upr_m_25_29,
 result_m_30_34, sd_m_30_34, result_lwr_m_30_34, result_upr_m_30_34,
 result_m_35_39, sd_m_35_39, result_lwr_m_35_39, result_upr_m_35_39,
 result_m_40_44, sd_m_40_44, result_lwr_m_40_44, result_upr_m_40_44,
 result_m_45_49, sd_m_45_49, result_lwr_m_45_49, result_upr_m_45_49,
 result_m_50_54, sd_m_50_54, result_lwr_m_50_54, result_upr_m_50_54,
 result_m_55_59, sd_m_55_59, result_lwr_m_55_59, result_upr_m_55_59,
 result_m_60_64, sd_m_60_64, result_lwr_m_60_64, result_upr_m_60_64,
 result_m_65_69, sd_m_65_69, result_lwr_m_65_69, result_upr_m_65_69,
 result_m_70_74, sd_m_70_74, result_lwr_m_70_74, result_upr_m_70_74,
 result_m_75_79, sd_m_75_79, result_lwr_m_75_79, result_upr_m_75_79,
 result_m_80_84, sd_m_80_84, result_lwr_m_80_84, result_upr_m_80_84,
 result_m_85_89, sd_m_85_89, result_lwr_m_85_89, result_upr_m_85_89,
 result_m_90_94, sd_m_90_94, result_lwr_m_90_94, result_upr_m_90_94,
 result_m_95_, sd_m_95_, result_lwr_m_95_, result_upr_m_95_)
 if (shape == "long") {
 dths_stroke_sexage_global <- dths_stroke_sexage_global

 }
 }
 if (outcome %in% c("DALYratio")){
 envelope_stroke_sexage_global <- df_in %>%
 filter(outcome == "DALYparent",
 year == !!year,
 cause == !!cause) %>%
 arrange(region, country_abbrev, year, sex, ilo_age_bands_5yr) %>%
 drop_na() %>%
 mutate(sd = (result_upr - result_lwr)/2/1.96) %>%
 group_by(sex, ilo_age_bands_5yr) %>%
 summarise(result = sum(result),
 sd = sqrt(sum((sd)^2))) %>%
 ungroup %>%
 pivot_wider(names_from = sex:ilo_age_bands_5yr, values_from = result:sd)

 dths_stroke_sexage_global <- df_in %>%
 filter(outcome == "DALYenvelope",
 year == !!year,
 cause == !!cause) %>%
 mutate(result_lwr = case_when(is.na(result_lwr) ~ result,
 TRUE ~ result_lwr),
 result_upr = case_when(is.na(result_upr) ~ result,
 TRUE ~ result_upr)) %>%
 arrange(region, country_abbrev, year, sex, ilo_age_bands_5yr) %>%
 drop_na() %>%
 mutate(sd = (result_upr - result_lwr)/2/1.96) %>%
 group_by(sex, ilo_age_bands_5yr) %>%
 summarise(result = sum(result),
 sd = sqrt(sum((sd)^2))) %>%
 ungroup %>%
 pivot_wider(names_from = sex:ilo_age_bands_5yr, values_from = result:sd) %>%

 select_all(.funs = funs(str_c(., "1")))

 dths_stroke_sexage_global <- bind_cols(dths_stroke_sexage_global, envelope_stroke_sexage_global) %>%
 rowwise() %>%
 transmute(result_lwr_f_15_19 = whoilo_paf_summary(result_f_15_19, result_f_15_191, sd_f_15_19, sd_f_15_191, CI = 0.025),
 result_upr_f_15_19 = whoilo_paf_summary(result_f_15_19, result_f_15_191, sd_f_15_19, sd_f_15_191, CI = 0.975),
 result_f_15_19 = whoilo_paf_summary(result_f_15_19, result_f_15_191, CI = NULL),
 result_lwr_f_20_24 = whoilo_paf_summary(result_f_20_24, result_f_20_241, sd_f_20_24, sd_f_20_241, CI = 0.025),
 result_upr_f_20_24 = whoilo_paf_summary(result_f_20_24, result_f_20_241, sd_f_20_24, sd_f_20_241, CI = 0.975),
 result_f_20_24 = whoilo_paf_summary(result_f_20_24, result_f_20_241, CI = NULL),
 result_lwr_f_25_29 = whoilo_paf_summary(result_f_25_29, result_f_25_291, sd_f_25_29, sd_f_25_291, CI = 0.025),
 result_upr_f_25_29 = whoilo_paf_summary(result_f_25_29, result_f_25_291, sd_f_25_29, sd_f_25_291, CI = 0.975),
 result_f_25_29 = whoilo_paf_summary(result_f_25_29, result_f_25_291, CI = NULL),
 result_lwr_f_30_34 = whoilo_paf_summary(result_f_30_34, result_f_30_341, sd_f_30_34, sd_f_30_341, CI = 0.025),
 result_upr_f_30_34 = whoilo_paf_summary(result_f_30_34, result_f_30_341, sd_f_30_34, sd_f_30_341, CI = 0.975),
 result_f_30_34 = whoilo_paf_summary(result_f_30_34, result_f_30_341, CI = NULL),
 result_lwr_f_35_39 = whoilo_paf_summary(result_f_35_39, result_f_35_391, sd_f_35_39, sd_f_35_391, CI = 0.025),
 result_upr_f_35_39 = whoilo_paf_summary(result_f_35_39, result_f_35_391, sd_f_35_39, sd_f_35_391, CI = 0.975),
 result_f_35_39 = whoilo_paf_summary(result_f_35_39, result_f_35_391, CI = NULL),
 result_lwr_f_40_44 = whoilo_paf_summary(result_f_40_44, result_f_40_441, sd_f_40_44, sd_f_40_441, CI = 0.025),
 result_upr_f_40_44 = whoilo_paf_summary(result_f_40_44, result_f_40_441, sd_f_40_44, sd_f_40_441, CI = 0.975),
 result_f_40_44 = whoilo_paf_summary(result_f_40_44, result_f_40_441, CI = NULL),
 result_lwr_f_45_49 = whoilo_paf_summary(result_f_45_49, result_f_45_491, sd_f_45_49, sd_f_45_491, CI = 0.025),
 result_upr_f_45_49 = whoilo_paf_summary(result_f_45_49, result_f_45_491, sd_f_45_49, sd_f_45_491, CI = 0.975),
 result_f_45_49 = whoilo_paf_summary(result_f_45_49, result_f_45_491, CI = NULL),
 result_lwr_f_50_54 = whoilo_paf_summary(result_f_50_54, result_f_50_541, sd_f_50_54, sd_f_50_541, CI = 0.025),
 result_upr_f_50_54 = whoilo_paf_summary(result_f_50_54, result_f_50_541, sd_f_50_54, sd_f_50_541, CI = 0.975),
 result_f_50_54 = whoilo_paf_summary(result_f_50_54, result_f_50_541, CI = NULL),
 result_lwr_f_55_59 = whoilo_paf_summary(result_f_55_59, result_f_55_591, sd_f_55_59, sd_f_55_591, CI = 0.025),
 result_upr_f_55_59 = whoilo_paf_summary(result_f_55_59, result_f_55_591, sd_f_55_59, sd_f_55_591, CI = 0.975),
 result_f_55_59 = whoilo_paf_summary(result_f_55_59, result_f_55_591, CI = NULL),
 result_lwr_f_60_64 = whoilo_paf_summary(result_f_60_64, result_f_60_641, sd_f_60_64, sd_f_60_641, CI = 0.025),
 result_upr_f_60_64 = whoilo_paf_summary(result_f_60_64, result_f_60_641, sd_f_60_64, sd_f_60_641, CI = 0.975),
 result_f_60_64 = whoilo_paf_summary(result_f_60_64, result_f_60_641, CI = NULL),
 result_lwr_f_65_69 = whoilo_paf_summary(result_f_65_69, result_f_65_691, sd_f_65_69, sd_f_65_691, CI = 0.025),
 result_upr_f_65_69 = whoilo_paf_summary(result_f_65_69, result_f_65_691, sd_f_65_69, sd_f_65_691, CI = 0.975),
 result_f_65_69 = whoilo_paf_summary(result_f_65_69, result_f_65_691, CI = NULL),
 result_lwr_f_70_74 = whoilo_paf_summary(result_f_70_74, result_f_70_741, sd_f_70_74, sd_f_70_741, CI = 0.025),
 result_upr_f_70_74 = whoilo_paf_summary(result_f_70_74, result_f_70_741, sd_f_70_74, sd_f_70_741, CI = 0.975),
 result_f_70_74 = whoilo_paf_summary(result_f_70_74, result_f_70_741, CI = NULL),
 result_lwr_f_75_79 = whoilo_paf_summary(result_f_75_79, result_f_75_791, sd_f_75_79, sd_f_75_791, CI = 0.025),
 result_upr_f_75_79 = whoilo_paf_summary(result_f_75_79, result_f_75_791, sd_f_75_79, sd_f_75_791, CI = 0.975),
 result_f_75_79 = whoilo_paf_summary(result_f_75_79, result_f_75_791, CI = NULL),
 result_lwr_f_80_84 = whoilo_paf_summary(result_f_80_84, result_f_80_841, sd_f_80_84, sd_f_80_841, CI = 0.025),
 result_upr_f_80_84 = whoilo_paf_summary(result_f_80_84, result_f_80_841, sd_f_80_84, sd_f_80_841, CI = 0.975),
 result_f_80_84 = whoilo_paf_summary(result_f_80_84, result_f_80_841, CI = NULL),
 result_lwr_f_85_89 = whoilo_paf_summary(result_f_85_89, result_f_85_891, sd_f_85_89, sd_f_85_891, CI = 0.025),
 result_upr_f_85_89 = whoilo_paf_summary(result_f_85_89, result_f_85_891, sd_f_85_89, sd_f_85_891, CI = 0.975),
 result_f_85_89 = whoilo_paf_summary(result_f_85_89, result_f_85_891, CI = NULL),
 result_lwr_f_90_94 = whoilo_paf_summary(result_f_90_94, result_f_90_941, sd_f_90_94, sd_f_90_941, CI = 0.025),
 result_upr_f_90_94 = whoilo_paf_summary(result_f_90_94, result_f_90_941, sd_f_90_94, sd_f_90_941, CI = 0.975),
 result_f_90_94 = whoilo_paf_summary(result_f_90_94, result_f_90_941, CI = NULL),
 result_lwr_f_95_ = whoilo_paf_summary(result_f_95_, result_f_95_1, sd_f_95_, sd_f_95_1, CI = 0.025),
 result_upr_f_95_ = whoilo_paf_summary(result_f_95_, result_f_95_1, sd_f_95_, sd_f_95_1, CI = 0.975),
 result_f_95_ = whoilo_paf_summary(result_f_95_, result_f_95_1, CI = NULL),
 result_lwr_m_15_19 = whoilo_paf_summary(result_m_15_19, result_m_15_191, sd_m_15_19, sd_m_15_191, CI = 0.025),
 result_upr_m_15_19 = whoilo_paf_summary(result_m_15_19, result_m_15_191, sd_m_15_19, sd_m_15_191, CI = 0.975),
 result_m_15_19 = whoilo_paf_summary(result_m_15_19, result_m_15_191, CI = NULL),
 result_lwr_m_20_24 = whoilo_paf_summary(result_m_20_24, result_m_20_241, sd_m_20_24, sd_m_20_241, CI = 0.025),
 result_upr_m_20_24 = whoilo_paf_summary(result_m_20_24, result_m_20_241, sd_m_20_24, sd_m_20_241, CI = 0.975),
 result_m_20_24 = whoilo_paf_summary(result_m_20_24, result_m_20_241, CI = NULL),
 result_lwr_m_25_29 = whoilo_paf_summary(result_m_25_29, result_m_25_291, sd_m_25_29, sd_m_25_291, CI = 0.025),
 result_upr_m_25_29 = whoilo_paf_summary(result_m_25_29, result_m_25_291, sd_m_25_29, sd_m_25_291, CI = 0.975),
 result_m_25_29 = whoilo_paf_summary(result_m_25_29, result_m_25_291, CI = NULL),
 result_lwr_m_30_34 = whoilo_paf_summary(result_m_30_34, result_m_30_341, sd_m_30_34, sd_m_30_341, CI = 0.025),
 result_upr_m_30_34 = whoilo_paf_summary(result_m_30_34, result_m_30_341, sd_m_30_34, sd_m_30_341, CI = 0.975),
 result_m_30_34 = whoilo_paf_summary(result_m_30_34, result_m_30_341, CI = NULL),
 result_lwr_m_35_39 = whoilo_paf_summary(result_m_35_39, result_m_35_391, sd_m_35_39, sd_m_35_391, CI = 0.025),
 result_upr_m_35_39 = whoilo_paf_summary(result_m_35_39, result_m_35_391, sd_m_35_39, sd_m_35_391, CI = 0.975),
 result_m_35_39 = whoilo_paf_summary(result_m_35_39, result_m_35_391, CI = NULL),
 result_lwr_m_40_44 = whoilo_paf_summary(result_m_40_44, result_m_40_441, sd_m_40_44, sd_m_40_441, CI = 0.025),
 result_upr_m_40_44 = whoilo_paf_summary(result_m_40_44, result_m_40_441, sd_m_40_44, sd_m_40_441, CI = 0.975),
 result_m_40_44 = whoilo_paf_summary(result_m_40_44, result_m_40_441, CI = NULL),
 result_lwr_m_45_49 = whoilo_paf_summary(result_m_45_49, result_m_45_491, sd_m_45_49, sd_m_45_491, CI = 0.025),
 result_upr_m_45_49 = whoilo_paf_summary(result_m_45_49, result_m_45_491, sd_m_45_49, sd_m_45_491, CI = 0.975),
 result_m_45_49 = whoilo_paf_summary(result_m_45_49, result_m_45_491, CI = NULL),
 result_lwr_m_50_54 = whoilo_paf_summary(result_m_50_54, result_m_50_541, sd_m_50_54, sd_m_50_541, CI = 0.025),
 result_upr_m_50_54 = whoilo_paf_summary(result_m_50_54, result_m_50_541, sd_m_50_54, sd_m_50_541, CI = 0.975),
 result_m_50_54 = whoilo_paf_summary(result_m_50_54, result_m_50_541, CI = NULL),
 result_lwr_m_55_59 = whoilo_paf_summary(result_m_55_59, result_m_55_591, sd_m_55_59, sd_m_55_591, CI = 0.025),
 result_upr_m_55_59 = whoilo_paf_summary(result_m_55_59, result_m_55_591, sd_m_55_59, sd_m_55_591, CI = 0.975),
 result_m_55_59 = whoilo_paf_summary(result_m_55_59, result_m_55_591, CI = NULL),
 result_lwr_m_60_64 = whoilo_paf_summary(result_m_60_64, result_m_60_641, sd_m_60_64, sd_m_60_641, CI = 0.025),
 result_upr_m_60_64 = whoilo_paf_summary(result_m_60_64, result_m_60_641, sd_m_60_64, sd_m_60_641, CI = 0.975),
 result_m_60_64 = whoilo_paf_summary(result_m_60_64, result_m_60_641, CI = NULL),
 result_lwr_m_65_69 = whoilo_paf_summary(result_m_65_69, result_m_65_691, sd_m_65_69, sd_m_65_691, CI = 0.025),
 result_upr_m_65_69 = whoilo_paf_summary(result_m_65_69, result_m_65_691, sd_m_65_69, sd_m_65_691, CI = 0.975),
 result_m_65_69 = whoilo_paf_summary(result_m_65_69, result_m_65_691, CI = NULL),
 result_lwr_m_70_74 = whoilo_paf_summary(result_m_70_74, result_m_70_741, sd_m_70_74, sd_m_70_741, CI = 0.025),
 result_upr_m_70_74 = whoilo_paf_summary(result_m_70_74, result_m_70_741, sd_m_70_74, sd_m_70_741, CI = 0.975),
 result_m_70_74 = whoilo_paf_summary(result_m_70_74, result_m_70_741, CI = NULL),
 result_lwr_m_75_79 = whoilo_paf_summary(result_m_75_79, result_m_75_791, sd_m_75_79, sd_m_75_791, CI = 0.025),
 result_upr_m_75_79 = whoilo_paf_summary(result_m_75_79, result_m_75_791, sd_m_75_79, sd_m_75_791, CI = 0.975),
 result_m_75_79 = whoilo_paf_summary(result_m_75_79, result_m_75_791, CI = NULL),
 result_lwr_m_80_84 = whoilo_paf_summary(result_m_80_84, result_m_80_841, sd_m_80_84, sd_m_80_841, CI = 0.025),
 result_upr_m_80_84 = whoilo_paf_summary(result_m_80_84, result_m_80_841, sd_m_80_84, sd_m_80_841, CI = 0.975),
 result_m_80_84 = whoilo_paf_summary(result_m_80_84, result_m_80_841, CI = NULL),
 result_lwr_m_85_89 = whoilo_paf_summary(result_m_85_89, result_m_85_891, sd_m_85_89, sd_m_85_891, CI = 0.025),
 result_upr_m_85_89 = whoilo_paf_summary(result_m_85_89, result_m_85_891, sd_m_85_89, sd_m_85_891, CI = 0.975),
 result_m_85_89 = whoilo_paf_summary(result_m_85_89, result_m_85_891, CI = NULL),
 result_lwr_m_90_94 = whoilo_paf_summary(result_m_90_94, result_m_90_941, sd_m_90_94, sd_m_90_941, CI = 0.025),
 result_upr_m_90_94 = whoilo_paf_summary(result_m_90_94, result_m_90_941, sd_m_90_94, sd_m_90_941, CI = 0.975),
 result_m_90_94 = whoilo_paf_summary(result_m_90_94, result_m_90_941, CI = NULL),
 result_lwr_m_95_ = whoilo_paf_summary(result_m_95_, result_m_95_1, sd_m_95_, sd_m_95_1, CI = 0.025),
 result_upr_m_95_ = whoilo_paf_summary(result_m_95_, result_m_95_1, sd_m_95_, sd_m_95_1, CI = 0.975),
 result_m_95_ = whoilo_paf_summary(result_m_95_, result_m_95_1, CI = NULL)) %>%
 ungroup() %>%
 mutate(sd_f_15_19 = (result_upr_f_15_19 - result_lwr_f_15_19)/2/1.96,
 sd_f_20_24 = (result_upr_f_20_24 - result_lwr_f_20_24)/2/1.96,
 sd_f_25_29 = (result_upr_f_25_29 - result_lwr_f_25_29)/2/1.96,
 sd_f_30_34 = (result_upr_f_30_34 - result_lwr_f_30_34)/2/1.96,
 sd_f_35_39 = (result_upr_f_35_39 - result_lwr_f_35_39)/2/1.96,
 sd_f_40_44 = (result_upr_f_40_44 - result_lwr_f_40_44)/2/1.96,
 sd_f_45_49 = (result_upr_f_45_49 - result_lwr_f_45_49)/2/1.96,
 sd_f_50_54 = (result_upr_f_50_54 - result_lwr_f_50_54)/2/1.96,
 sd_f_55_59 = (result_upr_f_55_59 - result_lwr_f_55_59)/2/1.96,
 sd_f_60_64 = (result_upr_f_60_64 - result_lwr_f_60_64)/2/1.96,
 sd_f_65_69 = (result_upr_f_65_69 - result_lwr_f_65_69)/2/1.96,
 sd_f_70_74 = (result_upr_f_70_74 - result_lwr_f_70_74)/2/1.96,
 sd_f_75_79 = (result_upr_f_75_79 - result_lwr_f_75_79)/2/1.96,
 sd_f_80_84 = (result_upr_f_80_84 - result_lwr_f_80_84)/2/1.96,
 sd_f_85_89 = (result_upr_f_85_89 - result_lwr_f_85_89)/2/1.96,
 sd_f_90_94 = (result_upr_f_90_94 - result_lwr_f_90_94)/2/1.96,
 sd_f_95_ = (result_upr_f_95_ - result_lwr_f_95_)/2/1.96,
 sd_m_15_19 = (result_upr_m_15_19 - result_lwr_m_15_19)/2/1.96,
 sd_m_20_24 = (result_upr_m_20_24 - result_lwr_m_20_24)/2/1.96,
 sd_m_25_29 = (result_upr_m_25_29 - result_lwr_m_25_29)/2/1.96,
 sd_m_30_34 = (result_upr_m_30_34 - result_lwr_m_30_34)/2/1.96,
 sd_m_35_39 = (result_upr_m_35_39 - result_lwr_m_35_39)/2/1.96,
 sd_m_40_44 = (result_upr_m_40_44 - result_lwr_m_40_44)/2/1.96,
 sd_m_45_49 = (result_upr_m_45_49 - result_lwr_m_45_49)/2/1.96,
 sd_m_50_54 = (result_upr_m_50_54 - result_lwr_m_50_54)/2/1.96,
 sd_m_55_59 = (result_upr_m_55_59 - result_lwr_m_55_59)/2/1.96,
 sd_m_60_64 = (result_upr_m_60_64 - result_lwr_m_60_64)/2/1.96,
 sd_m_65_69 = (result_upr_m_65_69 - result_lwr_m_65_69)/2/1.96,
 sd_m_70_74 = (result_upr_m_70_74 - result_lwr_m_70_74)/2/1.96,
 sd_m_75_79 = (result_upr_m_75_79 - result_lwr_m_75_79)/2/1.96,
 sd_m_80_84 = (result_upr_m_80_84 - result_lwr_m_80_84)/2/1.96,
 sd_m_85_89 = (result_upr_m_85_89 - result_lwr_m_85_89)/2/1.96,
 sd_m_90_94 = (result_upr_m_90_94 - result_lwr_m_90_94)/2/1.96,
 sd_m_95_ = (result_upr_m_95_ - result_lwr_m_95_)/2/1.96) %>%
 select(result_f_15_19, sd_f_15_19, result_lwr_f_15_19, result_upr_f_15_19,
 result_f_20_24, sd_f_20_24, result_lwr_f_20_24, result_upr_f_20_24,
 result_f_25_29, sd_f_25_29, result_lwr_f_25_29, result_upr_f_25_29,
 result_f_30_34, sd_f_30_34, result_lwr_f_30_34, result_upr_f_30_34,
 result_f_35_39, sd_f_35_39, result_lwr_f_35_39, result_upr_f_35_39,
 result_f_40_44, sd_f_40_44, result_lwr_f_40_44, result_upr_f_40_44,
 result_f_45_49, sd_f_45_49, result_lwr_f_45_49, result_upr_f_45_49,
 result_f_50_54, sd_f_50_54, result_lwr_f_50_54, result_upr_f_50_54,
 result_f_55_59, sd_f_55_59, result_lwr_f_55_59, result_upr_f_55_59,
 result_f_60_64, sd_f_60_64, result_lwr_f_60_64, result_upr_f_60_64,
 result_f_65_69, sd_f_65_69, result_lwr_f_65_69, result_upr_f_65_69,
 result_f_70_74, sd_f_70_74, result_lwr_f_70_74, result_upr_f_70_74,
 result_f_75_79, sd_f_75_79, result_lwr_f_75_79, result_upr_f_75_79,
 result_f_80_84, sd_f_80_84, result_lwr_f_80_84, result_upr_f_80_84,
 result_f_85_89, sd_f_85_89, result_lwr_f_85_89, result_upr_f_85_89,
 result_f_90_94, sd_f_90_94, result_lwr_f_90_94, result_upr_f_90_94,
 result_f_95_, sd_f_95_, result_lwr_f_95_, result_upr_f_95_,
 result_m_15_19, sd_m_15_19, result_lwr_m_15_19, result_upr_m_15_19,
 result_m_20_24, sd_m_20_24, result_lwr_m_20_24, result_upr_m_20_24,
 result_m_25_29, sd_m_25_29, result_lwr_m_25_29, result_upr_m_25_29,
 result_m_30_34, sd_m_30_34, result_lwr_m_30_34, result_upr_m_30_34,
 result_m_35_39, sd_m_35_39, result_lwr_m_35_39, result_upr_m_35_39,
 result_m_40_44, sd_m_40_44, result_lwr_m_40_44, result_upr_m_40_44,
 result_m_45_49, sd_m_45_49, result_lwr_m_45_49, result_upr_m_45_49,
 result_m_50_54, sd_m_50_54, result_lwr_m_50_54, result_upr_m_50_54,
 result_m_55_59, sd_m_55_59, result_lwr_m_55_59, result_upr_m_55_59,
 result_m_60_64, sd_m_60_64, result_lwr_m_60_64, result_upr_m_60_64,
 result_m_65_69, sd_m_65_69, result_lwr_m_65_69, result_upr_m_65_69,
 result_m_70_74, sd_m_70_74, result_lwr_m_70_74, result_upr_m_70_74,
 result_m_75_79, sd_m_75_79, result_lwr_m_75_79, result_upr_m_75_79,
 result_m_80_84, sd_m_80_84, result_lwr_m_80_84, result_upr_m_80_84,
 result_m_85_89, sd_m_85_89, result_lwr_m_85_89, result_upr_m_85_89,
 result_m_90_94, sd_m_90_94, result_lwr_m_90_94, result_upr_m_90_94,
 result_m_95_, sd_m_95_, result_lwr_m_95_, result_upr_m_95_)
 if (shape == "long") {
 dths_stroke_sexage_global <- dths_stroke_sexage_global

 }
 }
 }
 # combine them all
 df_out <- as.data.frame(year)
 names(df_out) <- "year"
 if ("total" %in% breakdown){
 df_out <- df_out %>%
 bind_cols(dths_stroke_global)
 }
 if ("sex" %in% breakdown){
 df_out <- df_out %>%
 bind_cols(dths_stroke_sex_global)
 }
 if ("age" %in% breakdown){
 df_out <- df_out %>%
 bind_cols(dths_stroke_age_global)
 }
 if ("sexage" %in% breakdown){
 df_out <- df_out %>%
 bind_cols(dths_stroke_sexage_global)
 }
 # return the final output
 return(df_out)
}
my_func_regional <- function(df_in,
 year,
 outcome,
 cause,
 breakdown){
 # total
 if ("total" %in% breakdown){
 if (outcome %in% c("PAF", "DTHpaf")){
 envelope_stroke_region <- df_in %>%
 filter(outcome == "DTHenvelope",
 year == !!year,
 cause == !!cause) %>%
 arrange(region, country_abbrev, year, sex, ilo_age_bands_5yr) %>%
 drop_na() %>%
 group_by(region) %>%
 mutate(sd = (result_upr - result_lwr)/2/1.96) %>%
 summarise(result = sum(result),
 sd = sqrt(sum((sd)^2))) %>%
 mutate(result_lwr = qnorm(p = 0.025, mean = result, sd = sd),
 result_upr = qnorm(p = 0.975, mean = result, sd = sd)) %>%
 ungroup %>%

 select_all(.funs = funs(str_c(., "1")))

 dths_stroke_global <- df_in %>%
 filter(outcome == "DTH",
 year == !!year,
 cause == !!cause) %>%
 arrange(region, country_abbrev, year, sex, ilo_age_bands_5yr) %>%
 drop_na() %>%
 group_by(region) %>%
 mutate(sd = (result_upr - result_lwr)/2/1.96) %>%
 summarise(result = sum(result),
 sd = sqrt(sum((sd)^2))) %>%
 mutate(result_lwr = qnorm(p = 0.025, mean = result, sd = sd),
 result_upr = qnorm(p = 0.975, mean = result, sd = sd)) %>%
 ungroup
 dths_stroke_global <- bind_cols(dths_stroke_global, envelope_stroke_region) %>%
 rowwise() %>%
 transmute(region = region,
 result_b = whoilo_paf_summary(result, result1, CI = NULL),
 result_lwr_b = whoilo_paf_summary(result, result1, sd, sd1, CI = 0.025),
 result_upr_b = whoilo_paf_summary(result, result1, sd, sd1, CI = 0.975)) %>%
 mutate(sd_b = (result_upr_b - result_lwr_b)/2/1.96) %>%
 ungroup() %>%
 select(region, result_b, sd_b, result_lwr_b, result_upr_b)
 }
 if (outcome %in% c("DALYpaf")){
 envelope_stroke_region <- df_in %>%
 filter(outcome == "DALYenvelope",
 year == !!year,
 cause == !!cause) %>%
 mutate(result_lwr = case_when(is.na(result_lwr) ~ result,
 TRUE ~ result_lwr),
 result_upr = case_when(is.na(result_upr) ~ result,
 TRUE ~ result_upr)) %>%
 arrange(region, country_abbrev, year, sex, ilo_age_bands_5yr) %>%
 drop_na() %>%
 group_by(region) %>%
 mutate(sd = (result_upr - result_lwr)/2/1.96) %>%
 summarise(result = sum(result),
 sd = sqrt(sum((sd)^2))) %>%
 mutate(result_lwr = qnorm(p = 0.025, mean = result, sd = sd),
 result_upr = qnorm(p = 0.975, mean = result, sd = sd)) %>%
 ungroup %>%

 select_all(.funs = funs(str_c(., "1")))

 dths_stroke_global <- df_in %>%
 filter(outcome == "DALY",
 year == !!year,
 cause == !!cause) %>%
 arrange(region, country_abbrev, year, sex, ilo_age_bands_5yr) %>%
 drop_na() %>%
 group_by(region) %>%
 mutate(sd = (result_upr - result_lwr)/2/1.96) %>%
 summarise(result = sum(result),
 sd = sqrt(sum((sd)^2))) %>%
 mutate(result_lwr = qnorm(p = 0.025, mean = result, sd = sd),
 result_upr = qnorm(p = 0.975, mean = result, sd = sd)) %>%
 ungroup
 dths_stroke_global <- bind_cols(dths_stroke_global, envelope_stroke_region) %>%
 rowwise() %>%
 transmute(region = region,
 result_b = whoilo_paf_summary(result, result1, CI = NULL),
 result_lwr_b = whoilo_paf_summary(result, result1, sd, sd1, CI = 0.025),
 result_upr_b = whoilo_paf_summary(result, result1, sd, sd1, CI = 0.975)) %>%
 mutate(sd_b = (result_upr_b - result_lwr_b)/2/1.96) %>%
 ungroup() %>%
 select(region, result_b, sd_b, result_lwr_b, result_upr_b)
 }
 if (outcome %in% c("h0", "h1", "h2", "h3", "h4", "h5")){
 dths_stroke_global <- df_in %>%
 filter(outcome == !!outcome,
 year == !!year,
 cause == !!cause) %>%
 arrange(region, country_abbrev, year, sex, ilo_age_bands_5yr) %>%
 mutate(sd = (result_upr - result_lwr)/2/1.96) %>%
 left_join(who_ilo_long_populations, by = c("region", "country_abbrev", "year", "sex", "ilo_age_bands_5yr" = "age_bands_5yr")) %>%
 mutate(result = result*population,
 sd = sd*population) %>%
 drop_na() %>%
 group_by(region) %>%
 summarise(result = sum(result),
 sd = sqrt(sum((sd)^2)),
 population = sum(population)) %>%
 mutate(result_lwr = qnorm(p = 0.025, mean = result, sd = sd)/ population,
 result_upr = qnorm(p = 0.975, mean = result, sd = sd)/ population,
 result = result / population,
 sd = sd/population) %>%
 select(-population) %>%
 ungroup %>%
 mutate(sex = "b") %>%
 pivot_wider(names_from = sex, values_from = result:result_upr)
 dths_stroke_global[dths_stroke_global < 0] <- 0
 }
 if (outcome %in% c("h0_number", "h1_number", "h2_number", "h3_number", "h4_number", "h5_number")){
 outcome2 <- str_sub(outcome, end = -8L)
 dths_stroke_global <- df_in %>%
 filter(outcome == !!outcome2,
 year == !!year,
 cause == !!cause) %>%
 arrange(region, country_abbrev, year, sex, ilo_age_bands_5yr) %>%
 mutate(sd = (result_upr - result_lwr)/2/1.96) %>%
 left_join(who_ilo_long_populations, by = c("region", "country_abbrev", "year", "sex", "ilo_age_bands_5yr" = "age_bands_5yr")) %>%
 mutate(population = population * 1000) %>%
 mutate(result = result*population,
 sd = sd*population) %>%
 drop_na() %>%
 group_by(region) %>%
 summarise(result = sum(result),
 sd = sqrt(sum((sd)^2)),
 population = sum(population)) %>%
 mutate(result_lwr = qnorm(p = 0.025, mean = result, sd = sd),
 result_upr = qnorm(p = 0.975, mean = result, sd = sd),
 result = result,
 sd = sd) %>%
 select(-population) %>%
 ungroup %>%
 mutate(sex = "b") %>%
 pivot_wider(names_from = sex, values_from = result:result_upr)
 dths_stroke_global[dths_stroke_global < 0] <- 0
 }
 if (outcome %in% c("DTH", "DALY", "DTHenvelope", "DALYenvelope")) {
 dths_stroke_global <- df_in %>%
 filter(outcome == !!outcome,
 year == !!year,
 cause == !!cause) %>%
 arrange(region, country_abbrev, year, sex, ilo_age_bands_5yr) %>%
 mutate(sd = (result_upr - result_lwr)/2/1.96) %>%
 drop_na(region, result) %>%
 group_by(region) %>%
 summarise(result = sum(result),
 sd = sqrt(sum((sd)^2))) %>%
 mutate(result_lwr = qnorm(p = 0.025, mean = result, sd = sd),
 result_upr = qnorm(p = 0.975, mean = result, sd = sd)) %>%
 ungroup %>%
 mutate(sex = "b") %>%
 pivot_wider(names_from = sex, values_from = result:result_upr)
 }
 if (outcome %in% c("DTHS", "DALYS")) {
 outcome2 <- str_sub(outcome, end = -2L)
 dths_stroke_global <- df_in %>%
 filter(outcome == !!outcome2,
 year == !!year,
 cause %in% c("stroke_h5", "ihd_h5")) %>%
 arrange(region, country_abbrev, year, sex, ilo_age_bands_5yr) %>%
 mutate(sd = (result_upr - result_lwr)/2/1.96) %>%
 drop_na() %>%
 group_by(region) %>%
 summarise(result = sum(result),
 sd = sqrt(sum((sd)^2))) %>%
 mutate(result_lwr = qnorm(p = 0.025, mean = result, sd = sd),
 result_upr = qnorm(p = 0.975, mean = result, sd = sd)) %>%
 ungroup %>%
 mutate(sex = "b") %>%
 pivot_wider(names_from = sex, values_from = result:result_upr)
 }
 if (outcome %in% c("h45")){
 dths_stroke_global <- df_in %>%
 filter(outcome %in% c("h4", "h5"),
 year == !!year,
 cause == !!cause) %>%
 arrange(region, country_abbrev, year, sex, ilo_age_bands_5yr) %>%
 mutate(sd = (result_upr - result_lwr)/2/1.96) %>%
 left_join(who_ilo_long_populations, by = c("region", "country_abbrev", "year", "sex", "ilo_age_bands_5yr" = "age_bands_5yr")) %>%
 mutate(result = result*population,
 sd = sd*population) %>%
 drop_na() %>%
 group_by(region) %>%
 summarise(result = sum(result),
 sd = sqrt(sum((sd)^2)),
 population = sum(population)/2) %>%
 mutate(result_lwr = qnorm(p = 0.025, mean = result, sd = sd)/ population,
 result_upr = qnorm(p = 0.975, mean = result, sd = sd)/ population,
 result = result / population,
 sd = sd/population) %>%
 select(-population) %>%
 ungroup %>%
 mutate(sex = "b") %>%
 pivot_wider(names_from = sex, values_from = result:result_upr)
 dths_stroke_global[dths_stroke_global < 0] <- 0
 }
 if (outcome %in% c("DTHrate")){
 outcome2 <- case_when(outcome == "DTHrate" ~ "DTH",
 outcome == "DALYrate" ~ "DALY",
 TRUE ~ NA_character_)
 dths_stroke_global <- df_in %>%
 filter(outcome == !!outcome2,
 year == !!year,
 cause == !!cause) %>%
 arrange(region, country_abbrev, year, sex, ilo_age_bands_5yr) %>%
 mutate(sd = (result_upr - result_lwr)/2/1.96) %>%
 left_join(who_ilo_long_populations, by = c("region", "country_abbrev", "year", "sex", "ilo_age_bands_5yr" = "age_bands_5yr")) %>%
 drop_na() %>%
 group_by(region) %>%
 summarise(result = sum(result),
 sd = sqrt(sum((sd)^2)),
 population = sum(population)) %>%
 mutate(population = population / 100,
 result = result / population,
 sd = sd / population,
 result_lwr = qnorm(p = 0.025, mean = result, sd = sd),
 result_upr = qnorm(p = 0.975, mean = result, sd = sd)) %>%
 select(-population) %>%
 ungroup %>%
 mutate(sex = "b") %>%
 pivot_wider(names_from = sex, values_from = result:result_upr)
 }
 if (outcome %in% c("DALYrate")){
 outcome2 <- case_when(outcome == "DTHrate" ~ "DTH",
 outcome == "DALYrate" ~ "DALY",
 TRUE ~ NA_character_)
 dths_stroke_global <- df_in %>%
 filter(outcome == !!outcome2,
 year == !!year,
 cause == !!cause) %>%
 arrange(region, country_abbrev, year, sex, ilo_age_bands_5yr) %>%
 mutate(sd = (result_upr - result_lwr)/2/1.96) %>%
 left_join(who_ilo_long_populations, by = c("region", "country_abbrev", "year", "sex", "ilo_age_bands_5yr" = "age_bands_5yr")) %>%
 drop_na() %>%
 group_by(region) %>%
 summarise(result = sum(result),
 sd = sqrt(sum((sd)^2)),
 population = sum(population)) %>%
 mutate(population = population,
 result = result / population * 100000,
 sd = sd / population * 100000,
 result_lwr = qnorm(p = 0.025, mean = result, sd = sd),
 result_upr = qnorm(p = 0.975, mean = result, sd = sd)) %>%
 select(-population) %>%
 ungroup %>%
 mutate(sex = "b") %>%
 pivot_wider(names_from = sex, values_from = result:result_upr)
 }
 if (outcome %in% c("population")){
 dths_stroke_global <- who_ilo_long_populations %>%
 filter(age_bands_5yr %in% c("15_19", "20_24", "25_29", "30_34", "35_39", "40_44", "45_49", "50_54", "55_59", "60_64", "65_69", "70_74", "75_79", "80_84", "85_89", "90_94", "95_"),
 year == !!year) %>%
 arrange(region, country_abbrev, year, sex, age_bands_5yr) %>%
 drop_na() %>%
 group_by(region) %>%
 summarise(result_b = sum(population)) %>%
 ungroup %>%
 mutate(sd_b = NA,
 result_lwr_b = NA,
 result_upr_b = NA)
 }
 if (outcome %in% c("DTHratio")){
 envelope_stroke_region <- df_in %>%
 filter(outcome == "DTHparent",
 year == !!year,
 cause == !!cause) %>%
 arrange(region, country_abbrev, year, sex, ilo_age_bands_5yr) %>%
 drop_na() %>%
 group_by(region) %>%
 mutate(sd = (result_upr - result_lwr)/2/1.96) %>%
 summarise(result = sum(result),
 sd = sqrt(sum((sd)^2))) %>%
 mutate(result_lwr = qnorm(p = 0.025, mean = result, sd = sd),
 result_upr = qnorm(p = 0.975, mean = result, sd = sd)) %>%
 ungroup

 dths_stroke_global <- df_in %>%
 filter(outcome == "DTHenvelope",
 year == !!year,
 cause == !!cause) %>%
 arrange(region, country_abbrev, year, sex, ilo_age_bands_5yr) %>%
 drop_na() %>%
 group_by(region) %>%
 mutate(sd = (result_upr - result_lwr)/2/1.96) %>%
 summarise(result = sum(result),
 sd = sqrt(sum((sd)^2))) %>%
 mutate(result_lwr = qnorm(p = 0.025, mean = result, sd = sd),
 result_upr = qnorm(p = 0.975, mean = result, sd = sd)) %>%
 ungroup %>%

 select_all(.funs = funs(str_c(., "1")))

 dths_stroke_global <- bind_cols(dths_stroke_global, envelope_stroke_region) %>%
 rowwise() %>%
 transmute(region = region,
 result_b = whoilo_paf_summary(result, result1, CI = NULL),
 result_lwr_b = whoilo_paf_summary(result, result1, sd, sd1, CI = 0.025),
 result_upr_b = whoilo_paf_summary(result, result1, sd, sd1, CI = 0.975)) %>%
 mutate(sd_b = (result_upr_b - result_lwr_b)/2/1.96) %>%
 ungroup() %>%
 select(region, result_b, sd_b, result_lwr_b, result_upr_b)
 }
 if (outcome %in% c("DALYratio")){
 envelope_stroke_region <- df_in %>%
 filter(outcome == "DALYparent",
 year == !!year,
 cause == !!cause) %>%
 arrange(region, country_abbrev, year, sex, ilo_age_bands_5yr) %>%
 drop_na() %>%
 group_by(region) %>%
 mutate(sd = (result_upr - result_lwr)/2/1.96) %>%
 summarise(result = sum(result),
 sd = sqrt(sum((sd)^2))) %>%
 mutate(result_lwr = qnorm(p = 0.025, mean = result, sd = sd),
 result_upr = qnorm(p = 0.975, mean = result, sd = sd)) %>%
 ungroup

 dths_stroke_global <- df_in %>%
 filter(outcome == "DALYenvelope",
 year == !!year,
 cause == !!cause) %>%
 mutate(result_lwr = case_when(is.na(result_lwr) ~ result,
 TRUE ~ result_lwr),
 result_upr = case_when(is.na(result_upr) ~ result,
 TRUE ~ result_upr)) %>%
 arrange(region, country_abbrev, year, sex, ilo_age_bands_5yr) %>%
 drop_na() %>%
 group_by(region) %>%
 mutate(sd = (result_upr - result_lwr)/2/1.96) %>%
 summarise(result = sum(result),
 sd = sqrt(sum((sd)^2))) %>%
 mutate(result_lwr = qnorm(p = 0.025, mean = result, sd = sd),
 result_upr = qnorm(p = 0.975, mean = result, sd = sd)) %>%
 ungroup %>%

 select_all(.funs = funs(str_c(., "1")))

 dths_stroke_global <- bind_cols(dths_stroke_global, envelope_stroke_region) %>%
 rowwise() %>%
 transmute(region = region,
 result_b = whoilo_paf_summary(result, result1, CI = NULL),
 result_lwr_b = whoilo_paf_summary(result, result1, sd, sd1, CI = 0.025),
 result_upr_b = whoilo_paf_summary(result, result1, sd, sd1, CI = 0.975)) %>%
 mutate(sd_b = (result_upr_b - result_lwr_b)/2/1.96) %>%
 ungroup() %>%
 select(region, result_b, sd_b, result_lwr_b, result_upr_b)
 }
 }
 # by sex
 if ("sex" %in% breakdown){
 if (outcome %in% c("PAF", "DTHpaf")){
 envelope_stroke_sex_region <- df_in %>%
 filter(outcome == "DTHenvelope",
 year == !!year,
 cause == !!cause) %>%
 arrange(region, country_abbrev, year, sex, ilo_age_bands_5yr) %>%
 drop_na() %>%
 mutate(sd = (result_upr - result_lwr)/2/1.96) %>%
 group_by(region, sex) %>%
 summarise(result = sum(result),
 sd = sqrt(sum((sd)^2))) %>%
 ungroup %>%
 pivot_wider(names_from = sex, values_from = result:sd) %>%

 select_all(.funs = funs(str_c(., "1")))

 dths_stroke_sex_global <- df_in %>%
 filter(outcome == "DTH",
 year == !!year,
 cause == !!cause) %>%
 arrange(region, country_abbrev, year, sex, ilo_age_bands_5yr) %>%
 drop_na() %>%
 mutate(sd = (result_upr - result_lwr)/2/1.96) %>%
 group_by(region, sex) %>%
 summarise(result = sum(result),
 sd = sqrt(sum((sd)^2))) %>%
 ungroup %>%
 pivot_wider(names_from = sex, values_from = result:sd)
 dths_stroke_sex_global <- bind_cols(dths_stroke_sex_global, envelope_stroke_sex_region) %>%
 rowwise() %>%
 transmute(region = region,
 result_lwr_f = whoilo_paf_summary(result_f, result_f1, sd_f, sd_f1, CI = 0.025),
 result_upr_f = whoilo_paf_summary(result_f, result_f1, sd_f, sd_f1, CI = 0.975),
 result_f = whoilo_paf_summary(result_f, result_f1, CI = NULL),
 result_lwr_m = whoilo_paf_summary(result_m, result_m1, sd_m, sd_m1, CI = 0.025),
 result_upr_m = whoilo_paf_summary(result_m, result_m1, sd_m, sd_m1, CI = 0.975),
 result_m = whoilo_paf_summary(result_m, result_m1, CI = NULL)) %>%
 mutate(sd_f = (result_upr_f - result_lwr_f)/2/1.96,
 sd_m = (result_upr_m - result_lwr_m)/2/1.96) %>%
 ungroup() %>%
 select(region, result_f, sd_f, result_lwr_f, result_upr_f, result_m, sd_m, result_lwr_m, result_upr_m)
 }
 if (outcome %in% c("DALYpaf")){
 envelope_stroke_sex_region <- df_in %>%
 filter(outcome == "DALYenvelope",
 year == !!year,
 cause == !!cause) %>%
 mutate(result_lwr = case_when(is.na(result_lwr) ~ result,
 TRUE ~ result_lwr),
 result_upr = case_when(is.na(result_upr) ~ result,
 TRUE ~ result_upr)) %>%
 arrange(region, country_abbrev, year, sex, ilo_age_bands_5yr) %>%
 drop_na() %>%
 mutate(sd = (result_upr - result_lwr)/2/1.96) %>%
 group_by(region, sex) %>%
 summarise(result = sum(result),
 sd = sqrt(sum((sd)^2))) %>%
 ungroup %>%
 pivot_wider(names_from = sex, values_from = result:sd) %>%

 select_all(.funs = funs(str_c(., "1")))

 dths_stroke_sex_global <- df_in %>%
 filter(outcome == "DALY",
 year == !!year,
 cause == !!cause) %>%
 arrange(region, country_abbrev, year, sex, ilo_age_bands_5yr) %>%
 drop_na() %>%
 mutate(sd = (result_upr - result_lwr)/2/1.96) %>%
 group_by(region, sex) %>%
 summarise(result = sum(result),
 sd = sqrt(sum((sd)^2))) %>%
 ungroup %>%
 pivot_wider(names_from = sex, values_from = result:sd)
 dths_stroke_sex_global <- bind_cols(dths_stroke_sex_global, envelope_stroke_sex_region) %>%
 rowwise() %>%
 transmute(region = region,
 result_lwr_f = whoilo_paf_summary(result_f, result_f1, sd_f, sd_f1, CI = 0.025),
 result_upr_f = whoilo_paf_summary(result_f, result_f1, sd_f, sd_f1, CI = 0.975),
 result_f = whoilo_paf_summary(result_f, result_f1, CI = NULL),
 result_lwr_m = whoilo_paf_summary(result_m, result_m1, sd_m, sd_m1, CI = 0.025),
 result_upr_m = whoilo_paf_summary(result_m, result_m1, sd_m, sd_m1, CI = 0.975),
 result_m = whoilo_paf_summary(result_m, result_m1, CI = NULL)) %>%
 mutate(sd_f = (result_upr_f - result_lwr_f)/2/1.96,
 sd_m = (result_upr_m - result_lwr_m)/2/1.96) %>%
 ungroup() %>%
 select(region, result_f, sd_f, result_lwr_f, result_upr_f, result_m, sd_m, result_lwr_m, result_upr_m)
 }
 if (outcome %in% c("h0", "h1", "h2", "h3", "h4", "h5")){
 dths_stroke_sex_global <- df_in %>%
 filter(outcome == !!outcome,
 year == !!year,
 cause == !!cause) %>%
 arrange(region, country_abbrev, year, sex, ilo_age_bands_5yr) %>%
 mutate(sd = (result_upr - result_lwr)/2/1.96) %>%
 group_by(region, sex) %>%
 left_join(who_ilo_long_populations, by = c("region", "country_abbrev", "year", "sex", "ilo_age_bands_5yr" = "age_bands_5yr")) %>%
 mutate(result = result*population,
 sd = sd*population) %>%
 drop_na() %>%
 summarise(result = sum(result),
 sd = sqrt(sum((sd)^2)),
 population = sum(population)) %>%
 mutate(result_lwr = qnorm(p = 0.025, mean = result, sd = sd)/ population,
 result_upr = qnorm(p = 0.975, mean = result, sd = sd)/ population,
 result = result / population,
 sd = sd/population) %>%
 select(-population) %>%
 ungroup %>%
 pivot_wider(names_from = sex, values_from = result:result_upr) %>%
 select(region, result_f, sd_f, result_lwr_f, result_upr_f, result_m, sd_m, result_lwr_m, result_upr_m)
 dths_stroke_sex_global[dths_stroke_sex_global < 0] <- 0
 }
 if (outcome %in% c("h0_number", "h1_number", "h2_number", "h3_number", "h4_number", "h5_number")){
 outcome2 <- str_sub(outcome, end = -8L)
 dths_stroke_sex_global <- df_in %>%
 filter(outcome == !!outcome2,
 year == !!year,
 cause == !!cause) %>%
 arrange(region, country_abbrev, year, sex, ilo_age_bands_5yr) %>%
 mutate(sd = (result_upr - result_lwr)/2/1.96) %>%
 group_by(region, sex) %>%
 left_join(who_ilo_long_populations, by = c("region", "country_abbrev", "year", "sex", "ilo_age_bands_5yr" = "age_bands_5yr")) %>%
 mutate(population = population * 1000) %>%
 mutate(result = result*population,
 sd = sd*population) %>%
 drop_na() %>%
 summarise(result = sum(result),
 sd = sqrt(sum((sd)^2)),
 population = sum(population)) %>%
 mutate(result_lwr = qnorm(p = 0.025, mean = result, sd = sd),
 result_upr = qnorm(p = 0.975, mean = result, sd = sd),
 result = result,
 sd = sd) %>%
 select(-population) %>%
 ungroup %>%
 pivot_wider(names_from = sex, values_from = result:result_upr) %>%
 select(region, result_f, sd_f, result_lwr_f, result_upr_f, result_m, sd_m, result_lwr_m, result_upr_m)
 dths_stroke_sex_global[dths_stroke_sex_global < 0] <- 0
 }
 if (outcome %in% c("DTH", "DALY", "DTHenvelope", "DALYenvelope")) {
 dths_stroke_sex_global <- df_in %>%
 filter(outcome == !!outcome,
 year == !!year,
 cause == !!cause) %>%
 arrange(region, country_abbrev, year, sex, ilo_age_bands_5yr) %>%
 mutate(sd = (result_upr - result_lwr)/2/1.96) %>%
 drop_na(region, result) %>%
 group_by(region, sex) %>%
 summarise(result = sum(result),
 sd = sqrt(sum((sd)^2))) %>%
 mutate(result_lwr = qnorm(p = 0.025, mean = result, sd = sd),
 result_upr = qnorm(p = 0.975, mean = result, sd = sd)) %>%
 ungroup %>%
 pivot_wider(names_from = sex, values_from = result:result_upr) %>%
 select(region, result_f, sd_f, result_lwr_f, result_upr_f, result_m, sd_m, result_lwr_m, result_upr_m)
 }
 if (outcome %in% c("DTHS", "DALYS")) {
 outcome2 <- str_sub(outcome, end = -2L)
 dths_stroke_sex_global <- df_in %>%
 filter(outcome == !!outcome2,
 year == !!year,
 cause %in% c("stroke_h5", "ihd_h5")) %>%
 arrange(region, country_abbrev, year, sex, ilo_age_bands_5yr) %>%
 mutate(sd = (result_upr - result_lwr)/2/1.96) %>%
 drop_na() %>%
 group_by(region, sex) %>%
 summarise(result = sum(result),
 sd = sqrt(sum((sd)^2))) %>%
 mutate(result_lwr = qnorm(p = 0.025, mean = result, sd = sd),
 result_upr = qnorm(p = 0.975, mean = result, sd = sd)) %>%
 ungroup %>%
 pivot_wider(names_from = sex, values_from = result:result_upr) %>%
 select(region, result_f, sd_f, result_lwr_f, result_upr_f, result_m, sd_m, result_lwr_m, result_upr_m)
 }
 if (outcome %in% c("h45")){
 dths_stroke_sex_global <- df_in %>%
 filter(outcome %in% c("h4", "h5"),
 year == !!year,
 cause == !!cause) %>%
 arrange(region, country_abbrev, year, sex, ilo_age_bands_5yr) %>%
 mutate(sd = (result_upr - result_lwr)/2/1.96) %>%
 group_by(region, sex) %>%
 left_join(who_ilo_long_populations, by = c("region", "country_abbrev", "year", "sex", "ilo_age_bands_5yr" = "age_bands_5yr")) %>%
 mutate(result = result*population,
 sd = sd*population) %>%
 drop_na() %>%
 summarise(result = sum(result),
 sd = sqrt(sum((sd)^2)),
 population = sum(population)/2) %>%
 mutate(result_lwr = qnorm(p = 0.025, mean = result, sd = sd)/ population,
 result_upr = qnorm(p = 0.975, mean = result, sd = sd)/ population,
 result = result / population,
 sd = sd/population) %>%
 select(-population) %>%
 ungroup %>%
 pivot_wider(names_from = sex, values_from = result:result_upr) %>%
 select(region, result_f, sd_f, result_lwr_f, result_upr_f, result_m, sd_m, result_lwr_m, result_upr_m)
 dths_stroke_sex_global[dths_stroke_sex_global < 0] <- 0
 }
 if (outcome %in% c("DTHrate")){
 outcome2 <- case_when(outcome == "DTHrate" ~ "DTH",
 outcome == "DALYrate" ~ "DALY",
 TRUE ~ NA_character_)
 dths_stroke_sex_global <- df_in %>%
 filter(outcome == !!outcome2,
 year == !!year,
 cause == !!cause) %>%
 arrange(region, country_abbrev, year, sex, ilo_age_bands_5yr) %>%
 mutate(sd = (result_upr - result_lwr)/2/1.96) %>%
 group_by(region, sex) %>%
 left_join(who_ilo_long_populations, by = c("region", "country_abbrev", "year", "sex", "ilo_age_bands_5yr" = "age_bands_5yr")) %>%
 drop_na() %>%
 summarise(result = sum(result),
 sd = sqrt(sum((sd)^2)),
 population = sum(population)) %>%
 mutate(population = population / 100,
 result = result / population,
 sd = sd / population,
 result_lwr = qnorm(p = 0.025, mean = result, sd = sd),
 result_upr = qnorm(p = 0.975, mean = result, sd = sd)) %>%
 select(-population) %>%
 ungroup %>%
 pivot_wider(names_from = sex, values_from = result:result_upr) %>%
 select(region, result_f, sd_f, result_lwr_f, result_upr_f, result_m, sd_m, result_lwr_m, result_upr_m)
 }
 if (outcome %in% c("DALYrate")){
 outcome2 <- case_when(outcome == "DTHrate" ~ "DTH",
 outcome == "DALYrate" ~ "DALY",
 TRUE ~ NA_character_)
 dths_stroke_sex_global <- df_in %>%
 filter(outcome == !!outcome2,
 year == !!year,
 cause == !!cause) %>%
 arrange(region, country_abbrev, year, sex, ilo_age_bands_5yr) %>%
 mutate(sd = (result_upr - result_lwr)/2/1.96) %>%
 group_by(region, sex) %>%
 left_join(who_ilo_long_populations, by = c("region", "country_abbrev", "year", "sex", "ilo_age_bands_5yr" = "age_bands_5yr")) %>%
 drop_na() %>%
 summarise(result = sum(result),
 sd = sqrt(sum((sd)^2)),
 population = sum(population)) %>%
 mutate(population = population,
 result = result / population * 100000,
 sd = sd / population * 100000,
 result_lwr = qnorm(p = 0.025, mean = result, sd = sd),
 result_upr = qnorm(p = 0.975, mean = result, sd = sd)) %>%
 select(-population) %>%
 ungroup %>%
 pivot_wider(names_from = sex, values_from = result:result_upr) %>%
 select(region, result_f, sd_f, result_lwr_f, result_upr_f, result_m, sd_m, result_lwr_m, result_upr_m)
 }
 if (outcome %in% c("population")){
 dths_stroke_sex_global <- who_ilo_long_populations %>%
 filter(age_bands_5yr %in% c("15_19", "20_24", "25_29", "30_34", "35_39", "40_44", "45_49", "50_54", "55_59", "60_64", "65_69", "70_74", "75_79", "80_84", "85_89", "90_94", "95_"),
 year == !!year) %>%
 arrange(region, country_abbrev, year, sex, age_bands_5yr) %>%
 drop_na() %>%
 group_by(region, sex) %>%
 summarise(result = sum(population)) %>%
 mutate(sd = NA,
 result_lwr = NA,
 result_upr = NA) %>%
 ungroup %>%
 pivot_wider(names_from = sex, values_from = result:result_upr) %>%
 select(region,
 contains("_f"), contains("_m"))
 }
 if (outcome %in% c("DTHratio")){
 envelope_stroke_sex_region <- df_in %>%
 filter(outcome == "DTHparent",
 year == !!year,
 cause == !!cause) %>%
 arrange(region, country_abbrev, year, sex, ilo_age_bands_5yr) %>%
 drop_na() %>%
 mutate(sd = (result_upr - result_lwr)/2/1.96) %>%
 group_by(region, sex) %>%
 summarise(result = sum(result),
 sd = sqrt(sum((sd)^2))) %>%
 ungroup %>%
 pivot_wider(names_from = sex, values_from = result:sd)

 dths_stroke_sex_global <- df_in %>%
 filter(outcome == "DTHenvelope",
 year == !!year,
 cause == !!cause) %>%
 arrange(region, country_abbrev, year, sex, ilo_age_bands_5yr) %>%
 drop_na() %>%
 mutate(sd = (result_upr - result_lwr)/2/1.96) %>%
 group_by(region, sex) %>%
 summarise(result = sum(result),
 sd = sqrt(sum((sd)^2))) %>%
 ungroup %>%
 pivot_wider(names_from = sex, values_from = result:sd) %>%

 select_all(.funs = funs(str_c(., "1")))

 dths_stroke_sex_global <- bind_cols(dths_stroke_sex_global, envelope_stroke_sex_region) %>%
 rowwise() %>%
 transmute(region = region,
 result_lwr_f = whoilo_paf_summary(result_f, result_f1, sd_f, sd_f1, CI = 0.025),
 result_upr_f = whoilo_paf_summary(result_f, result_f1, sd_f, sd_f1, CI = 0.975),
 result_f = whoilo_paf_summary(result_f, result_f1, CI = NULL),
 result_lwr_m = whoilo_paf_summary(result_m, result_m1, sd_m, sd_m1, CI = 0.025),
 result_upr_m = whoilo_paf_summary(result_m, result_m1, sd_m, sd_m1, CI = 0.975),
 result_m = whoilo_paf_summary(result_m, result_m1, CI = NULL)) %>%
 mutate(sd_f = (result_upr_f - result_lwr_f)/2/1.96,
 sd_m = (result_upr_m - result_lwr_m)/2/1.96) %>%
 ungroup() %>%
 select(region, result_f, sd_f, result_lwr_f, result_upr_f, result_m, sd_m, result_lwr_m, result_upr_m)
 }
 if (outcome %in% c("DALYratio")){
 envelope_stroke_sex_region <- df_in %>%
 filter(outcome == "DALYparent",
 year == !!year,
 cause == !!cause) %>%
 arrange(region, country_abbrev, year, sex, ilo_age_bands_5yr) %>%
 drop_na() %>%
 mutate(sd = (result_upr - result_lwr)/2/1.96) %>%
 group_by(region, sex) %>%
 summarise(result = sum(result),
 sd = sqrt(sum((sd)^2))) %>%
 ungroup %>%
 pivot_wider(names_from = sex, values_from = result:sd)

 dths_stroke_sex_global <- df_in %>%
 filter(outcome == "DALYenvelope",
 year == !!year,
 cause == !!cause) %>%
 mutate(result_lwr = case_when(is.na(result_lwr) ~ result,
 TRUE ~ result_lwr),
 result_upr = case_when(is.na(result_upr) ~ result,
 TRUE ~ result_upr)) %>%
 arrange(region, country_abbrev, year, sex, ilo_age_bands_5yr) %>%
 drop_na() %>%
 mutate(sd = (result_upr - result_lwr)/2/1.96) %>%
 group_by(region, sex) %>%
 summarise(result = sum(result),
 sd = sqrt(sum((sd)^2))) %>%
 ungroup %>%
 pivot_wider(names_from = sex, values_from = result:sd) %>%

 select_all(.funs = funs(str_c(., "1")))

 dths_stroke_sex_global <- bind_cols(dths_stroke_sex_global, envelope_stroke_sex_region) %>%
 rowwise() %>%
 transmute(region = region,
 result_lwr_f = whoilo_paf_summary(result_f, result_f1, sd_f, sd_f1, CI = 0.025),
 result_upr_f = whoilo_paf_summary(result_f, result_f1, sd_f, sd_f1, CI = 0.975),
 result_f = whoilo_paf_summary(result_f, result_f1, CI = NULL),
 result_lwr_m = whoilo_paf_summary(result_m, result_m1, sd_m, sd_m1, CI = 0.025),
 result_upr_m = whoilo_paf_summary(result_m, result_m1, sd_m, sd_m1, CI = 0.975),
 result_m = whoilo_paf_summary(result_m, result_m1, CI = NULL)) %>%
 mutate(sd_f = (result_upr_f - result_lwr_f)/2/1.96,
 sd_m = (result_upr_m - result_lwr_m)/2/1.96) %>%
 ungroup() %>%
 select(region, result_f, sd_f, result_lwr_f, result_upr_f, result_m, sd_m, result_lwr_m, result_upr_m)
 }
 }
 # by age
 if ("age" %in% breakdown){
 if (outcome %in% c("PAF", "DTHpaf")){
 envelope_stroke_age_global <- df_in %>%
 filter(outcome == "DTHenvelope",
 year == !!year,
 cause == !!cause) %>%
 arrange(region, country_abbrev, year, sex, ilo_age_bands_5yr) %>%
 drop_na() %>%
 mutate(sd = (result_upr - result_lwr)/2/1.96) %>%
 group_by(region, ilo_age_bands_5yr) %>%
 summarise(result = sum(result),
 sd = sqrt(sum((sd)^2))) %>%
 ungroup %>%
 pivot_wider(names_from = ilo_age_bands_5yr, values_from = result:sd) %>%

 select_all(.funs = funs(str_c(., "1")))

 dths_stroke_age_global <- df_in %>%
 filter(outcome == "DTH",
 year == !!year,
 cause == !!cause) %>%
 arrange(region, country_abbrev, year, sex, ilo_age_bands_5yr) %>%
 drop_na() %>%
 mutate(sd = (result_upr - result_lwr)/2/1.96) %>%
 group_by(region, ilo_age_bands_5yr) %>%
 summarise(result = sum(result),
 sd = sqrt(sum((sd)^2))) %>%
 ungroup %>%
 pivot_wider(names_from = ilo_age_bands_5yr, values_from = result:sd)
 dths_stroke_age_global <- bind_cols(dths_stroke_age_global, envelope_stroke_age_global) %>%
 rowwise() %>%
 transmute(region = region,
 result_lwr_b_15_19 = whoilo_paf_summary(result_15_19, result_15_191, sd_15_19, sd_15_191, CI = 0.025),
 result_upr_b_15_19 = whoilo_paf_summary(result_15_19, result_15_191, sd_15_19, sd_15_191, CI = 0.975),
 result_b_15_19 = whoilo_paf_summary(result_15_19, result_15_191, CI = NULL),
 result_lwr_b_20_24 = whoilo_paf_summary(result_20_24, result_20_241, sd_20_24, sd_20_241, CI = 0.025),
 result_upr_b_20_24 = whoilo_paf_summary(result_20_24, result_20_241, sd_20_24, sd_20_241, CI = 0.975),
 result_b_20_24 = whoilo_paf_summary(result_20_24, result_20_241, CI = NULL),
 result_lwr_b_25_29 = whoilo_paf_summary(result_25_29, result_25_291, sd_25_29, sd_25_291, CI = 0.025),
 result_upr_b_25_29 = whoilo_paf_summary(result_25_29, result_25_291, sd_25_29, sd_25_291, CI = 0.975),
 result_b_25_29 = whoilo_paf_summary(result_25_29, result_25_291, CI = NULL),
 result_lwr_b_30_34 = whoilo_paf_summary(result_30_34, result_30_341, sd_30_34, sd_30_341, CI = 0.025),
 result_upr_b_30_34 = whoilo_paf_summary(result_30_34, result_30_341, sd_30_34, sd_30_341, CI = 0.975),
 result_b_30_34 = whoilo_paf_summary(result_30_34, result_30_341, CI = NULL),
 result_lwr_b_35_39 = whoilo_paf_summary(result_35_39, result_35_391, sd_35_39, sd_35_391, CI = 0.025),
 result_upr_b_35_39 = whoilo_paf_summary(result_35_39, result_35_391, sd_35_39, sd_35_391, CI = 0.975),
 result_b_35_39 = whoilo_paf_summary(result_35_39, result_35_391, CI = NULL),
 result_lwr_b_40_44 = whoilo_paf_summary(result_40_44, result_40_441, sd_40_44, sd_40_441, CI = 0.025),
 result_upr_b_40_44 = whoilo_paf_summary(result_40_44, result_40_441, sd_40_44, sd_40_441, CI = 0.975),
 result_b_40_44 = whoilo_paf_summary(result_40_44, result_40_441, CI = NULL),
 result_lwr_b_45_49 = whoilo_paf_summary(result_45_49, result_45_491, sd_45_49, sd_45_491, CI = 0.025),
 result_upr_b_45_49 = whoilo_paf_summary(result_45_49, result_45_491, sd_45_49, sd_45_491, CI = 0.975),
 result_b_45_49 = whoilo_paf_summary(result_45_49, result_45_491, CI = NULL),
 result_lwr_b_50_54 = whoilo_paf_summary(result_50_54, result_50_541, sd_50_54, sd_50_541, CI = 0.025),
 result_upr_b_50_54 = whoilo_paf_summary(result_50_54, result_50_541, sd_50_54, sd_50_541, CI = 0.975),
 result_b_50_54 = whoilo_paf_summary(result_50_54, result_50_541, CI = NULL),
 result_lwr_b_55_59 = whoilo_paf_summary(result_55_59, result_55_591, sd_55_59, sd_55_591, CI = 0.025),
 result_upr_b_55_59 = whoilo_paf_summary(result_55_59, result_55_591, sd_55_59, sd_55_591, CI = 0.975),
 result_b_55_59 = whoilo_paf_summary(result_55_59, result_55_591, CI = NULL),
 result_lwr_b_60_64 = whoilo_paf_summary(result_60_64, result_60_641, sd_60_64, sd_60_641, CI = 0.025),
 result_upr_b_60_64 = whoilo_paf_summary(result_60_64, result_60_641, sd_60_64, sd_60_641, CI = 0.975),
 result_b_60_64 = whoilo_paf_summary(result_60_64, result_60_641, CI = NULL),
 result_lwr_b_65_69 = whoilo_paf_summary(result_65_69, result_65_691, sd_65_69, sd_65_691, CI = 0.025),
 result_upr_b_65_69 = whoilo_paf_summary(result_65_69, result_65_691, sd_65_69, sd_65_691, CI = 0.975),
 result_b_65_69 = whoilo_paf_summary(result_65_69, result_65_691, CI = NULL),
 result_lwr_b_70_74 = whoilo_paf_summary(result_70_74, result_70_741, sd_70_74, sd_70_741, CI = 0.025),
 result_upr_b_70_74 = whoilo_paf_summary(result_70_74, result_70_741, sd_70_74, sd_70_741, CI = 0.975),
 result_b_70_74 = whoilo_paf_summary(result_70_74, result_70_741, CI = NULL),
 result_lwr_b_75_79 = whoilo_paf_summary(result_75_79, result_75_791, sd_75_79, sd_75_791, CI = 0.025),
 result_upr_b_75_79 = whoilo_paf_summary(result_75_79, result_75_791, sd_75_79, sd_75_791, CI = 0.975),
 result_b_75_79 = whoilo_paf_summary(result_75_79, result_75_791, CI = NULL),
 result_lwr_b_80_84 = whoilo_paf_summary(result_80_84, result_80_841, sd_80_84, sd_80_841, CI = 0.025),
 result_upr_b_80_84 = whoilo_paf_summary(result_80_84, result_80_841, sd_80_84, sd_80_841, CI = 0.975),
 result_b_80_84 = whoilo_paf_summary(result_80_84, result_80_841, CI = NULL),
 result_lwr_b_85_89 = whoilo_paf_summary(result_85_89, result_85_891, sd_85_89, sd_85_891, CI = 0.025),
 result_upr_b_85_89 = whoilo_paf_summary(result_85_89, result_85_891, sd_85_89, sd_85_891, CI = 0.975),
 result_b_85_89 = whoilo_paf_summary(result_85_89, result_85_891, CI = NULL),
 result_lwr_b_90_94 = whoilo_paf_summary(result_90_94, result_90_941, sd_90_94, sd_90_941, CI = 0.025),
 result_upr_b_90_94 = whoilo_paf_summary(result_90_94, result_90_941, sd_90_94, sd_90_941, CI = 0.975),
 result_b_90_94 = whoilo_paf_summary(result_90_94, result_90_941, CI = NULL),
 result_lwr_b_95_ = whoilo_paf_summary(result_95_, result_95_1, sd_95_, sd_95_1, CI = 0.025),
 result_upr_b_95_ = whoilo_paf_summary(result_95_, result_95_1, sd_95_, sd_95_1, CI = 0.975),
 result_b_95_ = whoilo_paf_summary(result_95_, result_95_1, CI = NULL)) %>%
 ungroup() %>%
 mutate(sd_b_15_19 = (result_upr_b_15_19 - result_lwr_b_15_19)/2/1.96,
 sd_b_20_24 = (result_upr_b_20_24 - result_lwr_b_20_24)/2/1.96,
 sd_b_25_29 = (result_upr_b_25_29 - result_lwr_b_25_29)/2/1.96,
 sd_b_30_34 = (result_upr_b_30_34 - result_lwr_b_30_34)/2/1.96,
 sd_b_35_39 = (result_upr_b_35_39 - result_lwr_b_35_39)/2/1.96,
 sd_b_40_44 = (result_upr_b_40_44 - result_lwr_b_40_44)/2/1.96,
 sd_b_45_49 = (result_upr_b_45_49 - result_lwr_b_45_49)/2/1.96,
 sd_b_50_54 = (result_upr_b_50_54 - result_lwr_b_50_54)/2/1.96,
 sd_b_55_59 = (result_upr_b_55_59 - result_lwr_b_55_59)/2/1.96,
 sd_b_60_64 = (result_upr_b_60_64 - result_lwr_b_60_64)/2/1.96,
 sd_b_65_69 = (result_upr_b_65_69 - result_lwr_b_65_69)/2/1.96,
 sd_b_70_74 = (result_upr_b_70_74 - result_lwr_b_70_74)/2/1.96,
 sd_b_75_79 = (result_upr_b_75_79 - result_lwr_b_75_79)/2/1.96,
 sd_b_80_84 = (result_upr_b_80_84 - result_lwr_b_80_84)/2/1.96,
 sd_b_85_89 = (result_upr_b_85_89 - result_lwr_b_85_89)/2/1.96,
 sd_b_90_94 = (result_upr_b_90_94 - result_lwr_b_90_94)/2/1.96,
 sd_b_95_ = (result_upr_b_95_ - result_lwr_b_95_)/2/1.96) %>%
 select(region,
 result_b_15_19, sd_b_15_19, result_lwr_b_15_19, result_upr_b_15_19,
 result_b_20_24, sd_b_20_24, result_lwr_b_20_24, result_upr_b_20_24,
 result_b_25_29, sd_b_25_29, result_lwr_b_25_29, result_upr_b_25_29,
 result_b_30_34, sd_b_30_34, result_lwr_b_30_34, result_upr_b_30_34,
 result_b_35_39, sd_b_35_39, result_lwr_b_35_39, result_upr_b_35_39,
 result_b_40_44, sd_b_40_44, result_lwr_b_40_44, result_upr_b_40_44,
 result_b_45_49, sd_b_45_49, result_lwr_b_45_49, result_upr_b_45_49,
 result_b_50_54, sd_b_50_54, result_lwr_b_50_54, result_upr_b_50_54,
 result_b_55_59, sd_b_55_59, result_lwr_b_55_59, result_upr_b_55_59,
 result_b_60_64, sd_b_60_64, result_lwr_b_60_64, result_upr_b_60_64,
 result_b_65_69, sd_b_65_69, result_lwr_b_65_69, result_upr_b_65_69,
 result_b_70_74, sd_b_70_74, result_lwr_b_70_74, result_upr_b_70_74,
 result_b_75_79, sd_b_75_79, result_lwr_b_75_79, result_upr_b_75_79,
 result_b_80_84, sd_b_80_84, result_lwr_b_80_84, result_upr_b_80_84,
 result_b_85_89, sd_b_85_89, result_lwr_b_85_89, result_upr_b_85_89,
 result_b_90_94, sd_b_90_94, result_lwr_b_90_94, result_upr_b_90_94,
 result_b_95_, sd_b_95_, result_lwr_b_95_, result_upr_b_95_)
 }
 if (outcome %in% c("DALYpaf")){
 envelope_stroke_age_global <- df_in %>%
 filter(outcome == "DALYenvelope",
 year == !!year,
 cause == !!cause) %>%
 mutate(result_lwr = case_when(is.na(result_lwr) ~ result,
 TRUE ~ result_lwr),
 result_upr = case_when(is.na(result_upr) ~ result,
 TRUE ~ result_upr)) %>%
 arrange(region, country_abbrev, year, sex, ilo_age_bands_5yr) %>%
 drop_na() %>%
 mutate(sd = (result_upr - result_lwr)/2/1.96) %>%
 group_by(region, ilo_age_bands_5yr) %>%
 summarise(result = sum(result),
 sd = sqrt(sum((sd)^2))) %>%
 ungroup %>%
 pivot_wider(names_from = ilo_age_bands_5yr, values_from = result:sd) %>%

 select_all(.funs = funs(str_c(., "1")))

 dths_stroke_age_global <- df_in %>%
 filter(outcome == "DALY",
 year == !!year,
 cause == !!cause) %>%
 arrange(region, country_abbrev, year, sex, ilo_age_bands_5yr) %>%
 drop_na() %>%
 mutate(sd = (result_upr - result_lwr)/2/1.96) %>%
 group_by(region, ilo_age_bands_5yr) %>%
 summarise(result = sum(result),
 sd = sqrt(sum((sd)^2))) %>%
 ungroup %>%
 pivot_wider(names_from = ilo_age_bands_5yr, values_from = result:sd)
 dths_stroke_age_global <- bind_cols(dths_stroke_age_global, envelope_stroke_age_global) %>%
 rowwise() %>%
 transmute(region = region,
 result_lwr_b_15_19 = whoilo_paf_summary(result_15_19, result_15_191, sd_15_19, sd_15_191, CI = 0.025),
 result_upr_b_15_19 = whoilo_paf_summary(result_15_19, result_15_191, sd_15_19, sd_15_191, CI = 0.975),
 result_b_15_19 = whoilo_paf_summary(result_15_19, result_15_191, CI = NULL),
 result_lwr_b_20_24 = whoilo_paf_summary(result_20_24, result_20_241, sd_20_24, sd_20_241, CI = 0.025),
 result_upr_b_20_24 = whoilo_paf_summary(result_20_24, result_20_241, sd_20_24, sd_20_241, CI = 0.975),
 result_b_20_24 = whoilo_paf_summary(result_20_24, result_20_241, CI = NULL),
 result_lwr_b_25_29 = whoilo_paf_summary(result_25_29, result_25_291, sd_25_29, sd_25_291, CI = 0.025),
 result_upr_b_25_29 = whoilo_paf_summary(result_25_29, result_25_291, sd_25_29, sd_25_291, CI = 0.975),
 result_b_25_29 = whoilo_paf_summary(result_25_29, result_25_291, CI = NULL),
 result_lwr_b_30_34 = whoilo_paf_summary(result_30_34, result_30_341, sd_30_34, sd_30_341, CI = 0.025),
 result_upr_b_30_34 = whoilo_paf_summary(result_30_34, result_30_341, sd_30_34, sd_30_341, CI = 0.975),
 result_b_30_34 = whoilo_paf_summary(result_30_34, result_30_341, CI = NULL),
 result_lwr_b_35_39 = whoilo_paf_summary(result_35_39, result_35_391, sd_35_39, sd_35_391, CI = 0.025),
 result_upr_b_35_39 = whoilo_paf_summary(result_35_39, result_35_391, sd_35_39, sd_35_391, CI = 0.975),
 result_b_35_39 = whoilo_paf_summary(result_35_39, result_35_391, CI = NULL),
 result_lwr_b_40_44 = whoilo_paf_summary(result_40_44, result_40_441, sd_40_44, sd_40_441, CI = 0.025),
 result_upr_b_40_44 = whoilo_paf_summary(result_40_44, result_40_441, sd_40_44, sd_40_441, CI = 0.975),
 result_b_40_44 = whoilo_paf_summary(result_40_44, result_40_441, CI = NULL),
 result_lwr_b_45_49 = whoilo_paf_summary(result_45_49, result_45_491, sd_45_49, sd_45_491, CI = 0.025),
 result_upr_b_45_49 = whoilo_paf_summary(result_45_49, result_45_491, sd_45_49, sd_45_491, CI = 0.975),
 result_b_45_49 = whoilo_paf_summary(result_45_49, result_45_491, CI = NULL),
 result_lwr_b_50_54 = whoilo_paf_summary(result_50_54, result_50_541, sd_50_54, sd_50_541, CI = 0.025),
 result_upr_b_50_54 = whoilo_paf_summary(result_50_54, result_50_541, sd_50_54, sd_50_541, CI = 0.975),
 result_b_50_54 = whoilo_paf_summary(result_50_54, result_50_541, CI = NULL),
 result_lwr_b_55_59 = whoilo_paf_summary(result_55_59, result_55_591, sd_55_59, sd_55_591, CI = 0.025),
 result_upr_b_55_59 = whoilo_paf_summary(result_55_59, result_55_591, sd_55_59, sd_55_591, CI = 0.975),
 result_b_55_59 = whoilo_paf_summary(result_55_59, result_55_591, CI = NULL),
 result_lwr_b_60_64 = whoilo_paf_summary(result_60_64, result_60_641, sd_60_64, sd_60_641, CI = 0.025),
 result_upr_b_60_64 = whoilo_paf_summary(result_60_64, result_60_641, sd_60_64, sd_60_641, CI = 0.975),
 result_b_60_64 = whoilo_paf_summary(result_60_64, result_60_641, CI = NULL),
 result_lwr_b_65_69 = whoilo_paf_summary(result_65_69, result_65_691, sd_65_69, sd_65_691, CI = 0.025),
 result_upr_b_65_69 = whoilo_paf_summary(result_65_69, result_65_691, sd_65_69, sd_65_691, CI = 0.975),
 result_b_65_69 = whoilo_paf_summary(result_65_69, result_65_691, CI = NULL),
 result_lwr_b_70_74 = whoilo_paf_summary(result_70_74, result_70_741, sd_70_74, sd_70_741, CI = 0.025),
 result_upr_b_70_74 = whoilo_paf_summary(result_70_74, result_70_741, sd_70_74, sd_70_741, CI = 0.975),
 result_b_70_74 = whoilo_paf_summary(result_70_74, result_70_741, CI = NULL),
 result_lwr_b_75_79 = whoilo_paf_summary(result_75_79, result_75_791, sd_75_79, sd_75_791, CI = 0.025),
 result_upr_b_75_79 = whoilo_paf_summary(result_75_79, result_75_791, sd_75_79, sd_75_791, CI = 0.975),
 result_b_75_79 = whoilo_paf_summary(result_75_79, result_75_791, CI = NULL),
 result_lwr_b_80_84 = whoilo_paf_summary(result_80_84, result_80_841, sd_80_84, sd_80_841, CI = 0.025),
 result_upr_b_80_84 = whoilo_paf_summary(result_80_84, result_80_841, sd_80_84, sd_80_841, CI = 0.975),
 result_b_80_84 = whoilo_paf_summary(result_80_84, result_80_841, CI = NULL),
 result_lwr_b_85_89 = whoilo_paf_summary(result_85_89, result_85_891, sd_85_89, sd_85_891, CI = 0.025),
 result_upr_b_85_89 = whoilo_paf_summary(result_85_89, result_85_891, sd_85_89, sd_85_891, CI = 0.975),
 result_b_85_89 = whoilo_paf_summary(result_85_89, result_85_891, CI = NULL),
 result_lwr_b_90_94 = whoilo_paf_summary(result_90_94, result_90_941, sd_90_94, sd_90_941, CI = 0.025),
 result_upr_b_90_94 = whoilo_paf_summary(result_90_94, result_90_941, sd_90_94, sd_90_941, CI = 0.975),
 result_b_90_94 = whoilo_paf_summary(result_90_94, result_90_941, CI = NULL),
 result_lwr_b_95_ = whoilo_paf_summary(result_95_, result_95_1, sd_95_, sd_95_1, CI = 0.025),
 result_upr_b_95_ = whoilo_paf_summary(result_95_, result_95_1, sd_95_, sd_95_1, CI = 0.975),
 result_b_95_ = whoilo_paf_summary(result_95_, result_95_1, CI = NULL)) %>%
 ungroup() %>%
 mutate(sd_b_15_19 = (result_upr_b_15_19 - result_lwr_b_15_19)/2/1.96,
 sd_b_20_24 = (result_upr_b_20_24 - result_lwr_b_20_24)/2/1.96,
 sd_b_25_29 = (result_upr_b_25_29 - result_lwr_b_25_29)/2/1.96,
 sd_b_30_34 = (result_upr_b_30_34 - result_lwr_b_30_34)/2/1.96,
 sd_b_35_39 = (result_upr_b_35_39 - result_lwr_b_35_39)/2/1.96,
 sd_b_40_44 = (result_upr_b_40_44 - result_lwr_b_40_44)/2/1.96,
 sd_b_45_49 = (result_upr_b_45_49 - result_lwr_b_45_49)/2/1.96,
 sd_b_50_54 = (result_upr_b_50_54 - result_lwr_b_50_54)/2/1.96,
 sd_b_55_59 = (result_upr_b_55_59 - result_lwr_b_55_59)/2/1.96,
 sd_b_60_64 = (result_upr_b_60_64 - result_lwr_b_60_64)/2/1.96,
 sd_b_65_69 = (result_upr_b_65_69 - result_lwr_b_65_69)/2/1.96,
 sd_b_70_74 = (result_upr_b_70_74 - result_lwr_b_70_74)/2/1.96,
 sd_b_75_79 = (result_upr_b_75_79 - result_lwr_b_75_79)/2/1.96,
 sd_b_80_84 = (result_upr_b_80_84 - result_lwr_b_80_84)/2/1.96,
 sd_b_85_89 = (result_upr_b_85_89 - result_lwr_b_85_89)/2/1.96,
 sd_b_90_94 = (result_upr_b_90_94 - result_lwr_b_90_94)/2/1.96,
 sd_b_95_ = (result_upr_b_95_ - result_lwr_b_95_)/2/1.96) %>%
 select(region,
 result_b_15_19, sd_b_15_19, result_lwr_b_15_19, result_upr_b_15_19,
 result_b_20_24, sd_b_20_24, result_lwr_b_20_24, result_upr_b_20_24,
 result_b_25_29, sd_b_25_29, result_lwr_b_25_29, result_upr_b_25_29,
 result_b_30_34, sd_b_30_34, result_lwr_b_30_34, result_upr_b_30_34,
 result_b_35_39, sd_b_35_39, result_lwr_b_35_39, result_upr_b_35_39,
 result_b_40_44, sd_b_40_44, result_lwr_b_40_44, result_upr_b_40_44,
 result_b_45_49, sd_b_45_49, result_lwr_b_45_49, result_upr_b_45_49,
 result_b_50_54, sd_b_50_54, result_lwr_b_50_54, result_upr_b_50_54,
 result_b_55_59, sd_b_55_59, result_lwr_b_55_59, result_upr_b_55_59,
 result_b_60_64, sd_b_60_64, result_lwr_b_60_64, result_upr_b_60_64,
 result_b_65_69, sd_b_65_69, result_lwr_b_65_69, result_upr_b_65_69,
 result_b_70_74, sd_b_70_74, result_lwr_b_70_74, result_upr_b_70_74,
 result_b_75_79, sd_b_75_79, result_lwr_b_75_79, result_upr_b_75_79,
 result_b_80_84, sd_b_80_84, result_lwr_b_80_84, result_upr_b_80_84,
 result_b_85_89, sd_b_85_89, result_lwr_b_85_89, result_upr_b_85_89,
 result_b_90_94, sd_b_90_94, result_lwr_b_90_94, result_upr_b_90_94,
 result_b_95_, sd_b_95_, result_lwr_b_95_, result_upr_b_95_)
 }
 if (outcome %in% c("h0", "h1", "h2", "h3", "h4", "h5")){
 dths_stroke_age_global <- df_in %>%
 filter(outcome == !!outcome,
 year == !!year,
 cause == !!cause) %>%
 arrange(region, country_abbrev, year, sex, ilo_age_bands_5yr) %>%
 mutate(sd = (result_upr - result_lwr)/2/1.96) %>%
 group_by(region, ilo_age_bands_5yr) %>%
 left_join(who_ilo_long_populations, by = c("region", "country_abbrev", "year", "sex", "ilo_age_bands_5yr" = "age_bands_5yr")) %>%
 mutate(result = result*population,
 sd = sd*population) %>%
 drop_na() %>%
 summarise(result = sum(result),
 sd = sqrt(sum((sd)^2)),
 population = sum(population)) %>%
 mutate(result_lwr = qnorm(p = 0.025, mean = result, sd = sd)/ population,
 result_upr = qnorm(p = 0.975, mean = result, sd = sd)/ population,
 result = result / population,
 sd = sd/population) %>%
 select(-population) %>%
 ungroup %>%
 pivot_wider(names_from = ilo_age_bands_5yr, values_from = result:result_upr)
 names(dths_stroke_age_global) <- str_replace_all(names(dths_stroke_age_global), pattern = "result_", replacement = "result_b_")
 names(dths_stroke_age_global) <- str_replace_all(names(dths_stroke_age_global), pattern = "sd_", replacement = "sd_b_")
 names(dths_stroke_age_global) <- str_replace_all(names(dths_stroke_age_global), pattern = "result_b_lwr", replacement = "result_lwr_b")
 names(dths_stroke_age_global) <- str_replace_all(names(dths_stroke_age_global), pattern = "result_b_upr", replacement = "result_upr_b")
 dths_stroke_age_global <- dths_stroke_age_global %>%
 select(region,
 result_b_15_19, sd_b_15_19, result_lwr_b_15_19, result_upr_b_15_19,
 result_b_20_24, sd_b_20_24, result_lwr_b_20_24, result_upr_b_20_24,
 result_b_25_29, sd_b_25_29, result_lwr_b_25_29, result_upr_b_25_29,
 result_b_30_34, sd_b_30_34, result_lwr_b_30_34, result_upr_b_30_34,
 result_b_35_39, sd_b_35_39, result_lwr_b_35_39, result_upr_b_35_39,
 result_b_40_44, sd_b_40_44, result_lwr_b_40_44, result_upr_b_40_44,
 result_b_45_49, sd_b_45_49, result_lwr_b_45_49, result_upr_b_45_49,
 result_b_50_54, sd_b_50_54, result_lwr_b_50_54, result_upr_b_50_54,
 result_b_55_59, sd_b_55_59, result_lwr_b_55_59, result_upr_b_55_59,
 result_b_60_64, sd_b_60_64, result_lwr_b_60_64, result_upr_b_60_64,
 result_b_65_69, sd_b_65_69, result_lwr_b_65_69, result_upr_b_65_69,
 result_b_70_74, sd_b_70_74, result_lwr_b_70_74, result_upr_b_70_74,
 result_b_75_79, sd_b_75_79, result_lwr_b_75_79, result_upr_b_75_79,
 result_b_80_84, sd_b_80_84, result_lwr_b_80_84, result_upr_b_80_84,
 result_b_85_89, sd_b_85_89, result_lwr_b_85_89, result_upr_b_85_89,
 result_b_90_94, sd_b_90_94, result_lwr_b_90_94, result_upr_b_90_94,
 result_b_95_, sd_b_95_, result_lwr_b_95_, result_upr_b_95_)
 dths_stroke_age_global[dths_stroke_age_global < 0] <- 0
 }
 if (outcome %in% c("h0_number", "h1_number", "h2_number", "h3_number", "h4_number", "h5_number")){
 outcome2 <- str_sub(outcome, end = -8L)
 dths_stroke_age_global <- df_in %>%
 filter(outcome == !!outcome2,
 year == !!year,
 cause == !!cause) %>%
 arrange(region, country_abbrev, year, sex, ilo_age_bands_5yr) %>%
 mutate(sd = (result_upr - result_lwr)/2/1.96) %>%
 group_by(region, ilo_age_bands_5yr) %>%
 left_join(who_ilo_long_populations, by = c("region", "country_abbrev", "year", "sex", "ilo_age_bands_5yr" = "age_bands_5yr")) %>%
 mutate(population = population * 1000) %>%
 mutate(result = result*population,
 sd = sd*population) %>%
 drop_na() %>%
 summarise(result = sum(result),
 sd = sqrt(sum((sd)^2)),
 population = sum(population)) %>%
 mutate(result_lwr = qnorm(p = 0.025, mean = result, sd = sd),
 result_upr = qnorm(p = 0.975, mean = result, sd = sd),
 result = result,
 sd = sd) %>%
 select(-population) %>%
 ungroup %>%
 pivot_wider(names_from = ilo_age_bands_5yr, values_from = result:result_upr)
 names(dths_stroke_age_global) <- str_replace_all(names(dths_stroke_age_global), pattern = "result_", replacement = "result_b_")
 names(dths_stroke_age_global) <- str_replace_all(names(dths_stroke_age_global), pattern = "sd_", replacement = "sd_b_")
 names(dths_stroke_age_global) <- str_replace_all(names(dths_stroke_age_global), pattern = "result_b_lwr", replacement = "result_lwr_b")
 names(dths_stroke_age_global) <- str_replace_all(names(dths_stroke_age_global), pattern = "result_b_upr", replacement = "result_upr_b")
 dths_stroke_age_global <- dths_stroke_age_global %>%
 select(region,
 result_b_15_19, sd_b_15_19, result_lwr_b_15_19, result_upr_b_15_19,
 result_b_20_24, sd_b_20_24, result_lwr_b_20_24, result_upr_b_20_24,
 result_b_25_29, sd_b_25_29, result_lwr_b_25_29, result_upr_b_25_29,
 result_b_30_34, sd_b_30_34, result_lwr_b_30_34, result_upr_b_30_34,
 result_b_35_39, sd_b_35_39, result_lwr_b_35_39, result_upr_b_35_39,
 result_b_40_44, sd_b_40_44, result_lwr_b_40_44, result_upr_b_40_44,
 result_b_45_49, sd_b_45_49, result_lwr_b_45_49, result_upr_b_45_49,
 result_b_50_54, sd_b_50_54, result_lwr_b_50_54, result_upr_b_50_54,
 result_b_55_59, sd_b_55_59, result_lwr_b_55_59, result_upr_b_55_59,
 result_b_60_64, sd_b_60_64, result_lwr_b_60_64, result_upr_b_60_64,
 result_b_65_69, sd_b_65_69, result_lwr_b_65_69, result_upr_b_65_69,
 result_b_70_74, sd_b_70_74, result_lwr_b_70_74, result_upr_b_70_74,
 result_b_75_79, sd_b_75_79, result_lwr_b_75_79, result_upr_b_75_79,
 result_b_80_84, sd_b_80_84, result_lwr_b_80_84, result_upr_b_80_84,
 result_b_85_89, sd_b_85_89, result_lwr_b_85_89, result_upr_b_85_89,
 result_b_90_94, sd_b_90_94, result_lwr_b_90_94, result_upr_b_90_94,
 result_b_95_, sd_b_95_, result_lwr_b_95_, result_upr_b_95_)
 dths_stroke_age_global[dths_stroke_age_global < 0] <- 0
 }
 if (outcome %in% c("DTH", "DALY", "DTHenvelope", "DALYenvelope")) {
 dths_stroke_age_global <- df_in %>%
 filter(outcome == !!outcome,
 year == !!year,
 cause == !!cause) %>%
 arrange(region, country_abbrev, year, sex, ilo_age_bands_5yr) %>%
 mutate(sd = (result_upr - result_lwr)/2/1.96) %>%
 drop_na(region, result) %>%
 group_by(region, ilo_age_bands_5yr) %>%
 summarise(result = sum(result),
 sd = sqrt(sum((sd)^2))) %>%
 mutate(result_lwr = qnorm(p = 0.025, mean = result, sd = sd),
 result_upr = qnorm(p = 0.975, mean = result, sd = sd)) %>%
 ungroup %>%
 pivot_wider(names_from = ilo_age_bands_5yr, values_from = result:result_upr)
 names(dths_stroke_age_global) <- str_replace_all(names(dths_stroke_age_global), pattern = "result_", replacement = "result_b_")
 names(dths_stroke_age_global) <- str_replace_all(names(dths_stroke_age_global), pattern = "sd_", replacement = "sd_b_")
 names(dths_stroke_age_global) <- str_replace_all(names(dths_stroke_age_global), pattern = "result_b_lwr", replacement = "result_lwr_b")
 names(dths_stroke_age_global) <- str_replace_all(names(dths_stroke_age_global), pattern = "result_b_upr", replacement = "result_upr_b")
 dths_stroke_age_global <- dths_stroke_age_global %>%
 select(region,
 result_b_15_19, sd_b_15_19, result_lwr_b_15_19, result_upr_b_15_19,
 result_b_20_24, sd_b_20_24, result_lwr_b_20_24, result_upr_b_20_24,
 result_b_25_29, sd_b_25_29, result_lwr_b_25_29, result_upr_b_25_29,
 result_b_30_34, sd_b_30_34, result_lwr_b_30_34, result_upr_b_30_34,
 result_b_35_39, sd_b_35_39, result_lwr_b_35_39, result_upr_b_35_39,
 result_b_40_44, sd_b_40_44, result_lwr_b_40_44, result_upr_b_40_44,
 result_b_45_49, sd_b_45_49, result_lwr_b_45_49, result_upr_b_45_49,
 result_b_50_54, sd_b_50_54, result_lwr_b_50_54, result_upr_b_50_54,
 result_b_55_59, sd_b_55_59, result_lwr_b_55_59, result_upr_b_55_59,
 result_b_60_64, sd_b_60_64, result_lwr_b_60_64, result_upr_b_60_64,
 result_b_65_69, sd_b_65_69, result_lwr_b_65_69, result_upr_b_65_69,
 result_b_70_74, sd_b_70_74, result_lwr_b_70_74, result_upr_b_70_74,
 result_b_75_79, sd_b_75_79, result_lwr_b_75_79, result_upr_b_75_79,
 result_b_80_84, sd_b_80_84, result_lwr_b_80_84, result_upr_b_80_84,
 result_b_85_89, sd_b_85_89, result_lwr_b_85_89, result_upr_b_85_89,
 result_b_90_94, sd_b_90_94, result_lwr_b_90_94, result_upr_b_90_94,
 result_b_95_, sd_b_95_, result_lwr_b_95_, result_upr_b_95_)
 }
 if (outcome %in% c("DTHS", "DALYS")) {
 outcome2 <- str_sub(outcome, end = -2L)
 dths_stroke_age_global <- df_in %>%
 filter(outcome == !!outcome2,
 year == !!year,
 cause %in% c("stroke_h5", "ihd_h5")) %>%
 arrange(region, country_abbrev, year, sex, ilo_age_bands_5yr) %>%
 mutate(sd = (result_upr - result_lwr)/2/1.96) %>%
 drop_na() %>%
 group_by(region, ilo_age_bands_5yr) %>%
 summarise(result = sum(result),
 sd = sqrt(sum((sd)^2))) %>%
 mutate(result_lwr = qnorm(p = 0.025, mean = result, sd = sd),
 result_upr = qnorm(p = 0.975, mean = result, sd = sd)) %>%
 ungroup %>%
 pivot_wider(names_from = ilo_age_bands_5yr, values_from = result:result_upr)
 names(dths_stroke_age_global) <- str_replace_all(names(dths_stroke_age_global), pattern = "result_", replacement = "result_b_")
 names(dths_stroke_age_global) <- str_replace_all(names(dths_stroke_age_global), pattern = "sd_", replacement = "sd_b_")
 names(dths_stroke_age_global) <- str_replace_all(names(dths_stroke_age_global), pattern = "result_b_lwr", replacement = "result_lwr_b")
 names(dths_stroke_age_global) <- str_replace_all(names(dths_stroke_age_global), pattern = "result_b_upr", replacement = "result_upr_b")
 dths_stroke_age_global <- dths_stroke_age_global %>%
 select(region, result_b_15_19, sd_b_15_19, result_lwr_b_15_19, result_upr_b_15_19,
 result_b_20_24, sd_b_20_24, result_lwr_b_20_24, result_upr_b_20_24,
 result_b_25_29, sd_b_25_29, result_lwr_b_25_29, result_upr_b_25_29,
 result_b_30_34, sd_b_30_34, result_lwr_b_30_34, result_upr_b_30_34,
 result_b_35_39, sd_b_35_39, result_lwr_b_35_39, result_upr_b_35_39,
 result_b_40_44, sd_b_40_44, result_lwr_b_40_44, result_upr_b_40_44,
 result_b_45_49, sd_b_45_49, result_lwr_b_45_49, result_upr_b_45_49,
 result_b_50_54, sd_b_50_54, result_lwr_b_50_54, result_upr_b_50_54,
 result_b_55_59, sd_b_55_59, result_lwr_b_55_59, result_upr_b_55_59,
 result_b_60_64, sd_b_60_64, result_lwr_b_60_64, result_upr_b_60_64,
 result_b_65_69, sd_b_65_69, result_lwr_b_65_69, result_upr_b_65_69,
 result_b_70_74, sd_b_70_74, result_lwr_b_70_74, result_upr_b_70_74,
 result_b_75_79, sd_b_75_79, result_lwr_b_75_79, result_upr_b_75_79,
 result_b_80_84, sd_b_80_84, result_lwr_b_80_84, result_upr_b_80_84,
 result_b_85_89, sd_b_85_89, result_lwr_b_85_89, result_upr_b_85_89,
 result_b_90_94, sd_b_90_94, result_lwr_b_90_94, result_upr_b_90_94,
 result_b_95_, sd_b_95_, result_lwr_b_95_, result_upr_b_95_)
 }
 if (outcome %in% c("h45")){
 dths_stroke_age_global <- df_in %>%
 filter(outcome %in% c("h4", "h5"),
 year == !!year,
 cause == !!cause) %>%
 arrange(region, country_abbrev, year, sex, ilo_age_bands_5yr) %>%
 mutate(sd = (result_upr - result_lwr)/2/1.96) %>%
 group_by(region, ilo_age_bands_5yr) %>%
 left_join(who_ilo_long_populations, by = c("region", "country_abbrev", "year", "sex", "ilo_age_bands_5yr" = "age_bands_5yr")) %>%
 mutate(result = result*population,
 sd = sd*population) %>%
 drop_na() %>%
 summarise(result = sum(result),
 sd = sqrt(sum((sd)^2)),
 population = sum(population)/2) %>%
 mutate(result_lwr = qnorm(p = 0.025, mean = result, sd = sd)/ population,
 result_upr = qnorm(p = 0.975, mean = result, sd = sd)/ population,
 result = result / population,
 sd = sd/population) %>%
 select(-population) %>%
 ungroup %>%
 pivot_wider(names_from = ilo_age_bands_5yr, values_from = result:result_upr)
 names(dths_stroke_age_global) <- str_replace_all(names(dths_stroke_age_global), pattern = "result_", replacement = "result_b_")
 names(dths_stroke_age_global) <- str_replace_all(names(dths_stroke_age_global), pattern = "sd_", replacement = "sd_b_")
 names(dths_stroke_age_global) <- str_replace_all(names(dths_stroke_age_global), pattern = "result_b_lwr", replacement = "result_lwr_b")
 names(dths_stroke_age_global) <- str_replace_all(names(dths_stroke_age_global), pattern = "result_b_upr", replacement = "result_upr_b")
 dths_stroke_age_global <- dths_stroke_age_global %>%
 select(region, result_b_15_19, sd_b_15_19, result_lwr_b_15_19, result_upr_b_15_19,
 result_b_20_24, sd_b_20_24, result_lwr_b_20_24, result_upr_b_20_24,
 result_b_25_29, sd_b_25_29, result_lwr_b_25_29, result_upr_b_25_29,
 result_b_30_34, sd_b_30_34, result_lwr_b_30_34, result_upr_b_30_34,
 result_b_35_39, sd_b_35_39, result_lwr_b_35_39, result_upr_b_35_39,
 result_b_40_44, sd_b_40_44, result_lwr_b_40_44, result_upr_b_40_44,
 result_b_45_49, sd_b_45_49, result_lwr_b_45_49, result_upr_b_45_49,
 result_b_50_54, sd_b_50_54, result_lwr_b_50_54, result_upr_b_50_54,
 result_b_55_59, sd_b_55_59, result_lwr_b_55_59, result_upr_b_55_59,
 result_b_60_64, sd_b_60_64, result_lwr_b_60_64, result_upr_b_60_64,
 result_b_65_69, sd_b_65_69, result_lwr_b_65_69, result_upr_b_65_69,
 result_b_70_74, sd_b_70_74, result_lwr_b_70_74, result_upr_b_70_74,
 result_b_75_79, sd_b_75_79, result_lwr_b_75_79, result_upr_b_75_79,
 result_b_80_84, sd_b_80_84, result_lwr_b_80_84, result_upr_b_80_84,
 result_b_85_89, sd_b_85_89, result_lwr_b_85_89, result_upr_b_85_89,
 result_b_90_94, sd_b_90_94, result_lwr_b_90_94, result_upr_b_90_94,
 result_b_95_, sd_b_95_, result_lwr_b_95_, result_upr_b_95_)
 dths_stroke_age_global[dths_stroke_age_global < 0] <- 0
 }
 if (outcome %in% c("DTHrate")){
 outcome2 <- case_when(outcome == "DTHrate" ~ "DTH",
 outcome == "DALYrate" ~ "DALY",
 TRUE ~ NA_character_)
 dths_stroke_age_global <- df_in %>%
 filter(outcome == !!outcome2,
 year == !!year,
 cause == !!cause) %>%
 arrange(region, country_abbrev, year, sex, ilo_age_bands_5yr) %>%
 mutate(sd = (result_upr - result_lwr)/2/1.96) %>%
 group_by(region, ilo_age_bands_5yr) %>%
 left_join(who_ilo_long_populations, by = c("region", "country_abbrev", "year", "sex", "ilo_age_bands_5yr" = "age_bands_5yr")) %>%
 drop_na() %>%
 summarise(result = sum(result),
 sd = sqrt(sum((sd)^2)),
 population = sum(population)) %>%
 mutate(population = population / 100,
 result = result / population,
 sd = sd / population,
 result_lwr = qnorm(p = 0.025, mean = result, sd = sd),
 result_upr = qnorm(p = 0.975, mean = result, sd = sd)) %>%
 select(-population) %>%
 ungroup %>%
 pivot_wider(names_from = ilo_age_bands_5yr, values_from = result:result_upr)
 names(dths_stroke_age_global) <- str_replace_all(names(dths_stroke_age_global), pattern = "result_", replacement = "result_b_")
 names(dths_stroke_age_global) <- str_replace_all(names(dths_stroke_age_global), pattern = "sd_", replacement = "sd_b_")
 names(dths_stroke_age_global) <- str_replace_all(names(dths_stroke_age_global), pattern = "result_b_lwr", replacement = "result_lwr_b")
 names(dths_stroke_age_global) <- str_replace_all(names(dths_stroke_age_global), pattern = "result_b_upr", replacement = "result_upr_b")
 dths_stroke_age_global <- dths_stroke_age_global %>%
 select(region,
 result_b_15_19, sd_b_15_19, result_lwr_b_15_19, result_upr_b_15_19,
 result_b_20_24, sd_b_20_24, result_lwr_b_20_24, result_upr_b_20_24,
 result_b_25_29, sd_b_25_29, result_lwr_b_25_29, result_upr_b_25_29,
 result_b_30_34, sd_b_30_34, result_lwr_b_30_34, result_upr_b_30_34,
 result_b_35_39, sd_b_35_39, result_lwr_b_35_39, result_upr_b_35_39,
 result_b_40_44, sd_b_40_44, result_lwr_b_40_44, result_upr_b_40_44,
 result_b_45_49, sd_b_45_49, result_lwr_b_45_49, result_upr_b_45_49,
 result_b_50_54, sd_b_50_54, result_lwr_b_50_54, result_upr_b_50_54,
 result_b_55_59, sd_b_55_59, result_lwr_b_55_59, result_upr_b_55_59,
 result_b_60_64, sd_b_60_64, result_lwr_b_60_64, result_upr_b_60_64,
 result_b_65_69, sd_b_65_69, result_lwr_b_65_69, result_upr_b_65_69,
 result_b_70_74, sd_b_70_74, result_lwr_b_70_74, result_upr_b_70_74,
 result_b_75_79, sd_b_75_79, result_lwr_b_75_79, result_upr_b_75_79,
 result_b_80_84, sd_b_80_84, result_lwr_b_80_84, result_upr_b_80_84,
 result_b_85_89, sd_b_85_89, result_lwr_b_85_89, result_upr_b_85_89,
 result_b_90_94, sd_b_90_94, result_lwr_b_90_94, result_upr_b_90_94,
 result_b_95_, sd_b_95_, result_lwr_b_95_, result_upr_b_95_)
 }
 if (outcome %in% c("DALYrate")){
 outcome2 <- case_when(outcome == "DTHrate" ~ "DTH",
 outcome == "DALYrate" ~ "DALY",
 TRUE ~ NA_character_)
 dths_stroke_age_global <- df_in %>%
 filter(outcome == !!outcome2,
 year == !!year,
 cause == !!cause) %>%
 arrange(region, country_abbrev, year, sex, ilo_age_bands_5yr) %>%
 mutate(sd = (result_upr - result_lwr)/2/1.96) %>%
 group_by(region, ilo_age_bands_5yr) %>%
 left_join(who_ilo_long_populations, by = c("region", "country_abbrev", "year", "sex", "ilo_age_bands_5yr" = "age_bands_5yr")) %>%
 drop_na() %>%
 summarise(result = sum(result),
 sd = sqrt(sum((sd)^2)),
 population = sum(population)) %>%
 mutate(population = population,
 result = result / population * 100000,
 sd = sd / population * 100000,
 result_lwr = qnorm(p = 0.025, mean = result, sd = sd),
 result_upr = qnorm(p = 0.975, mean = result, sd = sd)) %>%
 select(-population) %>%
 ungroup %>%
 pivot_wider(names_from = ilo_age_bands_5yr, values_from = result:result_upr)
 names(dths_stroke_age_global) <- str_replace_all(names(dths_stroke_age_global), pattern = "result_", replacement = "result_b_")
 names(dths_stroke_age_global) <- str_replace_all(names(dths_stroke_age_global), pattern = "sd_", replacement = "sd_b_")
 names(dths_stroke_age_global) <- str_replace_all(names(dths_stroke_age_global), pattern = "result_b_lwr", replacement = "result_lwr_b")
 names(dths_stroke_age_global) <- str_replace_all(names(dths_stroke_age_global), pattern = "result_b_upr", replacement = "result_upr_b")
 dths_stroke_age_global <- dths_stroke_age_global %>%
 select(region,
 result_b_15_19, sd_b_15_19, result_lwr_b_15_19, result_upr_b_15_19,
 result_b_20_24, sd_b_20_24, result_lwr_b_20_24, result_upr_b_20_24,
 result_b_25_29, sd_b_25_29, result_lwr_b_25_29, result_upr_b_25_29,
 result_b_30_34, sd_b_30_34, result_lwr_b_30_34, result_upr_b_30_34,
 result_b_35_39, sd_b_35_39, result_lwr_b_35_39, result_upr_b_35_39,
 result_b_40_44, sd_b_40_44, result_lwr_b_40_44, result_upr_b_40_44,
 result_b_45_49, sd_b_45_49, result_lwr_b_45_49, result_upr_b_45_49,
 result_b_50_54, sd_b_50_54, result_lwr_b_50_54, result_upr_b_50_54,
 result_b_55_59, sd_b_55_59, result_lwr_b_55_59, result_upr_b_55_59,
 result_b_60_64, sd_b_60_64, result_lwr_b_60_64, result_upr_b_60_64,
 result_b_65_69, sd_b_65_69, result_lwr_b_65_69, result_upr_b_65_69,
 result_b_70_74, sd_b_70_74, result_lwr_b_70_74, result_upr_b_70_74,
 result_b_75_79, sd_b_75_79, result_lwr_b_75_79, result_upr_b_75_79,
 result_b_80_84, sd_b_80_84, result_lwr_b_80_84, result_upr_b_80_84,
 result_b_85_89, sd_b_85_89, result_lwr_b_85_89, result_upr_b_85_89,
 result_b_90_94, sd_b_90_94, result_lwr_b_90_94, result_upr_b_90_94,
 result_b_95_, sd_b_95_, result_lwr_b_95_, result_upr_b_95_)
 }
 if (outcome %in% c("population")){
 dths_stroke_age_global <- who_ilo_long_populations %>%
 filter(age_bands_5yr %in% c("15_19", "20_24", "25_29", "30_34", "35_39", "40_44", "45_49", "50_54", "55_59", "60_64", "65_69", "70_74", "75_79", "80_84", "85_89", "90_94", "95_"),
 year == !!year) %>%
 arrange(region, country_abbrev, year, sex, age_bands_5yr) %>%
 drop_na() %>%
 group_by(region, age_bands_5yr) %>%
 summarise(result_b = sum(population)) %>%
 mutate(sd_b = NA,
 result_lwr_b = NA,
 result_upr_b = NA) %>%
 ungroup %>%
 pivot_wider(names_from = age_bands_5yr, values_from = result_b:result_upr_b) %>%
 select(region,
 result_b_15_19, sd_b_15_19, result_lwr_b_15_19, result_upr_b_15_19,
 result_b_20_24, sd_b_20_24, result_lwr_b_20_24, result_upr_b_20_24,
 result_b_25_29, sd_b_25_29, result_lwr_b_25_29, result_upr_b_25_29,
 result_b_30_34, sd_b_30_34, result_lwr_b_30_34, result_upr_b_30_34,
 result_b_35_39, sd_b_35_39, result_lwr_b_35_39, result_upr_b_35_39,
 result_b_40_44, sd_b_40_44, result_lwr_b_40_44, result_upr_b_40_44,
 result_b_45_49, sd_b_45_49, result_lwr_b_45_49, result_upr_b_45_49,
 result_b_50_54, sd_b_50_54, result_lwr_b_50_54, result_upr_b_50_54,
 result_b_55_59, sd_b_55_59, result_lwr_b_55_59, result_upr_b_55_59,
 result_b_60_64, sd_b_60_64, result_lwr_b_60_64, result_upr_b_60_64,
 result_b_65_69, sd_b_65_69, result_lwr_b_65_69, result_upr_b_65_69,
 result_b_70_74, sd_b_70_74, result_lwr_b_70_74, result_upr_b_70_74,
 result_b_75_79, sd_b_75_79, result_lwr_b_75_79, result_upr_b_75_79,
 result_b_80_84, sd_b_80_84, result_lwr_b_80_84, result_upr_b_80_84,
 result_b_85_89, sd_b_85_89, result_lwr_b_85_89, result_upr_b_85_89,
 result_b_90_94, sd_b_90_94, result_lwr_b_90_94, result_upr_b_90_94,
 result_b_95_, sd_b_95_, result_lwr_b_95_, result_upr_b_95_)
 }
 if (outcome %in% c("DTHratio")){
 envelope_stroke_age_global <- df_in %>%
 filter(outcome == "DTHparent",
 year == !!year,
 cause == !!cause) %>%
 arrange(region, country_abbrev, year, sex, ilo_age_bands_5yr) %>%
 drop_na() %>%
 mutate(sd = (result_upr - result_lwr)/2/1.96) %>%
 group_by(region, ilo_age_bands_5yr) %>%
 summarise(result = sum(result),
 sd = sqrt(sum((sd)^2))) %>%
 ungroup %>%
 pivot_wider(names_from = ilo_age_bands_5yr, values_from = result:sd)

 dths_stroke_age_global <- df_in %>%
 filter(outcome == "DTHenvelope",
 year == !!year,
 cause == !!cause) %>%
 arrange(region, country_abbrev, year, sex, ilo_age_bands_5yr) %>%
 drop_na() %>%
 mutate(sd = (result_upr - result_lwr)/2/1.96) %>%
 group_by(region, ilo_age_bands_5yr) %>%
 summarise(result = sum(result),
 sd = sqrt(sum((sd)^2))) %>%
 ungroup %>%
 pivot_wider(names_from = ilo_age_bands_5yr, values_from = result:sd) %>%

 select_all(.funs = funs(str_c(., "1")))

 dths_stroke_age_global <- bind_cols(dths_stroke_age_global, envelope_stroke_age_global) %>%
 rowwise() %>%
 transmute(region = region,
 result_lwr_b_15_19 = whoilo_paf_summary(result_15_19, result_15_191, sd_15_19, sd_15_191, CI = 0.025),
 result_upr_b_15_19 = whoilo_paf_summary(result_15_19, result_15_191, sd_15_19, sd_15_191, CI = 0.975),
 result_b_15_19 = whoilo_paf_summary(result_15_19, result_15_191, CI = NULL),
 result_lwr_b_20_24 = whoilo_paf_summary(result_20_24, result_20_241, sd_20_24, sd_20_241, CI = 0.025),
 result_upr_b_20_24 = whoilo_paf_summary(result_20_24, result_20_241, sd_20_24, sd_20_241, CI = 0.975),
 result_b_20_24 = whoilo_paf_summary(result_20_24, result_20_241, CI = NULL),
 result_lwr_b_25_29 = whoilo_paf_summary(result_25_29, result_25_291, sd_25_29, sd_25_291, CI = 0.025),
 result_upr_b_25_29 = whoilo_paf_summary(result_25_29, result_25_291, sd_25_29, sd_25_291, CI = 0.975),
 result_b_25_29 = whoilo_paf_summary(result_25_29, result_25_291, CI = NULL),
 result_lwr_b_30_34 = whoilo_paf_summary(result_30_34, result_30_341, sd_30_34, sd_30_341, CI = 0.025),
 result_upr_b_30_34 = whoilo_paf_summary(result_30_34, result_30_341, sd_30_34, sd_30_341, CI = 0.975),
 result_b_30_34 = whoilo_paf_summary(result_30_34, result_30_341, CI = NULL),
 result_lwr_b_35_39 = whoilo_paf_summary(result_35_39, result_35_391, sd_35_39, sd_35_391, CI = 0.025),
 result_upr_b_35_39 = whoilo_paf_summary(result_35_39, result_35_391, sd_35_39, sd_35_391, CI = 0.975),
 result_b_35_39 = whoilo_paf_summary(result_35_39, result_35_391, CI = NULL),
 result_lwr_b_40_44 = whoilo_paf_summary(result_40_44, result_40_441, sd_40_44, sd_40_441, CI = 0.025),
 result_upr_b_40_44 = whoilo_paf_summary(result_40_44, result_40_441, sd_40_44, sd_40_441, CI = 0.975),
 result_b_40_44 = whoilo_paf_summary(result_40_44, result_40_441, CI = NULL),
 result_lwr_b_45_49 = whoilo_paf_summary(result_45_49, result_45_491, sd_45_49, sd_45_491, CI = 0.025),
 result_upr_b_45_49 = whoilo_paf_summary(result_45_49, result_45_491, sd_45_49, sd_45_491, CI = 0.975),
 result_b_45_49 = whoilo_paf_summary(result_45_49, result_45_491, CI = NULL),
 result_lwr_b_50_54 = whoilo_paf_summary(result_50_54, result_50_541, sd_50_54, sd_50_541, CI = 0.025),
 result_upr_b_50_54 = whoilo_paf_summary(result_50_54, result_50_541, sd_50_54, sd_50_541, CI = 0.975),
 result_b_50_54 = whoilo_paf_summary(result_50_54, result_50_541, CI = NULL),
 result_lwr_b_55_59 = whoilo_paf_summary(result_55_59, result_55_591, sd_55_59, sd_55_591, CI = 0.025),
 result_upr_b_55_59 = whoilo_paf_summary(result_55_59, result_55_591, sd_55_59, sd_55_591, CI = 0.975),
 result_b_55_59 = whoilo_paf_summary(result_55_59, result_55_591, CI = NULL),
 result_lwr_b_60_64 = whoilo_paf_summary(result_60_64, result_60_641, sd_60_64, sd_60_641, CI = 0.025),
 result_upr_b_60_64 = whoilo_paf_summary(result_60_64, result_60_641, sd_60_64, sd_60_641, CI = 0.975),
 result_b_60_64 = whoilo_paf_summary(result_60_64, result_60_641, CI = NULL),
 result_lwr_b_65_69 = whoilo_paf_summary(result_65_69, result_65_691, sd_65_69, sd_65_691, CI = 0.025),
 result_upr_b_65_69 = whoilo_paf_summary(result_65_69, result_65_691, sd_65_69, sd_65_691, CI = 0.975),
 result_b_65_69 = whoilo_paf_summary(result_65_69, result_65_691, CI = NULL),
 result_lwr_b_70_74 = whoilo_paf_summary(result_70_74, result_70_741, sd_70_74, sd_70_741, CI = 0.025),
 result_upr_b_70_74 = whoilo_paf_summary(result_70_74, result_70_741, sd_70_74, sd_70_741, CI = 0.975),
 result_b_70_74 = whoilo_paf_summary(result_70_74, result_70_741, CI = NULL),
 result_lwr_b_75_79 = whoilo_paf_summary(result_75_79, result_75_791, sd_75_79, sd_75_791, CI = 0.025),
 result_upr_b_75_79 = whoilo_paf_summary(result_75_79, result_75_791, sd_75_79, sd_75_791, CI = 0.975),
 result_b_75_79 = whoilo_paf_summary(result_75_79, result_75_791, CI = NULL),
 result_lwr_b_80_84 = whoilo_paf_summary(result_80_84, result_80_841, sd_80_84, sd_80_841, CI = 0.025),
 result_upr_b_80_84 = whoilo_paf_summary(result_80_84, result_80_841, sd_80_84, sd_80_841, CI = 0.975),
 result_b_80_84 = whoilo_paf_summary(result_80_84, result_80_841, CI = NULL),
 result_lwr_b_85_89 = whoilo_paf_summary(result_85_89, result_85_891, sd_85_89, sd_85_891, CI = 0.025),
 result_upr_b_85_89 = whoilo_paf_summary(result_85_89, result_85_891, sd_85_89, sd_85_891, CI = 0.975),
 result_b_85_89 = whoilo_paf_summary(result_85_89, result_85_891, CI = NULL),
 result_lwr_b_90_94 = whoilo_paf_summary(result_90_94, result_90_941, sd_90_94, sd_90_941, CI = 0.025),
 result_upr_b_90_94 = whoilo_paf_summary(result_90_94, result_90_941, sd_90_94, sd_90_941, CI = 0.975),
 result_b_90_94 = whoilo_paf_summary(result_90_94, result_90_941, CI = NULL),
 result_lwr_b_95_ = whoilo_paf_summary(result_95_, result_95_1, sd_95_, sd_95_1, CI = 0.025),
 result_upr_b_95_ = whoilo_paf_summary(result_95_, result_95_1, sd_95_, sd_95_1, CI = 0.975),
 result_b_95_ = whoilo_paf_summary(result_95_, result_95_1, CI = NULL)) %>%
 ungroup() %>%
 mutate(sd_b_15_19 = (result_upr_b_15_19 - result_lwr_b_15_19)/2/1.96,
 sd_b_20_24 = (result_upr_b_20_24 - result_lwr_b_20_24)/2/1.96,
 sd_b_25_29 = (result_upr_b_25_29 - result_lwr_b_25_29)/2/1.96,
 sd_b_30_34 = (result_upr_b_30_34 - result_lwr_b_30_34)/2/1.96,
 sd_b_35_39 = (result_upr_b_35_39 - result_lwr_b_35_39)/2/1.96,
 sd_b_40_44 = (result_upr_b_40_44 - result_lwr_b_40_44)/2/1.96,
 sd_b_45_49 = (result_upr_b_45_49 - result_lwr_b_45_49)/2/1.96,
 sd_b_50_54 = (result_upr_b_50_54 - result_lwr_b_50_54)/2/1.96,
 sd_b_55_59 = (result_upr_b_55_59 - result_lwr_b_55_59)/2/1.96,
 sd_b_60_64 = (result_upr_b_60_64 - result_lwr_b_60_64)/2/1.96,
 sd_b_65_69 = (result_upr_b_65_69 - result_lwr_b_65_69)/2/1.96,
 sd_b_70_74 = (result_upr_b_70_74 - result_lwr_b_70_74)/2/1.96,
 sd_b_75_79 = (result_upr_b_75_79 - result_lwr_b_75_79)/2/1.96,
 sd_b_80_84 = (result_upr_b_80_84 - result_lwr_b_80_84)/2/1.96,
 sd_b_85_89 = (result_upr_b_85_89 - result_lwr_b_85_89)/2/1.96,
 sd_b_90_94 = (result_upr_b_90_94 - result_lwr_b_90_94)/2/1.96,
 sd_b_95_ = (result_upr_b_95_ - result_lwr_b_95_)/2/1.96) %>%
 select(region,
 result_b_15_19, sd_b_15_19, result_lwr_b_15_19, result_upr_b_15_19,
 result_b_20_24, sd_b_20_24, result_lwr_b_20_24, result_upr_b_20_24,
 result_b_25_29, sd_b_25_29, result_lwr_b_25_29, result_upr_b_25_29,
 result_b_30_34, sd_b_30_34, result_lwr_b_30_34, result_upr_b_30_34,
 result_b_35_39, sd_b_35_39, result_lwr_b_35_39, result_upr_b_35_39,
 result_b_40_44, sd_b_40_44, result_lwr_b_40_44, result_upr_b_40_44,
 result_b_45_49, sd_b_45_49, result_lwr_b_45_49, result_upr_b_45_49,
 result_b_50_54, sd_b_50_54, result_lwr_b_50_54, result_upr_b_50_54,
 result_b_55_59, sd_b_55_59, result_lwr_b_55_59, result_upr_b_55_59,
 result_b_60_64, sd_b_60_64, result_lwr_b_60_64, result_upr_b_60_64,
 result_b_65_69, sd_b_65_69, result_lwr_b_65_69, result_upr_b_65_69,
 result_b_70_74, sd_b_70_74, result_lwr_b_70_74, result_upr_b_70_74,
 result_b_75_79, sd_b_75_79, result_lwr_b_75_79, result_upr_b_75_79,
 result_b_80_84, sd_b_80_84, result_lwr_b_80_84, result_upr_b_80_84,
 result_b_85_89, sd_b_85_89, result_lwr_b_85_89, result_upr_b_85_89,
 result_b_90_94, sd_b_90_94, result_lwr_b_90_94, result_upr_b_90_94,
 result_b_95_, sd_b_95_, result_lwr_b_95_, result_upr_b_95_)
 }
 if (outcome %in% c("DALYratio")){
 envelope_stroke_age_global <- df_in %>%
 filter(outcome == "DALYparent",
 year == !!year,
 cause == !!cause) %>%
 arrange(region, country_abbrev, year, sex, ilo_age_bands_5yr) %>%
 drop_na() %>%
 mutate(sd = (result_upr - result_lwr)/2/1.96) %>%
 group_by(region, ilo_age_bands_5yr) %>%
 summarise(result = sum(result),
 sd = sqrt(sum((sd)^2))) %>%
 ungroup %>%
 pivot_wider(names_from = ilo_age_bands_5yr, values_from = result:sd)

 dths_stroke_age_global <- df_in %>%
 filter(outcome == "DALYenvelope",
 year == !!year,
 cause == !!cause) %>%
 mutate(result_lwr = case_when(is.na(result_lwr) ~ result,
 TRUE ~ result_lwr),
 result_upr = case_when(is.na(result_upr) ~ result,
 TRUE ~ result_upr)) %>%
 arrange(region, country_abbrev, year, sex, ilo_age_bands_5yr) %>%
 drop_na() %>%
 mutate(sd = (result_upr - result_lwr)/2/1.96) %>%
 group_by(region, ilo_age_bands_5yr) %>%
 summarise(result = sum(result),
 sd = sqrt(sum((sd)^2))) %>%
 ungroup %>%
 pivot_wider(names_from = ilo_age_bands_5yr, values_from = result:sd) %>%

 select_all(.funs = funs(str_c(., "1")))

 dths_stroke_age_global <- bind_cols(dths_stroke_age_global, envelope_stroke_age_global) %>%
 rowwise() %>%
 transmute(region = region,
 result_lwr_b_15_19 = whoilo_paf_summary(result_15_19, result_15_191, sd_15_19, sd_15_191, CI = 0.025),
 result_upr_b_15_19 = whoilo_paf_summary(result_15_19, result_15_191, sd_15_19, sd_15_191, CI = 0.975),
 result_b_15_19 = whoilo_paf_summary(result_15_19, result_15_191, CI = NULL),
 result_lwr_b_20_24 = whoilo_paf_summary(result_20_24, result_20_241, sd_20_24, sd_20_241, CI = 0.025),
 result_upr_b_20_24 = whoilo_paf_summary(result_20_24, result_20_241, sd_20_24, sd_20_241, CI = 0.975),
 result_b_20_24 = whoilo_paf_summary(result_20_24, result_20_241, CI = NULL),
 result_lwr_b_25_29 = whoilo_paf_summary(result_25_29, result_25_291, sd_25_29, sd_25_291, CI = 0.025),
 result_upr_b_25_29 = whoilo_paf_summary(result_25_29, result_25_291, sd_25_29, sd_25_291, CI = 0.975),
 result_b_25_29 = whoilo_paf_summary(result_25_29, result_25_291, CI = NULL),
 result_lwr_b_30_34 = whoilo_paf_summary(result_30_34, result_30_341, sd_30_34, sd_30_341, CI = 0.025),
 result_upr_b_30_34 = whoilo_paf_summary(result_30_34, result_30_341, sd_30_34, sd_30_341, CI = 0.975),
 result_b_30_34 = whoilo_paf_summary(result_30_34, result_30_341, CI = NULL),
 result_lwr_b_35_39 = whoilo_paf_summary(result_35_39, result_35_391, sd_35_39, sd_35_391, CI = 0.025),
 result_upr_b_35_39 = whoilo_paf_summary(result_35_39, result_35_391, sd_35_39, sd_35_391, CI = 0.975),
 result_b_35_39 = whoilo_paf_summary(result_35_39, result_35_391, CI = NULL),
 result_lwr_b_40_44 = whoilo_paf_summary(result_40_44, result_40_441, sd_40_44, sd_40_441, CI = 0.025),
 result_upr_b_40_44 = whoilo_paf_summary(result_40_44, result_40_441, sd_40_44, sd_40_441, CI = 0.975),
 result_b_40_44 = whoilo_paf_summary(result_40_44, result_40_441, CI = NULL),
 result_lwr_b_45_49 = whoilo_paf_summary(result_45_49, result_45_491, sd_45_49, sd_45_491, CI = 0.025),
 result_upr_b_45_49 = whoilo_paf_summary(result_45_49, result_45_491, sd_45_49, sd_45_491, CI = 0.975),
 result_b_45_49 = whoilo_paf_summary(result_45_49, result_45_491, CI = NULL),
 result_lwr_b_50_54 = whoilo_paf_summary(result_50_54, result_50_541, sd_50_54, sd_50_541, CI = 0.025),
 result_upr_b_50_54 = whoilo_paf_summary(result_50_54, result_50_541, sd_50_54, sd_50_541, CI = 0.975),
 result_b_50_54 = whoilo_paf_summary(result_50_54, result_50_541, CI = NULL),
 result_lwr_b_55_59 = whoilo_paf_summary(result_55_59, result_55_591, sd_55_59, sd_55_591, CI = 0.025),
 result_upr_b_55_59 = whoilo_paf_summary(result_55_59, result_55_591, sd_55_59, sd_55_591, CI = 0.975),
 result_b_55_59 = whoilo_paf_summary(result_55_59, result_55_591, CI = NULL),
 result_lwr_b_60_64 = whoilo_paf_summary(result_60_64, result_60_641, sd_60_64, sd_60_641, CI = 0.025),
 result_upr_b_60_64 = whoilo_paf_summary(result_60_64, result_60_641, sd_60_64, sd_60_641, CI = 0.975),
 result_b_60_64 = whoilo_paf_summary(result_60_64, result_60_641, CI = NULL),
 result_lwr_b_65_69 = whoilo_paf_summary(result_65_69, result_65_691, sd_65_69, sd_65_691, CI = 0.025),
 result_upr_b_65_69 = whoilo_paf_summary(result_65_69, result_65_691, sd_65_69, sd_65_691, CI = 0.975),
 result_b_65_69 = whoilo_paf_summary(result_65_69, result_65_691, CI = NULL),
 result_lwr_b_70_74 = whoilo_paf_summary(result_70_74, result_70_741, sd_70_74, sd_70_741, CI = 0.025),
 result_upr_b_70_74 = whoilo_paf_summary(result_70_74, result_70_741, sd_70_74, sd_70_741, CI = 0.975),
 result_b_70_74 = whoilo_paf_summary(result_70_74, result_70_741, CI = NULL),
 result_lwr_b_75_79 = whoilo_paf_summary(result_75_79, result_75_791, sd_75_79, sd_75_791, CI = 0.025),
 result_upr_b_75_79 = whoilo_paf_summary(result_75_79, result_75_791, sd_75_79, sd_75_791, CI = 0.975),
 result_b_75_79 = whoilo_paf_summary(result_75_79, result_75_791, CI = NULL),
 result_lwr_b_80_84 = whoilo_paf_summary(result_80_84, result_80_841, sd_80_84, sd_80_841, CI = 0.025),
 result_upr_b_80_84 = whoilo_paf_summary(result_80_84, result_80_841, sd_80_84, sd_80_841, CI = 0.975),
 result_b_80_84 = whoilo_paf_summary(result_80_84, result_80_841, CI = NULL),
 result_lwr_b_85_89 = whoilo_paf_summary(result_85_89, result_85_891, sd_85_89, sd_85_891, CI = 0.025),
 result_upr_b_85_89 = whoilo_paf_summary(result_85_89, result_85_891, sd_85_89, sd_85_891, CI = 0.975),
 result_b_85_89 = whoilo_paf_summary(result_85_89, result_85_891, CI = NULL),
 result_lwr_b_90_94 = whoilo_paf_summary(result_90_94, result_90_941, sd_90_94, sd_90_941, CI = 0.025),
 result_upr_b_90_94 = whoilo_paf_summary(result_90_94, result_90_941, sd_90_94, sd_90_941, CI = 0.975),
 result_b_90_94 = whoilo_paf_summary(result_90_94, result_90_941, CI = NULL),
 result_lwr_b_95_ = whoilo_paf_summary(result_95_, result_95_1, sd_95_, sd_95_1, CI = 0.025),
 result_upr_b_95_ = whoilo_paf_summary(result_95_, result_95_1, sd_95_, sd_95_1, CI = 0.975),
 result_b_95_ = whoilo_paf_summary(result_95_, result_95_1, CI = NULL)) %>%
 ungroup() %>%
 mutate(sd_b_15_19 = (result_upr_b_15_19 - result_lwr_b_15_19)/2/1.96,
 sd_b_20_24 = (result_upr_b_20_24 - result_lwr_b_20_24)/2/1.96,
 sd_b_25_29 = (result_upr_b_25_29 - result_lwr_b_25_29)/2/1.96,
 sd_b_30_34 = (result_upr_b_30_34 - result_lwr_b_30_34)/2/1.96,
 sd_b_35_39 = (result_upr_b_35_39 - result_lwr_b_35_39)/2/1.96,
 sd_b_40_44 = (result_upr_b_40_44 - result_lwr_b_40_44)/2/1.96,
 sd_b_45_49 = (result_upr_b_45_49 - result_lwr_b_45_49)/2/1.96,
 sd_b_50_54 = (result_upr_b_50_54 - result_lwr_b_50_54)/2/1.96,
 sd_b_55_59 = (result_upr_b_55_59 - result_lwr_b_55_59)/2/1.96,
 sd_b_60_64 = (result_upr_b_60_64 - result_lwr_b_60_64)/2/1.96,
 sd_b_65_69 = (result_upr_b_65_69 - result_lwr_b_65_69)/2/1.96,
 sd_b_70_74 = (result_upr_b_70_74 - result_lwr_b_70_74)/2/1.96,
 sd_b_75_79 = (result_upr_b_75_79 - result_lwr_b_75_79)/2/1.96,
 sd_b_80_84 = (result_upr_b_80_84 - result_lwr_b_80_84)/2/1.96,
 sd_b_85_89 = (result_upr_b_85_89 - result_lwr_b_85_89)/2/1.96,
 sd_b_90_94 = (result_upr_b_90_94 - result_lwr_b_90_94)/2/1.96,
 sd_b_95_ = (result_upr_b_95_ - result_lwr_b_95_)/2/1.96) %>%
 select(region,
 result_b_15_19, sd_b_15_19, result_lwr_b_15_19, result_upr_b_15_19,
 result_b_20_24, sd_b_20_24, result_lwr_b_20_24, result_upr_b_20_24,
 result_b_25_29, sd_b_25_29, result_lwr_b_25_29, result_upr_b_25_29,
 result_b_30_34, sd_b_30_34, result_lwr_b_30_34, result_upr_b_30_34,
 result_b_35_39, sd_b_35_39, result_lwr_b_35_39, result_upr_b_35_39,
 result_b_40_44, sd_b_40_44, result_lwr_b_40_44, result_upr_b_40_44,
 result_b_45_49, sd_b_45_49, result_lwr_b_45_49, result_upr_b_45_49,
 result_b_50_54, sd_b_50_54, result_lwr_b_50_54, result_upr_b_50_54,
 result_b_55_59, sd_b_55_59, result_lwr_b_55_59, result_upr_b_55_59,
 result_b_60_64, sd_b_60_64, result_lwr_b_60_64, result_upr_b_60_64,
 result_b_65_69, sd_b_65_69, result_lwr_b_65_69, result_upr_b_65_69,
 result_b_70_74, sd_b_70_74, result_lwr_b_70_74, result_upr_b_70_74,
 result_b_75_79, sd_b_75_79, result_lwr_b_75_79, result_upr_b_75_79,
 result_b_80_84, sd_b_80_84, result_lwr_b_80_84, result_upr_b_80_84,
 result_b_85_89, sd_b_85_89, result_lwr_b_85_89, result_upr_b_85_89,
 result_b_90_94, sd_b_90_94, result_lwr_b_90_94, result_upr_b_90_94,
 result_b_95_, sd_b_95_, result_lwr_b_95_, result_upr_b_95_)
 }
 }
 # by sexage
 if ("sexage" %in% breakdown){
 if (outcome %in% c("PAF", "DTHpaf")){
 envelope_stroke_sexage_global <- df_in %>%
 filter(outcome == "DTHenvelope",
 year == !!year,
 cause == !!cause) %>%
 arrange(region, country_abbrev, year, sex, ilo_age_bands_5yr) %>%
 drop_na() %>%
 mutate(sd = (result_upr - result_lwr)/2/1.96) %>%
 group_by(region, sex, ilo_age_bands_5yr) %>%
 summarise(result = sum(result),
 sd = sqrt(sum((sd)^2))) %>%
 ungroup %>%
 pivot_wider(names_from = sex:ilo_age_bands_5yr, values_from = result:sd) %>%

 select_all(.funs = funs(str_c(., "1")))

 dths_stroke_sexage_global <- df_in %>%
 filter(outcome == "DTH",
 year == !!year,
 cause == !!cause) %>%
 arrange(region, country_abbrev, year, sex, ilo_age_bands_5yr) %>%
 drop_na() %>%
 mutate(sd = (result_upr - result_lwr)/2/1.96) %>%
 group_by(region, sex, ilo_age_bands_5yr) %>%
 summarise(result = sum(result),
 sd = sqrt(sum((sd)^2))) %>%
 ungroup %>%
 pivot_wider(names_from = sex:ilo_age_bands_5yr, values_from = result:sd)
 dths_stroke_sexage_global <- bind_cols(dths_stroke_sexage_global, envelope_stroke_sexage_global) %>%
 rowwise() %>%
 transmute(region = region,
 result_lwr_f_15_19 = whoilo_paf_summary(result_f_15_19, result_f_15_191, sd_f_15_19, sd_f_15_191, CI = 0.025),
 result_upr_f_15_19 = whoilo_paf_summary(result_f_15_19, result_f_15_191, sd_f_15_19, sd_f_15_191, CI = 0.975),
 result_f_15_19 = whoilo_paf_summary(result_f_15_19, result_f_15_191, CI = NULL),
 result_lwr_f_20_24 = whoilo_paf_summary(result_f_20_24, result_f_20_241, sd_f_20_24, sd_f_20_241, CI = 0.025),
 result_upr_f_20_24 = whoilo_paf_summary(result_f_20_24, result_f_20_241, sd_f_20_24, sd_f_20_241, CI = 0.975),
 result_f_20_24 = whoilo_paf_summary(result_f_20_24, result_f_20_241, CI = NULL),
 result_lwr_f_25_29 = whoilo_paf_summary(result_f_25_29, result_f_25_291, sd_f_25_29, sd_f_25_291, CI = 0.025),
 result_upr_f_25_29 = whoilo_paf_summary(result_f_25_29, result_f_25_291, sd_f_25_29, sd_f_25_291, CI = 0.975),
 result_f_25_29 = whoilo_paf_summary(result_f_25_29, result_f_25_291, CI = NULL),
 result_lwr_f_30_34 = whoilo_paf_summary(result_f_30_34, result_f_30_341, sd_f_30_34, sd_f_30_341, CI = 0.025),
 result_upr_f_30_34 = whoilo_paf_summary(result_f_30_34, result_f_30_341, sd_f_30_34, sd_f_30_341, CI = 0.975),
 result_f_30_34 = whoilo_paf_summary(result_f_30_34, result_f_30_341, CI = NULL),
 result_lwr_f_35_39 = whoilo_paf_summary(result_f_35_39, result_f_35_391, sd_f_35_39, sd_f_35_391, CI = 0.025),
 result_upr_f_35_39 = whoilo_paf_summary(result_f_35_39, result_f_35_391, sd_f_35_39, sd_f_35_391, CI = 0.975),
 result_f_35_39 = whoilo_paf_summary(result_f_35_39, result_f_35_391, CI = NULL),
 result_lwr_f_40_44 = whoilo_paf_summary(result_f_40_44, result_f_40_441, sd_f_40_44, sd_f_40_441, CI = 0.025),
 result_upr_f_40_44 = whoilo_paf_summary(result_f_40_44, result_f_40_441, sd_f_40_44, sd_f_40_441, CI = 0.975),
 result_f_40_44 = whoilo_paf_summary(result_f_40_44, result_f_40_441, CI = NULL),
 result_lwr_f_45_49 = whoilo_paf_summary(result_f_45_49, result_f_45_491, sd_f_45_49, sd_f_45_491, CI = 0.025),
 result_upr_f_45_49 = whoilo_paf_summary(result_f_45_49, result_f_45_491, sd_f_45_49, sd_f_45_491, CI = 0.975),
 result_f_45_49 = whoilo_paf_summary(result_f_45_49, result_f_45_491, CI = NULL),
 result_lwr_f_50_54 = whoilo_paf_summary(result_f_50_54, result_f_50_541, sd_f_50_54, sd_f_50_541, CI = 0.025),
 result_upr_f_50_54 = whoilo_paf_summary(result_f_50_54, result_f_50_541, sd_f_50_54, sd_f_50_541, CI = 0.975),
 result_f_50_54 = whoilo_paf_summary(result_f_50_54, result_f_50_541, CI = NULL),
 result_lwr_f_55_59 = whoilo_paf_summary(result_f_55_59, result_f_55_591, sd_f_55_59, sd_f_55_591, CI = 0.025),
 result_upr_f_55_59 = whoilo_paf_summary(result_f_55_59, result_f_55_591, sd_f_55_59, sd_f_55_591, CI = 0.975),
 result_f_55_59 = whoilo_paf_summary(result_f_55_59, result_f_55_591, CI = NULL),
 result_lwr_f_60_64 = whoilo_paf_summary(result_f_60_64, result_f_60_641, sd_f_60_64, sd_f_60_641, CI = 0.025),
 result_upr_f_60_64 = whoilo_paf_summary(result_f_60_64, result_f_60_641, sd_f_60_64, sd_f_60_641, CI = 0.975),
 result_f_60_64 = whoilo_paf_summary(result_f_60_64, result_f_60_641, CI = NULL),
 result_lwr_f_65_69 = whoilo_paf_summary(result_f_65_69, result_f_65_691, sd_f_65_69, sd_f_65_691, CI = 0.025),
 result_upr_f_65_69 = whoilo_paf_summary(result_f_65_69, result_f_65_691, sd_f_65_69, sd_f_65_691, CI = 0.975),
 result_f_65_69 = whoilo_paf_summary(result_f_65_69, result_f_65_691, CI = NULL),
 result_lwr_f_70_74 = whoilo_paf_summary(result_f_70_74, result_f_70_741, sd_f_70_74, sd_f_70_741, CI = 0.025),
 result_upr_f_70_74 = whoilo_paf_summary(result_f_70_74, result_f_70_741, sd_f_70_74, sd_f_70_741, CI = 0.975),
 result_f_70_74 = whoilo_paf_summary(result_f_70_74, result_f_70_741, CI = NULL),
 result_lwr_f_75_79 = whoilo_paf_summary(result_f_75_79, result_f_75_791, sd_f_75_79, sd_f_75_791, CI = 0.025),
 result_upr_f_75_79 = whoilo_paf_summary(result_f_75_79, result_f_75_791, sd_f_75_79, sd_f_75_791, CI = 0.975),
 result_f_75_79 = whoilo_paf_summary(result_f_75_79, result_f_75_791, CI = NULL),
 result_lwr_f_80_84 = whoilo_paf_summary(result_f_80_84, result_f_80_841, sd_f_80_84, sd_f_80_841, CI = 0.025),
 result_upr_f_80_84 = whoilo_paf_summary(result_f_80_84, result_f_80_841, sd_f_80_84, sd_f_80_841, CI = 0.975),
 result_f_80_84 = whoilo_paf_summary(result_f_80_84, result_f_80_841, CI = NULL),
 result_lwr_f_85_89 = whoilo_paf_summary(result_f_85_89, result_f_85_891, sd_f_85_89, sd_f_85_891, CI = 0.025),
 result_upr_f_85_89 = whoilo_paf_summary(result_f_85_89, result_f_85_891, sd_f_85_89, sd_f_85_891, CI = 0.975),
 result_f_85_89 = whoilo_paf_summary(result_f_85_89, result_f_85_891, CI = NULL),
 result_lwr_f_90_94 = whoilo_paf_summary(result_f_90_94, result_f_90_941, sd_f_90_94, sd_f_90_941, CI = 0.025),
 result_upr_f_90_94 = whoilo_paf_summary(result_f_90_94, result_f_90_941, sd_f_90_94, sd_f_90_941, CI = 0.975),
 result_f_90_94 = whoilo_paf_summary(result_f_90_94, result_f_90_941, CI = NULL),
 result_lwr_f_95_ = whoilo_paf_summary(result_f_95_, result_f_95_1, sd_f_95_, sd_f_95_1, CI = 0.025),
 result_upr_f_95_ = whoilo_paf_summary(result_f_95_, result_f_95_1, sd_f_95_, sd_f_95_1, CI = 0.975),
 result_f_95_ = whoilo_paf_summary(result_f_95_, result_f_95_1, CI = NULL),
 result_lwr_m_15_19 = whoilo_paf_summary(result_m_15_19, result_m_15_191, sd_m_15_19, sd_m_15_191, CI = 0.025),
 result_upr_m_15_19 = whoilo_paf_summary(result_m_15_19, result_m_15_191, sd_m_15_19, sd_m_15_191, CI = 0.975),
 result_m_15_19 = whoilo_paf_summary(result_m_15_19, result_m_15_191, CI = NULL),
 result_lwr_m_20_24 = whoilo_paf_summary(result_m_20_24, result_m_20_241, sd_m_20_24, sd_m_20_241, CI = 0.025),
 result_upr_m_20_24 = whoilo_paf_summary(result_m_20_24, result_m_20_241, sd_m_20_24, sd_m_20_241, CI = 0.975),
 result_m_20_24 = whoilo_paf_summary(result_m_20_24, result_m_20_241, CI = NULL),
 result_lwr_m_25_29 = whoilo_paf_summary(result_m_25_29, result_m_25_291, sd_m_25_29, sd_m_25_291, CI = 0.025),
 result_upr_m_25_29 = whoilo_paf_summary(result_m_25_29, result_m_25_291, sd_m_25_29, sd_m_25_291, CI = 0.975),
 result_m_25_29 = whoilo_paf_summary(result_m_25_29, result_m_25_291, CI = NULL),
 result_lwr_m_30_34 = whoilo_paf_summary(result_m_30_34, result_m_30_341, sd_m_30_34, sd_m_30_341, CI = 0.025),
 result_upr_m_30_34 = whoilo_paf_summary(result_m_30_34, result_m_30_341, sd_m_30_34, sd_m_30_341, CI = 0.975),
 result_m_30_34 = whoilo_paf_summary(result_m_30_34, result_m_30_341, CI = NULL),
 result_lwr_m_35_39 = whoilo_paf_summary(result_m_35_39, result_m_35_391, sd_m_35_39, sd_m_35_391, CI = 0.025),
 result_upr_m_35_39 = whoilo_paf_summary(result_m_35_39, result_m_35_391, sd_m_35_39, sd_m_35_391, CI = 0.975),
 result_m_35_39 = whoilo_paf_summary(result_m_35_39, result_m_35_391, CI = NULL),
 result_lwr_m_40_44 = whoilo_paf_summary(result_m_40_44, result_m_40_441, sd_m_40_44, sd_m_40_441, CI = 0.025),
 result_upr_m_40_44 = whoilo_paf_summary(result_m_40_44, result_m_40_441, sd_m_40_44, sd_m_40_441, CI = 0.975),
 result_m_40_44 = whoilo_paf_summary(result_m_40_44, result_m_40_441, CI = NULL),
 result_lwr_m_45_49 = whoilo_paf_summary(result_m_45_49, result_m_45_491, sd_m_45_49, sd_m_45_491, CI = 0.025),
 result_upr_m_45_49 = whoilo_paf_summary(result_m_45_49, result_m_45_491, sd_m_45_49, sd_m_45_491, CI = 0.975),
 result_m_45_49 = whoilo_paf_summary(result_m_45_49, result_m_45_491, CI = NULL),
 result_lwr_m_50_54 = whoilo_paf_summary(result_m_50_54, result_m_50_541, sd_m_50_54, sd_m_50_541, CI = 0.025),
 result_upr_m_50_54 = whoilo_paf_summary(result_m_50_54, result_m_50_541, sd_m_50_54, sd_m_50_541, CI = 0.975),
 result_m_50_54 = whoilo_paf_summary(result_m_50_54, result_m_50_541, CI = NULL),
 result_lwr_m_55_59 = whoilo_paf_summary(result_m_55_59, result_m_55_591, sd_m_55_59, sd_m_55_591, CI = 0.025),
 result_upr_m_55_59 = whoilo_paf_summary(result_m_55_59, result_m_55_591, sd_m_55_59, sd_m_55_591, CI = 0.975),
 result_m_55_59 = whoilo_paf_summary(result_m_55_59, result_m_55_591, CI = NULL),
 result_lwr_m_60_64 = whoilo_paf_summary(result_m_60_64, result_m_60_641, sd_m_60_64, sd_m_60_641, CI = 0.025),
 result_upr_m_60_64 = whoilo_paf_summary(result_m_60_64, result_m_60_641, sd_m_60_64, sd_m_60_641, CI = 0.975),
 result_m_60_64 = whoilo_paf_summary(result_m_60_64, result_m_60_641, CI = NULL),
 result_lwr_m_65_69 = whoilo_paf_summary(result_m_65_69, result_m_65_691, sd_m_65_69, sd_m_65_691, CI = 0.025),
 result_upr_m_65_69 = whoilo_paf_summary(result_m_65_69, result_m_65_691, sd_m_65_69, sd_m_65_691, CI = 0.975),
 result_m_65_69 = whoilo_paf_summary(result_m_65_69, result_m_65_691, CI = NULL),
 result_lwr_m_70_74 = whoilo_paf_summary(result_m_70_74, result_m_70_741, sd_m_70_74, sd_m_70_741, CI = 0.025),
 result_upr_m_70_74 = whoilo_paf_summary(result_m_70_74, result_m_70_741, sd_m_70_74, sd_m_70_741, CI = 0.975),
 result_m_70_74 = whoilo_paf_summary(result_m_70_74, result_m_70_741, CI = NULL),
 result_lwr_m_75_79 = whoilo_paf_summary(result_m_75_79, result_m_75_791, sd_m_75_79, sd_m_75_791, CI = 0.025),
 result_upr_m_75_79 = whoilo_paf_summary(result_m_75_79, result_m_75_791, sd_m_75_79, sd_m_75_791, CI = 0.975),
 result_m_75_79 = whoilo_paf_summary(result_m_75_79, result_m_75_791, CI = NULL),
 result_lwr_m_80_84 = whoilo_paf_summary(result_m_80_84, result_m_80_841, sd_m_80_84, sd_m_80_841, CI = 0.025),
 result_upr_m_80_84 = whoilo_paf_summary(result_m_80_84, result_m_80_841, sd_m_80_84, sd_m_80_841, CI = 0.975),
 result_m_80_84 = whoilo_paf_summary(result_m_80_84, result_m_80_841, CI = NULL),
 result_lwr_m_85_89 = whoilo_paf_summary(result_m_85_89, result_m_85_891, sd_m_85_89, sd_m_85_891, CI = 0.025),
 result_upr_m_85_89 = whoilo_paf_summary(result_m_85_89, result_m_85_891, sd_m_85_89, sd_m_85_891, CI = 0.975),
 result_m_85_89 = whoilo_paf_summary(result_m_85_89, result_m_85_891, CI = NULL),
 result_lwr_m_90_94 = whoilo_paf_summary(result_m_90_94, result_m_90_941, sd_m_90_94, sd_m_90_941, CI = 0.025),
 result_upr_m_90_94 = whoilo_paf_summary(result_m_90_94, result_m_90_941, sd_m_90_94, sd_m_90_941, CI = 0.975),
 result_m_90_94 = whoilo_paf_summary(result_m_90_94, result_m_90_941, CI = NULL),
 result_lwr_m_95_ = whoilo_paf_summary(result_m_95_, result_m_95_1, sd_m_95_, sd_m_95_1, CI = 0.025),
 result_upr_m_95_ = whoilo_paf_summary(result_m_95_, result_m_95_1, sd_m_95_, sd_m_95_1, CI = 0.975),
 result_m_95_ = whoilo_paf_summary(result_m_95_, result_m_95_1, CI = NULL)) %>%
 ungroup() %>%
 mutate(sd_f_15_19 = (result_upr_f_15_19 - result_lwr_f_15_19)/2/1.96,
 sd_f_20_24 = (result_upr_f_20_24 - result_lwr_f_20_24)/2/1.96,
 sd_f_25_29 = (result_upr_f_25_29 - result_lwr_f_25_29)/2/1.96,
 sd_f_30_34 = (result_upr_f_30_34 - result_lwr_f_30_34)/2/1.96,
 sd_f_35_39 = (result_upr_f_35_39 - result_lwr_f_35_39)/2/1.96,
 sd_f_40_44 = (result_upr_f_40_44 - result_lwr_f_40_44)/2/1.96,
 sd_f_45_49 = (result_upr_f_45_49 - result_lwr_f_45_49)/2/1.96,
 sd_f_50_54 = (result_upr_f_50_54 - result_lwr_f_50_54)/2/1.96,
 sd_f_55_59 = (result_upr_f_55_59 - result_lwr_f_55_59)/2/1.96,
 sd_f_60_64 = (result_upr_f_60_64 - result_lwr_f_60_64)/2/1.96,
 sd_f_65_69 = (result_upr_f_65_69 - result_lwr_f_65_69)/2/1.96,
 sd_f_70_74 = (result_upr_f_70_74 - result_lwr_f_70_74)/2/1.96,
 sd_f_75_79 = (result_upr_f_75_79 - result_lwr_f_75_79)/2/1.96,
 sd_f_80_84 = (result_upr_f_80_84 - result_lwr_f_80_84)/2/1.96,
 sd_f_85_89 = (result_upr_f_85_89 - result_lwr_f_85_89)/2/1.96,
 sd_f_90_94 = (result_upr_f_90_94 - result_lwr_f_90_94)/2/1.96,
 sd_f_95_ = (result_upr_f_95_ - result_lwr_f_95_)/2/1.96,
 sd_m_15_19 = (result_upr_m_15_19 - result_lwr_m_15_19)/2/1.96,
 sd_m_20_24 = (result_upr_m_20_24 - result_lwr_m_20_24)/2/1.96,
 sd_m_25_29 = (result_upr_m_25_29 - result_lwr_m_25_29)/2/1.96,
 sd_m_30_34 = (result_upr_m_30_34 - result_lwr_m_30_34)/2/1.96,
 sd_m_35_39 = (result_upr_m_35_39 - result_lwr_m_35_39)/2/1.96,
 sd_m_40_44 = (result_upr_m_40_44 - result_lwr_m_40_44)/2/1.96,
 sd_m_45_49 = (result_upr_m_45_49 - result_lwr_m_45_49)/2/1.96,
 sd_m_50_54 = (result_upr_m_50_54 - result_lwr_m_50_54)/2/1.96,
 sd_m_55_59 = (result_upr_m_55_59 - result_lwr_m_55_59)/2/1.96,
 sd_m_60_64 = (result_upr_m_60_64 - result_lwr_m_60_64)/2/1.96,
 sd_m_65_69 = (result_upr_m_65_69 - result_lwr_m_65_69)/2/1.96,
 sd_m_70_74 = (result_upr_m_70_74 - result_lwr_m_70_74)/2/1.96,
 sd_m_75_79 = (result_upr_m_75_79 - result_lwr_m_75_79)/2/1.96,
 sd_m_80_84 = (result_upr_m_80_84 - result_lwr_m_80_84)/2/1.96,
 sd_m_85_89 = (result_upr_m_85_89 - result_lwr_m_85_89)/2/1.96,
 sd_m_90_94 = (result_upr_m_90_94 - result_lwr_m_90_94)/2/1.96,
 sd_m_95_ = (result_upr_m_95_ - result_lwr_m_95_)/2/1.96) %>%
 select(region,
 result_f_15_19, sd_f_15_19, result_lwr_f_15_19, result_upr_f_15_19,
 result_f_20_24, sd_f_20_24, result_lwr_f_20_24, result_upr_f_20_24,
 result_f_25_29, sd_f_25_29, result_lwr_f_25_29, result_upr_f_25_29,
 result_f_30_34, sd_f_30_34, result_lwr_f_30_34, result_upr_f_30_34,
 result_f_35_39, sd_f_35_39, result_lwr_f_35_39, result_upr_f_35_39,
 result_f_40_44, sd_f_40_44, result_lwr_f_40_44, result_upr_f_40_44,
 result_f_45_49, sd_f_45_49, result_lwr_f_45_49, result_upr_f_45_49,
 result_f_50_54, sd_f_50_54, result_lwr_f_50_54, result_upr_f_50_54,
 result_f_55_59, sd_f_55_59, result_lwr_f_55_59, result_upr_f_55_59,
 result_f_60_64, sd_f_60_64, result_lwr_f_60_64, result_upr_f_60_64,
 result_f_65_69, sd_f_65_69, result_lwr_f_65_69, result_upr_f_65_69,
 result_f_70_74, sd_f_70_74, result_lwr_f_70_74, result_upr_f_70_74,
 result_f_75_79, sd_f_75_79, result_lwr_f_75_79, result_upr_f_75_79,
 result_f_80_84, sd_f_80_84, result_lwr_f_80_84, result_upr_f_80_84,
 result_f_85_89, sd_f_85_89, result_lwr_f_85_89, result_upr_f_85_89,
 result_f_90_94, sd_f_90_94, result_lwr_f_90_94, result_upr_f_90_94,
 result_f_95_, sd_f_95_, result_lwr_f_95_, result_upr_f_95_,
 result_m_15_19, sd_m_15_19, result_lwr_m_15_19, result_upr_m_15_19,
 result_m_20_24, sd_m_20_24, result_lwr_m_20_24, result_upr_m_20_24,
 result_m_25_29, sd_m_25_29, result_lwr_m_25_29, result_upr_m_25_29,
 result_m_30_34, sd_m_30_34, result_lwr_m_30_34, result_upr_m_30_34,
 result_m_35_39, sd_m_35_39, result_lwr_m_35_39, result_upr_m_35_39,
 result_m_40_44, sd_m_40_44, result_lwr_m_40_44, result_upr_m_40_44,
 result_m_45_49, sd_m_45_49, result_lwr_m_45_49, result_upr_m_45_49,
 result_m_50_54, sd_m_50_54, result_lwr_m_50_54, result_upr_m_50_54,
 result_m_55_59, sd_m_55_59, result_lwr_m_55_59, result_upr_m_55_59,
 result_m_60_64, sd_m_60_64, result_lwr_m_60_64, result_upr_m_60_64,
 result_m_65_69, sd_m_65_69, result_lwr_m_65_69, result_upr_m_65_69,
 result_m_70_74, sd_m_70_74, result_lwr_m_70_74, result_upr_m_70_74,
 result_m_75_79, sd_m_75_79, result_lwr_m_75_79, result_upr_m_75_79,
 result_m_80_84, sd_m_80_84, result_lwr_m_80_84, result_upr_m_80_84,
 result_m_85_89, sd_m_85_89, result_lwr_m_85_89, result_upr_m_85_89,
 result_m_90_94, sd_m_90_94, result_lwr_m_90_94, result_upr_m_90_94,
 result_m_95_, sd_m_95_, result_lwr_m_95_, result_upr_m_95_)
 }
 if (outcome %in% c("DALYpaf")){
 envelope_stroke_sexage_global <- df_in %>%
 filter(outcome == "DALYenvelope",
 year == !!year,
 cause == !!cause) %>%
 mutate(result_lwr = case_when(is.na(result_lwr) ~ result,
 TRUE ~ result_lwr),
 result_upr = case_when(is.na(result_upr) ~ result,
 TRUE ~ result_upr)) %>%
 arrange(region, country_abbrev, year, sex, ilo_age_bands_5yr) %>%
 drop_na() %>%
 mutate(sd = (result_upr - result_lwr)/2/1.96) %>%
 group_by(region, sex, ilo_age_bands_5yr) %>%
 summarise(result = sum(result),
 sd = sqrt(sum((sd)^2))) %>%
 ungroup %>%
 pivot_wider(names_from = sex:ilo_age_bands_5yr, values_from = result:sd) %>%

 select_all(.funs = funs(str_c(., "1")))

 dths_stroke_sexage_global <- df_in %>%
 filter(outcome == "DALY",
 year == !!year,
 cause == !!cause) %>%
 arrange(region, country_abbrev, year, sex, ilo_age_bands_5yr) %>%
 drop_na() %>%
 mutate(sd = (result_upr - result_lwr)/2/1.96) %>%
 group_by(region, sex, ilo_age_bands_5yr) %>%
 summarise(result = sum(result),
 sd = sqrt(sum((sd)^2))) %>%
 ungroup %>%
 pivot_wider(names_from = sex:ilo_age_bands_5yr, values_from = result:sd)
 dths_stroke_sexage_global <- bind_cols(dths_stroke_sexage_global, envelope_stroke_sexage_global) %>%
 rowwise() %>%
 transmute(region = region,
 result_lwr_f_15_19 = whoilo_paf_summary(result_f_15_19, result_f_15_191, sd_f_15_19, sd_f_15_191, CI = 0.025),
 result_upr_f_15_19 = whoilo_paf_summary(result_f_15_19, result_f_15_191, sd_f_15_19, sd_f_15_191, CI = 0.975),
 result_f_15_19 = whoilo_paf_summary(result_f_15_19, result_f_15_191, CI = NULL),
 result_lwr_f_20_24 = whoilo_paf_summary(result_f_20_24, result_f_20_241, sd_f_20_24, sd_f_20_241, CI = 0.025),
 result_upr_f_20_24 = whoilo_paf_summary(result_f_20_24, result_f_20_241, sd_f_20_24, sd_f_20_241, CI = 0.975),
 result_f_20_24 = whoilo_paf_summary(result_f_20_24, result_f_20_241, CI = NULL),
 result_lwr_f_25_29 = whoilo_paf_summary(result_f_25_29, result_f_25_291, sd_f_25_29, sd_f_25_291, CI = 0.025),
 result_upr_f_25_29 = whoilo_paf_summary(result_f_25_29, result_f_25_291, sd_f_25_29, sd_f_25_291, CI = 0.975),
 result_f_25_29 = whoilo_paf_summary(result_f_25_29, result_f_25_291, CI = NULL),
 result_lwr_f_30_34 = whoilo_paf_summary(result_f_30_34, result_f_30_341, sd_f_30_34, sd_f_30_341, CI = 0.025),
 result_upr_f_30_34 = whoilo_paf_summary(result_f_30_34, result_f_30_341, sd_f_30_34, sd_f_30_341, CI = 0.975),
 result_f_30_34 = whoilo_paf_summary(result_f_30_34, result_f_30_341, CI = NULL),
 result_lwr_f_35_39 = whoilo_paf_summary(result_f_35_39, result_f_35_391, sd_f_35_39, sd_f_35_391, CI = 0.025),
 result_upr_f_35_39 = whoilo_paf_summary(result_f_35_39, result_f_35_391, sd_f_35_39, sd_f_35_391, CI = 0.975),
 result_f_35_39 = whoilo_paf_summary(result_f_35_39, result_f_35_391, CI = NULL),
 result_lwr_f_40_44 = whoilo_paf_summary(result_f_40_44, result_f_40_441, sd_f_40_44, sd_f_40_441, CI = 0.025),
 result_upr_f_40_44 = whoilo_paf_summary(result_f_40_44, result_f_40_441, sd_f_40_44, sd_f_40_441, CI = 0.975),
 result_f_40_44 = whoilo_paf_summary(result_f_40_44, result_f_40_441, CI = NULL),
 result_lwr_f_45_49 = whoilo_paf_summary(result_f_45_49, result_f_45_491, sd_f_45_49, sd_f_45_491, CI = 0.025),
 result_upr_f_45_49 = whoilo_paf_summary(result_f_45_49, result_f_45_491, sd_f_45_49, sd_f_45_491, CI = 0.975),
 result_f_45_49 = whoilo_paf_summary(result_f_45_49, result_f_45_491, CI = NULL),
 result_lwr_f_50_54 = whoilo_paf_summary(result_f_50_54, result_f_50_541, sd_f_50_54, sd_f_50_541, CI = 0.025),
 result_upr_f_50_54 = whoilo_paf_summary(result_f_50_54, result_f_50_541, sd_f_50_54, sd_f_50_541, CI = 0.975),
 result_f_50_54 = whoilo_paf_summary(result_f_50_54, result_f_50_541, CI = NULL),
 result_lwr_f_55_59 = whoilo_paf_summary(result_f_55_59, result_f_55_591, sd_f_55_59, sd_f_55_591, CI = 0.025),
 result_upr_f_55_59 = whoilo_paf_summary(result_f_55_59, result_f_55_591, sd_f_55_59, sd_f_55_591, CI = 0.975),
 result_f_55_59 = whoilo_paf_summary(result_f_55_59, result_f_55_591, CI = NULL),
 result_lwr_f_60_64 = whoilo_paf_summary(result_f_60_64, result_f_60_641, sd_f_60_64, sd_f_60_641, CI = 0.025),
 result_upr_f_60_64 = whoilo_paf_summary(result_f_60_64, result_f_60_641, sd_f_60_64, sd_f_60_641, CI = 0.975),
 result_f_60_64 = whoilo_paf_summary(result_f_60_64, result_f_60_641, CI = NULL),
 result_lwr_f_65_69 = whoilo_paf_summary(result_f_65_69, result_f_65_691, sd_f_65_69, sd_f_65_691, CI = 0.025),
 result_upr_f_65_69 = whoilo_paf_summary(result_f_65_69, result_f_65_691, sd_f_65_69, sd_f_65_691, CI = 0.975),
 result_f_65_69 = whoilo_paf_summary(result_f_65_69, result_f_65_691, CI = NULL),
 result_lwr_f_70_74 = whoilo_paf_summary(result_f_70_74, result_f_70_741, sd_f_70_74, sd_f_70_741, CI = 0.025),
 result_upr_f_70_74 = whoilo_paf_summary(result_f_70_74, result_f_70_741, sd_f_70_74, sd_f_70_741, CI = 0.975),
 result_f_70_74 = whoilo_paf_summary(result_f_70_74, result_f_70_741, CI = NULL),
 result_lwr_f_75_79 = whoilo_paf_summary(result_f_75_79, result_f_75_791, sd_f_75_79, sd_f_75_791, CI = 0.025),
 result_upr_f_75_79 = whoilo_paf_summary(result_f_75_79, result_f_75_791, sd_f_75_79, sd_f_75_791, CI = 0.975),
 result_f_75_79 = whoilo_paf_summary(result_f_75_79, result_f_75_791, CI = NULL),
 result_lwr_f_80_84 = whoilo_paf_summary(result_f_80_84, result_f_80_841, sd_f_80_84, sd_f_80_841, CI = 0.025),
 result_upr_f_80_84 = whoilo_paf_summary(result_f_80_84, result_f_80_841, sd_f_80_84, sd_f_80_841, CI = 0.975),
 result_f_80_84 = whoilo_paf_summary(result_f_80_84, result_f_80_841, CI = NULL),
 result_lwr_f_85_89 = whoilo_paf_summary(result_f_85_89, result_f_85_891, sd_f_85_89, sd_f_85_891, CI = 0.025),
 result_upr_f_85_89 = whoilo_paf_summary(result_f_85_89, result_f_85_891, sd_f_85_89, sd_f_85_891, CI = 0.975),
 result_f_85_89 = whoilo_paf_summary(result_f_85_89, result_f_85_891, CI = NULL),
 result_lwr_f_90_94 = whoilo_paf_summary(result_f_90_94, result_f_90_941, sd_f_90_94, sd_f_90_941, CI = 0.025),
 result_upr_f_90_94 = whoilo_paf_summary(result_f_90_94, result_f_90_941, sd_f_90_94, sd_f_90_941, CI = 0.975),
 result_f_90_94 = whoilo_paf_summary(result_f_90_94, result_f_90_941, CI = NULL),
 result_lwr_f_95_ = whoilo_paf_summary(result_f_95_, result_f_95_1, sd_f_95_, sd_f_95_1, CI = 0.025),
 result_upr_f_95_ = whoilo_paf_summary(result_f_95_, result_f_95_1, sd_f_95_, sd_f_95_1, CI = 0.975),
 result_f_95_ = whoilo_paf_summary(result_f_95_, result_f_95_1, CI = NULL),
 result_lwr_m_15_19 = whoilo_paf_summary(result_m_15_19, result_m_15_191, sd_m_15_19, sd_m_15_191, CI = 0.025),
 result_upr_m_15_19 = whoilo_paf_summary(result_m_15_19, result_m_15_191, sd_m_15_19, sd_m_15_191, CI = 0.975),
 result_m_15_19 = whoilo_paf_summary(result_m_15_19, result_m_15_191, CI = NULL),
 result_lwr_m_20_24 = whoilo_paf_summary(result_m_20_24, result_m_20_241, sd_m_20_24, sd_m_20_241, CI = 0.025),
 result_upr_m_20_24 = whoilo_paf_summary(result_m_20_24, result_m_20_241, sd_m_20_24, sd_m_20_241, CI = 0.975),
 result_m_20_24 = whoilo_paf_summary(result_m_20_24, result_m_20_241, CI = NULL),
 result_lwr_m_25_29 = whoilo_paf_summary(result_m_25_29, result_m_25_291, sd_m_25_29, sd_m_25_291, CI = 0.025),
 result_upr_m_25_29 = whoilo_paf_summary(result_m_25_29, result_m_25_291, sd_m_25_29, sd_m_25_291, CI = 0.975),
 result_m_25_29 = whoilo_paf_summary(result_m_25_29, result_m_25_291, CI = NULL),
 result_lwr_m_30_34 = whoilo_paf_summary(result_m_30_34, result_m_30_341, sd_m_30_34, sd_m_30_341, CI = 0.025),
 result_upr_m_30_34 = whoilo_paf_summary(result_m_30_34, result_m_30_341, sd_m_30_34, sd_m_30_341, CI = 0.975),
 result_m_30_34 = whoilo_paf_summary(result_m_30_34, result_m_30_341, CI = NULL),
 result_lwr_m_35_39 = whoilo_paf_summary(result_m_35_39, result_m_35_391, sd_m_35_39, sd_m_35_391, CI = 0.025),
 result_upr_m_35_39 = whoilo_paf_summary(result_m_35_39, result_m_35_391, sd_m_35_39, sd_m_35_391, CI = 0.975),
 result_m_35_39 = whoilo_paf_summary(result_m_35_39, result_m_35_391, CI = NULL),
 result_lwr_m_40_44 = whoilo_paf_summary(result_m_40_44, result_m_40_441, sd_m_40_44, sd_m_40_441, CI = 0.025),
 result_upr_m_40_44 = whoilo_paf_summary(result_m_40_44, result_m_40_441, sd_m_40_44, sd_m_40_441, CI = 0.975),
 result_m_40_44 = whoilo_paf_summary(result_m_40_44, result_m_40_441, CI = NULL),
 result_lwr_m_45_49 = whoilo_paf_summary(result_m_45_49, result_m_45_491, sd_m_45_49, sd_m_45_491, CI = 0.025),
 result_upr_m_45_49 = whoilo_paf_summary(result_m_45_49, result_m_45_491, sd_m_45_49, sd_m_45_491, CI = 0.975),
 result_m_45_49 = whoilo_paf_summary(result_m_45_49, result_m_45_491, CI = NULL),
 result_lwr_m_50_54 = whoilo_paf_summary(result_m_50_54, result_m_50_541, sd_m_50_54, sd_m_50_541, CI = 0.025),
 result_upr_m_50_54 = whoilo_paf_summary(result_m_50_54, result_m_50_541, sd_m_50_54, sd_m_50_541, CI = 0.975),
 result_m_50_54 = whoilo_paf_summary(result_m_50_54, result_m_50_541, CI = NULL),
 result_lwr_m_55_59 = whoilo_paf_summary(result_m_55_59, result_m_55_591, sd_m_55_59, sd_m_55_591, CI = 0.025),
 result_upr_m_55_59 = whoilo_paf_summary(result_m_55_59, result_m_55_591, sd_m_55_59, sd_m_55_591, CI = 0.975),
 result_m_55_59 = whoilo_paf_summary(result_m_55_59, result_m_55_591, CI = NULL),
 result_lwr_m_60_64 = whoilo_paf_summary(result_m_60_64, result_m_60_641, sd_m_60_64, sd_m_60_641, CI = 0.025),
 result_upr_m_60_64 = whoilo_paf_summary(result_m_60_64, result_m_60_641, sd_m_60_64, sd_m_60_641, CI = 0.975),
 result_m_60_64 = whoilo_paf_summary(result_m_60_64, result_m_60_641, CI = NULL),
 result_lwr_m_65_69 = whoilo_paf_summary(result_m_65_69, result_m_65_691, sd_m_65_69, sd_m_65_691, CI = 0.025),
 result_upr_m_65_69 = whoilo_paf_summary(result_m_65_69, result_m_65_691, sd_m_65_69, sd_m_65_691, CI = 0.975),
 result_m_65_69 = whoilo_paf_summary(result_m_65_69, result_m_65_691, CI = NULL),
 result_lwr_m_70_74 = whoilo_paf_summary(result_m_70_74, result_m_70_741, sd_m_70_74, sd_m_70_741, CI = 0.025),
 result_upr_m_70_74 = whoilo_paf_summary(result_m_70_74, result_m_70_741, sd_m_70_74, sd_m_70_741, CI = 0.975),
 result_m_70_74 = whoilo_paf_summary(result_m_70_74, result_m_70_741, CI = NULL),
 result_lwr_m_75_79 = whoilo_paf_summary(result_m_75_79, result_m_75_791, sd_m_75_79, sd_m_75_791, CI = 0.025),
 result_upr_m_75_79 = whoilo_paf_summary(result_m_75_79, result_m_75_791, sd_m_75_79, sd_m_75_791, CI = 0.975),
 result_m_75_79 = whoilo_paf_summary(result_m_75_79, result_m_75_791, CI = NULL),
 result_lwr_m_80_84 = whoilo_paf_summary(result_m_80_84, result_m_80_841, sd_m_80_84, sd_m_80_841, CI = 0.025),
 result_upr_m_80_84 = whoilo_paf_summary(result_m_80_84, result_m_80_841, sd_m_80_84, sd_m_80_841, CI = 0.975),
 result_m_80_84 = whoilo_paf_summary(result_m_80_84, result_m_80_841, CI = NULL),
 result_lwr_m_85_89 = whoilo_paf_summary(result_m_85_89, result_m_85_891, sd_m_85_89, sd_m_85_891, CI = 0.025),
 result_upr_m_85_89 = whoilo_paf_summary(result_m_85_89, result_m_85_891, sd_m_85_89, sd_m_85_891, CI = 0.975),
 result_m_85_89 = whoilo_paf_summary(result_m_85_89, result_m_85_891, CI = NULL),
 result_lwr_m_90_94 = whoilo_paf_summary(result_m_90_94, result_m_90_941, sd_m_90_94, sd_m_90_941, CI = 0.025),
 result_upr_m_90_94 = whoilo_paf_summary(result_m_90_94, result_m_90_941, sd_m_90_94, sd_m_90_941, CI = 0.975),
 result_m_90_94 = whoilo_paf_summary(result_m_90_94, result_m_90_941, CI = NULL),
 result_lwr_m_95_ = whoilo_paf_summary(result_m_95_, result_m_95_1, sd_m_95_, sd_m_95_1, CI = 0.025),
 result_upr_m_95_ = whoilo_paf_summary(result_m_95_, result_m_95_1, sd_m_95_, sd_m_95_1, CI = 0.975),
 result_m_95_ = whoilo_paf_summary(result_m_95_, result_m_95_1, CI = NULL)) %>%
 ungroup() %>%
 mutate(sd_f_15_19 = (result_upr_f_15_19 - result_lwr_f_15_19)/2/1.96,
 sd_f_20_24 = (result_upr_f_20_24 - result_lwr_f_20_24)/2/1.96,
 sd_f_25_29 = (result_upr_f_25_29 - result_lwr_f_25_29)/2/1.96,
 sd_f_30_34 = (result_upr_f_30_34 - result_lwr_f_30_34)/2/1.96,
 sd_f_35_39 = (result_upr_f_35_39 - result_lwr_f_35_39)/2/1.96,
 sd_f_40_44 = (result_upr_f_40_44 - result_lwr_f_40_44)/2/1.96,
 sd_f_45_49 = (result_upr_f_45_49 - result_lwr_f_45_49)/2/1.96,
 sd_f_50_54 = (result_upr_f_50_54 - result_lwr_f_50_54)/2/1.96,
 sd_f_55_59 = (result_upr_f_55_59 - result_lwr_f_55_59)/2/1.96,
 sd_f_60_64 = (result_upr_f_60_64 - result_lwr_f_60_64)/2/1.96,
 sd_f_65_69 = (result_upr_f_65_69 - result_lwr_f_65_69)/2/1.96,
 sd_f_70_74 = (result_upr_f_70_74 - result_lwr_f_70_74)/2/1.96,
 sd_f_75_79 = (result_upr_f_75_79 - result_lwr_f_75_79)/2/1.96,
 sd_f_80_84 = (result_upr_f_80_84 - result_lwr_f_80_84)/2/1.96,
 sd_f_85_89 = (result_upr_f_85_89 - result_lwr_f_85_89)/2/1.96,
 sd_f_90_94 = (result_upr_f_90_94 - result_lwr_f_90_94)/2/1.96,
 sd_f_95_ = (result_upr_f_95_ - result_lwr_f_95_)/2/1.96,
 sd_m_15_19 = (result_upr_m_15_19 - result_lwr_m_15_19)/2/1.96,
 sd_m_20_24 = (result_upr_m_20_24 - result_lwr_m_20_24)/2/1.96,
 sd_m_25_29 = (result_upr_m_25_29 - result_lwr_m_25_29)/2/1.96,
 sd_m_30_34 = (result_upr_m_30_34 - result_lwr_m_30_34)/2/1.96,
 sd_m_35_39 = (result_upr_m_35_39 - result_lwr_m_35_39)/2/1.96,
 sd_m_40_44 = (result_upr_m_40_44 - result_lwr_m_40_44)/2/1.96,
 sd_m_45_49 = (result_upr_m_45_49 - result_lwr_m_45_49)/2/1.96,
 sd_m_50_54 = (result_upr_m_50_54 - result_lwr_m_50_54)/2/1.96,
 sd_m_55_59 = (result_upr_m_55_59 - result_lwr_m_55_59)/2/1.96,
 sd_m_60_64 = (result_upr_m_60_64 - result_lwr_m_60_64)/2/1.96,
 sd_m_65_69 = (result_upr_m_65_69 - result_lwr_m_65_69)/2/1.96,
 sd_m_70_74 = (result_upr_m_70_74 - result_lwr_m_70_74)/2/1.96,
 sd_m_75_79 = (result_upr_m_75_79 - result_lwr_m_75_79)/2/1.96,
 sd_m_80_84 = (result_upr_m_80_84 - result_lwr_m_80_84)/2/1.96,
 sd_m_85_89 = (result_upr_m_85_89 - result_lwr_m_85_89)/2/1.96,
 sd_m_90_94 = (result_upr_m_90_94 - result_lwr_m_90_94)/2/1.96,
 sd_m_95_ = (result_upr_m_95_ - result_lwr_m_95_)/2/1.96) %>%
 select(region,
 result_f_15_19, sd_f_15_19, result_lwr_f_15_19, result_upr_f_15_19,
 result_f_20_24, sd_f_20_24, result_lwr_f_20_24, result_upr_f_20_24,
 result_f_25_29, sd_f_25_29, result_lwr_f_25_29, result_upr_f_25_29,
 result_f_30_34, sd_f_30_34, result_lwr_f_30_34, result_upr_f_30_34,
 result_f_35_39, sd_f_35_39, result_lwr_f_35_39, result_upr_f_35_39,
 result_f_40_44, sd_f_40_44, result_lwr_f_40_44, result_upr_f_40_44,
 result_f_45_49, sd_f_45_49, result_lwr_f_45_49, result_upr_f_45_49,
 result_f_50_54, sd_f_50_54, result_lwr_f_50_54, result_upr_f_50_54,
 result_f_55_59, sd_f_55_59, result_lwr_f_55_59, result_upr_f_55_59,
 result_f_60_64, sd_f_60_64, result_lwr_f_60_64, result_upr_f_60_64,
 result_f_65_69, sd_f_65_69, result_lwr_f_65_69, result_upr_f_65_69,
 result_f_70_74, sd_f_70_74, result_lwr_f_70_74, result_upr_f_70_74,
 result_f_75_79, sd_f_75_79, result_lwr_f_75_79, result_upr_f_75_79,
 result_f_80_84, sd_f_80_84, result_lwr_f_80_84, result_upr_f_80_84,
 result_f_85_89, sd_f_85_89, result_lwr_f_85_89, result_upr_f_85_89,
 result_f_90_94, sd_f_90_94, result_lwr_f_90_94, result_upr_f_90_94,
 result_f_95_, sd_f_95_, result_lwr_f_95_, result_upr_f_95_,
 result_m_15_19, sd_m_15_19, result_lwr_m_15_19, result_upr_m_15_19,
 result_m_20_24, sd_m_20_24, result_lwr_m_20_24, result_upr_m_20_24,
 result_m_25_29, sd_m_25_29, result_lwr_m_25_29, result_upr_m_25_29,
 result_m_30_34, sd_m_30_34, result_lwr_m_30_34, result_upr_m_30_34,
 result_m_35_39, sd_m_35_39, result_lwr_m_35_39, result_upr_m_35_39,
 result_m_40_44, sd_m_40_44, result_lwr_m_40_44, result_upr_m_40_44,
 result_m_45_49, sd_m_45_49, result_lwr_m_45_49, result_upr_m_45_49,
 result_m_50_54, sd_m_50_54, result_lwr_m_50_54, result_upr_m_50_54,
 result_m_55_59, sd_m_55_59, result_lwr_m_55_59, result_upr_m_55_59,
 result_m_60_64, sd_m_60_64, result_lwr_m_60_64, result_upr_m_60_64,
 result_m_65_69, sd_m_65_69, result_lwr_m_65_69, result_upr_m_65_69,
 result_m_70_74, sd_m_70_74, result_lwr_m_70_74, result_upr_m_70_74,
 result_m_75_79, sd_m_75_79, result_lwr_m_75_79, result_upr_m_75_79,
 result_m_80_84, sd_m_80_84, result_lwr_m_80_84, result_upr_m_80_84,
 result_m_85_89, sd_m_85_89, result_lwr_m_85_89, result_upr_m_85_89,
 result_m_90_94, sd_m_90_94, result_lwr_m_90_94, result_upr_m_90_94,
 result_m_95_, sd_m_95_, result_lwr_m_95_, result_upr_m_95_)
 }
 if (outcome %in% c("h0", "h1", "h2", "h3", "h4", "h5")){
 dths_stroke_sexage_global <- df_in %>%
 filter(outcome == !!outcome,
 year == !!year,
 cause == !!cause) %>%
 arrange(region, country_abbrev, year, sex, ilo_age_bands_5yr) %>%
 mutate(sd = (result_upr - result_lwr)/2/1.96) %>%
 group_by(region, sex, ilo_age_bands_5yr) %>%
 left_join(who_ilo_long_populations, by = c("region", "country_abbrev", "year", "sex", "ilo_age_bands_5yr" = "age_bands_5yr")) %>%
 mutate(result = result*population,
 sd = sd*population) %>%
 drop_na() %>%
 summarise(result = sum(result),
 sd = sqrt(sum((sd)^2)),
 population = sum(population)) %>%
 mutate(result_lwr = qnorm(p = 0.025, mean = result, sd = sd)/ population,
 result_upr = qnorm(p = 0.975, mean = result, sd = sd)/ population,
 result = result / population,
 sd = sd/population) %>%
 select(-population) %>%
 ungroup %>%
 pivot_wider(names_from = sex:ilo_age_bands_5yr, values_from = result:result_upr) %>%
 select(region,
 result_f_15_19, sd_f_15_19, result_lwr_f_15_19, result_upr_f_15_19,
 result_f_20_24, sd_f_20_24, result_lwr_f_20_24, result_upr_f_20_24,
 result_f_25_29, sd_f_25_29, result_lwr_f_25_29, result_upr_f_25_29,
 result_f_30_34, sd_f_30_34, result_lwr_f_30_34, result_upr_f_30_34,
 result_f_35_39, sd_f_35_39, result_lwr_f_35_39, result_upr_f_35_39,
 result_f_40_44, sd_f_40_44, result_lwr_f_40_44, result_upr_f_40_44,
 result_f_45_49, sd_f_45_49, result_lwr_f_45_49, result_upr_f_45_49,
 result_f_50_54, sd_f_50_54, result_lwr_f_50_54, result_upr_f_50_54,
 result_f_55_59, sd_f_55_59, result_lwr_f_55_59, result_upr_f_55_59,
 result_f_60_64, sd_f_60_64, result_lwr_f_60_64, result_upr_f_60_64,
 result_f_65_69, sd_f_65_69, result_lwr_f_65_69, result_upr_f_65_69,
 result_f_70_74, sd_f_70_74, result_lwr_f_70_74, result_upr_f_70_74,
 result_f_75_79, sd_f_75_79, result_lwr_f_75_79, result_upr_f_75_79,
 result_f_80_84, sd_f_80_84, result_lwr_f_80_84, result_upr_f_80_84,
 result_f_85_89, sd_f_85_89, result_lwr_f_85_89, result_upr_f_85_89,
 result_f_90_94, sd_f_90_94, result_lwr_f_90_94, result_upr_f_90_94,
 result_f_95_, sd_f_95_, result_lwr_f_95_, result_upr_f_95_,
 result_m_15_19, sd_m_15_19, result_lwr_m_15_19, result_upr_m_15_19,
 result_m_20_24, sd_m_20_24, result_lwr_m_20_24, result_upr_m_20_24,
 result_m_25_29, sd_m_25_29, result_lwr_m_25_29, result_upr_m_25_29,
 result_m_30_34, sd_m_30_34, result_lwr_m_30_34, result_upr_m_30_34,
 result_m_35_39, sd_m_35_39, result_lwr_m_35_39, result_upr_m_35_39,
 result_m_40_44, sd_m_40_44, result_lwr_m_40_44, result_upr_m_40_44,
 result_m_45_49, sd_m_45_49, result_lwr_m_45_49, result_upr_m_45_49,
 result_m_50_54, sd_m_50_54, result_lwr_m_50_54, result_upr_m_50_54,
 result_m_55_59, sd_m_55_59, result_lwr_m_55_59, result_upr_m_55_59,
 result_m_60_64, sd_m_60_64, result_lwr_m_60_64, result_upr_m_60_64,
 result_m_65_69, sd_m_65_69, result_lwr_m_65_69, result_upr_m_65_69,
 result_m_70_74, sd_m_70_74, result_lwr_m_70_74, result_upr_m_70_74,
 result_m_75_79, sd_m_75_79, result_lwr_m_75_79, result_upr_m_75_79,
 result_m_80_84, sd_m_80_84, result_lwr_m_80_84, result_upr_m_80_84,
 result_m_85_89, sd_m_85_89, result_lwr_m_85_89, result_upr_m_85_89,
 result_m_90_94, sd_m_90_94, result_lwr_m_90_94, result_upr_m_90_94,
 result_m_95_, sd_m_95_, result_lwr_m_95_, result_upr_m_95_)
 dths_stroke_sexage_global[dths_stroke_sexage_global < 0] <- 0
 }
 if (outcome %in% c("h0_number", "h1_number", "h2_number", "h3_number", "h4_number", "h5_number")){
 outcome2 <- str_sub(outcome, end = -8L)
 dths_stroke_sexage_global <- df_in %>%
 filter(outcome == !!outcome2,
 year == !!year,
 cause == !!cause) %>%
 arrange(region, country_abbrev, year, sex, ilo_age_bands_5yr) %>%
 mutate(sd = (result_upr - result_lwr)/2/1.96) %>%
 group_by(region, sex, ilo_age_bands_5yr) %>%
 left_join(who_ilo_long_populations, by = c("region", "country_abbrev", "year", "sex", "ilo_age_bands_5yr" = "age_bands_5yr")) %>%
 mutate(population = population * 1000) %>%
 mutate(result = result*population,
 sd = sd*population) %>%
 drop_na() %>%
 summarise(result = sum(result),
 sd = sqrt(sum((sd)^2)),
 population = sum(population)) %>%
 mutate(result_lwr = qnorm(p = 0.025, mean = result, sd = sd),
 result_upr = qnorm(p = 0.975, mean = result, sd = sd),
 result = result,
 sd = sd) %>%
 select(-population) %>%
 ungroup %>%
 pivot_wider(names_from = sex:ilo_age_bands_5yr, values_from = result:result_upr) %>%
 select(region,
 result_f_15_19, sd_f_15_19, result_lwr_f_15_19, result_upr_f_15_19,
 result_f_20_24, sd_f_20_24, result_lwr_f_20_24, result_upr_f_20_24,
 result_f_25_29, sd_f_25_29, result_lwr_f_25_29, result_upr_f_25_29,
 result_f_30_34, sd_f_30_34, result_lwr_f_30_34, result_upr_f_30_34,
 result_f_35_39, sd_f_35_39, result_lwr_f_35_39, result_upr_f_35_39,
 result_f_40_44, sd_f_40_44, result_lwr_f_40_44, result_upr_f_40_44,
 result_f_45_49, sd_f_45_49, result_lwr_f_45_49, result_upr_f_45_49,
 result_f_50_54, sd_f_50_54, result_lwr_f_50_54, result_upr_f_50_54,
 result_f_55_59, sd_f_55_59, result_lwr_f_55_59, result_upr_f_55_59,
 result_f_60_64, sd_f_60_64, result_lwr_f_60_64, result_upr_f_60_64,
 result_f_65_69, sd_f_65_69, result_lwr_f_65_69, result_upr_f_65_69,
 result_f_70_74, sd_f_70_74, result_lwr_f_70_74, result_upr_f_70_74,
 result_f_75_79, sd_f_75_79, result_lwr_f_75_79, result_upr_f_75_79,
 result_f_80_84, sd_f_80_84, result_lwr_f_80_84, result_upr_f_80_84,
 result_f_85_89, sd_f_85_89, result_lwr_f_85_89, result_upr_f_85_89,
 result_f_90_94, sd_f_90_94, result_lwr_f_90_94, result_upr_f_90_94,
 result_f_95_, sd_f_95_, result_lwr_f_95_, result_upr_f_95_,
 result_m_15_19, sd_m_15_19, result_lwr_m_15_19, result_upr_m_15_19,
 result_m_20_24, sd_m_20_24, result_lwr_m_20_24, result_upr_m_20_24,
 result_m_25_29, sd_m_25_29, result_lwr_m_25_29, result_upr_m_25_29,
 result_m_30_34, sd_m_30_34, result_lwr_m_30_34, result_upr_m_30_34,
 result_m_35_39, sd_m_35_39, result_lwr_m_35_39, result_upr_m_35_39,
 result_m_40_44, sd_m_40_44, result_lwr_m_40_44, result_upr_m_40_44,
 result_m_45_49, sd_m_45_49, result_lwr_m_45_49, result_upr_m_45_49,
 result_m_50_54, sd_m_50_54, result_lwr_m_50_54, result_upr_m_50_54,
 result_m_55_59, sd_m_55_59, result_lwr_m_55_59, result_upr_m_55_59,
 result_m_60_64, sd_m_60_64, result_lwr_m_60_64, result_upr_m_60_64,
 result_m_65_69, sd_m_65_69, result_lwr_m_65_69, result_upr_m_65_69,
 result_m_70_74, sd_m_70_74, result_lwr_m_70_74, result_upr_m_70_74,
 result_m_75_79, sd_m_75_79, result_lwr_m_75_79, result_upr_m_75_79,
 result_m_80_84, sd_m_80_84, result_lwr_m_80_84, result_upr_m_80_84,
 result_m_85_89, sd_m_85_89, result_lwr_m_85_89, result_upr_m_85_89,
 result_m_90_94, sd_m_90_94, result_lwr_m_90_94, result_upr_m_90_94,
 result_m_95_, sd_m_95_, result_lwr_m_95_, result_upr_m_95_)
 dths_stroke_sexage_global[dths_stroke_sexage_global < 0] <- 0
 }
 if (outcome %in% c("DTH", "DALY", "DTHenvelope", "DALYenvelope")) {
 dths_stroke_sexage_global <- df_in %>%
 filter(outcome == !!outcome,
 year == !!year,
 cause == !!cause) %>%
 arrange(region, country_abbrev, year, sex, ilo_age_bands_5yr) %>%
 mutate(sd = (result_upr - result_lwr)/2/1.96) %>%
 drop_na(region, result) %>%
 group_by(region, sex, ilo_age_bands_5yr) %>%
 summarise(result = sum(result),
 sd = sqrt(sum((sd)^2))) %>%
 mutate(result_lwr = qnorm(p = 0.025, mean = result, sd = sd),
 result_upr = qnorm(p = 0.975, mean = result, sd = sd)) %>%
 ungroup %>%
 pivot_wider(names_from = sex:ilo_age_bands_5yr, values_from = result:result_upr) %>%
 select(region,
 result_f_15_19, sd_f_15_19, result_lwr_f_15_19, result_upr_f_15_19,
 result_f_20_24, sd_f_20_24, result_lwr_f_20_24, result_upr_f_20_24,
 result_f_25_29, sd_f_25_29, result_lwr_f_25_29, result_upr_f_25_29,
 result_f_30_34, sd_f_30_34, result_lwr_f_30_34, result_upr_f_30_34,
 result_f_35_39, sd_f_35_39, result_lwr_f_35_39, result_upr_f_35_39,
 result_f_40_44, sd_f_40_44, result_lwr_f_40_44, result_upr_f_40_44,
 result_f_45_49, sd_f_45_49, result_lwr_f_45_49, result_upr_f_45_49,
 result_f_50_54, sd_f_50_54, result_lwr_f_50_54, result_upr_f_50_54,
 result_f_55_59, sd_f_55_59, result_lwr_f_55_59, result_upr_f_55_59,
 result_f_60_64, sd_f_60_64, result_lwr_f_60_64, result_upr_f_60_64,
 result_f_65_69, sd_f_65_69, result_lwr_f_65_69, result_upr_f_65_69,
 result_f_70_74, sd_f_70_74, result_lwr_f_70_74, result_upr_f_70_74,
 result_f_75_79, sd_f_75_79, result_lwr_f_75_79, result_upr_f_75_79,
 result_f_80_84, sd_f_80_84, result_lwr_f_80_84, result_upr_f_80_84,
 result_f_85_89, sd_f_85_89, result_lwr_f_85_89, result_upr_f_85_89,
 result_f_90_94, sd_f_90_94, result_lwr_f_90_94, result_upr_f_90_94,
 result_f_95_, sd_f_95_, result_lwr_f_95_, result_upr_f_95_,
 result_m_15_19, sd_m_15_19, result_lwr_m_15_19, result_upr_m_15_19,
 result_m_20_24, sd_m_20_24, result_lwr_m_20_24, result_upr_m_20_24,
 result_m_25_29, sd_m_25_29, result_lwr_m_25_29, result_upr_m_25_29,
 result_m_30_34, sd_m_30_34, result_lwr_m_30_34, result_upr_m_30_34,
 result_m_35_39, sd_m_35_39, result_lwr_m_35_39, result_upr_m_35_39,
 result_m_40_44, sd_m_40_44, result_lwr_m_40_44, result_upr_m_40_44,
 result_m_45_49, sd_m_45_49, result_lwr_m_45_49, result_upr_m_45_49,
 result_m_50_54, sd_m_50_54, result_lwr_m_50_54, result_upr_m_50_54,
 result_m_55_59, sd_m_55_59, result_lwr_m_55_59, result_upr_m_55_59,
 result_m_60_64, sd_m_60_64, result_lwr_m_60_64, result_upr_m_60_64,
 result_m_65_69, sd_m_65_69, result_lwr_m_65_69, result_upr_m_65_69,
 result_m_70_74, sd_m_70_74, result_lwr_m_70_74, result_upr_m_70_74,
 result_m_75_79, sd_m_75_79, result_lwr_m_75_79, result_upr_m_75_79,
 result_m_80_84, sd_m_80_84, result_lwr_m_80_84, result_upr_m_80_84,
 result_m_85_89, sd_m_85_89, result_lwr_m_85_89, result_upr_m_85_89,
 result_m_90_94, sd_m_90_94, result_lwr_m_90_94, result_upr_m_90_94,
 result_m_95_, sd_m_95_, result_lwr_m_95_, result_upr_m_95_)
 }
 if (outcome %in% c("DTHS", "DALYS")) {
 outcome2 <- str_sub(outcome, end = -2L)
 dths_stroke_sexage_global <- df_in %>%
 filter(outcome == !!outcome2,
 year == !!year,
 cause %in% c("stroke_h5", "ihd_h5")) %>%
 arrange(region, country_abbrev, year, sex, ilo_age_bands_5yr) %>%
 mutate(sd = (result_upr - result_lwr)/2/1.96) %>%
 drop_na() %>%
 group_by(region, sex, ilo_age_bands_5yr) %>%
 summarise(result = sum(result),
 sd = sqrt(sum((sd)^2))) %>%
 mutate(result_lwr = qnorm(p = 0.025, mean = result, sd = sd),
 result_upr = qnorm(p = 0.975, mean = result, sd = sd)) %>%
 ungroup %>%
 pivot_wider(names_from = sex:ilo_age_bands_5yr, values_from = result:result_upr) %>%
 select(region,
 result_f_15_19, sd_f_15_19, result_lwr_f_15_19, result_upr_f_15_19,
 result_f_20_24, sd_f_20_24, result_lwr_f_20_24, result_upr_f_20_24,
 result_f_25_29, sd_f_25_29, result_lwr_f_25_29, result_upr_f_25_29,
 result_f_30_34, sd_f_30_34, result_lwr_f_30_34, result_upr_f_30_34,
 result_f_35_39, sd_f_35_39, result_lwr_f_35_39, result_upr_f_35_39,
 result_f_40_44, sd_f_40_44, result_lwr_f_40_44, result_upr_f_40_44,
 result_f_45_49, sd_f_45_49, result_lwr_f_45_49, result_upr_f_45_49,
 result_f_50_54, sd_f_50_54, result_lwr_f_50_54, result_upr_f_50_54,
 result_f_55_59, sd_f_55_59, result_lwr_f_55_59, result_upr_f_55_59,
 result_f_60_64, sd_f_60_64, result_lwr_f_60_64, result_upr_f_60_64,
 result_f_65_69, sd_f_65_69, result_lwr_f_65_69, result_upr_f_65_69,
 result_f_70_74, sd_f_70_74, result_lwr_f_70_74, result_upr_f_70_74,
 result_f_75_79, sd_f_75_79, result_lwr_f_75_79, result_upr_f_75_79,
 result_f_80_84, sd_f_80_84, result_lwr_f_80_84, result_upr_f_80_84,
 result_f_85_89, sd_f_85_89, result_lwr_f_85_89, result_upr_f_85_89,
 result_f_90_94, sd_f_90_94, result_lwr_f_90_94, result_upr_f_90_94,
 result_f_95_, sd_f_95_, result_lwr_f_95_, result_upr_f_95_,
 result_m_15_19, sd_m_15_19, result_lwr_m_15_19, result_upr_m_15_19,
 result_m_20_24, sd_m_20_24, result_lwr_m_20_24, result_upr_m_20_24,
 result_m_25_29, sd_m_25_29, result_lwr_m_25_29, result_upr_m_25_29,
 result_m_30_34, sd_m_30_34, result_lwr_m_30_34, result_upr_m_30_34,
 result_m_35_39, sd_m_35_39, result_lwr_m_35_39, result_upr_m_35_39,
 result_m_40_44, sd_m_40_44, result_lwr_m_40_44, result_upr_m_40_44,
 result_m_45_49, sd_m_45_49, result_lwr_m_45_49, result_upr_m_45_49,
 result_m_50_54, sd_m_50_54, result_lwr_m_50_54, result_upr_m_50_54,
 result_m_55_59, sd_m_55_59, result_lwr_m_55_59, result_upr_m_55_59,
 result_m_60_64, sd_m_60_64, result_lwr_m_60_64, result_upr_m_60_64,
 result_m_65_69, sd_m_65_69, result_lwr_m_65_69, result_upr_m_65_69,
 result_m_70_74, sd_m_70_74, result_lwr_m_70_74, result_upr_m_70_74,
 result_m_75_79, sd_m_75_79, result_lwr_m_75_79, result_upr_m_75_79,
 result_m_80_84, sd_m_80_84, result_lwr_m_80_84, result_upr_m_80_84,
 result_m_85_89, sd_m_85_89, result_lwr_m_85_89, result_upr_m_85_89,
 result_m_90_94, sd_m_90_94, result_lwr_m_90_94, result_upr_m_90_94,
 result_m_95_, sd_m_95_, result_lwr_m_95_, result_upr_m_95_)
 }
 if (outcome %in% c("h45")){
 dths_stroke_sexage_global <- df_in %>%
 filter(outcome %in% c("h4", "h5"),
 year == !!year,
 cause == !!cause) %>%
 arrange(region, country_abbrev, year, sex, ilo_age_bands_5yr) %>%
 mutate(sd = (result_upr - result_lwr)/2/1.96) %>%
 group_by(region, sex, ilo_age_bands_5yr) %>%
 left_join(who_ilo_long_populations, by = c("region", "country_abbrev", "year", "sex", "ilo_age_bands_5yr" = "age_bands_5yr")) %>%
 mutate(result = result*population,
 sd = sd*population) %>%
 drop_na() %>%
 summarise(result = sum(result),
 sd = sqrt(sum((sd)^2)),
 population = sum(population)/2) %>%
 mutate(result_lwr = qnorm(p = 0.025, mean = result, sd = sd)/ population,
 result_upr = qnorm(p = 0.975, mean = result, sd = sd)/ population,
 result = result / population,
 sd = sd/population) %>%
 select(-population) %>%
 ungroup %>%
 pivot_wider(names_from = sex:ilo_age_bands_5yr, values_from = result:result_upr) %>%
 select(region,
 result_f_15_19, sd_f_15_19, result_lwr_f_15_19, result_upr_f_15_19,
 result_f_20_24, sd_f_20_24, result_lwr_f_20_24, result_upr_f_20_24,
 result_f_25_29, sd_f_25_29, result_lwr_f_25_29, result_upr_f_25_29,
 result_f_30_34, sd_f_30_34, result_lwr_f_30_34, result_upr_f_30_34,
 result_f_35_39, sd_f_35_39, result_lwr_f_35_39, result_upr_f_35_39,
 result_f_40_44, sd_f_40_44, result_lwr_f_40_44, result_upr_f_40_44,
 result_f_45_49, sd_f_45_49, result_lwr_f_45_49, result_upr_f_45_49,
 result_f_50_54, sd_f_50_54, result_lwr_f_50_54, result_upr_f_50_54,
 result_f_55_59, sd_f_55_59, result_lwr_f_55_59, result_upr_f_55_59,
 result_f_60_64, sd_f_60_64, result_lwr_f_60_64, result_upr_f_60_64,
 result_f_65_69, sd_f_65_69, result_lwr_f_65_69, result_upr_f_65_69,
 result_f_70_74, sd_f_70_74, result_lwr_f_70_74, result_upr_f_70_74,
 result_f_75_79, sd_f_75_79, result_lwr_f_75_79, result_upr_f_75_79,
 result_f_80_84, sd_f_80_84, result_lwr_f_80_84, result_upr_f_80_84,
 result_f_85_89, sd_f_85_89, result_lwr_f_85_89, result_upr_f_85_89,
 result_f_90_94, sd_f_90_94, result_lwr_f_90_94, result_upr_f_90_94,
 result_f_95_, sd_f_95_, result_lwr_f_95_, result_upr_f_95_,
 result_m_15_19, sd_m_15_19, result_lwr_m_15_19, result_upr_m_15_19,
 result_m_20_24, sd_m_20_24, result_lwr_m_20_24, result_upr_m_20_24,
 result_m_25_29, sd_m_25_29, result_lwr_m_25_29, result_upr_m_25_29,
 result_m_30_34, sd_m_30_34, result_lwr_m_30_34, result_upr_m_30_34,
 result_m_35_39, sd_m_35_39, result_lwr_m_35_39, result_upr_m_35_39,
 result_m_40_44, sd_m_40_44, result_lwr_m_40_44, result_upr_m_40_44,
 result_m_45_49, sd_m_45_49, result_lwr_m_45_49, result_upr_m_45_49,
 result_m_50_54, sd_m_50_54, result_lwr_m_50_54, result_upr_m_50_54,
 result_m_55_59, sd_m_55_59, result_lwr_m_55_59, result_upr_m_55_59,
 result_m_60_64, sd_m_60_64, result_lwr_m_60_64, result_upr_m_60_64,
 result_m_65_69, sd_m_65_69, result_lwr_m_65_69, result_upr_m_65_69,
 result_m_70_74, sd_m_70_74, result_lwr_m_70_74, result_upr_m_70_74,
 result_m_75_79, sd_m_75_79, result_lwr_m_75_79, result_upr_m_75_79,
 result_m_80_84, sd_m_80_84, result_lwr_m_80_84, result_upr_m_80_84,
 result_m_85_89, sd_m_85_89, result_lwr_m_85_89, result_upr_m_85_89,
 result_m_90_94, sd_m_90_94, result_lwr_m_90_94, result_upr_m_90_94,
 result_m_95_, sd_m_95_, result_lwr_m_95_, result_upr_m_95_)
 dths_stroke_sexage_global[dths_stroke_sexage_global < 0] <- 0
 }
 if (outcome %in% c("DTHrate")){
 outcome2 <- case_when(outcome == "DTHrate" ~ "DTH",
 outcome == "DALYrate" ~ "DALY",
 TRUE ~ NA_character_)
 dths_stroke_sexage_global <- df_in %>%
 filter(outcome == !!outcome2,
 year == !!year,
 cause == !!cause) %>%
 arrange(region, country_abbrev, year, sex, ilo_age_bands_5yr) %>%
 mutate(sd = (result_upr - result_lwr)/2/1.96) %>%
 group_by(region, sex, ilo_age_bands_5yr) %>%
 left_join(who_ilo_long_populations, by = c("region", "country_abbrev", "year", "sex", "ilo_age_bands_5yr" = "age_bands_5yr")) %>%
 drop_na() %>%
 summarise(result = sum(result),
 sd = sqrt(sum((sd)^2)),
 population = sum(population)) %>%
 mutate(population = population / 100,
 result = result / population,
 sd = sd / population,
 result_lwr = qnorm(p = 0.025, mean = result, sd = sd),
 result_upr = qnorm(p = 0.975, mean = result, sd = sd)) %>%
 select(-population) %>%
 ungroup %>%
 pivot_wider(names_from = sex:ilo_age_bands_5yr, values_from = result:result_upr) %>%
 select(region,
 result_f_15_19, sd_f_15_19, result_lwr_f_15_19, result_upr_f_15_19,
 result_f_20_24, sd_f_20_24, result_lwr_f_20_24, result_upr_f_20_24,
 result_f_25_29, sd_f_25_29, result_lwr_f_25_29, result_upr_f_25_29,
 result_f_30_34, sd_f_30_34, result_lwr_f_30_34, result_upr_f_30_34,
 result_f_35_39, sd_f_35_39, result_lwr_f_35_39, result_upr_f_35_39,
 result_f_40_44, sd_f_40_44, result_lwr_f_40_44, result_upr_f_40_44,
 result_f_45_49, sd_f_45_49, result_lwr_f_45_49, result_upr_f_45_49,
 result_f_50_54, sd_f_50_54, result_lwr_f_50_54, result_upr_f_50_54,
 result_f_55_59, sd_f_55_59, result_lwr_f_55_59, result_upr_f_55_59,
 result_f_60_64, sd_f_60_64, result_lwr_f_60_64, result_upr_f_60_64,
 result_f_65_69, sd_f_65_69, result_lwr_f_65_69, result_upr_f_65_69,
 result_f_70_74, sd_f_70_74, result_lwr_f_70_74, result_upr_f_70_74,
 result_f_75_79, sd_f_75_79, result_lwr_f_75_79, result_upr_f_75_79,
 result_f_80_84, sd_f_80_84, result_lwr_f_80_84, result_upr_f_80_84,
 result_f_85_89, sd_f_85_89, result_lwr_f_85_89, result_upr_f_85_89,
 result_f_90_94, sd_f_90_94, result_lwr_f_90_94, result_upr_f_90_94,
 result_f_95_, sd_f_95_, result_lwr_f_95_, result_upr_f_95_,
 result_m_15_19, sd_m_15_19, result_lwr_m_15_19, result_upr_m_15_19,
 result_m_20_24, sd_m_20_24, result_lwr_m_20_24, result_upr_m_20_24,
 result_m_25_29, sd_m_25_29, result_lwr_m_25_29, result_upr_m_25_29,
 result_m_30_34, sd_m_30_34, result_lwr_m_30_34, result_upr_m_30_34,
 result_m_35_39, sd_m_35_39, result_lwr_m_35_39, result_upr_m_35_39,
 result_m_40_44, sd_m_40_44, result_lwr_m_40_44, result_upr_m_40_44,
 result_m_45_49, sd_m_45_49, result_lwr_m_45_49, result_upr_m_45_49,
 result_m_50_54, sd_m_50_54, result_lwr_m_50_54, result_upr_m_50_54,
 result_m_55_59, sd_m_55_59, result_lwr_m_55_59, result_upr_m_55_59,
 result_m_60_64, sd_m_60_64, result_lwr_m_60_64, result_upr_m_60_64,
 result_m_65_69, sd_m_65_69, result_lwr_m_65_69, result_upr_m_65_69,
 result_m_70_74, sd_m_70_74, result_lwr_m_70_74, result_upr_m_70_74,
 result_m_75_79, sd_m_75_79, result_lwr_m_75_79, result_upr_m_75_79,
 result_m_80_84, sd_m_80_84, result_lwr_m_80_84, result_upr_m_80_84,
 result_m_85_89, sd_m_85_89, result_lwr_m_85_89, result_upr_m_85_89,
 result_m_90_94, sd_m_90_94, result_lwr_m_90_94, result_upr_m_90_94,
 result_m_95_, sd_m_95_, result_lwr_m_95_, result_upr_m_95_)
 }
 if (outcome %in% c("DALYrate")){
 outcome2 <- case_when(outcome == "DTHrate" ~ "DTH",
 outcome == "DALYrate" ~ "DALY",
 TRUE ~ NA_character_)
 dths_stroke_sexage_global <- df_in %>%
 filter(outcome == !!outcome2,
 year == !!year,
 cause == !!cause) %>%
 arrange(region, country_abbrev, year, sex, ilo_age_bands_5yr) %>%
 mutate(sd = (result_upr - result_lwr)/2/1.96) %>%
 group_by(region, sex, ilo_age_bands_5yr) %>%
 left_join(who_ilo_long_populations, by = c("region", "country_abbrev", "year", "sex", "ilo_age_bands_5yr" = "age_bands_5yr")) %>%
 drop_na() %>%
 summarise(result = sum(result),
 sd = sqrt(sum((sd)^2)),
 population = sum(population)) %>%
 mutate(population = population,
 result = result / population * 100000,
 sd = sd / population * 100000,
 result_lwr = qnorm(p = 0.025, mean = result, sd = sd),
 result_upr = qnorm(p = 0.975, mean = result, sd = sd)) %>%
 select(-population) %>%
 ungroup %>%
 pivot_wider(names_from = sex:ilo_age_bands_5yr, values_from = result:result_upr) %>%
 select(region,
 result_f_15_19, sd_f_15_19, result_lwr_f_15_19, result_upr_f_15_19,
 result_f_20_24, sd_f_20_24, result_lwr_f_20_24, result_upr_f_20_24,
 result_f_25_29, sd_f_25_29, result_lwr_f_25_29, result_upr_f_25_29,
 result_f_30_34, sd_f_30_34, result_lwr_f_30_34, result_upr_f_30_34,
 result_f_35_39, sd_f_35_39, result_lwr_f_35_39, result_upr_f_35_39,
 result_f_40_44, sd_f_40_44, result_lwr_f_40_44, result_upr_f_40_44,
 result_f_45_49, sd_f_45_49, result_lwr_f_45_49, result_upr_f_45_49,
 result_f_50_54, sd_f_50_54, result_lwr_f_50_54, result_upr_f_50_54,
 result_f_55_59, sd_f_55_59, result_lwr_f_55_59, result_upr_f_55_59,
 result_f_60_64, sd_f_60_64, result_lwr_f_60_64, result_upr_f_60_64,
 result_f_65_69, sd_f_65_69, result_lwr_f_65_69, result_upr_f_65_69,
 result_f_70_74, sd_f_70_74, result_lwr_f_70_74, result_upr_f_70_74,
 result_f_75_79, sd_f_75_79, result_lwr_f_75_79, result_upr_f_75_79,
 result_f_80_84, sd_f_80_84, result_lwr_f_80_84, result_upr_f_80_84,
 result_f_85_89, sd_f_85_89, result_lwr_f_85_89, result_upr_f_85_89,
 result_f_90_94, sd_f_90_94, result_lwr_f_90_94, result_upr_f_90_94,
 result_f_95_, sd_f_95_, result_lwr_f_95_, result_upr_f_95_,
 result_m_15_19, sd_m_15_19, result_lwr_m_15_19, result_upr_m_15_19,
 result_m_20_24, sd_m_20_24, result_lwr_m_20_24, result_upr_m_20_24,
 result_m_25_29, sd_m_25_29, result_lwr_m_25_29, result_upr_m_25_29,
 result_m_30_34, sd_m_30_34, result_lwr_m_30_34, result_upr_m_30_34,
 result_m_35_39, sd_m_35_39, result_lwr_m_35_39, result_upr_m_35_39,
 result_m_40_44, sd_m_40_44, result_lwr_m_40_44, result_upr_m_40_44,
 result_m_45_49, sd_m_45_49, result_lwr_m_45_49, result_upr_m_45_49,
 result_m_50_54, sd_m_50_54, result_lwr_m_50_54, result_upr_m_50_54,
 result_m_55_59, sd_m_55_59, result_lwr_m_55_59, result_upr_m_55_59,
 result_m_60_64, sd_m_60_64, result_lwr_m_60_64, result_upr_m_60_64,
 result_m_65_69, sd_m_65_69, result_lwr_m_65_69, result_upr_m_65_69,
 result_m_70_74, sd_m_70_74, result_lwr_m_70_74, result_upr_m_70_74,
 result_m_75_79, sd_m_75_79, result_lwr_m_75_79, result_upr_m_75_79,
 result_m_80_84, sd_m_80_84, result_lwr_m_80_84, result_upr_m_80_84,
 result_m_85_89, sd_m_85_89, result_lwr_m_85_89, result_upr_m_85_89,
 result_m_90_94, sd_m_90_94, result_lwr_m_90_94, result_upr_m_90_94,
 result_m_95_, sd_m_95_, result_lwr_m_95_, result_upr_m_95_)
 }
 if (outcome %in% c("population")){
 dths_stroke_sexage_global <- who_ilo_long_populations %>%
 filter(age_bands_5yr %in% c("15_19", "20_24", "25_29", "30_34", "35_39", "40_44", "45_49", "50_54", "55_59", "60_64", "65_69", "70_74", "75_79", "80_84", "85_89", "90_94", "95_"),
 year == !!year) %>%
 arrange(region, country_abbrev, year, sex, age_bands_5yr) %>%
 drop_na() %>%
 group_by(region, sex, age_bands_5yr) %>%
 summarise(result = sum(population)) %>%
 mutate(sd = NA,
 result_lwr = NA,
 result_upr = NA) %>%
 ungroup %>%
 pivot_wider(names_from = sex:age_bands_5yr, values_from = result:result_upr) %>%
 select(region,
 result_f_15_19, sd_f_15_19, result_lwr_f_15_19, result_upr_f_15_19,
 result_f_20_24, sd_f_20_24, result_lwr_f_20_24, result_upr_f_20_24,
 result_f_25_29, sd_f_25_29, result_lwr_f_25_29, result_upr_f_25_29,
 result_f_30_34, sd_f_30_34, result_lwr_f_30_34, result_upr_f_30_34,
 result_f_35_39, sd_f_35_39, result_lwr_f_35_39, result_upr_f_35_39,
 result_f_40_44, sd_f_40_44, result_lwr_f_40_44, result_upr_f_40_44,
 result_f_45_49, sd_f_45_49, result_lwr_f_45_49, result_upr_f_45_49,
 result_f_50_54, sd_f_50_54, result_lwr_f_50_54, result_upr_f_50_54,
 result_f_55_59, sd_f_55_59, result_lwr_f_55_59, result_upr_f_55_59,
 result_f_60_64, sd_f_60_64, result_lwr_f_60_64, result_upr_f_60_64,
 result_f_65_69, sd_f_65_69, result_lwr_f_65_69, result_upr_f_65_69,
 result_f_70_74, sd_f_70_74, result_lwr_f_70_74, result_upr_f_70_74,
 result_f_75_79, sd_f_75_79, result_lwr_f_75_79, result_upr_f_75_79,
 result_f_80_84, sd_f_80_84, result_lwr_f_80_84, result_upr_f_80_84,
 result_f_85_89, sd_f_85_89, result_lwr_f_85_89, result_upr_f_85_89,
 result_f_90_94, sd_f_90_94, result_lwr_f_90_94, result_upr_f_90_94,
 result_f_95_, sd_f_95_, result_lwr_f_95_, result_upr_f_95_,
 result_m_15_19, sd_m_15_19, result_lwr_m_15_19, result_upr_m_15_19,
 result_m_20_24, sd_m_20_24, result_lwr_m_20_24, result_upr_m_20_24,
 result_m_25_29, sd_m_25_29, result_lwr_m_25_29, result_upr_m_25_29,
 result_m_30_34, sd_m_30_34, result_lwr_m_30_34, result_upr_m_30_34,
 result_m_35_39, sd_m_35_39, result_lwr_m_35_39, result_upr_m_35_39,
 result_m_40_44, sd_m_40_44, result_lwr_m_40_44, result_upr_m_40_44,
 result_m_45_49, sd_m_45_49, result_lwr_m_45_49, result_upr_m_45_49,
 result_m_50_54, sd_m_50_54, result_lwr_m_50_54, result_upr_m_50_54,
 result_m_55_59, sd_m_55_59, result_lwr_m_55_59, result_upr_m_55_59,
 result_m_60_64, sd_m_60_64, result_lwr_m_60_64, result_upr_m_60_64,
 result_m_65_69, sd_m_65_69, result_lwr_m_65_69, result_upr_m_65_69,
 result_m_70_74, sd_m_70_74, result_lwr_m_70_74, result_upr_m_70_74,
 result_m_75_79, sd_m_75_79, result_lwr_m_75_79, result_upr_m_75_79,
 result_m_80_84, sd_m_80_84, result_lwr_m_80_84, result_upr_m_80_84,
 result_m_85_89, sd_m_85_89, result_lwr_m_85_89, result_upr_m_85_89,
 result_m_90_94, sd_m_90_94, result_lwr_m_90_94, result_upr_m_90_94,
 result_m_95_, sd_m_95_, result_lwr_m_95_, result_upr_m_95_)
 }
 if (outcome %in% c("DTHratio")){
 envelope_stroke_sexage_global <- df_in %>%
 filter(outcome == "DTHparent",
 year == !!year,
 cause == !!cause) %>%
 arrange(region, country_abbrev, year, sex, ilo_age_bands_5yr) %>%
 drop_na() %>%
 mutate(sd = (result_upr - result_lwr)/2/1.96) %>%
 group_by(region, sex, ilo_age_bands_5yr) %>%
 summarise(result = sum(result),
 sd = sqrt(sum((sd)^2))) %>%
 ungroup %>%
 pivot_wider(names_from = sex:ilo_age_bands_5yr, values_from = result:sd)

 dths_stroke_sexage_global <- df_in %>%
 filter(outcome == "DTHenvelope",
 year == !!year,
 cause == !!cause) %>%
 arrange(region, country_abbrev, year, sex, ilo_age_bands_5yr) %>%
 drop_na() %>%
 mutate(sd = (result_upr - result_lwr)/2/1.96) %>%
 group_by(region, sex, ilo_age_bands_5yr) %>%
 summarise(result = sum(result),
 sd = sqrt(sum((sd)^2))) %>%
 ungroup %>%
 pivot_wider(names_from = sex:ilo_age_bands_5yr, values_from = result:sd) %>%

 select_all(.funs = funs(str_c(., "1")))

 dths_stroke_sexage_global <- bind_cols(dths_stroke_sexage_global, envelope_stroke_sexage_global) %>%
 rowwise() %>%
 transmute(region = region,
 result_lwr_f_15_19 = whoilo_paf_summary(result_f_15_19, result_f_15_191, sd_f_15_19, sd_f_15_191, CI = 0.025),
 result_upr_f_15_19 = whoilo_paf_summary(result_f_15_19, result_f_15_191, sd_f_15_19, sd_f_15_191, CI = 0.975),
 result_f_15_19 = whoilo_paf_summary(result_f_15_19, result_f_15_191, CI = NULL),
 result_lwr_f_20_24 = whoilo_paf_summary(result_f_20_24, result_f_20_241, sd_f_20_24, sd_f_20_241, CI = 0.025),
 result_upr_f_20_24 = whoilo_paf_summary(result_f_20_24, result_f_20_241, sd_f_20_24, sd_f_20_241, CI = 0.975),
 result_f_20_24 = whoilo_paf_summary(result_f_20_24, result_f_20_241, CI = NULL),
 result_lwr_f_25_29 = whoilo_paf_summary(result_f_25_29, result_f_25_291, sd_f_25_29, sd_f_25_291, CI = 0.025),
 result_upr_f_25_29 = whoilo_paf_summary(result_f_25_29, result_f_25_291, sd_f_25_29, sd_f_25_291, CI = 0.975),
 result_f_25_29 = whoilo_paf_summary(result_f_25_29, result_f_25_291, CI = NULL),
 result_lwr_f_30_34 = whoilo_paf_summary(result_f_30_34, result_f_30_341, sd_f_30_34, sd_f_30_341, CI = 0.025),
 result_upr_f_30_34 = whoilo_paf_summary(result_f_30_34, result_f_30_341, sd_f_30_34, sd_f_30_341, CI = 0.975),
 result_f_30_34 = whoilo_paf_summary(result_f_30_34, result_f_30_341, CI = NULL),
 result_lwr_f_35_39 = whoilo_paf_summary(result_f_35_39, result_f_35_391, sd_f_35_39, sd_f_35_391, CI = 0.025),
 result_upr_f_35_39 = whoilo_paf_summary(result_f_35_39, result_f_35_391, sd_f_35_39, sd_f_35_391, CI = 0.975),
 result_f_35_39 = whoilo_paf_summary(result_f_35_39, result_f_35_391, CI = NULL),
 result_lwr_f_40_44 = whoilo_paf_summary(result_f_40_44, result_f_40_441, sd_f_40_44, sd_f_40_441, CI = 0.025),
 result_upr_f_40_44 = whoilo_paf_summary(result_f_40_44, result_f_40_441, sd_f_40_44, sd_f_40_441, CI = 0.975),
 result_f_40_44 = whoilo_paf_summary(result_f_40_44, result_f_40_441, CI = NULL),
 result_lwr_f_45_49 = whoilo_paf_summary(result_f_45_49, result_f_45_491, sd_f_45_49, sd_f_45_491, CI = 0.025),
 result_upr_f_45_49 = whoilo_paf_summary(result_f_45_49, result_f_45_491, sd_f_45_49, sd_f_45_491, CI = 0.975),
 result_f_45_49 = whoilo_paf_summary(result_f_45_49, result_f_45_491, CI = NULL),
 result_lwr_f_50_54 = whoilo_paf_summary(result_f_50_54, result_f_50_541, sd_f_50_54, sd_f_50_541, CI = 0.025),
 result_upr_f_50_54 = whoilo_paf_summary(result_f_50_54, result_f_50_541, sd_f_50_54, sd_f_50_541, CI = 0.975),
 result_f_50_54 = whoilo_paf_summary(result_f_50_54, result_f_50_541, CI = NULL),
 result_lwr_f_55_59 = whoilo_paf_summary(result_f_55_59, result_f_55_591, sd_f_55_59, sd_f_55_591, CI = 0.025),
 result_upr_f_55_59 = whoilo_paf_summary(result_f_55_59, result_f_55_591, sd_f_55_59, sd_f_55_591, CI = 0.975),
 result_f_55_59 = whoilo_paf_summary(result_f_55_59, result_f_55_591, CI = NULL),
 result_lwr_f_60_64 = whoilo_paf_summary(result_f_60_64, result_f_60_641, sd_f_60_64, sd_f_60_641, CI = 0.025),
 result_upr_f_60_64 = whoilo_paf_summary(result_f_60_64, result_f_60_641, sd_f_60_64, sd_f_60_641, CI = 0.975),
 result_f_60_64 = whoilo_paf_summary(result_f_60_64, result_f_60_641, CI = NULL),
 result_lwr_f_65_69 = whoilo_paf_summary(result_f_65_69, result_f_65_691, sd_f_65_69, sd_f_65_691, CI = 0.025),
 result_upr_f_65_69 = whoilo_paf_summary(result_f_65_69, result_f_65_691, sd_f_65_69, sd_f_65_691, CI = 0.975),
 result_f_65_69 = whoilo_paf_summary(result_f_65_69, result_f_65_691, CI = NULL),
 result_lwr_f_70_74 = whoilo_paf_summary(result_f_70_74, result_f_70_741, sd_f_70_74, sd_f_70_741, CI = 0.025),
 result_upr_f_70_74 = whoilo_paf_summary(result_f_70_74, result_f_70_741, sd_f_70_74, sd_f_70_741, CI = 0.975),
 result_f_70_74 = whoilo_paf_summary(result_f_70_74, result_f_70_741, CI = NULL),
 result_lwr_f_75_79 = whoilo_paf_summary(result_f_75_79, result_f_75_791, sd_f_75_79, sd_f_75_791, CI = 0.025),
 result_upr_f_75_79 = whoilo_paf_summary(result_f_75_79, result_f_75_791, sd_f_75_79, sd_f_75_791, CI = 0.975),
 result_f_75_79 = whoilo_paf_summary(result_f_75_79, result_f_75_791, CI = NULL),
 result_lwr_f_80_84 = whoilo_paf_summary(result_f_80_84, result_f_80_841, sd_f_80_84, sd_f_80_841, CI = 0.025),
 result_upr_f_80_84 = whoilo_paf_summary(result_f_80_84, result_f_80_841, sd_f_80_84, sd_f_80_841, CI = 0.975),
 result_f_80_84 = whoilo_paf_summary(result_f_80_84, result_f_80_841, CI = NULL),
 result_lwr_f_85_89 = whoilo_paf_summary(result_f_85_89, result_f_85_891, sd_f_85_89, sd_f_85_891, CI = 0.025),
 result_upr_f_85_89 = whoilo_paf_summary(result_f_85_89, result_f_85_891, sd_f_85_89, sd_f_85_891, CI = 0.975),
 result_f_85_89 = whoilo_paf_summary(result_f_85_89, result_f_85_891, CI = NULL),
 result_lwr_f_90_94 = whoilo_paf_summary(result_f_90_94, result_f_90_941, sd_f_90_94, sd_f_90_941, CI = 0.025),
 result_upr_f_90_94 = whoilo_paf_summary(result_f_90_94, result_f_90_941, sd_f_90_94, sd_f_90_941, CI = 0.975),
 result_f_90_94 = whoilo_paf_summary(result_f_90_94, result_f_90_941, CI = NULL),
 result_lwr_f_95_ = whoilo_paf_summary(result_f_95_, result_f_95_1, sd_f_95_, sd_f_95_1, CI = 0.025),
 result_upr_f_95_ = whoilo_paf_summary(result_f_95_, result_f_95_1, sd_f_95_, sd_f_95_1, CI = 0.975),
 result_f_95_ = whoilo_paf_summary(result_f_95_, result_f_95_1, CI = NULL),
 result_lwr_m_15_19 = whoilo_paf_summary(result_m_15_19, result_m_15_191, sd_m_15_19, sd_m_15_191, CI = 0.025),
 result_upr_m_15_19 = whoilo_paf_summary(result_m_15_19, result_m_15_191, sd_m_15_19, sd_m_15_191, CI = 0.975),
 result_m_15_19 = whoilo_paf_summary(result_m_15_19, result_m_15_191, CI = NULL),
 result_lwr_m_20_24 = whoilo_paf_summary(result_m_20_24, result_m_20_241, sd_m_20_24, sd_m_20_241, CI = 0.025),
 result_upr_m_20_24 = whoilo_paf_summary(result_m_20_24, result_m_20_241, sd_m_20_24, sd_m_20_241, CI = 0.975),
 result_m_20_24 = whoilo_paf_summary(result_m_20_24, result_m_20_241, CI = NULL),
 result_lwr_m_25_29 = whoilo_paf_summary(result_m_25_29, result_m_25_291, sd_m_25_29, sd_m_25_291, CI = 0.025),
 result_upr_m_25_29 = whoilo_paf_summary(result_m_25_29, result_m_25_291, sd_m_25_29, sd_m_25_291, CI = 0.975),
 result_m_25_29 = whoilo_paf_summary(result_m_25_29, result_m_25_291, CI = NULL),
 result_lwr_m_30_34 = whoilo_paf_summary(result_m_30_34, result_m_30_341, sd_m_30_34, sd_m_30_341, CI = 0.025),
 result_upr_m_30_34 = whoilo_paf_summary(result_m_30_34, result_m_30_341, sd_m_30_34, sd_m_30_341, CI = 0.975),
 result_m_30_34 = whoilo_paf_summary(result_m_30_34, result_m_30_341, CI = NULL),
 result_lwr_m_35_39 = whoilo_paf_summary(result_m_35_39, result_m_35_391, sd_m_35_39, sd_m_35_391, CI = 0.025),
 result_upr_m_35_39 = whoilo_paf_summary(result_m_35_39, result_m_35_391, sd_m_35_39, sd_m_35_391, CI = 0.975),
 result_m_35_39 = whoilo_paf_summary(result_m_35_39, result_m_35_391, CI = NULL),
 result_lwr_m_40_44 = whoilo_paf_summary(result_m_40_44, result_m_40_441, sd_m_40_44, sd_m_40_441, CI = 0.025),
 result_upr_m_40_44 = whoilo_paf_summary(result_m_40_44, result_m_40_441, sd_m_40_44, sd_m_40_441, CI = 0.975),
 result_m_40_44 = whoilo_paf_summary(result_m_40_44, result_m_40_441, CI = NULL),
 result_lwr_m_45_49 = whoilo_paf_summary(result_m_45_49, result_m_45_491, sd_m_45_49, sd_m_45_491, CI = 0.025),
 result_upr_m_45_49 = whoilo_paf_summary(result_m_45_49, result_m_45_491, sd_m_45_49, sd_m_45_491, CI = 0.975),
 result_m_45_49 = whoilo_paf_summary(result_m_45_49, result_m_45_491, CI = NULL),
 result_lwr_m_50_54 = whoilo_paf_summary(result_m_50_54, result_m_50_541, sd_m_50_54, sd_m_50_541, CI = 0.025),
 result_upr_m_50_54 = whoilo_paf_summary(result_m_50_54, result_m_50_541, sd_m_50_54, sd_m_50_541, CI = 0.975),
 result_m_50_54 = whoilo_paf_summary(result_m_50_54, result_m_50_541, CI = NULL),
 result_lwr_m_55_59 = whoilo_paf_summary(result_m_55_59, result_m_55_591, sd_m_55_59, sd_m_55_591, CI = 0.025),
 result_upr_m_55_59 = whoilo_paf_summary(result_m_55_59, result_m_55_591, sd_m_55_59, sd_m_55_591, CI = 0.975),
 result_m_55_59 = whoilo_paf_summary(result_m_55_59, result_m_55_591, CI = NULL),
 result_lwr_m_60_64 = whoilo_paf_summary(result_m_60_64, result_m_60_641, sd_m_60_64, sd_m_60_641, CI = 0.025),
 result_upr_m_60_64 = whoilo_paf_summary(result_m_60_64, result_m_60_641, sd_m_60_64, sd_m_60_641, CI = 0.975),
 result_m_60_64 = whoilo_paf_summary(result_m_60_64, result_m_60_641, CI = NULL),
 result_lwr_m_65_69 = whoilo_paf_summary(result_m_65_69, result_m_65_691, sd_m_65_69, sd_m_65_691, CI = 0.025),
 result_upr_m_65_69 = whoilo_paf_summary(result_m_65_69, result_m_65_691, sd_m_65_69, sd_m_65_691, CI = 0.975),
 result_m_65_69 = whoilo_paf_summary(result_m_65_69, result_m_65_691, CI = NULL),
 result_lwr_m_70_74 = whoilo_paf_summary(result_m_70_74, result_m_70_741, sd_m_70_74, sd_m_70_741, CI = 0.025),
 result_upr_m_70_74 = whoilo_paf_summary(result_m_70_74, result_m_70_741, sd_m_70_74, sd_m_70_741, CI = 0.975),
 result_m_70_74 = whoilo_paf_summary(result_m_70_74, result_m_70_741, CI = NULL),
 result_lwr_m_75_79 = whoilo_paf_summary(result_m_75_79, result_m_75_791, sd_m_75_79, sd_m_75_791, CI = 0.025),
 result_upr_m_75_79 = whoilo_paf_summary(result_m_75_79, result_m_75_791, sd_m_75_79, sd_m_75_791, CI = 0.975),
 result_m_75_79 = whoilo_paf_summary(result_m_75_79, result_m_75_791, CI = NULL),
 result_lwr_m_80_84 = whoilo_paf_summary(result_m_80_84, result_m_80_841, sd_m_80_84, sd_m_80_841, CI = 0.025),
 result_upr_m_80_84 = whoilo_paf_summary(result_m_80_84, result_m_80_841, sd_m_80_84, sd_m_80_841, CI = 0.975),
 result_m_80_84 = whoilo_paf_summary(result_m_80_84, result_m_80_841, CI = NULL),
 result_lwr_m_85_89 = whoilo_paf_summary(result_m_85_89, result_m_85_891, sd_m_85_89, sd_m_85_891, CI = 0.025),
 result_upr_m_85_89 = whoilo_paf_summary(result_m_85_89, result_m_85_891, sd_m_85_89, sd_m_85_891, CI = 0.975),
 result_m_85_89 = whoilo_paf_summary(result_m_85_89, result_m_85_891, CI = NULL),
 result_lwr_m_90_94 = whoilo_paf_summary(result_m_90_94, result_m_90_941, sd_m_90_94, sd_m_90_941, CI = 0.025),
 result_upr_m_90_94 = whoilo_paf_summary(result_m_90_94, result_m_90_941, sd_m_90_94, sd_m_90_941, CI = 0.975),
 result_m_90_94 = whoilo_paf_summary(result_m_90_94, result_m_90_941, CI = NULL),
 result_lwr_m_95_ = whoilo_paf_summary(result_m_95_, result_m_95_1, sd_m_95_, sd_m_95_1, CI = 0.025),
 result_upr_m_95_ = whoilo_paf_summary(result_m_95_, result_m_95_1, sd_m_95_, sd_m_95_1, CI = 0.975),
 result_m_95_ = whoilo_paf_summary(result_m_95_, result_m_95_1, CI = NULL)) %>%
 ungroup() %>%
 mutate(sd_f_15_19 = (result_upr_f_15_19 - result_lwr_f_15_19)/2/1.96,
 sd_f_20_24 = (result_upr_f_20_24 - result_lwr_f_20_24)/2/1.96,
 sd_f_25_29 = (result_upr_f_25_29 - result_lwr_f_25_29)/2/1.96,
 sd_f_30_34 = (result_upr_f_30_34 - result_lwr_f_30_34)/2/1.96,
 sd_f_35_39 = (result_upr_f_35_39 - result_lwr_f_35_39)/2/1.96,
 sd_f_40_44 = (result_upr_f_40_44 - result_lwr_f_40_44)/2/1.96,
 sd_f_45_49 = (result_upr_f_45_49 - result_lwr_f_45_49)/2/1.96,
 sd_f_50_54 = (result_upr_f_50_54 - result_lwr_f_50_54)/2/1.96,
 sd_f_55_59 = (result_upr_f_55_59 - result_lwr_f_55_59)/2/1.96,
 sd_f_60_64 = (result_upr_f_60_64 - result_lwr_f_60_64)/2/1.96,
 sd_f_65_69 = (result_upr_f_65_69 - result_lwr_f_65_69)/2/1.96,
 sd_f_70_74 = (result_upr_f_70_74 - result_lwr_f_70_74)/2/1.96,
 sd_f_75_79 = (result_upr_f_75_79 - result_lwr_f_75_79)/2/1.96,
 sd_f_80_84 = (result_upr_f_80_84 - result_lwr_f_80_84)/2/1.96,
 sd_f_85_89 = (result_upr_f_85_89 - result_lwr_f_85_89)/2/1.96,
 sd_f_90_94 = (result_upr_f_90_94 - result_lwr_f_90_94)/2/1.96,
 sd_f_95_ = (result_upr_f_95_ - result_lwr_f_95_)/2/1.96,
 sd_m_15_19 = (result_upr_m_15_19 - result_lwr_m_15_19)/2/1.96,
 sd_m_20_24 = (result_upr_m_20_24 - result_lwr_m_20_24)/2/1.96,
 sd_m_25_29 = (result_upr_m_25_29 - result_lwr_m_25_29)/2/1.96,
 sd_m_30_34 = (result_upr_m_30_34 - result_lwr_m_30_34)/2/1.96,
 sd_m_35_39 = (result_upr_m_35_39 - result_lwr_m_35_39)/2/1.96,
 sd_m_40_44 = (result_upr_m_40_44 - result_lwr_m_40_44)/2/1.96,
 sd_m_45_49 = (result_upr_m_45_49 - result_lwr_m_45_49)/2/1.96,
 sd_m_50_54 = (result_upr_m_50_54 - result_lwr_m_50_54)/2/1.96,
 sd_m_55_59 = (result_upr_m_55_59 - result_lwr_m_55_59)/2/1.96,
 sd_m_60_64 = (result_upr_m_60_64 - result_lwr_m_60_64)/2/1.96,
 sd_m_65_69 = (result_upr_m_65_69 - result_lwr_m_65_69)/2/1.96,
 sd_m_70_74 = (result_upr_m_70_74 - result_lwr_m_70_74)/2/1.96,
 sd_m_75_79 = (result_upr_m_75_79 - result_lwr_m_75_79)/2/1.96,
 sd_m_80_84 = (result_upr_m_80_84 - result_lwr_m_80_84)/2/1.96,
 sd_m_85_89 = (result_upr_m_85_89 - result_lwr_m_85_89)/2/1.96,
 sd_m_90_94 = (result_upr_m_90_94 - result_lwr_m_90_94)/2/1.96,
 sd_m_95_ = (result_upr_m_95_ - result_lwr_m_95_)/2/1.96) %>%
 select(region,
 result_f_15_19, sd_f_15_19, result_lwr_f_15_19, result_upr_f_15_19,
 result_f_20_24, sd_f_20_24, result_lwr_f_20_24, result_upr_f_20_24,
 result_f_25_29, sd_f_25_29, result_lwr_f_25_29, result_upr_f_25_29,
 result_f_30_34, sd_f_30_34, result_lwr_f_30_34, result_upr_f_30_34,
 result_f_35_39, sd_f_35_39, result_lwr_f_35_39, result_upr_f_35_39,
 result_f_40_44, sd_f_40_44, result_lwr_f_40_44, result_upr_f_40_44,
 result_f_45_49, sd_f_45_49, result_lwr_f_45_49, result_upr_f_45_49,
 result_f_50_54, sd_f_50_54, result_lwr_f_50_54, result_upr_f_50_54,
 result_f_55_59, sd_f_55_59, result_lwr_f_55_59, result_upr_f_55_59,
 result_f_60_64, sd_f_60_64, result_lwr_f_60_64, result_upr_f_60_64,
 result_f_65_69, sd_f_65_69, result_lwr_f_65_69, result_upr_f_65_69,
 result_f_70_74, sd_f_70_74, result_lwr_f_70_74, result_upr_f_70_74,
 result_f_75_79, sd_f_75_79, result_lwr_f_75_79, result_upr_f_75_79,
 result_f_80_84, sd_f_80_84, result_lwr_f_80_84, result_upr_f_80_84,
 result_f_85_89, sd_f_85_89, result_lwr_f_85_89, result_upr_f_85_89,
 result_f_90_94, sd_f_90_94, result_lwr_f_90_94, result_upr_f_90_94,
 result_f_95_, sd_f_95_, result_lwr_f_95_, result_upr_f_95_,
 result_m_15_19, sd_m_15_19, result_lwr_m_15_19, result_upr_m_15_19,
 result_m_20_24, sd_m_20_24, result_lwr_m_20_24, result_upr_m_20_24,
 result_m_25_29, sd_m_25_29, result_lwr_m_25_29, result_upr_m_25_29,
 result_m_30_34, sd_m_30_34, result_lwr_m_30_34, result_upr_m_30_34,
 result_m_35_39, sd_m_35_39, result_lwr_m_35_39, result_upr_m_35_39,
 result_m_40_44, sd_m_40_44, result_lwr_m_40_44, result_upr_m_40_44,
 result_m_45_49, sd_m_45_49, result_lwr_m_45_49, result_upr_m_45_49,
 result_m_50_54, sd_m_50_54, result_lwr_m_50_54, result_upr_m_50_54,
 result_m_55_59, sd_m_55_59, result_lwr_m_55_59, result_upr_m_55_59,
 result_m_60_64, sd_m_60_64, result_lwr_m_60_64, result_upr_m_60_64,
 result_m_65_69, sd_m_65_69, result_lwr_m_65_69, result_upr_m_65_69,
 result_m_70_74, sd_m_70_74, result_lwr_m_70_74, result_upr_m_70_74,
 result_m_75_79, sd_m_75_79, result_lwr_m_75_79, result_upr_m_75_79,
 result_m_80_84, sd_m_80_84, result_lwr_m_80_84, result_upr_m_80_84,
 result_m_85_89, sd_m_85_89, result_lwr_m_85_89, result_upr_m_85_89,
 result_m_90_94, sd_m_90_94, result_lwr_m_90_94, result_upr_m_90_94,
 result_m_95_, sd_m_95_, result_lwr_m_95_, result_upr_m_95_)
 }
 if (outcome %in% c("DALYratio")){
 envelope_stroke_sexage_global <- df_in %>%
 filter(outcome == "DALYparent",
 year == !!year,
 cause == !!cause) %>%
 arrange(region, country_abbrev, year, sex, ilo_age_bands_5yr) %>%
 drop_na() %>%
 mutate(sd = (result_upr - result_lwr)/2/1.96) %>%
 group_by(region, sex, ilo_age_bands_5yr) %>%
 summarise(result = sum(result),
 sd = sqrt(sum((sd)^2))) %>%
 ungroup %>%
 pivot_wider(names_from = sex:ilo_age_bands_5yr, values_from = result:sd)

 dths_stroke_sexage_global <- df_in %>%
 filter(outcome == "DALYenvelope",
 year == !!year,
 cause == !!cause) %>%
 mutate(result_lwr = case_when(is.na(result_lwr) ~ result,
 TRUE ~ result_lwr),
 result_upr = case_when(is.na(result_upr) ~ result,
 TRUE ~ result_upr)) %>%
 arrange(region, country_abbrev, year, sex, ilo_age_bands_5yr) %>%
 drop_na() %>%
 mutate(sd = (result_upr - result_lwr)/2/1.96) %>%
 group_by(region, sex, ilo_age_bands_5yr) %>%
 summarise(result = sum(result),
 sd = sqrt(sum((sd)^2))) %>%
 ungroup %>%
 pivot_wider(names_from = sex:ilo_age_bands_5yr, values_from = result:sd) %>%

 select_all(.funs = funs(str_c(., "1")))

 dths_stroke_sexage_global <- bind_cols(dths_stroke_sexage_global, envelope_stroke_sexage_global) %>%
 rowwise() %>%
 transmute(region = region,
 result_lwr_f_15_19 = whoilo_paf_summary(result_f_15_19, result_f_15_191, sd_f_15_19, sd_f_15_191, CI = 0.025),
 result_upr_f_15_19 = whoilo_paf_summary(result_f_15_19, result_f_15_191, sd_f_15_19, sd_f_15_191, CI = 0.975),
 result_f_15_19 = whoilo_paf_summary(result_f_15_19, result_f_15_191, CI = NULL),
 result_lwr_f_20_24 = whoilo_paf_summary(result_f_20_24, result_f_20_241, sd_f_20_24, sd_f_20_241, CI = 0.025),
 result_upr_f_20_24 = whoilo_paf_summary(result_f_20_24, result_f_20_241, sd_f_20_24, sd_f_20_241, CI = 0.975),
 result_f_20_24 = whoilo_paf_summary(result_f_20_24, result_f_20_241, CI = NULL),
 result_lwr_f_25_29 = whoilo_paf_summary(result_f_25_29, result_f_25_291, sd_f_25_29, sd_f_25_291, CI = 0.025),
 result_upr_f_25_29 = whoilo_paf_summary(result_f_25_29, result_f_25_291, sd_f_25_29, sd_f_25_291, CI = 0.975),
 result_f_25_29 = whoilo_paf_summary(result_f_25_29, result_f_25_291, CI = NULL),
 result_lwr_f_30_34 = whoilo_paf_summary(result_f_30_34, result_f_30_341, sd_f_30_34, sd_f_30_341, CI = 0.025),
 result_upr_f_30_34 = whoilo_paf_summary(result_f_30_34, result_f_30_341, sd_f_30_34, sd_f_30_341, CI = 0.975),
 result_f_30_34 = whoilo_paf_summary(result_f_30_34, result_f_30_341, CI = NULL),
 result_lwr_f_35_39 = whoilo_paf_summary(result_f_35_39, result_f_35_391, sd_f_35_39, sd_f_35_391, CI = 0.025),
 result_upr_f_35_39 = whoilo_paf_summary(result_f_35_39, result_f_35_391, sd_f_35_39, sd_f_35_391, CI = 0.975),
 result_f_35_39 = whoilo_paf_summary(result_f_35_39, result_f_35_391, CI = NULL),
 result_lwr_f_40_44 = whoilo_paf_summary(result_f_40_44, result_f_40_441, sd_f_40_44, sd_f_40_441, CI = 0.025),
 result_upr_f_40_44 = whoilo_paf_summary(result_f_40_44, result_f_40_441, sd_f_40_44, sd_f_40_441, CI = 0.975),
 result_f_40_44 = whoilo_paf_summary(result_f_40_44, result_f_40_441, CI = NULL),
 result_lwr_f_45_49 = whoilo_paf_summary(result_f_45_49, result_f_45_491, sd_f_45_49, sd_f_45_491, CI = 0.025),
 result_upr_f_45_49 = whoilo_paf_summary(result_f_45_49, result_f_45_491, sd_f_45_49, sd_f_45_491, CI = 0.975),
 result_f_45_49 = whoilo_paf_summary(result_f_45_49, result_f_45_491, CI = NULL),
 result_lwr_f_50_54 = whoilo_paf_summary(result_f_50_54, result_f_50_541, sd_f_50_54, sd_f_50_541, CI = 0.025),
 result_upr_f_50_54 = whoilo_paf_summary(result_f_50_54, result_f_50_541, sd_f_50_54, sd_f_50_541, CI = 0.975),
 result_f_50_54 = whoilo_paf_summary(result_f_50_54, result_f_50_541, CI = NULL),
 result_lwr_f_55_59 = whoilo_paf_summary(result_f_55_59, result_f_55_591, sd_f_55_59, sd_f_55_591, CI = 0.025),
 result_upr_f_55_59 = whoilo_paf_summary(result_f_55_59, result_f_55_591, sd_f_55_59, sd_f_55_591, CI = 0.975),
 result_f_55_59 = whoilo_paf_summary(result_f_55_59, result_f_55_591, CI = NULL),
 result_lwr_f_60_64 = whoilo_paf_summary(result_f_60_64, result_f_60_641, sd_f_60_64, sd_f_60_641, CI = 0.025),
 result_upr_f_60_64 = whoilo_paf_summary(result_f_60_64, result_f_60_641, sd_f_60_64, sd_f_60_641, CI = 0.975),
 result_f_60_64 = whoilo_paf_summary(result_f_60_64, result_f_60_641, CI = NULL),
 result_lwr_f_65_69 = whoilo_paf_summary(result_f_65_69, result_f_65_691, sd_f_65_69, sd_f_65_691, CI = 0.025),
 result_upr_f_65_69 = whoilo_paf_summary(result_f_65_69, result_f_65_691, sd_f_65_69, sd_f_65_691, CI = 0.975),
 result_f_65_69 = whoilo_paf_summary(result_f_65_69, result_f_65_691, CI = NULL),
 result_lwr_f_70_74 = whoilo_paf_summary(result_f_70_74, result_f_70_741, sd_f_70_74, sd_f_70_741, CI = 0.025),
 result_upr_f_70_74 = whoilo_paf_summary(result_f_70_74, result_f_70_741, sd_f_70_74, sd_f_70_741, CI = 0.975),
 result_f_70_74 = whoilo_paf_summary(result_f_70_74, result_f_70_741, CI = NULL),
 result_lwr_f_75_79 = whoilo_paf_summary(result_f_75_79, result_f_75_791, sd_f_75_79, sd_f_75_791, CI = 0.025),
 result_upr_f_75_79 = whoilo_paf_summary(result_f_75_79, result_f_75_791, sd_f_75_79, sd_f_75_791, CI = 0.975),
 result_f_75_79 = whoilo_paf_summary(result_f_75_79, result_f_75_791, CI = NULL),
 result_lwr_f_80_84 = whoilo_paf_summary(result_f_80_84, result_f_80_841, sd_f_80_84, sd_f_80_841, CI = 0.025),
 result_upr_f_80_84 = whoilo_paf_summary(result_f_80_84, result_f_80_841, sd_f_80_84, sd_f_80_841, CI = 0.975),
 result_f_80_84 = whoilo_paf_summary(result_f_80_84, result_f_80_841, CI = NULL),
 result_lwr_f_85_89 = whoilo_paf_summary(result_f_85_89, result_f_85_891, sd_f_85_89, sd_f_85_891, CI = 0.025),
 result_upr_f_85_89 = whoilo_paf_summary(result_f_85_89, result_f_85_891, sd_f_85_89, sd_f_85_891, CI = 0.975),
 result_f_85_89 = whoilo_paf_summary(result_f_85_89, result_f_85_891, CI = NULL),
 result_lwr_f_90_94 = whoilo_paf_summary(result_f_90_94, result_f_90_941, sd_f_90_94, sd_f_90_941, CI = 0.025),
 result_upr_f_90_94 = whoilo_paf_summary(result_f_90_94, result_f_90_941, sd_f_90_94, sd_f_90_941, CI = 0.975),
 result_f_90_94 = whoilo_paf_summary(result_f_90_94, result_f_90_941, CI = NULL),
 result_lwr_f_95_ = whoilo_paf_summary(result_f_95_, result_f_95_1, sd_f_95_, sd_f_95_1, CI = 0.025),
 result_upr_f_95_ = whoilo_paf_summary(result_f_95_, result_f_95_1, sd_f_95_, sd_f_95_1, CI = 0.975),
 result_f_95_ = whoilo_paf_summary(result_f_95_, result_f_95_1, CI = NULL),
 result_lwr_m_15_19 = whoilo_paf_summary(result_m_15_19, result_m_15_191, sd_m_15_19, sd_m_15_191, CI = 0.025),
 result_upr_m_15_19 = whoilo_paf_summary(result_m_15_19, result_m_15_191, sd_m_15_19, sd_m_15_191, CI = 0.975),
 result_m_15_19 = whoilo_paf_summary(result_m_15_19, result_m_15_191, CI = NULL),
 result_lwr_m_20_24 = whoilo_paf_summary(result_m_20_24, result_m_20_241, sd_m_20_24, sd_m_20_241, CI = 0.025),
 result_upr_m_20_24 = whoilo_paf_summary(result_m_20_24, result_m_20_241, sd_m_20_24, sd_m_20_241, CI = 0.975),
 result_m_20_24 = whoilo_paf_summary(result_m_20_24, result_m_20_241, CI = NULL),
 result_lwr_m_25_29 = whoilo_paf_summary(result_m_25_29, result_m_25_291, sd_m_25_29, sd_m_25_291, CI = 0.025),
 result_upr_m_25_29 = whoilo_paf_summary(result_m_25_29, result_m_25_291, sd_m_25_29, sd_m_25_291, CI = 0.975),
 result_m_25_29 = whoilo_paf_summary(result_m_25_29, result_m_25_291, CI = NULL),
 result_lwr_m_30_34 = whoilo_paf_summary(result_m_30_34, result_m_30_341, sd_m_30_34, sd_m_30_341, CI = 0.025),
 result_upr_m_30_34 = whoilo_paf_summary(result_m_30_34, result_m_30_341, sd_m_30_34, sd_m_30_341, CI = 0.975),
 result_m_30_34 = whoilo_paf_summary(result_m_30_34, result_m_30_341, CI = NULL),
 result_lwr_m_35_39 = whoilo_paf_summary(result_m_35_39, result_m_35_391, sd_m_35_39, sd_m_35_391, CI = 0.025),
 result_upr_m_35_39 = whoilo_paf_summary(result_m_35_39, result_m_35_391, sd_m_35_39, sd_m_35_391, CI = 0.975),
 result_m_35_39 = whoilo_paf_summary(result_m_35_39, result_m_35_391, CI = NULL),
 result_lwr_m_40_44 = whoilo_paf_summary(result_m_40_44, result_m_40_441, sd_m_40_44, sd_m_40_441, CI = 0.025),
 result_upr_m_40_44 = whoilo_paf_summary(result_m_40_44, result_m_40_441, sd_m_40_44, sd_m_40_441, CI = 0.975),
 result_m_40_44 = whoilo_paf_summary(result_m_40_44, result_m_40_441, CI = NULL),
 result_lwr_m_45_49 = whoilo_paf_summary(result_m_45_49, result_m_45_491, sd_m_45_49, sd_m_45_491, CI = 0.025),
 result_upr_m_45_49 = whoilo_paf_summary(result_m_45_49, result_m_45_491, sd_m_45_49, sd_m_45_491, CI = 0.975),
 result_m_45_49 = whoilo_paf_summary(result_m_45_49, result_m_45_491, CI = NULL),
 result_lwr_m_50_54 = whoilo_paf_summary(result_m_50_54, result_m_50_541, sd_m_50_54, sd_m_50_541, CI = 0.025),
 result_upr_m_50_54 = whoilo_paf_summary(result_m_50_54, result_m_50_541, sd_m_50_54, sd_m_50_541, CI = 0.975),
 result_m_50_54 = whoilo_paf_summary(result_m_50_54, result_m_50_541, CI = NULL),
 result_lwr_m_55_59 = whoilo_paf_summary(result_m_55_59, result_m_55_591, sd_m_55_59, sd_m_55_591, CI = 0.025),
 result_upr_m_55_59 = whoilo_paf_summary(result_m_55_59, result_m_55_591, sd_m_55_59, sd_m_55_591, CI = 0.975),
 result_m_55_59 = whoilo_paf_summary(result_m_55_59, result_m_55_591, CI = NULL),
 result_lwr_m_60_64 = whoilo_paf_summary(result_m_60_64, result_m_60_641, sd_m_60_64, sd_m_60_641, CI = 0.025),
 result_upr_m_60_64 = whoilo_paf_summary(result_m_60_64, result_m_60_641, sd_m_60_64, sd_m_60_641, CI = 0.975),
 result_m_60_64 = whoilo_paf_summary(result_m_60_64, result_m_60_641, CI = NULL),
 result_lwr_m_65_69 = whoilo_paf_summary(result_m_65_69, result_m_65_691, sd_m_65_69, sd_m_65_691, CI = 0.025),
 result_upr_m_65_69 = whoilo_paf_summary(result_m_65_69, result_m_65_691, sd_m_65_69, sd_m_65_691, CI = 0.975),
 result_m_65_69 = whoilo_paf_summary(result_m_65_69, result_m_65_691, CI = NULL),
 result_lwr_m_70_74 = whoilo_paf_summary(result_m_70_74, result_m_70_741, sd_m_70_74, sd_m_70_741, CI = 0.025),
 result_upr_m_70_74 = whoilo_paf_summary(result_m_70_74, result_m_70_741, sd_m_70_74, sd_m_70_741, CI = 0.975),
 result_m_70_74 = whoilo_paf_summary(result_m_70_74, result_m_70_741, CI = NULL),
 result_lwr_m_75_79 = whoilo_paf_summary(result_m_75_79, result_m_75_791, sd_m_75_79, sd_m_75_791, CI = 0.025),
 result_upr_m_75_79 = whoilo_paf_summary(result_m_75_79, result_m_75_791, sd_m_75_79, sd_m_75_791, CI = 0.975),
 result_m_75_79 = whoilo_paf_summary(result_m_75_79, result_m_75_791, CI = NULL),
 result_lwr_m_80_84 = whoilo_paf_summary(result_m_80_84, result_m_80_841, sd_m_80_84, sd_m_80_841, CI = 0.025),
 result_upr_m_80_84 = whoilo_paf_summary(result_m_80_84, result_m_80_841, sd_m_80_84, sd_m_80_841, CI = 0.975),
 result_m_80_84 = whoilo_paf_summary(result_m_80_84, result_m_80_841, CI = NULL),
 result_lwr_m_85_89 = whoilo_paf_summary(result_m_85_89, result_m_85_891, sd_m_85_89, sd_m_85_891, CI = 0.025),
 result_upr_m_85_89 = whoilo_paf_summary(result_m_85_89, result_m_85_891, sd_m_85_89, sd_m_85_891, CI = 0.975),
 result_m_85_89 = whoilo_paf_summary(result_m_85_89, result_m_85_891, CI = NULL),
 result_lwr_m_90_94 = whoilo_paf_summary(result_m_90_94, result_m_90_941, sd_m_90_94, sd_m_90_941, CI = 0.025),
 result_upr_m_90_94 = whoilo_paf_summary(result_m_90_94, result_m_90_941, sd_m_90_94, sd_m_90_941, CI = 0.975),
 result_m_90_94 = whoilo_paf_summary(result_m_90_94, result_m_90_941, CI = NULL),
 result_lwr_m_95_ = whoilo_paf_summary(result_m_95_, result_m_95_1, sd_m_95_, sd_m_95_1, CI = 0.025),
 result_upr_m_95_ = whoilo_paf_summary(result_m_95_, result_m_95_1, sd_m_95_, sd_m_95_1, CI = 0.975),
 result_m_95_ = whoilo_paf_summary(result_m_95_, result_m_95_1, CI = NULL)) %>%
 ungroup() %>%
 mutate(sd_f_15_19 = (result_upr_f_15_19 - result_lwr_f_15_19)/2/1.96,
 sd_f_20_24 = (result_upr_f_20_24 - result_lwr_f_20_24)/2/1.96,
 sd_f_25_29 = (result_upr_f_25_29 - result_lwr_f_25_29)/2/1.96,
 sd_f_30_34 = (result_upr_f_30_34 - result_lwr_f_30_34)/2/1.96,
 sd_f_35_39 = (result_upr_f_35_39 - result_lwr_f_35_39)/2/1.96,
 sd_f_40_44 = (result_upr_f_40_44 - result_lwr_f_40_44)/2/1.96,
 sd_f_45_49 = (result_upr_f_45_49 - result_lwr_f_45_49)/2/1.96,
 sd_f_50_54 = (result_upr_f_50_54 - result_lwr_f_50_54)/2/1.96,
 sd_f_55_59 = (result_upr_f_55_59 - result_lwr_f_55_59)/2/1.96,
 sd_f_60_64 = (result_upr_f_60_64 - result_lwr_f_60_64)/2/1.96,
 sd_f_65_69 = (result_upr_f_65_69 - result_lwr_f_65_69)/2/1.96,
 sd_f_70_74 = (result_upr_f_70_74 - result_lwr_f_70_74)/2/1.96,
 sd_f_75_79 = (result_upr_f_75_79 - result_lwr_f_75_79)/2/1.96,
 sd_f_80_84 = (result_upr_f_80_84 - result_lwr_f_80_84)/2/1.96,
 sd_f_85_89 = (result_upr_f_85_89 - result_lwr_f_85_89)/2/1.96,
 sd_f_90_94 = (result_upr_f_90_94 - result_lwr_f_90_94)/2/1.96,
 sd_f_95_ = (result_upr_f_95_ - result_lwr_f_95_)/2/1.96,
 sd_m_15_19 = (result_upr_m_15_19 - result_lwr_m_15_19)/2/1.96,
 sd_m_20_24 = (result_upr_m_20_24 - result_lwr_m_20_24)/2/1.96,
 sd_m_25_29 = (result_upr_m_25_29 - result_lwr_m_25_29)/2/1.96,
 sd_m_30_34 = (result_upr_m_30_34 - result_lwr_m_30_34)/2/1.96,
 sd_m_35_39 = (result_upr_m_35_39 - result_lwr_m_35_39)/2/1.96,
 sd_m_40_44 = (result_upr_m_40_44 - result_lwr_m_40_44)/2/1.96,
 sd_m_45_49 = (result_upr_m_45_49 - result_lwr_m_45_49)/2/1.96,
 sd_m_50_54 = (result_upr_m_50_54 - result_lwr_m_50_54)/2/1.96,
 sd_m_55_59 = (result_upr_m_55_59 - result_lwr_m_55_59)/2/1.96,
 sd_m_60_64 = (result_upr_m_60_64 - result_lwr_m_60_64)/2/1.96,
 sd_m_65_69 = (result_upr_m_65_69 - result_lwr_m_65_69)/2/1.96,
 sd_m_70_74 = (result_upr_m_70_74 - result_lwr_m_70_74)/2/1.96,
 sd_m_75_79 = (result_upr_m_75_79 - result_lwr_m_75_79)/2/1.96,
 sd_m_80_84 = (result_upr_m_80_84 - result_lwr_m_80_84)/2/1.96,
 sd_m_85_89 = (result_upr_m_85_89 - result_lwr_m_85_89)/2/1.96,
 sd_m_90_94 = (result_upr_m_90_94 - result_lwr_m_90_94)/2/1.96,
 sd_m_95_ = (result_upr_m_95_ - result_lwr_m_95_)/2/1.96) %>%
 select(region,
 result_f_15_19, sd_f_15_19, result_lwr_f_15_19, result_upr_f_15_19,
 result_f_20_24, sd_f_20_24, result_lwr_f_20_24, result_upr_f_20_24,
 result_f_25_29, sd_f_25_29, result_lwr_f_25_29, result_upr_f_25_29,
 result_f_30_34, sd_f_30_34, result_lwr_f_30_34, result_upr_f_30_34,
 result_f_35_39, sd_f_35_39, result_lwr_f_35_39, result_upr_f_35_39,
 result_f_40_44, sd_f_40_44, result_lwr_f_40_44, result_upr_f_40_44,
 result_f_45_49, sd_f_45_49, result_lwr_f_45_49, result_upr_f_45_49,
 result_f_50_54, sd_f_50_54, result_lwr_f_50_54, result_upr_f_50_54,
 result_f_55_59, sd_f_55_59, result_lwr_f_55_59, result_upr_f_55_59,
 result_f_60_64, sd_f_60_64, result_lwr_f_60_64, result_upr_f_60_64,
 result_f_65_69, sd_f_65_69, result_lwr_f_65_69, result_upr_f_65_69,
 result_f_70_74, sd_f_70_74, result_lwr_f_70_74, result_upr_f_70_74,
 result_f_75_79, sd_f_75_79, result_lwr_f_75_79, result_upr_f_75_79,
 result_f_80_84, sd_f_80_84, result_lwr_f_80_84, result_upr_f_80_84,
 result_f_85_89, sd_f_85_89, result_lwr_f_85_89, result_upr_f_85_89,
 result_f_90_94, sd_f_90_94, result_lwr_f_90_94, result_upr_f_90_94,
 result_f_95_, sd_f_95_, result_lwr_f_95_, result_upr_f_95_,
 result_m_15_19, sd_m_15_19, result_lwr_m_15_19, result_upr_m_15_19,
 result_m_20_24, sd_m_20_24, result_lwr_m_20_24, result_upr_m_20_24,
 result_m_25_29, sd_m_25_29, result_lwr_m_25_29, result_upr_m_25_29,
 result_m_30_34, sd_m_30_34, result_lwr_m_30_34, result_upr_m_30_34,
 result_m_35_39, sd_m_35_39, result_lwr_m_35_39, result_upr_m_35_39,
 result_m_40_44, sd_m_40_44, result_lwr_m_40_44, result_upr_m_40_44,
 result_m_45_49, sd_m_45_49, result_lwr_m_45_49, result_upr_m_45_49,
 result_m_50_54, sd_m_50_54, result_lwr_m_50_54, result_upr_m_50_54,
 result_m_55_59, sd_m_55_59, result_lwr_m_55_59, result_upr_m_55_59,
 result_m_60_64, sd_m_60_64, result_lwr_m_60_64, result_upr_m_60_64,
 result_m_65_69, sd_m_65_69, result_lwr_m_65_69, result_upr_m_65_69,
 result_m_70_74, sd_m_70_74, result_lwr_m_70_74, result_upr_m_70_74,
 result_m_75_79, sd_m_75_79, result_lwr_m_75_79, result_upr_m_75_79,
 result_m_80_84, sd_m_80_84, result_lwr_m_80_84, result_upr_m_80_84,
 result_m_85_89, sd_m_85_89, result_lwr_m_85_89, result_upr_m_85_89,
 result_m_90_94, sd_m_90_94, result_lwr_m_90_94, result_upr_m_90_94,
 result_m_95_, sd_m_95_, result_lwr_m_95_, result_upr_m_95_)
 }
 }
 # combine them all
 df_out <- tibble(region = character())
 if ("total" %in% breakdown){
 df_out <- df_out %>%
 full_join(dths_stroke_global)
 }
 if ("sex" %in% breakdown){
 df_out <- df_out %>%
 full_join(dths_stroke_sex_global)
 }
 if ("age" %in% breakdown){
 df_out <- df_out %>%
 full_join(dths_stroke_age_global)
 }
 if ("sexage" %in% breakdown){
 df_out <- df_out %>%
 full_join(dths_stroke_sexage_global)
 }
 # return the final output
 df_out <- df_out %>%
 mutate(year = year) %>%
 select(year, everything())
 tmp <- df_out %>%
 select(contains("region"))
 tmp <- names(tmp)
 if (length(tmp) >1) {
 tmp <- tmp[2:length(tmp)]
 df_out <- df_out %>%
 select(-contains(tmp))
 }
 return(df_out)
}
my_func_national <- function(df_in,
 year,
 outcome,
 cause,
 breakdown){
 # total
 if ("total" %in% breakdown){
 if (outcome %in% c("PAF", "DTHpaf")){
 envelope_stroke_region <- df_in %>%
 filter(outcome == "DTHenvelope",
 year == !!year,
 cause == !!cause) %>%
 arrange(region, country_abbrev, year, sex, ilo_age_bands_5yr) %>%
 drop_na() %>%
 group_by(region, country_abbrev) %>%
 mutate(sd = (result_upr - result_lwr)/2/1.96) %>%
 summarise(result = sum(result),
 sd = sqrt(sum((sd)^2))) %>%
 mutate(result_lwr = qnorm(p = 0.025, mean = result, sd = sd),
 result_upr = qnorm(p = 0.975, mean = result, sd = sd)) %>%
 ungroup %>%

 select_all(.funs = funs(str_c(., "1")))

 dths_stroke_global <- df_in %>%
 filter(outcome == "DTH",
 year == !!year,
 cause == !!cause) %>%
 arrange(region, country_abbrev, year, sex, ilo_age_bands_5yr) %>%
 drop_na() %>%
 group_by(region, country_abbrev) %>%
 mutate(sd = (result_upr - result_lwr)/2/1.96) %>%
 summarise(result = sum(result),
 sd = sqrt(sum((sd)^2))) %>%
 mutate(result_lwr = qnorm(p = 0.025, mean = result, sd = sd),
 result_upr = qnorm(p = 0.975, mean = result, sd = sd)) %>%
 ungroup
 dths_stroke_global <- bind_cols(dths_stroke_global, envelope_stroke_region) %>%
 rowwise() %>%
 transmute(region = region,
 country_abbrev = country_abbrev,
 result_b = whoilo_paf_summary(result, result1, CI = NULL),
 result_lwr_b = whoilo_paf_summary(result, result1, sd, sd1, CI = 0.025),
 result_upr_b = whoilo_paf_summary(result, result1, sd, sd1, CI = 0.975)) %>%
 mutate(sd_b = (result_upr_b - result_lwr_b)/2/1.96) %>%
 ungroup() %>%
 select(region, country_abbrev, result_b, sd_b, result_lwr_b, result_upr_b)
 }
 if (outcome %in% c("DALYpaf")){
 envelope_stroke_region <- df_in %>%
 filter(outcome == "DALYenvelope",
 year == !!year,
 cause == !!cause) %>%
 mutate(result_lwr = case_when(is.na(result_lwr) ~ result,
 TRUE ~ result_lwr),
 result_upr = case_when(is.na(result_upr) ~ result,
 TRUE ~ result_upr)) %>%
 arrange(region, country_abbrev, year, sex, ilo_age_bands_5yr) %>%
 drop_na() %>%
 group_by(region, country_abbrev) %>%
 mutate(sd = (result_upr - result_lwr)/2/1.96) %>%
 summarise(result = sum(result),
 sd = sqrt(sum((sd)^2))) %>%
 mutate(result_lwr = qnorm(p = 0.025, mean = result, sd = sd),
 result_upr = qnorm(p = 0.975, mean = result, sd = sd)) %>%
 ungroup %>%

 select_all(.funs = funs(str_c(., "1")))

 dths_stroke_global <- df_in %>%
 filter(outcome == "DALY",
 year == !!year,
 cause == !!cause) %>%
 arrange(region, country_abbrev, year, sex, ilo_age_bands_5yr) %>%
 drop_na() %>%
 group_by(region, country_abbrev) %>%
 mutate(sd = (result_upr - result_lwr)/2/1.96) %>%
 summarise(result = sum(result),
 sd = sqrt(sum((sd)^2))) %>%
 mutate(result_lwr = qnorm(p = 0.025, mean = result, sd = sd),
 result_upr = qnorm(p = 0.975, mean = result, sd = sd)) %>%
 ungroup
 dths_stroke_global <- bind_cols(dths_stroke_global, envelope_stroke_region) %>%
 rowwise() %>%
 transmute(region = region,
 country_abbrev = country_abbrev,
 result_b = whoilo_paf_summary(result, result1, CI = NULL),
 result_lwr_b = whoilo_paf_summary(result, result1, sd, sd1, CI = 0.025),
 result_upr_b = whoilo_paf_summary(result, result1, sd, sd1, CI = 0.975)) %>%
 mutate(sd_b = (result_upr_b - result_lwr_b)/2/1.96) %>%
 ungroup() %>%
 select(region, country_abbrev, result_b, sd_b, result_lwr_b, result_upr_b)
 }
 if (outcome %in% c("h0", "h1", "h2", "h3", "h4", "h5")){
 dths_stroke_global <- df_in %>%
 filter(outcome == !!outcome,
 year == !!year,
 cause == !!cause) %>%
 arrange(region, country_abbrev, year, sex, ilo_age_bands_5yr) %>%
 mutate(sd = (result_upr - result_lwr)/2/1.96) %>%
 left_join(who_ilo_long_populations, by = c("region", "country_abbrev", "year", "sex", "ilo_age_bands_5yr" = "age_bands_5yr")) %>%
 mutate(result = result*population,
 sd = sd*population) %>%
 drop_na() %>%
 group_by(region, country_abbrev) %>%
 summarise(result = sum(result),
 sd = sqrt(sum((sd)^2)),
 population = sum(population)) %>%
 mutate(result_lwr = qnorm(p = 0.025, mean = result, sd = sd)/ population,
 result_upr = qnorm(p = 0.975, mean = result, sd = sd)/ population,
 result = result / population,
 sd = sd/population) %>%
 select(-population) %>%
 ungroup %>%
 mutate(sex = "b") %>%
 pivot_wider(names_from = sex, values_from = result:result_upr) %>%
 select(region, country_abbrev, result_b, sd_b, result_lwr_b, result_upr_b)
 dths_stroke_global[dths_stroke_global < 0] <- 0
 }
 if (outcome %in% c("h0_number", "h1_number", "h2_number", "h3_number", "h4_number", "h5_number")){
 outcome2 <- str_sub(outcome, end = -8L)
 dths_stroke_global <- df_in %>%
 filter(outcome == !!outcome2,
 year == !!year,
 cause == !!cause) %>%
 arrange(region, country_abbrev, year, sex, ilo_age_bands_5yr) %>%
 mutate(sd = (result_upr - result_lwr)/2/1.96) %>%
 left_join(who_ilo_long_populations, by = c("region", "country_abbrev", "year", "sex", "ilo_age_bands_5yr" = "age_bands_5yr")) %>%
 mutate(population = population * 1000) %>%
 mutate(result = result*population,
 sd = sd*population) %>%
 drop_na() %>%
 group_by(region, country_abbrev) %>%
 summarise(result = sum(result),
 sd = sqrt(sum((sd)^2)),
 population = sum(population)) %>%
 mutate(result_lwr = qnorm(p = 0.025, mean = result, sd = sd),
 result_upr = qnorm(p = 0.975, mean = result, sd = sd),
 result = result,
 sd = sd) %>%
 select(-population) %>%
 ungroup %>%
 mutate(sex = "b") %>%
 pivot_wider(names_from = sex, values_from = result:result_upr) %>%
 select(region, country_abbrev, result_b, sd_b, result_lwr_b, result_upr_b)
 dths_stroke_global[dths_stroke_global < 0] <- 0
 }
 if (outcome %in% c("DTH", "DALY", "DTHenvelope", "DALYenvelope")) {
 dths_stroke_global <- df_in %>%
 filter(outcome == !!outcome,
 year == !!year,
 cause == !!cause) %>%
 arrange(region, country_abbrev, year, sex, ilo_age_bands_5yr) %>%
 mutate(sd = (result_upr - result_lwr)/2/1.96) %>%
 drop_na(region, result) %>%
 group_by(region, country_abbrev) %>%
 summarise(result = sum(result),
 sd = sqrt(sum((sd)^2))) %>%
 mutate(result_lwr = qnorm(p = 0.025, mean = result, sd = sd),
 result_upr = qnorm(p = 0.975, mean = result, sd = sd)) %>%
 ungroup %>%
 mutate(sex = "b") %>%
 pivot_wider(names_from = sex, values_from = result:result_upr) %>%
 select(region, country_abbrev, result_b, sd_b, result_lwr_b, result_upr_b)
 }
 if (outcome %in% c("DTHS", "DALYS")) {
 outcome2 <- str_sub(outcome, end = -2L)
 dths_stroke_global <- df_in %>%
 filter(outcome == !!outcome2,
 year == !!year,
 cause %in% c("stroke_h5", "ihd_h5")) %>%
 arrange(region, country_abbrev, year, sex, ilo_age_bands_5yr) %>%
 mutate(sd = (result_upr - result_lwr)/2/1.96) %>%
 drop_na() %>%
 group_by(region, country_abbrev) %>%
 summarise(result = sum(result),
 sd = sqrt(sum((sd)^2))) %>%
 mutate(result_lwr = qnorm(p = 0.025, mean = result, sd = sd),
 result_upr = qnorm(p = 0.975, mean = result, sd = sd)) %>%
 ungroup %>%
 mutate(sex = "b") %>%
 pivot_wider(names_from = sex, values_from = result:result_upr) %>%
 select(region, country_abbrev, result_b, sd_b, result_lwr_b, result_upr_b)
 }
 if (outcome %in% c("h45")){
 dths_stroke_global <- df_in %>%
 filter(outcome %in% c("h4", "h5"),
 year == !!year,
 cause == !!cause) %>%
 arrange(region, country_abbrev, year, sex, ilo_age_bands_5yr) %>%
 mutate(sd = (result_upr - result_lwr)/2/1.96) %>%
 left_join(who_ilo_long_populations, by = c("region", "country_abbrev", "year", "sex", "ilo_age_bands_5yr" = "age_bands_5yr")) %>%
 mutate(result = result*population,
 sd = sd*population) %>%
 drop_na() %>%
 group_by(region, country_abbrev) %>%
 summarise(result = sum(result),
 sd = sqrt(sum((sd)^2)),
 population = sum(population)/2) %>%
 mutate(result_lwr = qnorm(p = 0.025, mean = result, sd = sd)/ population,
 result_upr = qnorm(p = 0.975, mean = result, sd = sd)/ population,
 result = result / population,
 sd = sd/population) %>%
 select(-population) %>%
 ungroup %>%
 mutate(sex = "b") %>%
 pivot_wider(names_from = sex, values_from = result:result_upr) %>%
 select(region, country_abbrev, result_b, sd_b, result_lwr_b, result_upr_b)
 dths_stroke_global[dths_stroke_global < 0] <- 0
 }
 if (outcome %in% c("DTHrate")){
 outcome2 <- case_when(outcome == "DTHrate" ~ "DTH",
 outcome == "DALYrate" ~ "DALY",
 TRUE ~ NA_character_)
 dths_stroke_global <- df_in %>%
 filter(outcome == !!outcome2,
 year == !!year,
 cause == !!cause) %>%
 arrange(region, country_abbrev, year, sex, ilo_age_bands_5yr) %>%
 mutate(sd = (result_upr - result_lwr)/2/1.96) %>%
 left_join(who_ilo_long_populations, by = c("region", "country_abbrev", "year", "sex", "ilo_age_bands_5yr" = "age_bands_5yr")) %>%
 drop_na() %>%
 group_by(region, country_abbrev) %>%
 summarise(result = sum(result),
 sd = sqrt(sum((sd)^2)),
 population = sum(population)) %>%
 mutate(population = population / 100,
 result = result / population,
 sd = sd / population,
 result_lwr = qnorm(p = 0.025, mean = result, sd = sd),
 result_upr = qnorm(p = 0.975, mean = result, sd = sd)) %>%
 select(-population) %>%
 ungroup %>%
 mutate(sex = "b") %>%
 pivot_wider(names_from = sex, values_from = result:result_upr) %>%
 select(region, country_abbrev, result_b, sd_b, result_lwr_b, result_upr_b)
 }
 if (outcome %in% c("DALYrate")){
 outcome2 <- case_when(outcome == "DTHrate" ~ "DTH",
 outcome == "DALYrate" ~ "DALY",
 TRUE ~ NA_character_)
 dths_stroke_global <- df_in %>%
 filter(outcome == !!outcome2,
 year == !!year,
 cause == !!cause) %>%
 arrange(region, country_abbrev, year, sex, ilo_age_bands_5yr) %>%
 mutate(sd = (result_upr - result_lwr)/2/1.96) %>%
 left_join(who_ilo_long_populations, by = c("region", "country_abbrev", "year", "sex", "ilo_age_bands_5yr" = "age_bands_5yr")) %>%
 drop_na() %>%
 group_by(region, country_abbrev) %>%
 summarise(result = sum(result),
 sd = sqrt(sum((sd)^2)),
 population = sum(population)) %>%
 mutate(population = population,
 result = result / population * 100000,
 sd = sd / population * 100000,
[truncated: 157,632 more chars]
